# Hyperbolic adaptive spatial-aware multivariate time series anomaly detection

**DOI:** 10.1038/s41467-026-75881-1

**Published:** 2026-07-27

**Authors:** Jiaxin Han, Xuanrong Huo, Yuzhi Xiao, Zhonglin Ye, Yuhui Zheng, Haixing Zhao, Maosong Sun, Zhen Liu

**Affiliations:** 1 School of Computer, Qinghai Normal University, Xining, PR China; 2 State Key Laboratory of Tibetan Intelligent, Xining, PR China; 3 Key Laboratory of Tibetan Information Processing, Ministry of Education, Xining, PR China; 4 Qinghai Provincial Key Laboratory of Tibetan Information Processing and Machine Translation, Xining, PR China; 5 Tibetan Information Processing Engineering Technology Research Center of Qinghai Province, Xining, PR China; 6 Graduate School of Engineering, Nagasaki Institute of Applied Science, Nagasaki, Japan; 7 Qinghai Minzu University, Xining, PR China; 8 Tsinghua University, Beijing, PR China

**Keywords:** Computer science, Information technology

## Abstract

Existing multivariate anomaly detection methods suffer from practical industrial limitations stemming from distribution assumptions, volatile data and scarce reliable anomaly labels. This study proposes a hyperbolic adaptive spatial-aware multivariate anomaly detection method aimed at enhancing accuracy, robustness and interpretability in real-world applications. It first constructs an adaptive hyperbolic pre-training framework to embed coupled spatio-temporal hierarchical time-series features and mine latent cross-variable correlations. Next, an adaptive graph structure discovery and dynamic sparsification module removes rigid topology restraints, autonomously learning inherent data structures and pinpointing the spatio-temporal locations of anomalies for causal interpretation. A multi-level attention module and small-sample dynamic threshold algorithm further improve model stability when handling complex signals. Built upon self-adaptive graph topology, the unified end-to-end framework integrates anomaly recognition, diagnosis and interpretation with dedicated discrimination and correction mechanisms. Experiments demonstrate 10%–30% performance improvements across diverse detection benchmarks, verifying the method’s effectiveness and advancing interpretable AI research for anomaly detection.

## Abstract (web summary)

The authors propose an explainable multivariate anomaly detection framework that adaptively learns hyperbolic spatio-temporal structures, discovers dynamic graph topologies, and localizes anomaly causes. The method improves robustness, interpretability, and detection accuracy.

## Introduction

**Fig. 1:**
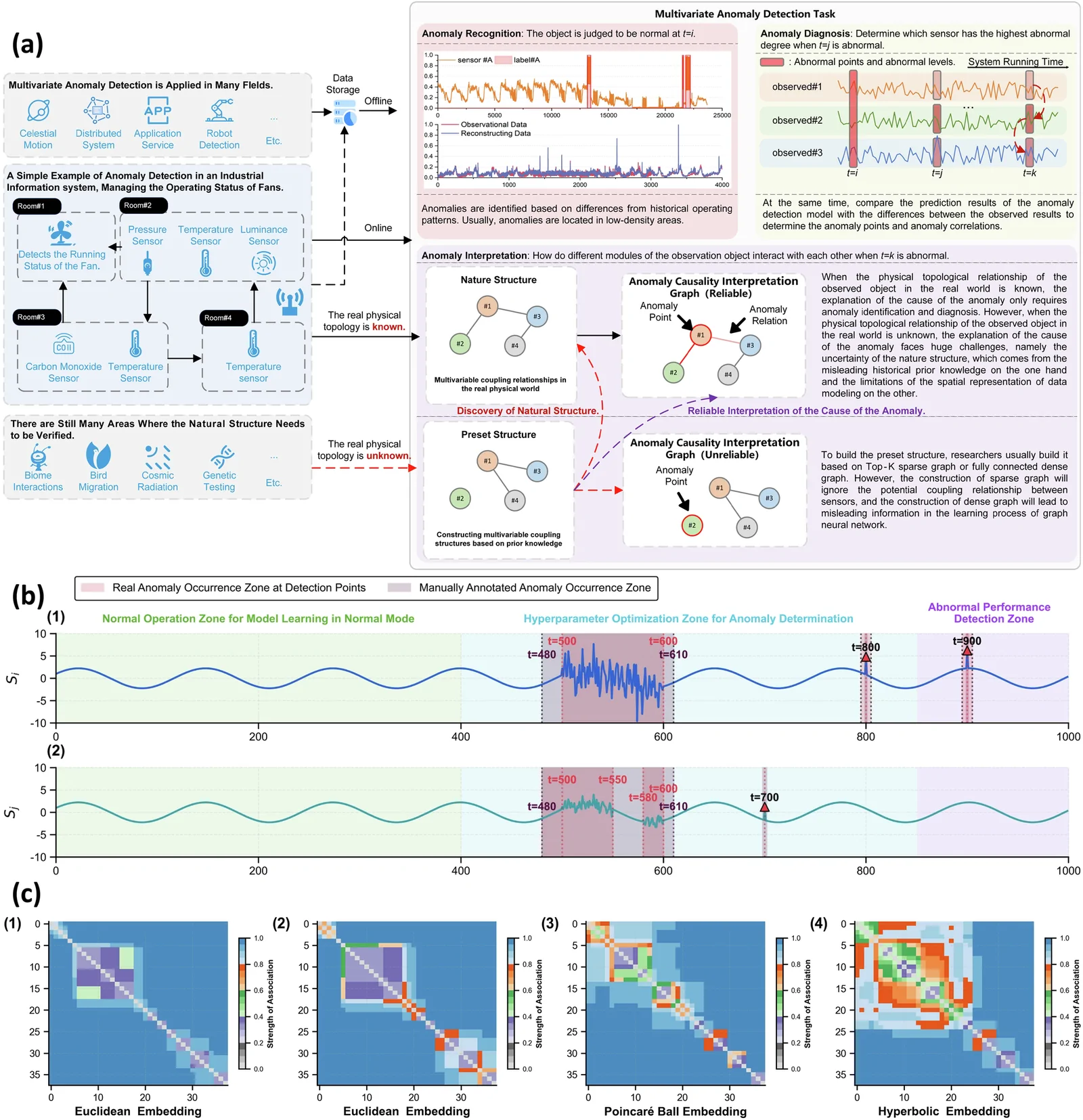
Multivariate anomaly detection and spatio-temporal dynamic analysis. **a** Anomaly detection framework. Multivariate anomaly detection evolves into a three-tier progressive framework. The recognition layer shifts anomalous segments from massive time series data. The diagnosis layer precisely locates spatio-temporal coordinates and determines thresholds. The interpretation layer reveals multilevel causal networks underlying anomalies and deep-seated systemic defects. This paradigm bridges the gap from observable phenomena to essential understanding, providing actionable decision support for continuous optimization of production systems. **b** Anomaly label reliability risks. Anomaly onset estimation limits often cause annotated anomaly regions to exceed true anomalous intervals. Segments appearing normal at small time scales may be mislabelled as anomalous over larger scales. Subtle point-level anomalies difficult to perceive are prone to omission. Mislabelling and insufficient labeling compromise objective performance assessment of high-precision models and further degrade the ability of high-performance models to correctly identify anomalies. **c** Observation window and spatial embedding effects. Using the SWaT dataset observation window size and spatial representation imposes dual constraints on resolving sensor coupling relationships. Euclidean embedding with a short window of 5 captures only local short-term coupling associations. Extended window of 110 reveals global long-range coupling patterns. Poincaré ball embedding accommodates hierarchical structures, distinguishing core and peripheral nodes. Hyperbolic space embedding enhances nonlinear non-Euclidean coupling representations, allowing accurate capture of weak long-distance couplings and better matching actual network topology, confirming superior adaptability over Euclidean embedding.

With the continuous advancement of Industrial Internet of Things (IIoT) and information systems, production monitoring sensors^1^ play a pivotal role in data acquisition and analysis during manufacturing processes, which is crucial for ensuring production safety and enhancing intelligent operation and maintenance^2^ (Supplementary Section 1). In current multivariate anomaly detection research tasks^3–5^, as shown in Fig. 1a, several challenges still exist, such as insufficient reliability of anomaly labels, unknown true system topologies, distorted data embedding spaces^6^ and conflated dimensions of anomaly interpretation. Moreover, over-reliance on structural priors has created an interpretation comfort zone trap that severely limits anomaly interpretability in dynamic scenarios^7^. Therefore, this study aims to construct an open adaptive mechanism of generalizing interpretation, enabling the maintenance of interpretive credibility and traceability precision even when unknown anomalies emerge. This is not only a key exploration toward advancing industrial cognitive intelligence from anomaly recognition to causal insight, but also it is critical to decision-making trustworthiness under complex operating conditions.

Challenge 1: credibility deficits in manual anomaly labeling. The credibility issue in multivariate anomaly labeling^8^ primarily stems from prevalent omission and mislabelling during manual labeling, as shown in Fig. 1b. Omission manifests at three levels: first, annotators’ subjective perception limits often lead them to misclassify subtle, potentially anomalous patterns that are difficult to detect visually as normal; second, labellers tend to mark only intervals with prominent signal deformation during system anomaly periods, whereas actual anomalies may have occurred earlier, resulting in a time lag in labeling; third, subsequent monitoring signals may exhibit similar deformation characteristics but remain unlabeled after an anomaly occurs. These unannotated anomalies can mislead the model in defining the anomaly-normal boundary, thereby reducing the accuracy, evaluation reliability and generalization capability of anomaly detection. Mislabelling arises from inherent flaws in the current labeling framework, such as the absence of unified, reliable criteria and fine-grained recognition tools, combined with excessive reliance on expert experience, which increases the risk of annotators misidentifying normal patterns embedded within anomalous regions as anomalies. This significantly hampers the reliability of root-cause interpretation in multivariate anomaly analysis. To address both insufficient labeling and mislabelling issues, we propose a rapid localization algorithm for anomaly spatio-temporal coordinates based on the system’s natural topology, which refers to the persistent connectivity pattern inherent to system elements, such as sensors and observation timestamps that emerge under normal operating conditions, as well as a fine-grained label correction algorithm. These aim to mitigate the limitations of manual labeling, enhance the interpretability of anomaly labels and improve the automatic discovery of anomaly patterns.

Challenge 2: unknown system natural topology. In open environments, the lack of explicit optimization objectives and domain expertise supervision makes it difficult to establish a natural structural representation that reflects the intrinsic nature of industrial scenarios. This issue is particularly evident in static models built upon prior assumptions (e.g., ϵ-radius, k-nearest neighbors)^9,10^, which struggle to adapt to the data’s natural topology, such as nonlinear dependencies and time-varying relationships. Consequently, false positive rates increase, and interpretative bias emerges. Although some studies attempt to adjust parameters via online learning^11^, the underlying topological structure remains rigid, preventing genuine data-adaptive structure formation. Such limitations are especially pronounced in non-stationary data streams and variable-dense systems, such as monitoring equipment undergoing gradual degradation^12^ and detecting distorted information in social networks^13^. In recent years, researchers have conducted extensive explorations into temporal dependencies, sensor couplings and heuristic learning in multivariate data^9,11,14,15^, developing topology evolution models that adapt to data. However, these approaches heavily rely on either known true topological relationships or substantial supervised information (e.g., nodes, edges, and graph functions^16–18^) as explicit optimization targets. Yet, the implicit constraints introduced by prior knowledge act as a filter on the data rather than enabling truly data-driven structural adaptation, which makes it difficult to uncover latent structural anomaly patterns in dynamic scenarios. These limitations, particularly acute in multivariate anomaly detection, stem from the scarcity of available topological optimization information. This motivates our investigation into discovering natural multivariate data structures without prior knowledge constraints, leading to the proposal of an adaptive hyperbolic graph structure discovery method that provides a reliable reference frame and objective evidence for interpreting the causes of multivariate anomalies.

Challenge 3: data embedding space distortion. Natural system structure discovery is constrained by geometric distortion in the data representation space^6^, which makes it difficult to effectively identify weak anomaly relationships, as shown in Fig. 1c. Early studies leveraging complex network theory^19–21^ successfully uncovered structural characteristics in physical systems, achieving significant breakthroughs in scenarios with well-defined entity relationships, such as epidemic propagation and computer virus spread^22–24^. However, when true system topologies are unavailable, such physics-based network models face applicability challenges. In multivariate anomaly detection, stacking multi-head attention modules or deepening temporal networks^14,25,26^ has been adopted to enhance sensitivity to weak anomalies and improve structural modeling capacity. Yet this strategy not only increases computational burden but also fails to address the fundamental bottleneck of feature representation. Meanwhile, graph representation learning^27^ captures latent structural features through data-driven approaches, but Euclidean space embeddings suffer from severe geometric distortion, markedly reducing node relationship identifiability. Consequently, researchers have exploited the superior geometric representation capacity of hyperbolic space^28,29^ to improve embedding quality. Nevertheless, graph representation learning relies on network data with known topological relationships. These research boundaries motivate us to consider how to match hyperbolic space with the geometric properties of node neighborhood structures and propose an adaptive hyperbolic pre-training framework that substantially enhances weak anomaly recognition capability.

Challenge 4: conflation of explanatory dimensions in anomaly causation. Existing multivariate anomaly detection models^9,10,14,30^ employ joint learning strategies to characterize spatio-temporal features of multivariate data. As a result, anomalous signals undergo coupled diffusion across both temporal and spatial dimensions, where dynamic perturbations in time-varying dependencies interweave with spatial constraints from static couplings, producing a causal masking effect. Current research on anomaly causation interpretation predominantly follows a structure-prior-driven framework^14,15,31,32^: first, an initial topology is constructed using association measures, such as directed distance matrices, similarity matrices, or covariance matrices between node pairs; next, edge weights are updated via message aggregation mechanisms to model the evolution of the system topology; finally, temporal dynamics and multivariate spatial couplings of nodes are jointly represented, enabling data reconstruction and anomaly causation interpretation. In this process, the model traces anomalies based on node weight magnitudes, implicitly assuming that high-weight nodes necessarily correspond to primary anomaly sources. However, this interpretation framework, rooted in a static structural isomorphism assumption, has serious limitations that the weight-driven mechanism^33^ cannot penetrate superficial association layers, essentially reducing complex system anomalies to a node importance ranking problem. This leads to causal confusion among heterogeneous anomaly sources, which makes it difficult to distinguish between endogenous weak anomaly accumulation (e.g., gradual equipment degradation) and exogenous sudden disturbances (e.g., malicious attacks or natural disasters). Despite potentially similar representations in terms of data reconstruction error, their physical origins and remediation strategies differ fundamentally. Therefore, we introduce a system topology adaptive discovery mechanism based on independent learning of spatio-temporal features, which significantly reduces mutual interference among causation interpretations.

Challenge 5: dynamic expansion of sensor scale. In recent years, the continuous growth in the number of Industrial Internet of Things sensors has made timeliness and robustness key performance indicators for anomaly detection models. Existing data-driven Transformer or RNN-based architectures for anomaly detection^14,25,31^ exhibit computational resource requirements that scale positively with sensor count, significantly degrading inference speed. Although graph representation learning has enabled structure discovery for million-scale nodes via link prediction^29^, such approaches rely on sampling or random walk strategies conditioned on known true topologies^34,35^, rendering them unsuitable as reliable references for system topology discovery. Therefore, we propose an anomaly detection architecture driven by data geometry. Compared with approaches that store historical variation patterns in additional memory units to mine latent topological attributes, leveraging Riemannian manifold geometry to capture natural data properties^36^ requires fewer computational resources, facilitating lightweight model design. Current research frameworks for studying geometric characteristics of data on Riemannian manifolds^37^ mainly fall into two categories: manifold-tangent-manifold mapping transformation strategies and end-to-end manifold-manifold operations. However, such hyperbolic composite learning units suffer from notable computational efficiency bottlenecks during training, specifically: Frequent conversions between hyperbolic space and approximate Euclidean space substantially increase computational overhead and severely limit the response speed of real-time inference. More critically, unsupervised anomaly monitoring shows obvious vulnerabilities to abrupt shifts in sensor measurements: dynamic thresholds fluctuate drastically and break monitoring continuity^38^; the method lacks sufficient adaptability to concept drift^30^ and complex sequence variations^8^; anomaly scoring mechanisms rely excessively on predefined normal benchmarks, which significantly degrades the fault tolerance of the whole system^39^. These drawbacks impair the overall robustness of models deployed in real-world industrial scenarios.

To address the aforementioned challenges, this study proposes a hyperbolic adaptive spatial-Aware multivariate time series anomaly detection model (HAO). By integrating a dual adaptive discovery strategy that combines hyperbolic space with natural data structure and introducing a task-driven dynamic sparsification method, the HAO model achieves efficient and reliable multivariate anomaly detection and causation interpretation. It effectively resolves key issues, such as insufficient credibility of anomaly labels, unknown true system topology, distortion in data embedding space and conflation of anomaly interpretation dimensions. The main contributions of this study are as follows:

This study establishes an adaptive hyperbolic space representation learning framework, which overcomes the inherent drawbacks of conventional methods dependent on predefined prior structures and Euclidean space representations. This study further proposes a collaborative mechanism integrating adaptive unsupervised hyperbolic graph structure discovery (AH-GSD) and adaptive hyperbolic reconstruction-prediction pre-training framework (AH-PH). On the one hand, AH-GSD enables low-loss dynamic generation and sparsification of natural structures of multivariate data, removes rigid restrictions imposed by manually predefined priors, and autonomously identifies and collaboratively localizes spatio-temporal anomaly correlations. On the other hand, AH-PH dynamically formulates reconstruction rules adaptable to complex data distributions and optimizes hyperbolic linear layers as well as graph convolutional layers, thereby substantially boosting computational efficiency and embedding quality. This work successfully deploys hyperbolic space theory for unsupervised adaptive graph structure discovery and constructs a high-quality fundamental embedding space for multivariate data analysis.

This study proposes a self-feedback anomaly detection research framework covering multivariate anomaly benchmark construction, anomaly recognition, anomaly diagnosis and anomaly interpretation. In terms of data annotation, we design an automatic anomaly annotation and correction framework. We utilize the geometric differences between natural topology and predicted topology, as well as inherent anomaly distribution characteristics, to implement coarse-to-fine label refinement and establish interpretable annotation criteria and quantitative evaluation benchmarks. In terms of anomaly interpretation, we put forward a cross-domain anomaly cause analysis method. By deeply mining inherent spatio-temporal natural structures, this method realizes accurate anomaly traceability and effectively compensates for the limitation that existing studies only focus on local features while ignoring global complex system coupling relationships. This work is an exploration of explainable artificial intelligence in multivariate anomaly detection, providing an effective analytical approach to reveal the deep internal mechanism of multivariate anomalies.

## Results

### Experimental setup

Symbol definitions are provided in Supplementary Section 2. Details on the multivariate anomaly detection benchmark datasets are provided in Supplementary Section 4. Data preprocessing procedures are elaborated in Supplementary Sections 5.1 and 5.2. Model evaluation metrics are illustrated in Supplementary Sections 5.3 and 6. Experimental hyperparameters and analytical configurations are presented in Supplementary Sections 5.4, 5.5, and 5.6. Comparative baselines for multivariate anomaly detection are listed in Supplementary Section 5.7.

### Multivariate anomaly recognition

In multivariate anomaly detection research, a model’s robustness to labeling quality and its generalization ability across diverse scenarios are key challenges. To address the problem that existing models are vulnerable to labeling omissions and mislabeling, this study focuses on the analytical performance of the HAO-series model. Through multi-dataset experiments, its performance stability and anomaly recognition effectiveness under different labeling states are verified, which provides a more reliable solution reference for multivariate anomaly detection in complex scenarios.

#### Comparative experiments

**Fig. 2:**
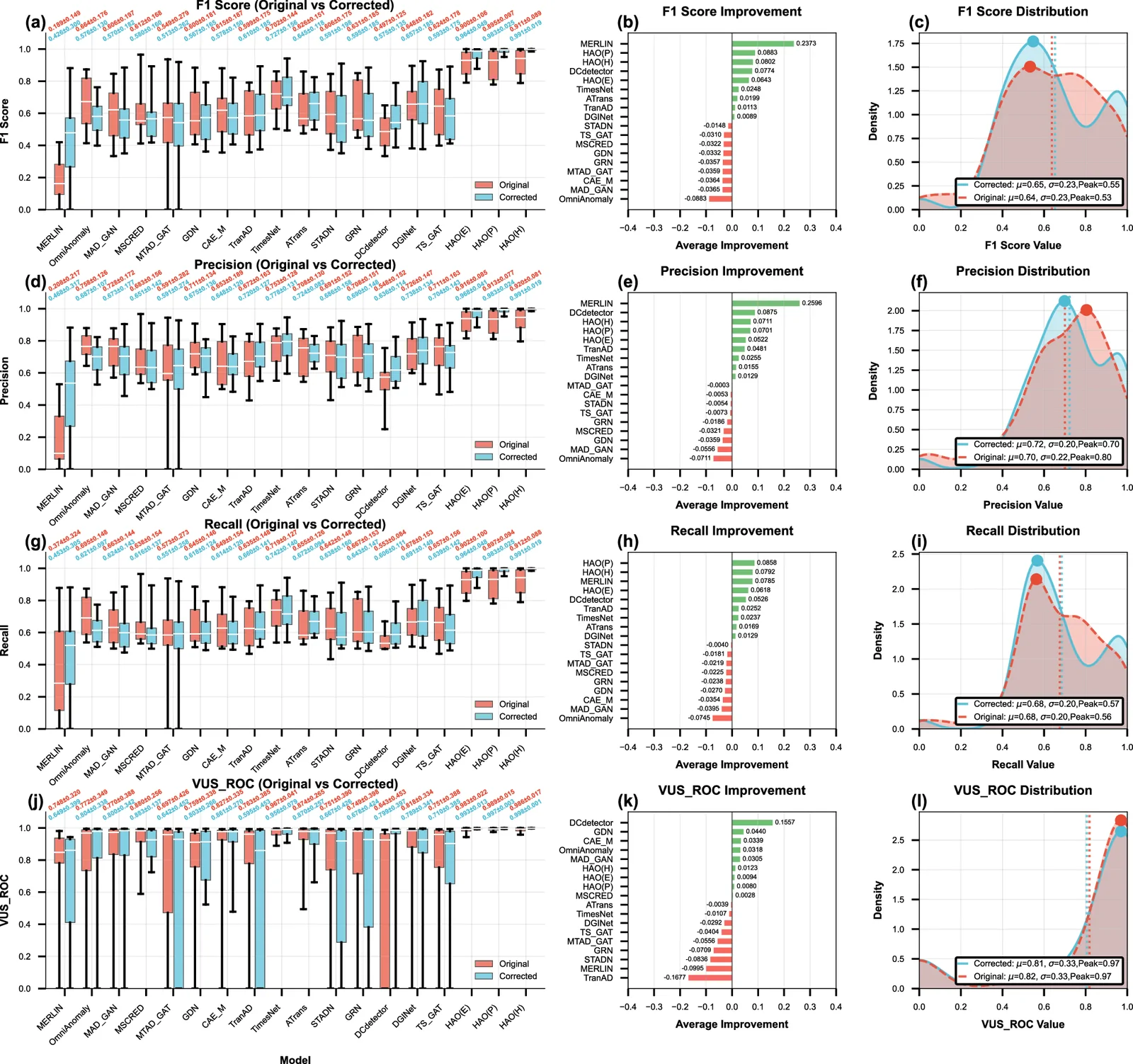
Comprehensive comparative analysis of multivariate anomaly detection model performance. **a**, **d**, **g**, **j** Show box plots of average performance across different datasets. **b**, **e**, **h**, **k** Show the performance improvement effects. **c**, **f**, **i**, **l** Show the performance density distributions.

The comprehensive F1-score performance of multivariate anomaly detection models before and after anomaly label correction is shown in Fig. 2a, b. Before correction, the F1-score of the HAO series models ranges from 0.8995 to 0.9112, which is significantly better than those of traditional models, ranging from 0.5489 to 0.7017. Specifically, the F1-score of HAO(H) is 0.9112, which is 0.2095 higher than the 0.7017 achieved by the best traditional model TimesNet^40^. Meanwhile, the F1-score gain of HAO(H) is 0.0802. Although this value is lower than 0.2373 compared with the F1-score of MERLIN^41^, the F1-score of HAO(H) is 4.8 times that of MERLIN before correction, indicating that the MERLIN model based on local numerical pattern matching is highly dependent on data annotation quality. As a result, MERLIN achieves an F1-score improvement of 0.2373 after label correction. After correction, the F1-scores of the HAO series models increase to between 0.9638 and 0.9914, with F1-score gains ranging from 0.0643 to 0.0883. In contrast, most traditional models have a decline in F1-score after label correction. For instance, the F1-score of OmniAnomaly^38^ drops from 0.6640 to 0.5758, with a decrease of 0.0883. Transformer-based models, including TimesNet, ATrans^42^, DCdetector^43^, and TranAD^25^, achieve significant performance gains after label correction, demonstrating their strong ability to capture temporal dependencies and anomalies. However, these models lack robustness to label noise and depend highly on accurate supervision. This demonstrates that the HAO series models are less sensitive to the limitations of anomaly labeling, and they can achieve effective performance on higher baseline models. The above differences arise from the dynamic adaptation and self-correction capabilities of the HAO series model architecture for cross-scenario data, which enable stable performance without relying on high-quality anomaly labeling. In comparison, traditional models, such as OmniAnomaly based on Euclidean space data representation, MAD-GAN^44^ based on static adversarial architecture and GDN^9^ based on static graph structure optimization, struggle to effectively handle noise disturbances in anomaly labeling due to issues, such as distorted geometric representation or fixed structural design. This leads to the degraded anomaly detection performance after label correction.

The precision and recall performance before and after label correction are shown in Fig. 2d, e, g, h. The HAO series models achieve significant improvements in both precision and recall. The average precision is 0.9163 before correction, and it increases to 0.9808 after correction, which represents an improvement of 0.0645. The average recall is 0.9039 before correction, and it increases to 0.9794 after correction, which represents an improvement of 0.0755. These results demonstrate that the HAO model architecture can dynamically adapt to labeling noise and effectively avoid performance degradation caused by labeling bias in traditional models. Specifically, HAO(H) achieves a recall of 0.9914 after correction, which represents an improvement of 0.0711 over the recall before correction, with a false negative rate below 1%. Its graph structure learning algorithm accurately identifies weak-signal anomalies and missed anomalies by deeply mining the dependencies among multivariate variables. Meanwhile, the hyperbolic space signal amplification mechanism further enhances the sensitivity to anomaly signals. These advancements effectively overcome the limitations of traditional models, such as insufficient generalization ability of MERLIN due to over-reliance on local numerical matching, inadequate detection of the weakly correlated anomalies by CAE-M^45^ with fixed memory networks, as well as unstable recall performance caused by the rigid GRU structure of GRN and the static threshold strategy of DGINet^32^ due to structural rigidity and over-dependence on distribution assumptions. In contrast, traditional models are highly sensitive to annotation quality. For example, OmniAnomaly, based on Euclidean space data representation and reconstruction, MAD-GAN and GDN, based on static structure learning and optimization lack the ability to identify annotation noise. They tend to misclassify mislabeled samples as normal samples after correction, which leads to an increased false negative rate. Their average precision and average recall decrease by 0.0542 and 0.0470, respectively, which exposes their inherent limitations in geometric space assumptions or static structure design. In addition, MERLIN is more sensitive to interpretable anomalies due to its detection mechanism, and its precision improvement reaches as high as 0.2596 after correction. The precision of ATrans, DCdetector and TimesNet increases by 0.0155, 0.0875, and 0.0255, respectively, after label correction. These improvements reflect that the label correction method proposed in this paper provides reliable and accurate supervision signals and helps these models give full play to their inherent detection performance. This indirectly reflects that the HAO series models can effectively analyze the original structural variations caused by anomalies and eliminate false anomaly signals, thus achieving a more interpretable anomaly detection mechanism. However, traditional models, such as the VAE reconstruction mechanism of OmniAnomaly and the adversarial generation mechanism of MAD-GAN, lack the ability to identify annotation noise. They tend to misjudge mislabeled samples as anomalies after correction, which results in decreased precision.

Label correction significantly improves the overall performance stability and average F1-score of multivariate anomaly detection models, as shown in Fig. 2c. After label correction, the mean F1-score of the anomaly detection models increases from 0.64 to 0.65, and the standard deviation remains at 0.23. This indicates that label correction effectively improves the overall detection accuracy while maintaining stable data dispersion. Although the distribution before label correction has a higher peak value of 0.74, and the peak value after label correction is 0.55, which shows that high-score samples are more concentrated in the original data, the distribution becomes more balanced after label correction and forms a new secondary peak near 0.9. This demonstrates that the multivariate anomaly detection models after label correction are more robust across different performance ranges, and they can adapt to a wider variety of anomaly patterns. The stability and average performance of precision and recall for multivariate anomaly detection models are shown in Fig. 2f, i. Label correction significantly improves the precision of model recognition. The mean precision increases from 0.70 to 0.72, with an increase of approximately 2.86%. Meanwhile, the standard deviation decreases from 0.22 to 0.20, with a reduction of 9.1%. This indicates that both the accuracy and stability of multivariate anomaly detection results are effectively enhanced. For recall, the mean value remains stable at 0.68 before and after correction, and the standard deviation stays at 0.2 with only a slight adjustment in the peak value. This shows that the multivariate anomaly detection models optimize the performance of reducing false positives while maintaining low false negatives. Finally, the multivariate anomaly detection models achieve the goal of effectively improving precision and result consistency while preserving the stability of higher recall.

VUS_ROC^46^ performance before and after label correction is shown in Fig. 2j–l. The HAO series models maintain VUS_ROC values above 0.98 throughout label correction and exhibit small performance fluctuations across datasets. The DCdetector model achieves considerable performance improvement but lacks sufficient stability across different scenarios. It is worth noting that ATrans and TimesNet achieve gains in F1 score, precision, and recall in Fig. 2b, e, h while suffering a decline in VUS_ROC in Fig. 2k. This contradiction arises because point‑wise indicators for discrete classification directly benefit from refined anomaly boundaries generated by label correction. VUS_ROC evaluates global ranking performance under continuous thresholds and is less disturbed by threshold selection strategies. It thus reveals structural limitations of models that rely on static attention, frequency domain periodic modeling, or contrastive learning priors and show weak generalization ability under clean labels. The density distribution in Fig. 2l confirms this trend. The overall average VUS_ROC decreases slightly from 0.82 to 0.81, which is caused by the performance degradation of unstable models.

#### Comparative analysis based on ROC curves and AUC values

The ROC curves and corresponding AUC values^25^ for the multivariate anomaly detection task are shown in Supplementary Fig. 8. The performance of the same model varies drastically across different datasets, which makes general adaptation difficult to achieve. For instance, MAD-GAN achieves an AUC score of 0.9154 on the Internet server cluster dataset SMD^38^, but only 0.5045 on the industrial water treatment plant intrusion detection dataset WADI^47^ and as low as 0.5570 on the water pollution quality detection dataset GECCO^48^, which sufficiently demonstrates that MAD-GAN exhibits severely limited cross-scenario anomaly detection capability. Even GRN^14^, which has strong temporal modeling ability, obtains an AUC of 0.8309 on the eBay Web server dataset PSM^49^, yet its AUC values on other industrial anomaly datasets, including Power System^50^, SKAB^51^, and GPT^52^ are all less than 0.55. As shown in Supplementary Table 2, where datasets are categorized by anomaly detection difficulty, the performance of baseline models exhibits a clear gradient correlation with dataset difficulty. Specifically, the average AUC values of baseline models on high-difficulty datasets, such as CICIDS, SKAB, and WADI, range only from 0.5066 to 0.5882; those on medium-difficulty datasets, including MSL^53^, SMAP^53^ and PSM, range from 0.6165 to 0.6945; and those on low-difficulty datasets, including SMD, SWaT^54^ and Synthetic^55^, range from 0.7417 to 0.8548. This pattern is highly correlated with dataset dimensionality and the complexity of anomaly types, which reflects the significant constraining effect of dataset difficulty on model performance.

Notably, when the design characteristics of a model match the background, dimensionality and anomaly types of a dataset, multivariate anomaly detection models may achieve relatively superior results; otherwise, the detection performance drops sharply. For example, MERLIN excels at univariate anomalous subsequence recognition and achieves an AUC of 0.9418 on the natural gas pipeline dataset GPT, but only 0.4650 on the larger-scale Internet application server dataset ASD. DGINet is suitable for high-noise industrial time series and achieves an AUC of 0.9331 on the Synthetic dataset, but only 0.7001 on the WADI dataset. ATrans models temporal association discrepancies through a two-branch anomaly-attention mechanism, achieving an AUC value of 0.8261 on the Synthetic dataset with prominent temporal features and 0.7943 on the high-dimensional and strongly coupled PSM dataset. GDN is adept at modeling dependencies among variables and ranks first among baseline models on both the CICIDS and GECCO datasets, with AUC values of 0.6331 and 0.6111, respectively. In sharp contrast to the baseline models, the HAO-series models demonstrate stronger generalization ability and full-scenario adaptability for multivariate anomaly detection. Specifically, the Euclidean version, Poincaré ball version and hyperbolic version of HAO all achieve significantly higher AUC values than baseline models across all datasets, which remain above 0.84 overall, while they exhibit a stable performance gradient that HAO(H) > HAO(P) > HAO(E). This validates the value of non-Euclidean space embedding for improving multivariate anomaly detection.

#### Comparative analysis based on precision–recall curves and AP values

The precision–recall (P–R)^46^ curves and average precision (AP) values for the multivariate anomaly detection task are shown in Supplementary Fig. 9. The baseline models exhibit limited ability to balance precision and recall in multivariate anomaly detection, with overall lower AP values and significant cross-dataset fluctuations. For example, TranAD achieves an AP of 0.8728 on the SWaT dataset for water treatment plant intrusion detection, but only 0.5001 on the CICIDS dataset for internet intrusion detection. Even the superior MTAD-GAT^56^ obtains an AP of 0.9134 on the Synthetic dataset, which is a simulated power grid dataset, yet it is only 0.6130 on the real-world Power System dataset, which shows a substantial performance degradation. Such performance discrepancies are closely related to the design mechanisms of the baseline models. For instance, MERLIN relies on numerical pattern matching and fails to effectively model the complex coupling effects among multiple variables. OmniAnomaly adopts Euclidean space embedding, which cannot fully capture the underlying nonlinear characteristics of multivariate joint distributions, which makes it difficult to simultaneously improve precision and recall in complex scenarios. The AP values of the baseline models tend to decrease with the increase in anomaly type diversity and data dimensionality. The baseline models can achieve a relatively favorable P–R balance on datasets with simple anomaly patterns and low dimensionality, but they suffer from significant performance degradation on datasets with diverse anomalies and higher dimensionality. Specifically, the AP of the optimal comparison model DGINet is 0.8699 on the SMD dataset, which is a low-dimensional internet application server cluster dataset with single anomaly patterns. The AP of the optimal comparison model MTAD-GAT is 0.6893 on the ASD dataset, which is a medium-dimensional internet application server cluster dataset containing point and regional anomalies. In contrast, the AP of the optimal comparison model TS-GAT^31^ is only 0.6262 on the CICIDS, which is a high-dimensional network intrusion detection dataset with multiple anomaly types. The main reason for this trend is that the baseline models lack the ability to disentangle high-dimensional features and struggle to adapt to the complex spatio-temporal coupling relationships of diverse anomalies, thereby impairing the synergistic performance between precision and recall. On the CICIDS dataset, TimesNet, ATrans, and DCdetector obtain APs of 0.6224, 0.7141, and 0.5581. All three models degrade with higher dimensionality and anomaly complexity, revealing challenges from high-dimensional intricate patterns instead of architectural representation bottlenecks.

In addition, the performance of baseline models on imbalanced anomaly detection datasets highly depends on their natural design logic. For example, MERLIN focuses on anomalous subsequence recognition and achieves the best AP of 0.8957 among baselines on the GPT dataset, which is a gas pipeline intrusion detection dataset dominated by contextual anomalies, but only 0.4883 on the ASD dataset with strongly coupled multivariate anomalies. DGINet integrates dynamic graph attention and the Informer module and is adept at handling high-noise time series; it achieves the best AP of 0.8699 among baselines on the SMD dataset, but only 0.7794 on the SWaT dataset. MTAD-GAT models multivariate dynamic dependencies via dual graph attention layers and attains an AP of 0.9134 on the Synthetic dataset, yet only 0.5992 on the SMD dataset with simple anomaly patterns. Models such as MAD-GAN and MSCRED exhibit insufficient capability in modeling spatio-temporal correlations for high-dimensional data with multiple anomalies, with AP values below 0.6 on most datasets, which indicates limited scenario applicability. In contrast, the HAO-series models demonstrate a more robust balance between precision and recall in the multivariate anomaly detection task. The performance gradient further validates that non-Euclidean space embedding plays a significant role in improving the balance between precision and recall. More importantly, the spatio-temporal anomaly-aware interpretable label correction method proposed in the HAO-series models can not only discover potential anomaly patterns but also enhance the model’s sensitivity to weak anomaly patterns, which is the key to achieving superior performance in both precision and recall on the P–R curves.

#### Ablation study and stability analysis

**Fig. 3:**
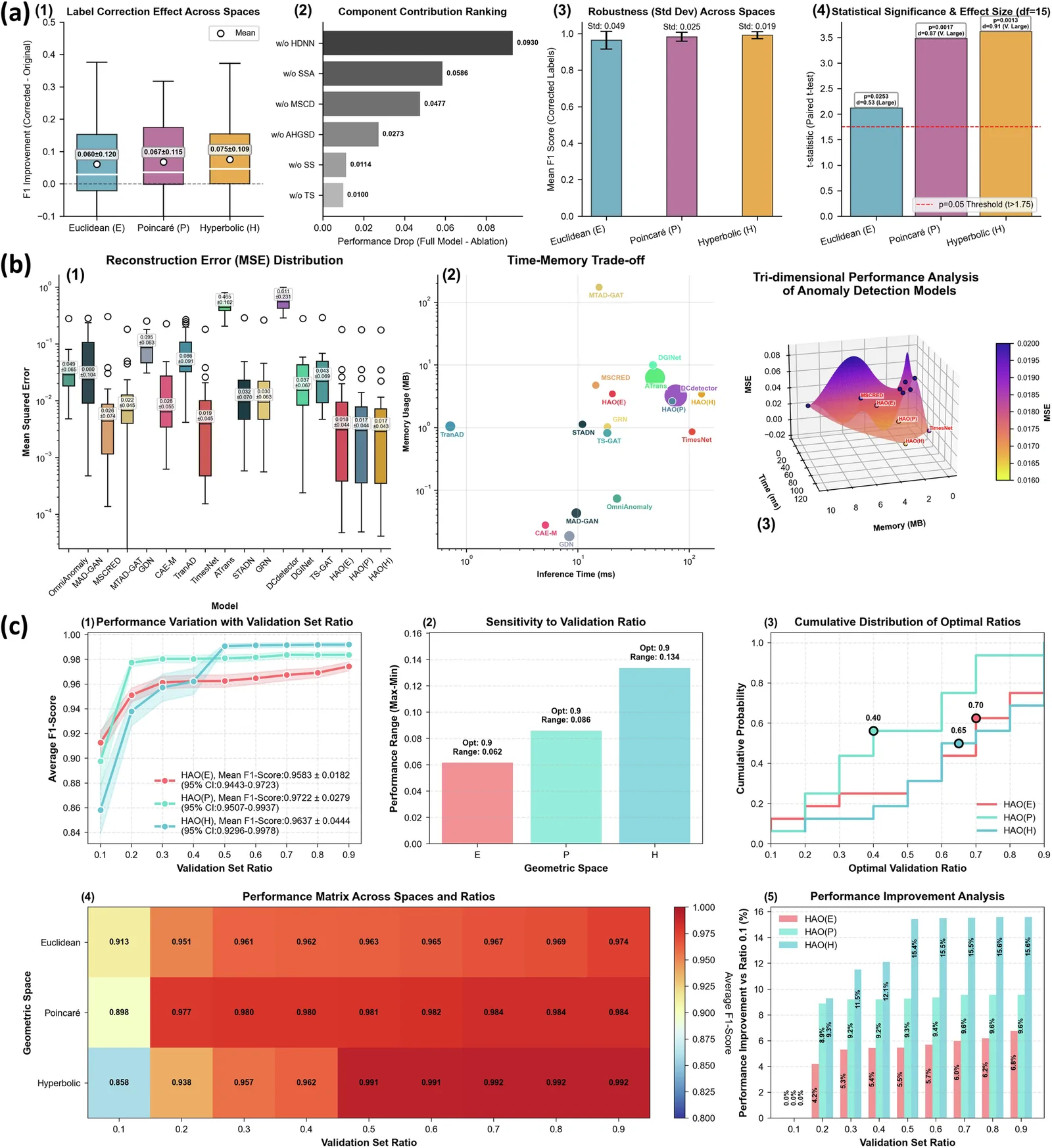
Ablation and stability analysis of the HAO model, spatio-temporal complexity, and sensitivity analysis. **a** Ablation study and stability analysis of the HAO model. **a(1)** Shows performance changes after removing each core component, where w/o indicates removal of the corresponding module: w/o HDNN removes the geometric learning module; w/o MSCD removes the multi-level signal parsing attention mechanism module; w/o AHGSD removes the spatio-temporal adaptive hyperbolic graph structure discovery module; w/o SSA removes the natural graph dynamic sparsification module; w/o TS removes the multivariate temporal natural structure discovery module; w/o SS removes the multivariate coupling structure discovery module. **a(4)** Shows the results of two-sided paired t-tests and corresponding effect sizes for performance differences between models in three geometric spaces and the baseline model. The effect size is reported as Cohen’s d, computed using the formula: $d=\overline{D}/{{\mathrm{SD}}}_{D}$, where $\bar{D}$ is the mean of performance differences between the proposed model and the baseline across all datasets, and ${{\mathrm{SD}}}_{D}$ is the standard deviation of these differences. **b** Spatio-temporal complexity–reconstruction error and three-dimensional performance analysis of multivariate anomaly detection models. **c** Sensitivity analysis of validation set proportion hyperparameter for the HAO model.

The label correction strategy designed for the HAO model series to address the prevalent issues of missing and false labels in multivariate anomaly detection datasets effectively improves the F1-score across different data representation spaces, with the magnitude of improvement showing a gradually increasing trend as the geometric representation capacity of the space strengthens, as shown in Fig. 3a(1). Specifically, the HAO model achieves an F1-score improvement of 0.060 for data embedding in the Euclidean space, 0.067 in the Poincaré ball space and up to 0.075 in the hyperbolic space. The F1-score enhancement of the HAO model series is closely associated with the complex nonlinear spatio-temporal coupling characteristics in multivariate time series. The geometric structure in the hyperbolic space better matches the inherent hierarchical and community organization of multivariate data, which enables more accurate capture of potential anomalies triggered by spatio-temporal structural variations and thus yields the most significant correction effect in this space. In contrast, the Euclidean space cannot fully characterize the complex spatio-temporal coupling relationships among variables, and data representation is prone to distortion, which limits the ability to identify labeling errors and results in a relatively limited label correction effect.

Unlike traditional models that perform embedding based on Riemannian manifold linear layers, the HAO model deeply integrates the geometric representation of multivariate data into all components of graph structure discovery, which aims to minimize data representation distortion and improve the geometric compatibility between the data representation space and the structure of multivariate data. As shown in Fig. 3a(2), ablation experiments on the six core components of the HAO model demonstrate that removing any component leads to performance degradation and the magnitude of the drop reflects the hierarchical differences in the importance of each component. Among them, the hyperbolic linear transformation layer (HDNN) serves as the core supporting component, and its removal causes the largest F1-score drop, even exceeding the drop caused by removing components, such as the small-sample dynamic threshold determination algorithm (SSA), multi-level signal parsing attention mechanism (MSCD) and adaptive hyperbolic graph structure discovery (AHGSD). The multivariate coupling natural structure discovery (SS) and multivariate temporal dependency natural structure discovery (TS) act as auxiliary core components, with relatively minor performance losses upon removal. These results reveal that the core logic of data representation and embedding space compatibility in multivariate anomaly detection lies in the geometric properties of data, which are fundamental to capturing spatio-temporal coupling relationships. Through dynamic adjustment and optimization of spatial geometric properties guided by Riemannian manifold curvature, the HDNN effectively alleviates geometric representation distortion encountered in conventional models. The other components of the HAO model provide support from the perspectives of data reconstruction, feature disentanglement and structure discovery, respectively, which form a synergistic effect with HDNN. It should be noted that although traditional models widely adopt HDNN-like data representation strategies in Riemannian manifold-based learning, achieve significant performance improvements over Euclidean space representation and have been widely applied in dynamic graph learning, community detection and recommender systems^57^, they typically treat graph structure learning only as a means to enhance data representation capability and lack a deep graph structure discovery mechanism.

The robustness of the F1-score after label correction by the HAO model series in various anomaly detection scenarios is shown in Fig. 3a(3). The stability of the F1-score achieved by the HAO model exhibits significant gradient differences across different data representation spaces. The standard deviation of the average F1-score is 0.049 for the HAO model with Euclidean space representation, 0.025 with Poincaré ball representation and 0.019 with hyperbolic space representation. Among various anomaly detection application scenarios, the HAO model series achieves the best anomaly detection performance in the hyperbolic space, which indicates that the hyperbolic space can better adapt to the geometric characteristics of data in different anomaly detection scenarios and effectively alleviate the poor generalization caused by the structural rigidity of traditional models. In contrast, its performance fluctuates greatly, and the robustness is insufficient when facing changes in anomaly detection scenarios due to the geometric limitations of data representation capability in the Euclidean space. These results demonstrate that the stronger the geometric representation capability of the embedding space, the higher the stability of the model on diverse datasets. The reliability of the label correction strategy of the HAO model has been verified. The paired t-test results are shown in Fig. 3a(4). The HAO model series passes the statistical significance test in all applied embedding spaces. The *p*-value of the HAO model is 0.0013 in the hyperbolic space, 0.0017 in the Poincaré ball space and 0.0253 in the Euclidean space, all of which are below the significance level threshold of 0.05, with the highest significance in the hyperbolic space. Labeling errors in high-difficulty and high-dimensional datasets are more subtle. By virtue of its ability to accurately capture multivariate nonlinear correlations, the hyperbolic space can identify labeling errors that are closer to the real distribution, which results in more prominent statistical significance, which further confirms the practical value of the proposed strategy in compensating for labeling defects.

Combined with the ablation experimental results, the label correction strategy of the HAO model effectively alleviates the labeling defects in multivariate datasets. Specifically, the core component HDNN serves as the main support for performance by optimizing geometric representation, while the geometric advantages of the hyperbolic space further strengthen the comprehensive performance of the model in terms of high performance, strong generalization and high robustness. These findings indicate that geometric design based on the intrinsic nature of multivariate data and the synergy among components are key directions for improving the practical value of multivariate anomaly detection models.

#### Spatio-temporal complexity and reconstruction error analysis

The data reconstruction error of the HAO model series is significantly lower than that of the baseline models, and the reconstruction error exhibits a gradually decreasing trend as the geometric representation capacity of the embedding space improves, as shown in Fig. 3b(1). Among all comparison methods, TimesNet achieves the best performance with a reconstruction error of 0.0039. In contrast, the reconstruction error of the HAO model is 0.0031 in the Euclidean space, 0.0030 in the Poincaré ball space and 0.0029 in the hyperbolic space, which represents a reduction of 34.09% compared with MSCRED. Considering that multivariate time series exhibit strong spatio-temporal coupling and nonlinear correlations, which impose higher requirements on reconstruction accuracy, the HAO model series achieves high-precision reconstruction by introducing a multi-level signal parsing attention mechanism. It further mines underlying data patterns by combining geometric prior learning and dynamically adjusts the embedding space based on Riemannian manifold curvature, which effectively alleviates the common feature distortion issue in traditional methods and thus yields superior reconstruction performance.

In addition, the HAO model series achieves a favorable trade-off between inference time and memory consumption (Supplementary Section 5.8). As shown in Fig. 3b(2), the inference time and memory usage of the HAO model series remain at a moderate level. TimesNet has inference time and memory usage close to HAO(H). Its frequency-domain transformation and 2D convolution bring higher time overhead but reasonable memory consumption. Despite similar spatio-temporal complexity, HAO(H) achieves better performance. This advantage originates from architecture-level optimizations, including an independent learning structure of spatial and temporal features effectively simplifies high-dimensional feature disentanglement and computational procedures. Meanwhile, the dynamic graph structure discovery algorithm reduces redundant topology modeling, significantly decreasing computational and storage costs while maintaining detection performance. For further comprehensive performance analysis, the performance of the HAO model series in the three-dimensional space composed of inference time, memory usage and reconstruction error is shown in Fig. 3b(3). The HAO-series models consistently lie within the optimal performance set and are the only multivariate anomaly detection models that simultaneously achieve lower spatial complexity, lower reconstruction error and higher detection performance. In contrast, some baseline models sacrifice performance in other dimensions by over-optimizing a single metric. For instance, MSCRED^55^ sacrifices spatio-temporal efficiency to improve detection effectiveness, while GDN suffers from degraded reconstruction and detection performance due to excessive computational simplification. Notably, although the inference time of the HAO model series increases slightly with performance improvement, its scalability remains controllable. Specifically, under the Euclidean space representation, the HAO model achieves the fastest inference speed with low memory usage and acceptable reconstruction error owing to its simple geometric structure. After introducing curved geometric constraints in the Poincaré ball space, the inference time rises moderately, but the reconstruction accuracy and detection performance are simultaneously improved. In the hyperbolic space, the model adopts a dynamic curvature adjustment mechanism and high-order geometric prior learning to achieve optimal reconstruction and detection results, which increases computational steps and results in the longest inference time among the three variants. This evolutionary trend indicates that the performance improvement of the HAO model series does not rely on simple computational overhead stacking, but it is achieved through the organic integration of natural geometric structures and multi-task collaborative optimization, which leads to cost-controllable performance expansion. This characteristic enables the HAO model series to present potential for real-world engineering deployment in multivariate anomaly detection scenarios with diverse real-time requirements.

#### Hyperparameter analysis of dataset split ratios

The F1-score of the HAO-series models shows a trend of rising first and then stabilizing as the validation set ratio increases from 0.1 to 0.9, as shown in Fig. 3c(1). When the validation set ratio is initially 0.1, the F1-score of the HAO model is 0.858 in Euclidean space, 0.898 in the Poincaré ball space and 0.913 in hyperbolic space. When the validation set ratio reaches 0.9, the F1-score of the HAO model increases to 0.974 in Euclidean space, 0.984 in the Poincaré ball space and 0.992 in hyperbolic space. Among these, the F1-score improvement of the HAO model in hyperbolic space exceeds 8.65%. These results indicate that complex spatio-temporal coupling and nonlinear features exist in multivariate datasets, and sufficient validation data facilitates the optimization of geometric parameters and the dynamic construction of graph structures. Benefiting from stronger geometric representation capability, hyperbolic space achieves higher utilization efficiency of validation data, thus exhibiting superior performance at the initial stage and reaching performance saturation more rapidly in the high-ratio range. When measuring the sensitivity to the validation set ratio based on the performance fluctuation range (Max-Min F1-score), as shown in Fig. 3c(2), the HAO-series models display obvious gradient differences. Specifically, the F1-score fluctuation of the HAO model in hyperbolic space is only 0.062, which is significantly lower than 0.134 in Euclidean space and 0.086 in the Poincaré ball space, which demonstrates that the HAO model achieves the most stable F1-score performance in hyperbolic space. Since real-world datasets often suffer from distribution heterogeneity and diverse anomaly types, variations in the validation set ratio tend to affect the stability of the learning process. Hyperbolic space can more accurately capture the natural geometric structure of data, thereby reducing the dependence on large-scale validation data and yielding smaller fluctuations. In contrast, Euclidean space is more susceptible to variations in data volume due to its limited geometric expressiveness, which results in weaker stability.

The cumulative distribution curves of the HAO-series models, as shown in Fig. 3c(3), reveal that the probability of achieving optimal performance gradually increases with a larger validation set ratio. This finding suggests that a higher validation set ratio provides more representative samples, covering a wider range of anomaly types and patterns. Key modules in the HAO model, such as dynamic graph structure discovery and geometric parameter optimization, rely on sufficient data for iterative refinement. The geometric advantages of hyperbolic space further enhance the utilization efficiency of high-proportion validation data, which leads to higher and more stable performance in the optimal ratio interval. We further construct a joint performance matrix of embedding dimension and validation ratio, as shown in Fig. 3c(4). The HAO model achieves the highest F1-score in hyperbolic space across the validation set ratio range of 0.1–0.9, followed by the Poincaré ball space and then Euclidean space. Moreover, the performance gap in anomaly detection among different data representation spaces gradually widens as the validation set ratio increases. For instance, at a validation set ratio of 0.5, the F1-score of the HAO model is 0.991 in hyperbolic space, 0.981 in the Poincaré ball space and 0.963 in Euclidean space, with a maximum performance gap of 0.028 among the HAO-series models. At a validation set ratio of 0.9, the F1-score reaches 0.992 in hyperbolic space, 0.984 in the Poincaré ball space and 0.974 in Euclidean space. The F1-score difference between hyperbolic space and the Poincaré ball space is 0.018, and that between the Poincaré ball space and Euclidean space is 0.019. This indicates that the model, given the complex feature structure in multivariate data, can fully exploit its architectural advantages only under sufficient data conditions. A higher validation set ratio provides a more solid foundation for dynamic graph structure discovery and geometric parameter optimization. Particularly in hyperbolic space, sufficient data support enables more precise tuning of the Riemannian manifold curvature, and the dynamic graph module can mine potential correlations more comprehensively, which makes the performance advantage increasingly prominent with a growing data ratio.

Regarding the performance improvement magnitude of the HAO-series models, as shown in Fig. 3c(5), the performance increment relative to the validation set ratio of 0.1 shows a trend of fast initial growth followed by gradual slowdown. The HAO model in hyperbolic space achieves the most significant improvement within the low validation set ratio range of 0.1–0.2, with an increase of up to 15.6% from 0.1 to 0.2, and the overall cumulative improvement is the highest. When the ratio of the validation set is ≥0.5, the F1-score improvement of the HAO model in hyperbolic space stabilizes at approximately 9%. When the validation set ratio reaches 0.9, the cumulative F1-score improvement is 9.6%, which is higher than 9.3% in the Poincaré ball space but lower than 12.1% in Euclidean space. This pattern aligns with the typical requirements of multivariate data learning: The model struggles to capture complex spatio-temporal coupling relationships if data are scarce, and even a small increase in data can lead to substantial performance gains. Once the ratio exceeds 0.5, the dominant patterns and anomaly types are sufficiently covered, which results in diminishing marginal returns. Due to its stronger geometric modeling capability, hyperbolic space can effectively learn the core structure even at low data ratios, and thus it is especially in the early improvement stage.

#### Hyperparameter analysis of sliding window size

**Fig. 4:**
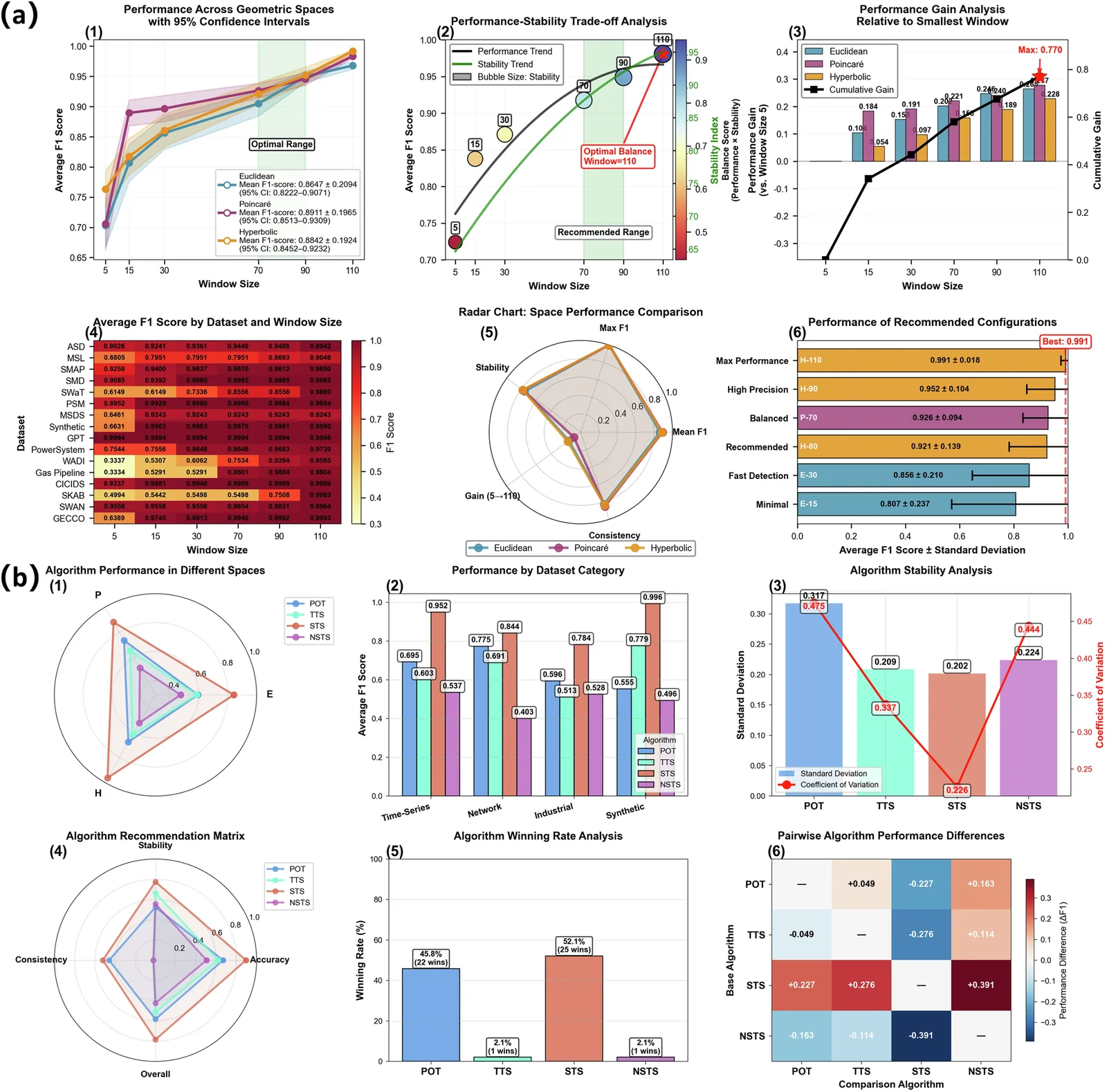
Sliding window sensitivity and performance stability analysis of anomaly evaluation algorithms for the HAO model. **a** Sensitivity analysis of the sliding window size hyperparameter for the HAO model. The stability metric in (**a(2)**) is defined as $1-{\mbox{CV}}({\mbox{X}})$, where ${\mbox{CV}}({\mbox{X}})=\sigma ({\mbox{X}})/\mu ({\mbox{X}})$ denotes the coefficient of variation. **b** Performance and stability analysis of anomaly evaluation algorithms for multivariate anomaly detection. POT refers to an anomaly detection method based on extreme value theory. TTS denotes the approach based on data smoothing. STS represents the anomaly detection method proposed in this work, which is based on natural structure sparsification. NSTS represents the natural structure non-sparsified anomaly detection method, which is also proposed in this work.

The F1-score of the HAO model series shows an overall upward trend with the increase of the sliding window size, as shown in Fig. 4a(1). When the data sliding sampling window is 5, the F1-score of the HAO model is 0.70 in the Euclidean space, 0.75 in the Poincaré ball space and 0.80 in the hyperbolic space. When the data sliding sampling window is 110, the F1-score of the HAO model increases to 0.85 in the Euclidean space, 0.90 in the Poincaré ball space and 0.95 in the hyperbolic space. Among them, the F1-score improvement of the HAO model in the hyperbolic space from window 5–110 reaches 18.75%. When the data sliding sampling window is ≥70, the growth of the HAO model series slows down and tends to stabilize. This trend can be attributed to the long-period dependencies and complex spatial-temporal coupling characteristics in multivariate time series. Small windows cannot fully capture abnormal patterns, while large windows can cover richer temporal information, which provides stronger support for geometric structure learning and dynamic graph structure discovery. Regarding the trade-off between performance and stability of the HAO model series, as shown in Fig. 4a(2), there exists an optimal window configuration for the HAO model that achieves the best balance between performance and stability. When the data sliding sampling window is 110 in the hyperbolic space, the balance score of the HAO model is 78.6, which is higher than other spaces and window combinations. The optimal data sliding sampling window of the HAO model is 90 in the Poincaré ball space and 70 in the Euclidean space, and their balance scores are both lower than that of the HAO model in the hyperbolic space. These results indicate that the multivariate anomaly detection task needs to consider both performance and stability. Small windows suffer from insufficient temporal information, which results in poor performance and stability, while large windows improve performance, but they may introduce instability due to redundant information.

The performance gain of the HAO model series relative to the minimum data sliding sampling window of 5 shows a trend of rapid increase followed by gradual slowdown as the window enlarges, as shown in Fig. 4a(3). The performance gain of the HAO model improves significantly when the data sliding sampling window ranges from 5 to 30, where the performance gain in the hyperbolic space reaches 0.202. When the data sliding sampling window is ≥90, the performance gain rate of the HAO model in the hyperbolic space slows down. At window 110, the cumulative performance gain in the hyperbolic space is 0.246, which is superior to 0.240 in the Poincaré ball space and 0.228 in the Euclidean space. This trend is consistent with the characteristics of multivariate time series: Lower sliding sampling windows have a limited temporal span, and this leads to obvious information gain. When the window is ≥90, it has basically covered the complete evolution of most anomalies, and the marginal benefit of information increment decreases. The adaptability of different datasets to window size varies significantly, which is especially reflected in the difference in task difficulty, as shown in Fig. 4a(4). For high-difficulty datasets, such as PSM, CICIDS and GECCO, the performance improvement of the HAO model series is particularly significant when the data sliding sampling window is ≥90. For low-difficulty datasets, such as SMD, SWaT, and Synthetic, satisfactory performance can be achieved with a window of 30–70. For example, the average F1-score of the HAO model is 0.75 at window 5 and 0.97 at window 110 on the Power System dataset, with an increase of 29.33%. On the SWaT dataset, the average F1-score is 0.73 at window 30 and 0.99 at window 110, with an increase of 35.62%. These experimental results show that high-difficulty datasets usually have high dimensionality, diverse anomaly types and strong variable coupling, requiring larger windows to capture anomaly evolution paths and cross-variable correlations. In contrast, low-difficulty datasets have simple anomaly structures and clear dependencies, which can be well modeled with medium windows.

The multi-dimensional performance comparison of the HAO model series, as shown in Fig. 4a(5), demonstrates that the HAO model in the hyperbolic space is comprehensively superior across multiple metrics, while the performance in the Euclidean space is the weakest. This indicates that the hyperbolic space improves not only the core detection performance in terms of F1-score, but also the gain efficiency, stability and consistency across datasets. The above results verify the adaptability and superiority of the hyperbolic space under the comprehensive performance requirements of multivariate anomaly detection. Finally, according to the deployment requirements of different application scenarios, Fig. 4a(6) provides the recommended configuration strategy. For high-precision scenarios, the recommended combinations of data representation space and sliding window are H-110 and H-90. For scenarios balancing performance and efficiency, P-70 and H-80 are recommended to achieve a good trade-off. For real-time fast detection scenarios, E-30 and E-15 are suggested. Although the F1-score of the HAO model is relatively low under these configurations, the small window enables fast inference and makes it suitable for real-time applications. It is worth noting that the F1-scores of all recommended configurations exceed 0.8, which ensures effectiveness in practical applications. This strategy reflects the strong scenario adaptability of the HAO model, and it can achieve the optimal balance of performance and efficiency by flexibly combining the representation space and sliding window in industrial high-precision monitoring, real-time network intrusion response, or resource-constrained edge computing scenarios.

#### Hyperparameter analysis of anomaly evaluation algorithms

The average F1-scores of the HAO-series models across the four anomaly detection algorithms are significantly higher than those of the HAO model in the Euclidean space, as shown in Fig. 4b(1). Among them, the STS anomaly detection algorithm achieves the best performance, and it is well-adapted to the geometric properties of multivariate data. Specifically, the average F1-scores of the HAO model in the hyperbolic space using the STS, POT, TTS, and NSTS anomaly detection algorithms are 0.996, 0.844, 0.779, and 0.775, respectively. In contrast, the F1-score of the STS algorithm in the Euclidean space is only 0.603, and the average F1-scores of the other algorithms (POT, TTS, and NSTS) are all below 0.6. Multivariate time series generally exhibit complex nonlinear spatial-temporal coupling relationships, and the geometric representation of hyperbolic space better aligns with the natural characteristics of such data, which provides a precise feature foundation for anomaly detection algorithms. The STS anomaly detection algorithm, which is designed to match the geometric optimization logic of hyperbolic space, can effectively capture both numerical and geometric differences of anomalies, thus yielding superior performance. In comparison, Euclidean space suffers from geometric representation distortion, which limits the discriminative capability of various anomaly detection algorithms and results in relatively low overall performance.

The adaptability of different anomaly detection algorithms to application scenarios varies significantly, as shown in Fig. 4b(2). The STS algorithm performs favorably across all anomaly detection scenarios, while the POT algorithm only shows certain advantages under specific conditions. In detail, the average F1-score of STS in industrial anomaly detection reaches 0.784, which outperforms other algorithms. In time series and network intrusion detection scenarios, POT achieves average F1-scores of 0.695 and 0.775, respectively, which are higher than those of TTS and NSTS. In synthetic anomaly detection scenarios, TTS attains an average F1-score of 0.779, which is higher than POT. Such discrepancies are closely related to the anomaly patterns in each scenario and the underlying principles of the algorithms. The four anomaly detection scenarios all involve complex patterns, such as multi-sensor collaborative anomalies and attack anomalies. By combining hyperbolic geometric features and temporal localization logic, STS can accurately identify these complex anomalies. In Internet, time series and industrial scenarios dominated by intrusion attacks, the thresholding strategy of POT better matches their anomaly distribution. In synthetic anomaly scenarios with relatively simple patterns, the basic evaluation mechanism of TTS is sufficient for detection. Notably, although NSTS adopts a natural structural design, it lacks a dedicated sparsification strategy, which makes it difficult to suppress background noise interference. In addition, because NSTS focuses on critical anomaly signals, which limits its localization performance across multiple scenarios.

Regarding the stability of the HAO-series models, as shown in Fig. 4b(3), STS demonstrates the best stability, and it is more robust to fluctuations across multiple datasets, with a standard deviation of only 0.202 and the lowest coefficient of variation. In contrast, POT has a standard deviation of 0.317 and the highest coefficient of variation. This indicates that the dual-modal scoring mechanism of STS, which integrates both geometric and numerical differences, reduces fluctuations caused by single-dimensional evaluation and improves stability. Conversely, POT relies on a single-threshold strategy, which makes it sensitive to changes in data distribution and results in inferior stability. For anomaly detection algorithm recommendation, as shown in Fig. 4b(4), STS is optimal across all dimensions, while POT is only a candidate for specific scenarios. This confirms that STS, through the combination of hyperbolic space geometric optimization, dual-modal scoring and temporal localization, achieves superior accuracy, consistency and stability, which makes it widely applicable to complex scenarios, such as industrial control and cybersecurity. Although POT achieves reasonable accuracy on some simple datasets, its insufficient stability and cross-scenario consistency restrict it to a supplementary role. NSTS and TTS rely on single-dimensional evaluation and exhibit limited overall performance, which is inadequate for practical applications.

The winning rates of the anomaly detection algorithms on different multivariate anomaly detection scenarios are shown in Fig. 4b(5). STS achieves the highest overall winning rate at 52.1%, followed by POT at 45.8%, while TTS and NSTS both stand at only 2.1%. This trend is strongly correlated with the complexity of multivariate datasets: STS, which uses hyperbolic geometric representation and precise evaluation logic, performs best on moderately and high-difficult datasets. POT achieves higher winning rates on modest difficulty datasets. TTS and NSTS employ relatively simple evaluation logic and struggle to adapt to complex multivariate scenarios, only occasionally achieving optimal results on simple datasets, which results in the lowest winning rates. Finally, pairwise performance comparisons using the F1-score differences of the HAO model as shown in Fig. 4b(6) show that STS outperforms POT, TTS and NSTS by F1-score margins of +0.391, +0.276 and +0.227, respectively. These are all significant positive improvements. The difference between POT and TTS is only +0.049. This validates the significant performance advantage of STS: Its integration of hyperbolic geometric features and multi-dimensional evaluation effectively compensates for the deficiencies of other algorithms and creates a clear performance gap. In comparison, POT and TTS are based on traditional thresholding or temporal evaluation mechanisms, and they fail to fully exploit the geometric properties of multivariate data, which results in minor performance differences.

#### Training stability analysis

The curvature variation analysis of the HAO model in hyperbolic space is shown in Supplementary Fig. 10. As training proceeds, the curvature of the spatio-temporal structure output module of the HAO model in hyperbolic space is dynamically adjusted and eventually converges to approximately 1.0. In contrast, although the curvature of the spatial linear module, temporal linear module and spatio-temporal structure discovery input module fluctuates, it remains globally stable without divergence or severe oscillation. For instance, the output curvature of the spatio-temporal natural structure discovery module of the HAO model stabilizes within the range of 0.999–1.000 on the ASD dataset (Supplementary Fig. 10a), SMAP dataset (Supplementary Fig. 10c) and WADI dataset (Supplementary Fig. 10k). The curvature fluctuation ranges of the spatial linear and temporal linear modules are controlled between 0.96 and 1.038, with no extreme deviation. This demonstrates that despite the complex spatio-temporal coupling in multivariate datasets, the HAO model can adaptively optimize its geometric components during training via a dynamic curvature adjustment mechanism. It not only fits the natural geometric properties of the data but also avoids curvature divergence through dynamic constraints, thereby achieving stable convergence during training. In addition, during the early training stage (0–30%), the curvature of geometric components varies considerably across datasets. For example, the initial curvature of the spatial linear module on the SKAB^51^ dataset (Supplementary Fig. 10n) reaches 1.204, while that of the temporal linear module on the Gas Pipeline dataset (Supplementary Fig. 10l) is only 0.978. The initial curvature of the spatial structure input module on the SMAP dataset (Supplementary Fig. 10c) is 0.972, and that of the temporal structure input module on the PSM dataset (Supplementary Fig. 10f) is 1.019. Such differences reflect the diversity of geometric characteristics among different datasets. Nevertheless, when training enters the middle and late stages ($\ge 50\%$), the curvature convergence paths of all modules gradually become smooth. This phenomenon is closely related to the heterogeneity of multivariate datasets: differences in dimensionality, anomaly types and spatio-temporal dependencies lead to distinct initial geometric features. Benefiting from the synergy between dynamic graph structure learning and geometric parameter optimization, the HAO model automatically adapts to the heterogeneous geometric features of different datasets and eventually achieves consistent convergence. Specifically, the multi-module hierarchical curvature learning of the full model is fully implemented throughout the training process. The geometric modules at the input and middle layers maintain sufficient adaptive curvature adjustment margins to match diverse natural geometric distributions of data^58^. The final output module uniformly converges to a curvature of 1.0, which serves as the standardized geometric calibration criterion for downstream anomaly scoring and feature fusion.

From the perspective of anomaly detection difficulty, the curvature fluctuation amplitude of geometric components on datasets with high detection difficulty is significantly larger than that on low-difficulty datasets. For example, on the high-difficulty CICIDS dataset (Supplementary Fig. 10m), the curvature of the spatial linear module ranges from 0.979 to 1.010, and that of the temporal linear module ranges from 0.979 to 1.020. The fluctuation range is notably larger than that on the low-difficulty Synthetic dataset (Supplementary Fig. 10h), where the curvature of both spatio-temporal linear modules varies between 0.988 and 1.012. Similarly, the curvature fluctuation of the structure input module on the high-difficulty GECCO dataset (Supplementary Fig. 10f) is higher than that on the low-difficulty SWaT dataset (Supplementary Fig. 10i). This indicates that the geometric structure of high-difficulty datasets is generally more complex, and the deviation between the initial curvature and the true geometric properties of the data is larger, which results in a greater curvature adjustment required during training. By incorporating physical constraints into its geometric learning module, the HAO model effectively suppresses fluctuation amplitude and ensures stable convergence even in complex scenarios, which demonstrates strong adaptability to complicated environments. Crucially, the final curvature of the spatio-temporal structure output module converges to 1.0 for all datasets, which verifies that the geometric optimization objective of the HAO model is achieved. Through continuous learning of geometric parameters, the curvature of the output module is stabilized at the optimal point of 1.0. This not only guarantees the accuracy of geometric representation but also provides a unified geometric benchmark for the subsequent bimodal fusion scoring mechanism. The fulfillment of this objective enables the model to accurately capture the spatio-temporal coupling in multivariate data and avoid representation distortion caused by geometric degradation, thus laying a solid geometric foundation for anomaly detection and diagnosis. This also explains the fundamental reason why the HAO model consistently maintains high ROC performance across multiple datasets.

### Multivariate anomaly diagnosis

#### Comparative performance analysis of anomaly diagnosis

The label correction strategy of the HAO-series models effectively alleviates the problem of fine-grained label deficiency in multivariate anomaly detection datasets, thereby providing a unified and reliable benchmark for evaluating the anomaly diagnosis performance of baseline models and the proposed model in this study.

**Fig. 5:**
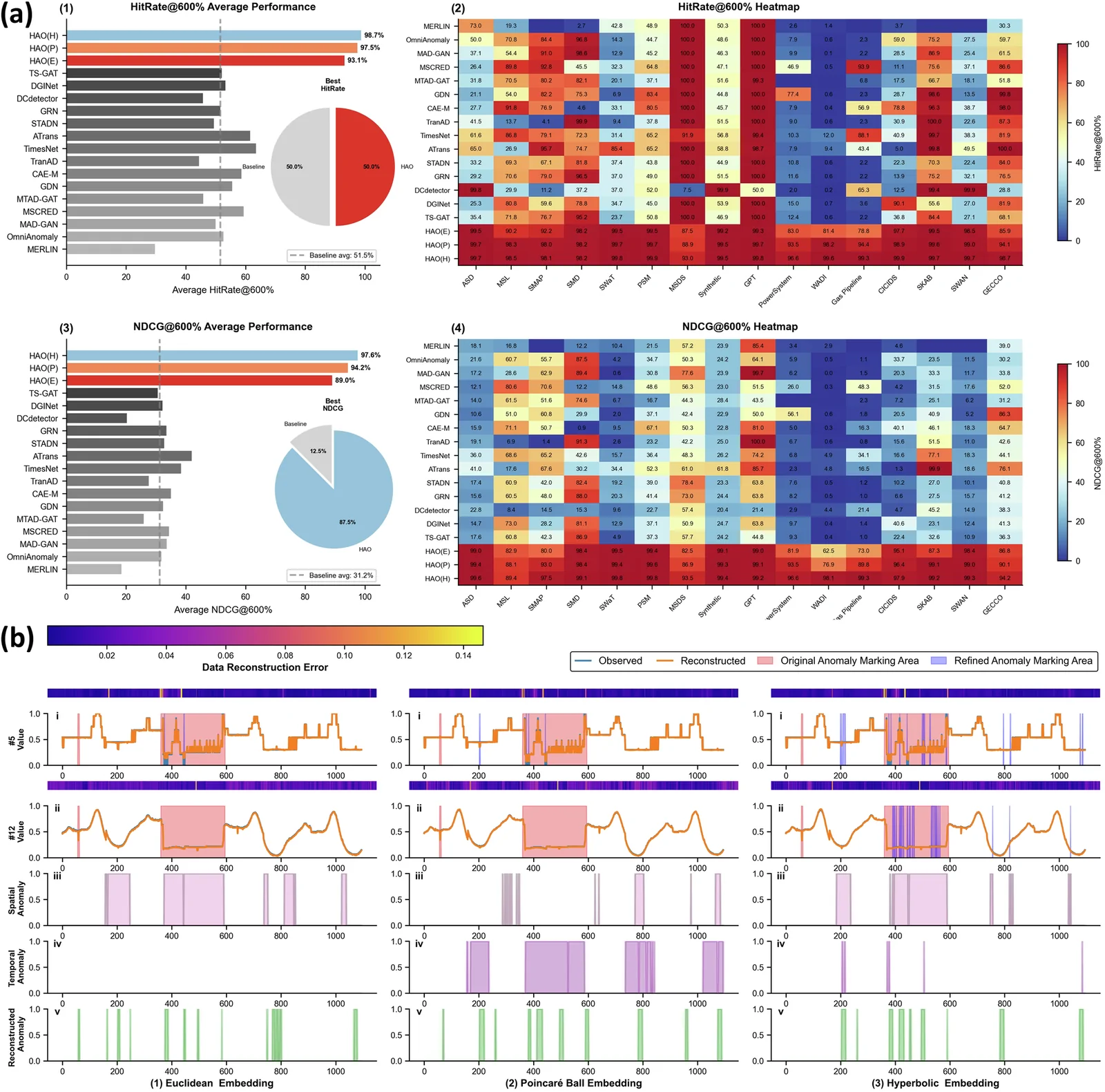
Multivariate anomaly diagnosis experiment. **a** Comparative analysis of anomaly diagnosis performance for multivariate anomaly detection models. **b** Analysis of HAO-series models correction performance on multivariate anomaly labelings.

The performance of multivariate anomaly diagnosis HitRate@600% under different detection scenarios is shown in Fig. 5a(1). Specifically, the average HitRate@600% of the HAO model in Euclidean space is 93.1%, that in the Poincaré ball space is 97.5% and that in hyperbolic space is 98.7%. In contrast, the average HitRate@600% of all baseline models is below 70%. ATrans and TimesNet achieve stable average HitRate@600% values ranging from 61.6 to 63.5% across all experimental datasets, yet remain within the low-performance range of traditional comparison models. Furthermore, in terms of anomaly diagnosis quality, as shown in Fig. 5a(3), the average NDCG@600%^25^ of the HAO model in Euclidean space is 89.0%, that in the Poincaré ball space is 94.2% and that in hyperbolic space is 97.6%, while the average NDCG@600% of baseline models is below 50%. These results demonstrate that the key challenge in multivariate anomaly diagnosis lies in the precise attribution of multivariate spatio-temporal coupling relationships. However, existing baseline models mostly rely on static structures or the fusion learning of spatio-temporal features and lack fine-grained spatio-temporal localization for anomalies. ATrans captures long-range temporal dependencies but neglects multivariate spatial topology. DCdetector screens local anomalies without global spatio-temporal logic. TimesNet extracts multi-scale temporal features but fails to decouple multivariate coupled anomalies. They can only identify anomaly locations based on sensor information during anomaly reconstruction, which results in limited generalization ability. Conversely, the HAO-series models, through geometric optimization of the data representation space and the independent collaborative mechanism for multivariate spatio-temporal graph structure discovery, can simultaneously integrate spatial anomalies, temporal anomalies and model reconstruction anomalies, which achieves more accurate multivariate spatio-temporal anomaly localization and thus significantly improving diagnosis performance.

The performance of multivariate anomaly diagnosis across various application scenarios is presented in Fig. 5a(2). The HitRate@600% of the HAO model under most detection scenarios falls within the high-accuracy region, whereas baseline models exhibit significant fluctuations across datasets, and they are mostly concentrated in the low-value range. For instance, on the distributed system dataset MSDS and the gas pipeline system dataset GPT, baseline models achieve relatively high HitRate@600%, because anomalies in these scenarios are mostly intrusion detection types with obvious numerical mutation characteristics, which benefit anomaly diagnosis based on numerical reconstruction methods. Nevertheless, due to the mutual constraints of multivariate spatio-temporal coupling relationships, the performance of diagnosis methods based on numerical reconstruction drops significantly in complex scenarios, such as the Internet service dataset ASD and the water treatment plant dataset SWaT. The lack of a systematic anomaly localization mechanism leads to large performance volatility. The quality of multivariate anomaly diagnosis is shown in Fig. 5a(4). Multivariate anomalies often originate from complex situations, such as collaborative anomalies of multiple sensors and mixtures of primary and secondary anomalies. The HAO-series models maintain high ranking quality on all datasets, while avoiding low-quality hits in HitRate@600% scenarios, thus improving the interpretability of anomaly causes. This indicates that, with the spatio-temporal anomaly decoupling representation and bimodal fusion scoring mechanism, the HAO model can accurately identify the primary-secondary relationships of anomalous sensors, and its ranking quality is significantly superior to that of baseline models. In contrast, baseline models struggle to effectively distinguish anomaly priorities due to the lack of fine-grained anomaly attribution capability, which results in poor ranking performance.

#### Impact analysis of label correction

The reconstruction anomaly regions of the HAO model in Euclidean space show lower coincidence with the original annotations, and the data reconstruction errors are widely scattered, which indicates a limited ability to identify and correct annotation errors, such as missed and false labels. The results of spatial anomalies (Fig. 5b(1(iii))) and reconstruction anomalies (Fig. 5b(1(v))) reveal that the corrected anomaly regions differ slightly from the original annotations. However, due to the inherent geometric distortion in Euclidean representation, it remains difficult to fully model the complex spatio-temporal coupling relationships in multivariate server data, even if the original annotated anomalies can be identified. The matching degree between reconstruction anomalies and original annotations is improved for the HAO model in the Poincaré ball space. Within the timestamp range of 200–400, temporal structural anomalies are locally concentrated in the originally annotated regions (Fig. 5b(2(iv))). Sparse additional annotation points appear in the spatial anomaly map (Fig. 5b(2(iii))), which correspond to the missed regions in the original annotations, although false-labeled regions are not completely eliminated. The non-Euclidean geometric property of the Poincaré ball space adapts to the nonlinear characteristics of the data to a certain extent, and it can discover some unannotated natural anomalies. Nevertheless, its ability to analyze anomaly relations is improved compared with Euclidean space, which is restricted by its limited geometric representation power, but the label correction performance still needs further enhancement.

The HAO model in hyperbolic space achieves a significantly higher matching degree between reconstruction anomaly regions and real anomaly patterns. Data reconstruction errors are concentrated in the original annotated regions and missed-label regions, which enables accurate recognition of missed labels and effective removal of false labels. Within the timestamp range of 200–600, reconstruction errors form obvious high-value concentrations in the original annotated regions, and new high-value missed-label regions appear between timestamps 800–1000 (Fig. 5b(3(v))). Spatial anomalies (Fig. 5b(3(iii))) and temporal anomalies (Fig. 5b(3(iv))) demonstrate that the corrected anomaly regions cover real anomalies and remove false-labeled points unsupported by reconstruction errors, which achieves strong alignment between reconstruction anomalies and ground-truth anomalies. The corrected anomaly regions can fully cover the real anomaly scope, eliminate false-labeled points without reconstruction error support, and realize precise alignment between reconstruction anomalies and real anomalies. The geometric properties of hyperbolic space effectively capture the spatio-temporal coupling anomaly patterns in server data, and its reconstruction results faithfully reflect the anomaly distribution. This space is significantly superior to the other two spaces in identifying and correcting missed and false labels. Overall, the HAO-series model can not only identify original anomalies but also locate potential anomalies through anomaly interpretability. In the process of correcting coarse-grained anomaly labels toward fine-grained annotations, the geometric representation capability of the HAO model accurately mines data anomaly patterns. The joint spatio-temporal anomaly localization provides an objective basis for label refinement, which lays a reliable label foundation for the subsequent training and performance evaluation of multivariate anomaly detection models.

### Multivariate anomaly interpretation

#### Analysis of multivariate spatio-temporal relationship parsing capability

**Fig. 6:**
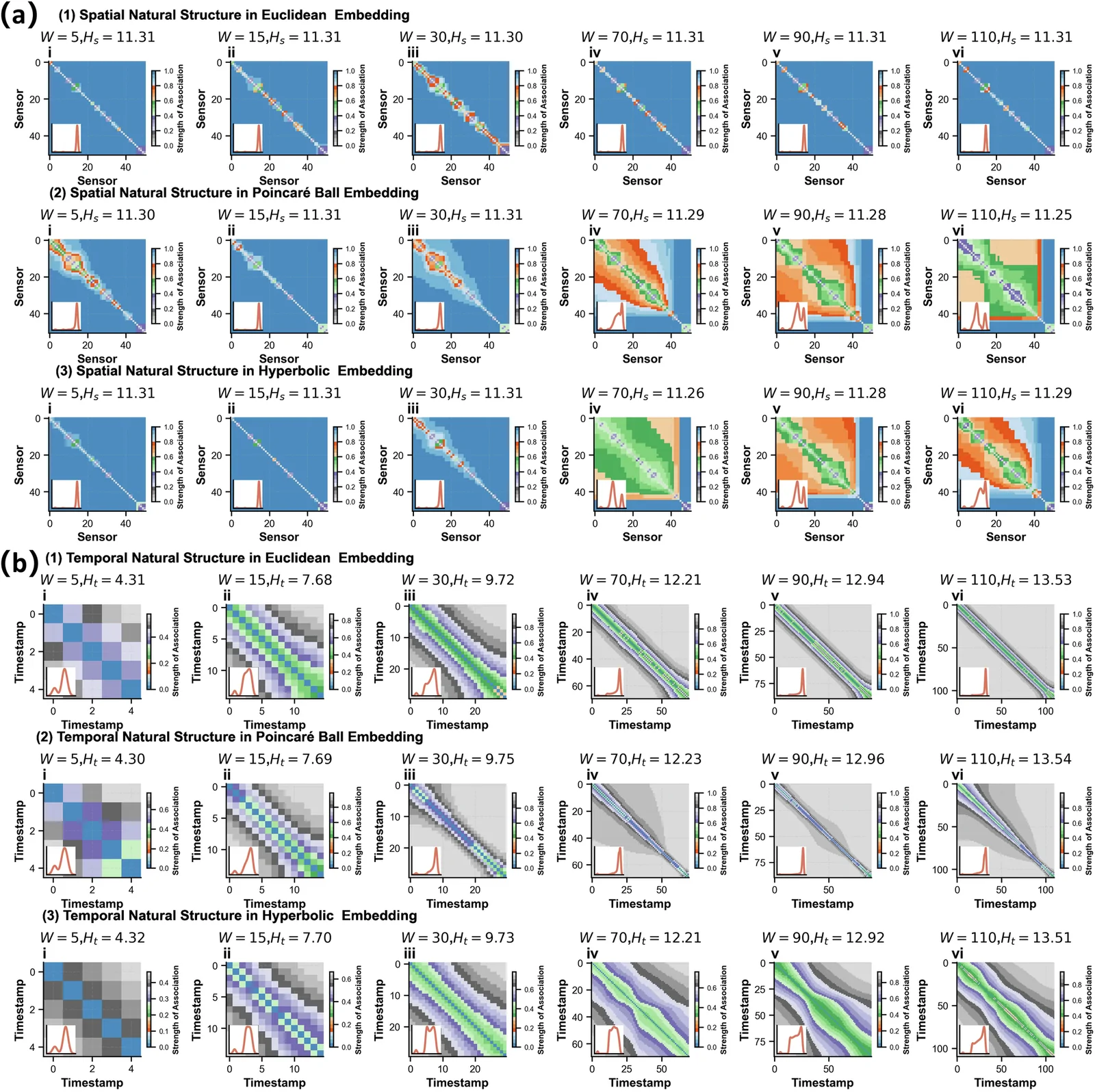
Comparative experiment on multivariate spatio-temporal relation resolution. **a** Analysis of the impact of embedding space and sliding window on natural multivariate coupling structures. The analysis is conducted on the SWaT dataset, which exhibits dynamic coupling relationships. Edge entropy, denoted as ${H}_{{{\boldsymbol{s}}}}({{\bf{A}}})=-{\sum }_{i=1}^{N}{p}_{i}\cdot \log ({p}_{i})$, where ${p}_{i}={{{\bf{A}}}}_{i}/{\sum }_{j=1}^{N}{{{\bf{A}}}}_{j}$ is used to reflect the richness of coupling relationships, while the association strength distribution reflects the tightness of dependencies between different sensors. **b** Analysis of the impact of embedding space and sliding window on natural multivariate temporal structures. The analysis is conducted on the SMAP dataset, which exhibits long-term evolutionary characteristics. In this analysis, the edge entropy, denoted as ${H}_{{{\boldsymbol{t}}}}({{\bf{A}}})=-{\sum }_{i=1}^{N}{p}_{i}\cdot \log ({p}_{i})$, where ${p}_{i}={{{\bf{A}}}}_{i}/{\sum }_{j=1}^{N}{{{\bf{A}}}}_{j}$ is used to reflect the richness of temporal associations, while the association strength distribution is employed to reflect the tightness of dependencies between different timestamps.

Under the Euclidean space, the HAO model yields a uniform correlation strength distribution of multivariate coupling structures with fixed edge entropy ${H}_{{{\boldsymbol{s}}}}$ as shown in Fig. 6a. Varying the sliding sampling window does not bring obvious gain in mining coupling relationships, which indicates significant limitations in geometric representation. The correlation strengths corresponding to different sliding window sizes are concentrated in the range of 0.2–0.8 with a uniform distribution, and the ${H}_{{{\boldsymbol{s}}}}$ remains stable between 11.30 and 11.31. For instance, the ${H}_{{{\boldsymbol{s}}}}$ is 11.31 for both a sliding sampling window of 5 (Fig. 6a(1(i))) and 110 (Fig. 6a(1(vi))), with consistent trends in correlation strength distribution. This demonstrates that the linear geometric nature of Euclidean space is inadequate to characterize the nonlinear dependencies among multivariate variables. The incremental temporal information introduced by the sliding sampling window fails to improve the ability of coupling structure mining, which only homogenizes the correlation strength without distinguishing the relationships between core and secondary coupling. In the Poincaré ball space, the ${H}_{{{\boldsymbol{s}}}}$ of the multivariate coupling structure obtained by the HAO model decreases slightly as the sliding sampling window enlarges and the correlation strength exhibits local aggregation, which leads to better discrimination of coupling relationships compared with Euclidean space. The ${H}_{{{\boldsymbol{s}}}}$ drops from 11.31 to 11.25, and the correlation strength distribution gradually shifts from uniform to locally high-density regions, where core coupling relationships begin to emerge. For example, the ${H}_{{{\boldsymbol{s}}}}$ is 11.30 at a sliding sampling window of 5 (Fig. 6a(2(i))), and it decreases to 11.25 at a window size 110 (Fig. 6a(2(vi))). At window sizes of 70 (Fig. 6a(2(iv))) and 90 (Fig. 6a(2(v))), the correlation strength forms local high-density clusters in the range of 0.4–0.6, corresponding to the core dependencies among sensors. The non-Euclidean geometry of the Poincaré ball space preliminarily adapts to the nonlinear coupling characteristics of multivariate data. The sufficient temporal information brought by a larger sliding sampling window enables an initial distinction between core and secondary couplings. However, the reduction in ${H}_{{{\boldsymbol{s}}}}$ is limited, and it implies that the geometric representation capability still has room for improvement.

In the hyperbolic space, the ${H}_{{{\boldsymbol{s}}}}$ of the multivariate coupling structure obtained by the HAO model decreases notably with an increasing window size, and the correlation strength shows prominent aggregation, where core coupling relationships are precisely highlighted and match the nature well of dynamic multivariate coupling. The ${H}_{{{\boldsymbol{s}}}}$ falls from 11.31 to 11.26, and the correlation strength distribution displays obvious local clustering with a clear boundary between core and secondary coupling relationships. For example, the ${H}_{{{\boldsymbol{s}}}}$ is 11.31 at a sliding sampling window of 5 (Fig. 6a(3(i))); it drops to 11.26 at window size 70 (Fig. 6a(3(iv))) and remains at 11.28 at window size 90 (Fig. 6a(3(v))). Within the sliding window range of 30–100, the correlation strength forms a high-density cluster in the interval of 0.4–0.6, corresponding to the core control couplings between sensors and actuators, while secondary couplings are distributed in 0.2–0.4. The geometric properties of hyperbolic space can accurately capture the nonlinear dynamic coupling of multivariate signals. The incremental temporal information from a larger sliding sampling window is fully converted into gains in coupling structure mining, and the reduction in ${H}_{{{\boldsymbol{s}}}}$ refines the coupling relationships, which provides a precise structural foundation for anomaly root-cause analysis. A comparison of the coupling structures of the HAO-series models reveals that hyperbolic space performs the best, followed by the Poincaré ball space, and Euclidean space is the weakest in discrimination of correlation strength, highlighting of core coupling relationships and dynamic optimization of ${H}_{{{\boldsymbol{s}}}}$. Furthermore, a significant synergistic effect exists between the sliding sampling window and the embedding space, but it is only in hyperbolic space that increasing the window size can effectively improve the quality of coupled structure mining. For instance, at a sliding window of 70, the ${H}_{{{\boldsymbol{s}}}}$ in hyperbolic space is 11.26 (Fig. 6a(3(iv))), which is lower than 11.31 in Euclidean space and 11.29 in the Poincaré ball space at the same window size, with more pronounced correlation strength clustering. Euclidean space shows no synergistic effect, and the Poincaré ball space exhibits only a weak synergistic effect. These results verify the rationality of the spatial selection design in the HAO model. The geometric representation capability of hyperbolic space and the temporal information capture ability of the sliding window form an effective synergy and enable accurate mining of the natural coupling structure of multivariate data. In contrast, Euclidean space suffers from geometric distortion, and the Poincaré ball space lacks sufficient representation power, which cannot fully use the gain effect of the sliding window.

The ${H}_{{{\boldsymbol{t}}}}$ of the multivariate temporal structure learned by the HAO model in Euclidean space exhibits an upward trend as the sliding window increases. As shown in Fig. 6b, it rises from 4.31 to 13.53 with an increase of up to 214%. For example, when the sliding sampling window is 5 (Fig. 6b(1(i))) and 110 (Fig. 6b(1(vi))), the temporal correlation strength is uniformly distributed in the range of 0.0–0.8 without obvious high-density aggregation, which makes it difficult to accurately identify core temporal dependencies. This indicates that the geometric property of Euclidean space is inadequate to effectively characterize the nonlinear dependencies of time series. Although a larger sliding window introduces more temporal information, it only forms a correlation distribution without clear discrimination, resulting in a lack of pertinence in mining temporal dependencies. The ${H}_{{{\boldsymbol{t}}}}$ of the HAO model in the Poincaré ball space also rises steadily from 4.30 to 13.54. The overall growth trend is similar to that in Euclidean space, but the distribution of correlation strength begins to show a discernible regularity. When the sliding sampling window is 70 (Fig. 6b(2(iv))) and 90 (Fig. 6b(2(v))), the temporal correlation strength forms local high-density clusters in the range of 0.4–0.6, corresponding to the core dependencies at key timestamps. In the Poincaré ball space, the non-Euclidean geometric property can preliminarily adapt to the nonlinear evolution of time series and the incremental information brought by a larger sliding sampling window can be partially filtered and mapped to core dependencies. However, the aggregation range is still relatively limited, and the geometric representation capability remains to be improved.

For the HAO model in hyperbolic space, the ${H}_{{{\boldsymbol{t}}}}$ also increases from 4.32 to 13.51, and the correlation strength shows significant aggregation. When the sliding sampling window is within the range of 30–110, high-density clusters appear in the correlation strength interval of 0.4–0.6, corresponding to core temporal dependencies, while secondary dependencies are distributed in 0.0–0.4 with a clear boundary, which well matches the natural characteristics of temporal evolution. The geometric property of hyperbolic space can accurately characterize nonlinear evolution and long-period dependencies. The incremental information from a larger sliding window is efficiently converted into performance gains for coupling structure mining, which not only enriches the hierarchy of temporal relations through the orderly variation of ${H}_{{{\boldsymbol{t}}}}$, but also achieves accurate localization of core dependencies, thus providing a solid structural foundation for anomaly temporal root cause analysis. A comprehensive comparison of the HAO-series models in different embedding spaces shows that hyperbolic space performs best in terms of correlation strength aggregation, core dependency locking capability and rationality of ${H}_{{{\boldsymbol{t}}}}$ growth, followed by the Poincaré ball space, with Euclidean space being the weakest. A significant synergistic effect exists between the sliding sampling window and the embedding space. It is only in hyperbolic space that a larger sliding sampling window can effectively improve the quality of coupling structure mining. For example, when the sliding sampling window is 70 (Fig. 6b(3(iv))), the ${H}_{{{\boldsymbol{t}}}}$ is 12.21, which is close to those in the Poincaré ball space and Euclidean space at the same window size. However, hyperbolic space achieves a more significant correlation strength aggregation and higher discrimination of core dependencies. In contrast, Euclidean space shows no synergistic effect: although ${H}_{{{\boldsymbol{t}}}}$ increases with the window size, the temporal dependency distribution remains irregular. The Poincaré ball space presents only a weak synergistic effect. These results verify the rationality of choosing hyperbolic space for the HAO model. Its geometric representation and window information capture capability cooperate precisely, which enables deep mining of the natural temporal structure of multivariate data. In contrast, the geometric distortion in Euclidean space and the insufficient representation of the Poincaré ball space both fail to fully use the information gain brought by the sliding window.

#### Comparison of multivariate spatio-temporal natural structures

**Fig. 7:**
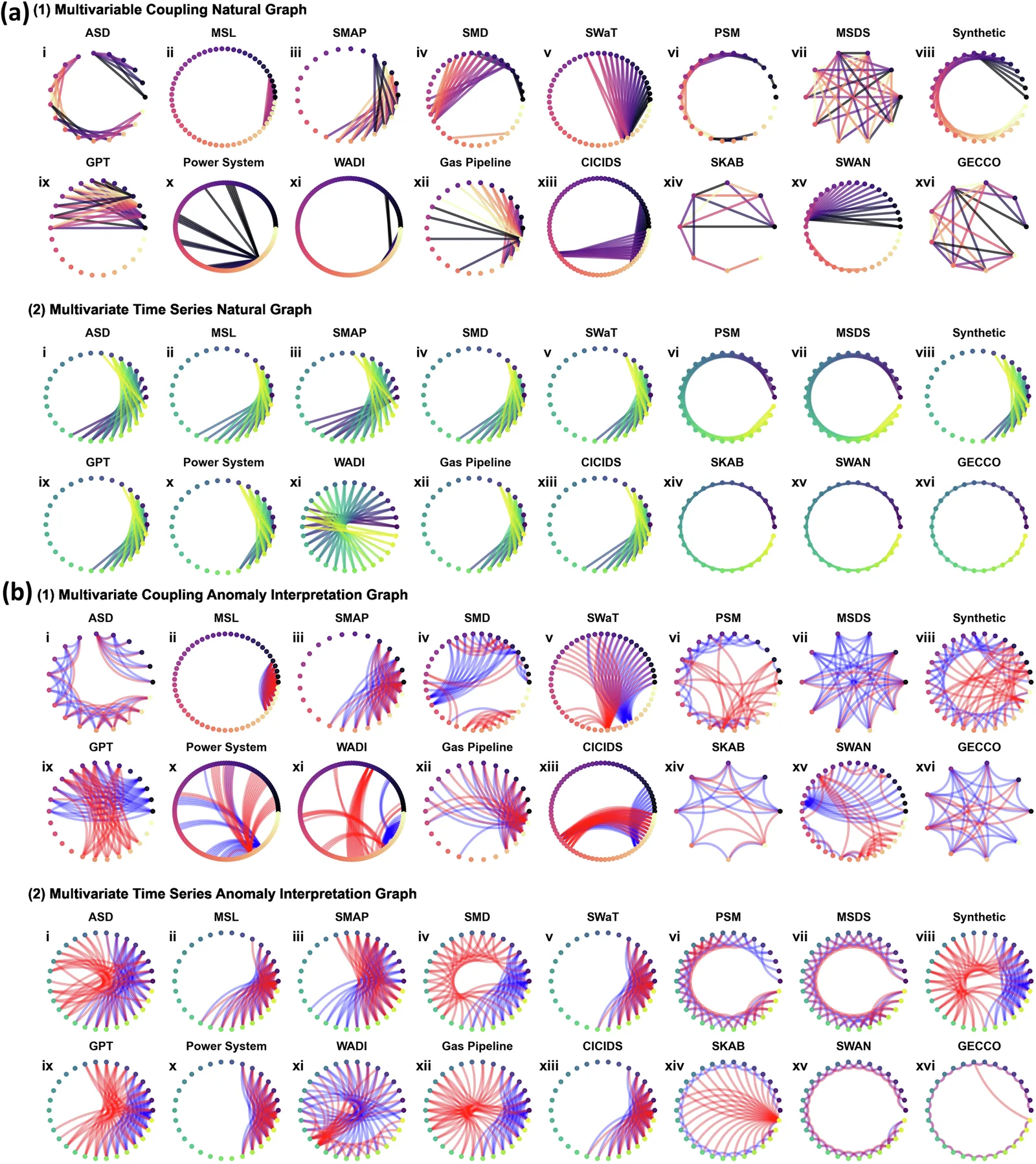
Analysis of multivariate spatio-temporal natural structure and interpretation. **a** Analysis of natural multivariate spatio-temporal structures. **b** Multivariate anomaly interpretation. It shows an analysis of the effectiveness of multivariate temporal structure anomaly interpretation. When constructing the interpretation graph for multivariate temporal dependencies, the proposed method follows the procedure below. Using normal mode data with a sliding window size of 30, the natural temporal dependency structure is calculated and visualized, with blue lines representing these dependencies. Simultaneously, the predicted temporal structure is computed using the same sliding window size. Red indicates dependency strength, and the top 30 strongest dependency relationships are visualized separately for comparative analysis. The analysis of the anomaly interpretation graph focuses on identifying co-occurrence relationships. If a predicted temporal dependency edge shown in red coexists with its corresponding normal mode edge in blue, this indicates the presence of accompanying phenomena and the dependency is classified as part of the normal pattern. Conversely, if the predicted edge lacks a corresponding blue edge, which means that the accompanying phenomenon is absent, it is identified as an anomalous association.

The natural data structure serves as a critical carrier for reflecting the operational logic of information systems and explaining the root causes of anomalies. By uncovering the spatio-temporal natural structure inherent in multivariate data and analyzing differences among these structures, the HAO model provides essential clues for achieving a system-level understanding of natural structure characteristics across different domains, as shown in Fig. 7a.

The multivariate coupling natural structure is shown in Fig. 7a(1). The server operating dataset (Fig. 7a(1(i, iv))) exhibits an obvious community structure in its natural pattern, where internal system components tend to form tightly correlated subgroups. In contrast, the aerospace dataset (Fig. 7a(1(ii, iii))) displays typical characteristics of high cohesion and low coupling, which directly reflects the system architecture principle of modular design in aerospace engineering. From the perspective of system scale, the results show clear differentiation. Small-scale datasets (Fig. 7a(1(xiv–xvi))) generally have higher graph density, indicating denser connections among components and higher overall integration in small systems. Large-scale datasets (Fig. 7a(1(x, xi, xiii))) present significantly lower natural graph density (Fig. 7a(1(iv–vi))), which reflects relatively sparse connections between components in large-scale systems. Notably, design principles still vary among large-scale systems. Both the power network and aerospace datasets adhere to the principle of high cohesion and low coupling, exhibiting distinct community structure in inter-subsystem connections: strong internal clustering and sparse inter-cluster links. However, the subsystems of the water system dataset WADI rely mainly on sparse inter-community links (Fig. 7a(1(xi))), which may make its security more vulnerable to potential attacks. Conversely, the dense node connections in the network intrusion detection dataset CICIDS (Fig. 7a(1(xiii))) can enhance system robustness and thus mitigate the risk of network intrusions. Medium-scale datasets (Fig. 7a(1(iii–vi, xv))) exhibit diverse connectivity features ranging from dense to sparse (Fig. 7a(1(vii–x))), which reflects a design strategy that improves system security by maintaining relatively independent connections among subsystems.

The multivariate time series natural structure is shown in Fig. 7a(2), where distinct structural characteristics are observed across datasets. As shown in Fig. 7a(2(i–v, viii–xiii)), multiple types of anomalies are intertwined with prominent long-period dependencies, which yields highly complex temporal patterns. Figure 7a(2(vi, vii)) generally exhibits dynamically coupled temporal structures and their evolutionary chains. In contrast, the structures in Fig. 7a(2(xiv–xvi)) are simple and stable, with no complex interweaving behaviors. This demonstrates that the HAO model can effectively address multi-level challenges in multivariate anomaly detection, which range from complex, intertwined, long-period patterns to simple, stable core anomalies and can achieve high-precision recognition and discrimination. Among the most frequently observed time series natural structures (Fig. 7a(2(i–v, viii–xiii))), different datasets show remarkable similarity. This phenomenon can be analogized to an alarm clock knob, where different information systems function as alarm clocks of various brands. Their temporal dependency patterns may differ in strength and time scale, similar to how turning the knob clockwise or counterclockwise strengthens or weakens dependencies and shifts the balance between short-term and long-term dominance. Nevertheless, the underlying physical mechanisms governing these temporal dependency patterns remain consistent. This observation suggests that temporal dependency properties may share a unified physical foundation for domain knowledge transfer across information systems. The characteristic of exponential decay of time-dependent information commonly observed in various information systems (Fig. 7a(2(vi, vii))) is a concrete manifestation of this unified principle. Notably, despite belonging to entirely different application domains, medium-scale systems still display highly similar natural structures (Fig. 7a(2(vii–x))). Such cross-domain structural commonality provides strong empirical support for knowledge transfer among heterogeneous information systems. In addition, all medium-scale systems maintain dense connections via core network nodes to ensure essential information interaction between subsystems. However, it must be noted that these high-impact nodes serving as critical hubs may also become primary targets of security attacks.

#### Multivariate anomaly cause interpretation graph

The interpretation of multivariate anomaly causes relies on a reliable anomaly pattern benchmark. The HAO model constructs this benchmark by mining the inherent structural characteristics embedded in multivariate data under normal operating conditions. Based on, a multivariate spatio-temporal anomaly interpretation graph is further proposed, which systematically characterizes the sources of anomalies and their correlation mechanisms, thereby revealing the diverse patterns and evolutionary laws of anomaly causes, as shown in Fig. 7b.

Multivariate anomalies exhibit three typical correlation structures in the spatio-temporal dimension, as shown in Fig. 7b(1, 2). The first category is a co-occurrence relationship, as shown in Fig. 7b(1(i–iii, xii)) and Fig. 7b(2(ii, v, vii, xiii)). The key coupling edges in the predicted structure and the natural structure exhibit a significant synchronous distribution, which is consistent with the original spatio-temporal structural pattern of the multivariate data. This indicates that the system remains stable in most submodules. The second category is a partial concomitant phenomenon, as shown in Fig. 7b(1(iv–ix, xiv, xv, xvi)) and Fig. 7b(2(i, iii, vi, x, xi, xv, xvi)). Some key coupling edges in the predicted structure are missing or weakened in the original structure, which suggests that the corresponding subsystems may deviate from the normal operating mode and reflects local functional reconstruction or the cumulative effect of disturbances. The third category is an unstructured disorder, as shown in Fig. 7b(1(iv, x, xi)) and Fig. 7b(2(iv, viii, ix, xii, xiv)). The coupling patterns between the predicted structure and the original structure are highly fragmented without stable correspondence, which demonstrates that the system has entered a severely disordered state. The above classification reveals that the causes of multivariate anomalies can stem from either local changes in coupling relationships or global structural mismatch, which reflects the multi-level analytical capability of the proposed model for anomaly patterns. It also provides quantitative and structured support for subsequent anomaly localization and cause interpretation.

Furthermore, this study takes the water treatment system^54^ as the research object and implements a complete pipeline of anomaly recognition, diagnosis and interpretation, aiming to reveal the intrinsic mechanism of the proposed method, as shown in Supplementary Section 7.

## Discussion

Industrial Internet of Things and complex information systems face challenges in anomaly detection, such as the lack of credibility in manual labeling and the need for anomaly interpretation. For a long time, mainstream methods in this field have been based on Euclidean space for embedding features. Due to the geometric property of Euclidean space, these methods inevitably result in geometric distortion when modeling hierarchical relationships and nonlinear coupling in multivariate data. This leads to a significant deficiency in detecting weak coupling and long-range dependency anomalies. Therefore, this study proposes a hyperbolic adaptive spatial-aware multivariate anomaly detection method. By integrating a dual adaptive discovery strategy that combines hyperbolic space with the natural structure of the data and introduces a downstream task-driven dynamic sparsification method, the model achieves efficient and reliable multivariate anomaly detection along with interpretable cause interpretation. It effectively addresses key challenges, such as insufficient credibility of anomaly labelings, unknown system topology, distortion in data embedding space and dimensional confusion in anomaly interpretation. Validated on publicly available benchmark datasets from multiple domains, including server clusters, industrial control and network security, the proposed method achieves significant performance improvements across the entire process of anomaly recognition, diagnosis and cause interpretation. Specifically, the F1 score improves by at least 8% before and after anomaly label correction, and the AUC value across all datasets remains consistently above 0.84. In anomaly diagnosis tasks, HitRate@600% and NDCG@600% both exceed 89%. The method refines fine-grained anomaly labelings, achieving a normalized data reconstruction error as low as 0.0029, which is more than 30% lower than the optimal reconstruction errors reported by traditional models. It effectively mitigates long-standing issues in the anomaly detection field, such as missing or incorrect manual labeling and establishes a more reliable unified benchmark for objective model evaluation.

Notably, this study observes significant topological isomorphism in medium-scale systems across different domains, which offers important theoretical insights for cross-domain knowledge transfer and zero-sample anomaly detection. Future work could involve constructing a universal natural topology library and developing transfer learning methods based on topological isomorphism. This would significantly reduce the reliance of anomaly detection models on labeled data and promote the large-scale application of anomaly detection technologies in more emerging industrial scenarios.

## Methods

**Fig. 8:**
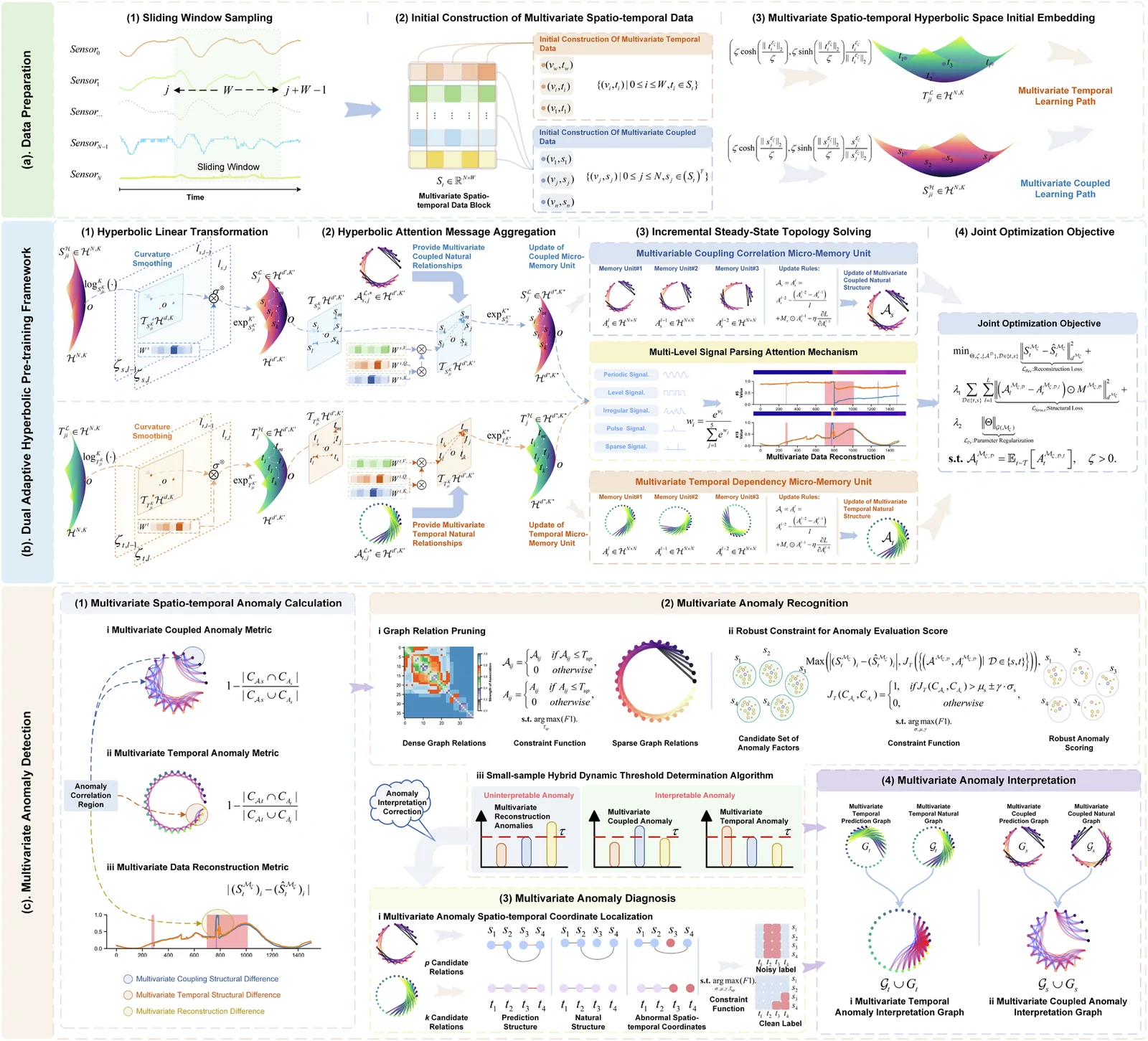
Hyperbolic adaptive spatial-aware multivariate temporal anomaly detection model framework. **a** Data preparation. **a(1)** Shows sliding window sampling. **a(2)** Shows the initial construction of multivariate spatio-temporal data. **a(3)** Shows multivariate spatio-temporal hyperbolic space initial embedding. **b** Dual adaptive hyperbolic pre-training framework. **b(1)** Shows a hyperbolic linear transformation. **b(2)** Shows hyperbolic attention message aggregation. **b(3)** Shows incremental steady-state topology solving, including a multivariate coupling correlation micro-memory unit, a multi-level signal parsing attention mechanism, and a multivariate temporal dependency micro-memory unit. **b(4)** Shows a joint optimization objective. **c** Multivariate anomaly detection. **c(1)** Shows multivariate spatio-temporal anomaly calculation, including (i) a multivariate coupled anomaly metric, (ii) a multivariate temporal anomaly metric, and (iii) a multivariate data reconstruction metric. **c(2)** Shows multivariate anomaly recognition, including (i) graph relation pruning, (ii) robust constraint for anomaly evaluation score, and (iii) a small-sample hybrid dynamic threshold determination algorithm. **c(3)** Shows multivariate anomaly diagnosis, including (i) multivariate anomaly spatio-temporal coordinate localization. **c(4)** Shows multivariate anomaly interpretation, including (i) a multivariate temporal anomaly interpretation graph and (ii) a multivariate coupled anomaly interpretation graph.

In complex industrial systems and Internet of Things (IoT) scenarios, anomaly detection in multivariate time-series data faces multiple challenges, including insufficient credibility of anomaly labels, unknown true system topology, distortion in the data embedding space, and confusion among anomaly interpretation dimensions. To address these issues, we propose a hyperbolic adaptive spatial-aware framework for multivariate time-series anomaly detection, as shown in Fig. 8. By leveraging the hierarchical modeling capabilities of hyperbolic geometry and a dual adaptive pre-training strategy, the framework enables the accurate recognition, diagnosis, and interpretable analysis of multivariate anomalies.

In the data preparation stage, this study first adopts a sliding window sampling strategy (Fig. 8a(1)) to continuously sample the observed time series using a sliding window with step size j and window length W. This generates multivariate spatio-temporal data blocks formulated as ${S}_{t}=\{{x}_{i,t+j}| 1\le i\le N,0\le j < W\}$ which preserves local temporal dependencies while mitigating the risk of global temporal information fragmentation. Based on this study, further constructs multivariate time series data and multivariate coupling graph data, respectively (Fig. 8a(2)). All structured data are then projected into hyperbolic space via the initial hyperbolic embedding module (Fig. 8a(3)), establishing a solid data foundation for discovering the natural spatio-temporal structures of multivariate time series. In the dual adaptive hyperbolic pre-training stage, the hyperbolic linear transformation module is first employed to suppress noise interference from raw data (Fig. 8b(1)). Subsequently, the hyperbolic attention message aggregation mechanism (Fig. 8b(2)) leverages spatial and temporal attention units to capture the natural dynamic topological representations ${{{\mathcal{A}}}}_{s}$ and ${{{\mathcal{A}}}}_{t}$ underlying multivariate coupling correlations and temporal dependencies. To enable incremental optimization of the inherent topological structure, we design multivariate coupling memory units and temporal dependency memory units. The topological representations are iteratively updated via the incremental steady-state topology solving module (Fig. 8b(3)), followed by multivariate data reconstruction using a multi-scale signal parsing attention mechanism. Finally, the joint optimization objective (Fig. 8b(4)) is optimized to achieve dynamic updating of natural multivariate spatio-temporal structures and stable representation learning in hyperbolic space.

In the multivariate anomaly detection stage, anomaly severity is first quantified from three perspectives: coupling structure discrepancy, temporal structure discrepancy, and data reconstruction discrepancy (Fig. 8c(1)), enabling the quantitative calculation of multivariate spatio-temporal anomalies. Subsequently, the graph relation pruning module (Fig. 8c(2(i))) and robust constraint optimization module (Fig. 8c(2(ii))) in the multivariate anomaly recognition procedure are adopted to screen sparse graph relations and anomaly factor sets. This procedure achieves the discrimination between explainable and unexplainable anomalies (Fig. 8c(2(iii))) and outputs reliable anomaly evaluation scores. For anomaly tracing, multivariate anomaly diagnosis is further performed (Fig. 8c(3)). With the spatio-temporal coordinate localization of multivariate anomalies (Fig. 8c(3(i))), the spatio-temporal positions of anomalies and the correlations among key variables are determined, and anomaly causal chains are constructed by integrating candidate relations and predicted topological structures. Finally, a multivariate spatio-temporal anomaly interpretation graph is established (Fig. 8c(4(i, ii))) to interpret the propagation paths and root-cause correlations of detected anomalies.

### Initial embedding of multivariate data

#### Definition of data representation space

The dynamic adjustment of embedding quality for data across different representation spaces ${{\mathcal{M}}}\in \{{{\mathcal{E}}},{{\mathcal{P}}},{{\mathcal{H}}}\}$ depends on the Riemannian metric ${g}^{{{\mathcal{M}}}}$ of the n-dimensional manifold $(n,{g}^{{{\mathcal{M}}}})$, which provides the natural geometric structure of the manifold ${{\mathcal{M}}}$^6^. For any point ${{\boldsymbol{x}}}$ on the manifold ${{\mathcal{M}}}$, there exists a corresponding tangent space ${{{\mathcal{T}}}}_{{{\boldsymbol{x}}}}{{\mathcal{M}}}$, which serves as the first-order approximation of ${{\mathcal{M}}}$ at ${{\boldsymbol{x}}}$ and is an n-dimensional vector space isomorphic to ${{\mathbb{R}}}^{n}$. In this study, the curvature of the Riemannian manifold is defined as $K=-\frac{1}{{\zeta }^{2}}$ with ζ>0, whereas the curvature of Euclidean space corresponds to ζ=0.

Euclidean model (${{\mathcal{E}}}$) refers to the Riemannian manifold $({{{\mathcal{E}}}}_{\zeta }^{n},{g}_{{{\boldsymbol{x}}}}^{{{\mathcal{E}}}})$, where ${{{\mathcal{E}}}}_{\zeta }^{n}={{\mathbb{R}}}^{n}$ denotes the entire space of n-dimensional real vectors and ${g}_{{{\boldsymbol{x}}}}^{{{\mathcal{E}}}}={g}^{{{\mathcal{E}}}}={I}_{n}$ is the standard Euclidean Riemannian metric (identity matrix), which is independent of both the curvature parameter ζ and the position ${{\boldsymbol{x}}}$. Its distance measure, spatial mapping and translation definitions are as follows:

1 $${d}^{{{{\mathcal{E}}}}_{\zeta }}({{\boldsymbol{x}}},{{\boldsymbol{y}}})=\Vert {{\boldsymbol{x}}}-{{\boldsymbol{y}}}{\Vert }_{2}$$

2 $${\log }_{{{\boldsymbol{x}}}}^{{{{\mathcal{E}}}}_{\zeta }}({{\boldsymbol{y}}})={{\boldsymbol{y}}},$$

3 $${\exp }_{{{\boldsymbol{x}}}}^{{{{\mathcal{E}}}}_{\zeta }}({{\boldsymbol{v}}})={{\boldsymbol{v}}},$$

4 $${P}_{{{\boldsymbol{x}}}\to {{\boldsymbol{y}}}}^{{{{\mathcal{E}}}}_{\zeta }}({{\boldsymbol{v}}})={{\boldsymbol{v}}},$$

Poincaré ball model (${{\mathcal{P}}}$)^59^ is a Riemannian manifold $({{{\mathcal{P}}}}_{\zeta }^{n},{g}_{{{\boldsymbol{x}}}}^{{{\mathcal{P}}}})$, where ${{{\mathcal{P}}}}_{\zeta }^{n}=\{{{\boldsymbol{x}}}\in {{\mathbb{R}}}^{n}:{\left\langle {{\boldsymbol{x}}},{{\boldsymbol{x}}}\right\rangle }_{2} < -K\}$ is an open n-dimensional ball of radius $\frac{1}{\zeta }$. The Riemannian metric is given by ${g}_{{{\boldsymbol{x}}}}^{{{\mathcal{P}}}}={({\lambda }_{{{\boldsymbol{x}}}}^{\zeta })}^{2}{g}^{{{\mathcal{E}}}}$, where ${\lambda }_{{{\boldsymbol{x}}}}^{\zeta }=2/(1+\Vert {{\boldsymbol{x}}}{\Vert }_{2}^{2}/{\zeta }^{2})$ is the conformal factor. Its distance measure, spatial mapping and translation definitions are as follows:

5 $${d}^{{{{\mathcal{P}}}}_{\zeta }}({{\boldsymbol{x}}},{{\boldsymbol{y}}})=\zeta {{\rm{arcCosh}}}\left(1-\frac{2{\zeta }^{2}\Vert {{\boldsymbol{x}}}-{{\boldsymbol{y}}}{\Vert }_{2}^{2}}{\left({\zeta }^{2}+\Vert {{\boldsymbol{x}}}{\Vert }_{2}^{2}\right)\left({\zeta }^{2}+\Vert {{\boldsymbol{y}}}{\Vert }_{2}^{2}\right)}\right),$$

6 $${\log }_{{{\boldsymbol{x}}}}^{{{{\mathcal{P}}}}_{\zeta }}({{\boldsymbol{y}}})=\frac{2\zeta }{{\lambda }_{{{\boldsymbol{x}}}}^{\zeta }}{{\rm{arctanh}}}\left(\frac{\Vert -{{\boldsymbol{x}}}{\oplus }_{\zeta }{{\boldsymbol{y}}}{\Vert }_{2}}{\zeta }\right)\frac{-{{\boldsymbol{x}}}{\oplus }_{\zeta }{{\boldsymbol{y}}}}{\Vert -{{\boldsymbol{x}}}{\oplus }_{\zeta }{{\boldsymbol{y}}}{\Vert }_{2}},$$

7 $${\exp }_{{{\boldsymbol{x}}}}^{{{{\mathcal{P}}}}_{\zeta }}({{\boldsymbol{v}}})={{\boldsymbol{x}}}{\oplus }_{\zeta }\left(\tanh \left(\frac{{\lambda }_{{{\boldsymbol{x}}}}^{\zeta }\Vert {{\boldsymbol{v}}}{\Vert }_{2}}{2\zeta }\right)\frac{{{\boldsymbol{v}}}\zeta }{\Vert {{\boldsymbol{v}}}{\Vert }_{2}}\right),$$

8 $${P}_{{{\boldsymbol{x}}}\to {{\boldsymbol{y}}}}^{{{{\mathcal{P}}}}_{\zeta }}({{\boldsymbol{v}}})=\frac{{\lambda }_{{{\boldsymbol{x}}}}^{\zeta }}{{\lambda }_{{{\boldsymbol{y}}}}^{\zeta }}{gyr}[{{\boldsymbol{y}}},-{{\boldsymbol{x}}}]{{\boldsymbol{v}}},$$

where ${\oplus }_{\zeta }$ denotes the Möbius addition operation^60^, which maps tangent vectors back to hyperbolic space.

9 $${{\boldsymbol{x}}}{\oplus }_{\zeta }{{\boldsymbol{y}}}:=\frac{({\zeta }^{2}+2{{\langle }}{{\boldsymbol{x}}},{{\boldsymbol{y}}}{{\rangle }}+\Vert {{\boldsymbol{y}}}{\Vert }_{2}^{2}){{\boldsymbol{x}}}+({\zeta }^{2}-\Vert {{\boldsymbol{x}}}{\Vert }_{2}^{2}){{\boldsymbol{y}}},}{{\zeta }^{2}+2{{\langle }}{{\boldsymbol{x}}},{{\boldsymbol{y}}}{{\rangle }}+{||}{{\boldsymbol{x}}}|{|}_{2}^{2}{||}{{\boldsymbol{y}}}|{|}_{2}^{2}/{\zeta }^{2}}$$

where ${gyr}[\cdot,\cdot ]{{\boldsymbol{v}}}$ denotes the rotation operator, $\left\langle,\right\rangle={v}_{1}\cdot {v}_{1}+{v}_{2}\cdot {v}_{2}+\ldots+{v}_{n}\cdot {v}_{n}$ is the scalar product and $\Vert {{\boldsymbol{v}}}{\Vert }_{2}=\sqrt{\left\langle,\right\rangle }$.

Hyperbolic model (${{\mathcal{H}}}$)^61^ is a Riemannian manifold $({{{\mathcal{H}}}}_{\zeta }^{n},{g}_{{{\boldsymbol{x}}}}^{{{\mathcal{H}}}})$, where ${{{\mathcal{H}}}}_{\zeta }^{n}=\{{{\boldsymbol{x}}}\in {{\mathbb{R}}}^{n+1}:\langle {{\boldsymbol{x}}},{{\boldsymbol{x}}}{\rangle }_{{{\mathcal{H}}}}=K\}$ and the Riemannian metric is given by ${g}_{{{\boldsymbol{x}}}}^{{{\mathcal{H}}}}={diag}([-{1,1},...,1])_{n}$. Its distance measure, spatial mapping and translation definitions are as follows:

10 $${d}^{{{{\mathcal{H}}}}_{\zeta }}({{\boldsymbol{x}}},{{\boldsymbol{y}}})=\zeta {{\rm{arcCosh}}}\left(\frac{\langle {{\boldsymbol{x}}},{{\boldsymbol{y}}}{\rangle }_{{{\mathcal{H}}}}}{{\zeta }{\scriptstyle{2}\atop}}\right),$$

11 $${\log }_{{{\boldsymbol{x}}}}^{{{{\mathcal{H}}}}_{\zeta }}({{\boldsymbol{y}}})={d}^{{{{\mathcal{H}}}}_{\zeta }}({{\boldsymbol{x}}},{{\boldsymbol{y}}})\frac{{{\boldsymbol{y}}}+\frac{1}{{\zeta }^{2}}{\left\langle {{\boldsymbol{x}}},{{\boldsymbol{y}}}\right\rangle }_{{{\mathcal{H}}}}{{\boldsymbol{x}}}}{\Vert {{\boldsymbol{y}}}+\frac{1}{{\zeta }^{2}}{\left\langle {{\boldsymbol{x}}},{{\boldsymbol{y}}}\right\rangle }_{{{\mathcal{H}}}}{{\boldsymbol{x}}}{\Vert }_{{{\mathcal{H}}}}},$$

12 $${\exp }_{{{\boldsymbol{x}}}}^{{{{\mathcal{H}}}}_{\zeta }}({{\boldsymbol{v}}})=\cosh \left(\frac{\Vert {{\boldsymbol{v}}}{\Vert }_{{{\mathcal{H}}}}}{\zeta }\right){{\boldsymbol{x}}}+\zeta \sinh \left(\frac{\Vert {{\boldsymbol{v}}}{\Vert }_{{{\mathcal{H}}}}}{\zeta }\right)\frac{{{\boldsymbol{v}}}}{\Vert {{\boldsymbol{v}}}{\Vert }_{{{\mathcal{H}}}}},$$

13 $${P}_{{{\boldsymbol{x}}}\to {{\boldsymbol{y}}}}^{{{{\mathcal{H}}}}_{\zeta }}({{\boldsymbol{v}}})={{\boldsymbol{v}}}-\frac{{{\langle }}{{\boldsymbol{y}}},{{\boldsymbol{v}}}{{{\rangle }}}_{{{\mathcal{H}}}}}{{\zeta }^{2}+{{\langle }}{{\boldsymbol{x}}},{{\boldsymbol{y}}}{{{\rangle }}}_{{{\mathcal{H}}}}}({{\boldsymbol{x}}}+{{\boldsymbol{y}}}),$$

where $\langle {{\boldsymbol{x}}},{{\boldsymbol{y}}}{\rangle }_{{{\mathcal{H}}}}=-{x}_{0}{y}_{0}+{x}_{1}{y}_{1}+\cdots+{x}_{n}{y}_{n}$ denotes the Lorentz inner product and $\Vert {\Vert }_{{{\mathcal{H}}}}=\sqrt{{\left\langle,\right\rangle }_{{{\mathcal{H}}}}}$ is the Lorentz norm of ${{\boldsymbol{v}}}$.

#### Hyperbolic initial embedding

Due to the dimensional consistency between the Euclidean space ${{\mathcal{E}}}$ and the hyperbolic space ${{\mathcal{P}}}$ (both n-dimensional), as well as the discrepancy caused by the (n+1)-dimensional data representations in the hyperbolic space ${{\mathcal{H}}}$, hyperbolic initial embedding is performed on the sliding-window sampled data ${S}^{{{{\mathcal{E}}}}_{\zeta }}\in {{\mathbb{R}}}^{N\times W}$. Let ${{{\boldsymbol{r}}}}_{0}^{{{{\mathcal{E}}}}_{\zeta }}\in {\left({S}^{{{{\mathcal{E}}}}_{\zeta }}\right)}_{i}\in {{\mathbb{R}}}^{W}$ denote the initial Euclidean embedded feature. Let ${{\boldsymbol{o}}}:=\{1/\zeta,0,\ldots,0\}\in {{{\mathcal{H}}}}_{\zeta }^{W}$ represent the north pole in ${{\mathcal{H}}}$, which acts as the reference point for tangent space operations. Since $\langle \left(0,{{{\boldsymbol{x}}}}^{0,E}\right),{{\boldsymbol{o}}}\rangle=0$, the vector $\left(0,{{{\boldsymbol{r}}}}_{0}^{{{{\mathcal{E}}}}_{\zeta }}\right)$ is regarded as a point in the tangent space ${{{\mathcal{T}}}}_{{{\boldsymbol{o}}}}{{{\mathcal{H}}}}_{\zeta }^{W}$ and is mapped to ${{{\mathcal{H}}}}_{\zeta }^{W}$ via the exponential map ${\exp }_{{{\boldsymbol{x}}}}^{{{{\mathcal{H}}}}_{\zeta }}(\cdot )$, as formulated below:

14 $${{{\boldsymbol{r}}}}_{0}^{{{{\mathcal{H}}}}_{\zeta }}={\exp }_{{{\boldsymbol{o}}}}^{{{{\mathcal{H}}}}_{\zeta }}\left(\left(0,{{{\boldsymbol{r}}}}_{0}^{{{{\mathcal{E}}}}_{\zeta }}\right)\right)=\left(\zeta \cosh \left(\frac{\Vert {{{\boldsymbol{r}}}}_{0}^{{{{\mathcal{E}}}}_{\zeta }}{\Vert }_{2}}{\zeta }\right),\zeta \sinh \left(\frac{\Vert {{{\boldsymbol{r}}}}_{0}^{{{{\mathcal{E}}}}_{\zeta }}{\Vert }_{2}}{\zeta }\right)\frac{{{{\boldsymbol{r}}}}_{0}^{{{{\mathcal{E}}}}_{\zeta }}}{\Vert {{{\boldsymbol{r}}}}_{0}^{{{{\mathcal{E}}}}_{\zeta }}{\Vert }_{2}}\right).$$

### Adaptive hyperbolic pre-training framework

The adaptive hyperbolic pre-training framework (AH-PF) proposed in this study is a lightweight and highly flexible multi-task learning framework. It adaptively optimizes data embedding quality and natural structure discovery by leveraging the curvature and structural characteristics of the data representation space, which includes hyperbolic linear transformation, hyperbolic graph convolution and hyperbolic adaptive graph structure learning.

#### Hyperbolic linear transformation

Hyperbolic linear transformation refers to a linear operation performed in a hyperbolic space with variable curvature. According to Supplementary Theorem 1. To ${{{\mathcal{T}}}}_{{{\boldsymbol{o}}}}{{{\mathcal{H}}}}_{\zeta }$ represents the first-order local approximation of the hyperbolic manifold at point ${{\boldsymbol{x}}}$ and its algebraic properties are similar to those of Euclidean space. Consequently, the Euclidean linear transformation is generalized and extended as follows:

15 $$	{{\boldsymbol{y}}}={{\boldsymbol{x}}}\times {{\bf{W}}}+{{\boldsymbol{b}}}\to {{{\boldsymbol{y}}}}^{{{{\mathcal{M}}}}_{\zeta }}={{{\boldsymbol{x}}}}^{{{{\mathcal{M}}}}_{\zeta }}{\otimes }^{\zeta }{{{\bf{W}}}}^{{{{\mathcal{M}}}}_{\zeta }}{\oplus }^{\zeta }{{{\boldsymbol{b}}}}^{{{{\mathcal{M}}}}_{\zeta }},\\ 	 {{{\boldsymbol{x}}}}^{{{{\mathcal{M}}}}_{\zeta }}{\otimes }^{\zeta }{{{\bf{W}}}}^{{{{\mathcal{M}}}}_{\zeta }}={\exp }_{{{\boldsymbol{o}}}}^{{{{\mathcal{M}}}}_{\zeta }}({\log }_{{{\boldsymbol{o}}}}^{{{{\mathcal{M}}}}_{\zeta }}({{{\boldsymbol{x}}}}^{{{{\mathcal{M}}}}_{\zeta }}){{{\bf{W}}}}^{{{{\mathcal{M}}}}_{\zeta }}),\\ 	{{{\boldsymbol{x}}}}^{{{{\mathcal{M}}}}_{\zeta }}{\oplus }^{\zeta }{{{\boldsymbol{b}}}}^{{{{\mathcal{M}}}}_{\zeta }}={\exp }_{{{\boldsymbol{x}}}}^{{{{\mathcal{M}}}}_{\zeta }}({P}_{{{\boldsymbol{o}}}\to {{\boldsymbol{x}}}}^{{{{\mathcal{M}}}}_{\zeta }}({{{\boldsymbol{b}}}}^{{{{\mathcal{M}}}}_{\zeta }})),$$

where ${{{\mathcal{M}}}}_{\zeta }$ denotes different representation spaces with curvature ζ, where ${{\mathcal{M}}}\in \left\{{{\mathcal{E}}},{{\mathcal{P}}},{{\mathcal{H}}}\right\}$. For example, ${{{\boldsymbol{y}}}}^{{{{\mathcal{P}}}}_{\zeta }}$ indicates that ${{\boldsymbol{y}}}$ resides in the Poincaré ball space with curvature ζ. The operator ${\otimes }^{\zeta }$ represents hyperbolic multiplication and ${\oplus }^{\zeta }$ denotes hyperbolic addition. The mapping ${\exp }_{{{\boldsymbol{x}}}}^{{{{\mathcal{M}}}}_{\zeta }}(\cdot )$ projects a point from the tangent space ${{{\mathcal{T}}}}_{{{\boldsymbol{x}}}}{{{\mathcal{M}}}}_{\zeta }$ to the hyperbolic space ${{{\mathcal{M}}}}_{\zeta }$. Conversely, ${\log }_{{{\boldsymbol{o}}}}^{{{{\mathcal{M}}}}_{\zeta }}(\cdot )$ maps a point from the hyperbolic space ${{{\mathcal{M}}}}_{\zeta }$ to the tangent space ${{{\mathcal{T}}}}_{{{\boldsymbol{o}}}}{{{\mathcal{M}}}}_{\zeta }$. Detailed mappings for different data representation spaces are provided in Section “Definition of Data Representation Space.”

Finally, the hyperbolic nonlinear activation function is defined as follows:

16 $${\sigma }^{{\otimes }^{{{{\mathcal{M}}}}_{{\zeta }_{l-1},{\zeta }_{l}}}}({{{\boldsymbol{x}}}}^{{{{\mathcal{M}}}}_{\zeta }})={\exp }_{{{\boldsymbol{o}}}}^{{{{\mathcal{M}}}}_{{\zeta }_{l}}}(\sigma ({\log }_{{{\boldsymbol{o}}}}^{{{{\mathcal{M}}}}_{{\zeta }_{l-1}}}({{{\boldsymbol{x}}}}^{{{{\mathcal{M}}}}_{\zeta }}))),$$

where ${\sigma }^{{\otimes }^{{{{\mathcal{M}}}}_{{\zeta }_{l-1},{\zeta }_{l}}}}$ denotes the hyperbolic nonlinear activation function. By introducing hyperbolic nonlinear activations with different curvatures at different layers, the model is able to smoothly vary the curvature of each layer. Furthermore, in order to apply the exponential map ${\log }_{{{\boldsymbol{o}}}}^{\zeta }(\cdot )$, the point ${{\boldsymbol{o}}}$ must lie at the north pole of the hyperbolic space. This is because the tangent plane at the north pole is shared across hyperbolic manifolds of the same dimension but with different curvatures, which ensures the validity of the nonlinear activation defined in Eq. 16.

Given the operational redundancy of hyperbolic space, frequent tangent space–hyperbolic space–tangent space conversions incurred in the cascade of traditional linear transformations are alleviated by conducting continuous feature transformations in the tangent space ${{{\mathcal{T}}}}_{{{\boldsymbol{x}}}}{{{\mathcal{H}}}}_{\zeta }$ of the hyperbolic space. The specific definition is given as follows:

17 $${{\rm{HDNN}}}({{{\boldsymbol{x}}}}^{{{{\mathcal{M}}}}_{{\zeta }_{l}}})=	{\sigma }^{{\otimes }^{{{{\mathcal{M}}}}_{{\zeta }_{l-1},{\zeta }_{l}}}}({\exp }_{{{\boldsymbol{o}}}}^{{{{\mathcal{M}}}}_{{\zeta }_{l}}}({{\rm{MLP}}}({\log }_{{{\boldsymbol{o}}}}^{{{{\mathcal{M}}}}_{{\zeta }_{l}}}({{{\boldsymbol{x}}}}^{{{{\mathcal{M}}}}_{{\zeta }_{l-1}}})))\\ 	{\otimes }^{{\zeta }_{l-1}}{{{\bf{W}}}}^{{{{\mathcal{M}}}}_{{\zeta }_{l}}}){\oplus }^{{\zeta }_{l-1}}{{{\boldsymbol{b}}}}^{{{{\mathcal{M}}}}_{{\zeta }_{l}}},$$

where ${{\rm{MLP}}}({\log }_{{{\boldsymbol{o}}}}^{{{{\mathcal{M}}}}_{{\zeta }_{l}}}({{{\boldsymbol{x}}}}^{{{{\mathcal{M}}}}_{{\zeta }_{l}}}))$ denotes the sequential execution of multilayer perceptron (MLP) operations within the tangent space ${{{\mathcal{T}}}}_{{{\boldsymbol{o}}}}{{{\mathcal{M}}}}_{\zeta }$. Specifically, ${{\rm{MLP}}}({{{\boldsymbol{x}}}}^{l})={{{\bf{W}}}}^{l}{{{\boldsymbol{x}}}}^{l-1}+{{{\boldsymbol{b}}}}^{l}$, where ${{{\bf{W}}}}^{l}$ and ${{{\boldsymbol{b}}}}^{l}$ are the weight matrix and bias vector of the l-th layer, respectively.

#### Hyperbolic attention message aggregation

GCN^62^ is a deep learning model that simultaneously processes both node features and structural information in graph data. Traditional GCNs primarily learn and update node features through a message-passing mechanism. For a graph G(V,E), the message-passing process for a node ${v}_{i}\in V$ at layer l is as follows:

18 $${{{\boldsymbol{h}}}}_{i}^{l}={{{\bf{W}}}}^{l}{{{\boldsymbol{x}}}}_{i}^{l-1}+{{{\boldsymbol{b}}}}^{l},\,{{{\boldsymbol{z}}}}_{i}^{l}={{\rm{ReLU}}}({{{\boldsymbol{h}}}}_{i}^{l}+\mathop{\sum }\limits_{j\in {{\mathcal{N}}}(i)}{{{\bf{W}}}}_{{ij}}{{{\boldsymbol{h}}}}_{j}^{l}),$$

where ${{\mathcal{N}}}(i)=\{{j|}(i,j)\in E\}$ denotes the first-order neighborhood of node ${v}_{i}$. In Euclidean space, the weighted average information aggregation takes the form ${\sum }_{j\in {{\mathcal{N}}}{(i)}}{{{\bf{W}}}}_{{ij}}{{{\boldsymbol{h}}}}_{j}^{l}$, where ${{{\bf{W}}}}_{{ij}}$ is the weight associated with neighbor ${v}_{j}\in {{\mathcal{N}}}(i)$ of node ${v}_{i}$ and ${{{\boldsymbol{h}}}}_{j}^{l}$ is the iterative feature of node ${v}_{j}$. However, in hyperbolic space, the weighted average information aggregation does not admit a closed-form solution^63^. According to Supplementary Theorem 1, the attention-based weighted average information aggregation is performed in the tangent space ${{{\mathcal{T}}}}_{{{\boldsymbol{x}}}}{{{\mathcal{M}}}}_{\zeta }$ of node ${{\boldsymbol{x}}}$ within the hyperbolic space ${{{\mathcal{M}}}}_{\zeta }\in \{{{\mathcal{E}}},{{\mathcal{P}}},{{\mathcal{H}}}\}$, as defined below^61^:

19 $$	{{{\boldsymbol{w}}}}_{{ij}}^{{{{\mathcal{M}}}}_{\zeta }}=\frac{\exp ({{\rm{MLP}}}({\log }_{{{\boldsymbol{o}}}}^{{{{\mathcal{M}}}}_{\zeta }}({{{\boldsymbol{x}}}}_{i}^{{{{\mathcal{M}}}}_{\zeta }}\cdot {{{\bf{W}}}}^{{{{\mathcal{M}}}}_{\zeta,Q}})\Vert {\log }_{{{\boldsymbol{o}}}}^{{{{\mathcal{M}}}}_{\zeta }}({{{\boldsymbol{x}}}}_{j}^{{{{\mathcal{M}}}}_{\zeta }}\cdot {{{\bf{W}}}}^{{{{\mathcal{M}}}}_{\zeta,K}})))}{{\sum }_{k\in {{\mathcal{N}}}(i)}\exp ({{\rm{MLP}}}({\log }_{{{\boldsymbol{o}}}}^{{{{\mathcal{M}}}}_{\zeta }}({{{\boldsymbol{x}}}}_{i}^{{{{\mathcal{M}}}}_{\zeta }}\cdot {{{\bf{W}}}}^{{{{\mathcal{M}}}}_{\zeta,Q}})\Vert {\log }_{{{\boldsymbol{o}}}}^{{{{\mathcal{M}}}}_{\zeta }}({{{\boldsymbol{x}}}}_{k}^{{{{\mathcal{M}}}}_{\zeta }}\cdot {{{\bf{W}}}}^{{{{\mathcal{M}}}}_{\zeta,K}})))},\\ 	{{{\rm{HAAG}}}}^{{{{\mathcal{M}}}}_{\zeta }}({{{\boldsymbol{x}}}}_{i}^{{{{\mathcal{M}}}}_{\zeta }})={\exp }_{{{{\boldsymbol{x}}}}_{i}}^{{{{\mathcal{M}}}}_{\zeta }}\left({\sum }_{k\in {{\mathcal{N}}}(i)}{{{\boldsymbol{w}}}}_{{ij}}^{{{{\mathcal{M}}}}_{\zeta }}\cdot {{{\bf{W}}}}^{{{{\mathcal{M}}}}_{\zeta,V}}\cdot {\log }_{{{{\boldsymbol{x}}}}_{i}}^{{{{\mathcal{M}}}}_{\zeta }}({{{\boldsymbol{x}}}}_{k}^{{{{\mathcal{M}}}}_{\zeta }})\right),$$

where ${{{\boldsymbol{w}}}}_{{ij}}^{{{{\mathcal{M}}}}_{\zeta }}$ denotes the importance weight between node ${v}_{i}$ and its neighbor ${v}_{j}$, learned via an attention mechanism^64^. The matrices ${{{\bf{W}}}}^{{{{\mathcal{M}}}}_{\zeta,Q}}$, ${{{\bf{W}}}}^{{{{\mathcal{M}}}}_{\zeta,K}}$ and ${{{\bf{W}}}}^{{{{\mathcal{M}}}}_{\zeta,V}}$ are the weight matrices for the query, key and value vectors, respectively, determining how to extract query information, match key information and retrieve value information from the original features. The operator ${{{\rm{HAAG}}}}^{{{{\mathcal{M}}}}_{\zeta }}\left(\cdot \right)$ represents hyperbolic average aggregation. Finally, the general geometric-space message aggregation designed in this study is defined as follows:

20 $${{{\boldsymbol{h}}}}_{i}^{{{{\mathcal{M}}}}_{{\zeta }_{l}}}=({{{\bf{W}}}}^{{{{\mathcal{M}}}}_{{\zeta }_{l}}}{\otimes }^{{\zeta }_{l-1}}{{{\boldsymbol{x}}}}_{i}^{{{{\mathcal{M}}}}_{{\zeta }_{l-1}}}){\oplus }^{{\zeta }_{l-1}}{{{\boldsymbol{b}}}}^{{{{\mathcal{M}}}}_{{\zeta }_{l}}},$$

21 $${{{\boldsymbol{y}}}}_{i}^{{{{\mathcal{M}}}}_{{\zeta }_{l}}}={{{\rm{HAAG}}}}^{{{{\mathcal{M}}}}_{{\zeta }_{l-1}}}({{{\boldsymbol{h}}}}_{i}^{{{{\mathcal{M}}}}_{{\zeta }_{l}}}),$$

22 $${{{\boldsymbol{x}}}}_{i}^{{{{\mathcal{M}}}}_{{\zeta }_{l}}}={\sigma }^{{\otimes }^{{{{\mathcal{M}}}}_{{\zeta }_{l-1},{\zeta }_{l}}}}({{{\boldsymbol{y}}}}_{i}^{{{{\mathcal{M}}}}_{{\zeta }_{l}}}).$$

#### Multivariate spatio-temporal graph construction and constraint initialization

Multivariate temporal dependency relationships and multivariate coupling correlations are two key analytical dimensions for explaining the causes of multivariate anomalies. To reduce mutual interference among different explanatory perspectives of anomaly causes, the HAO-series models’ independent optimization mechanisms for temporal dependencies and coupling correlations are leveraged, enabling the independent discovery of their natural structures within a unified framework.

Definition of multivariate temporal dependency: by analyzing the transmission laws of temporal causal chains under normal operating modes of the information system, an explanatory benchmark for temporal anomaly causation is established. At time t, the temporal dependency topology graph is defined as ${G}_{t}^{{{{\mathcal{M}}}}_{\zeta,t}}({V}_{t}^{{{{\mathcal{M}}}}_{\zeta,t}},{E}_{t}^{{{{\mathcal{M}}}}_{\zeta,t}},{S}_{t}^{{{{\mathcal{M}}}}_{\zeta,t}})$, where ${V}_{t}^{{{{\mathcal{M}}}}_{\zeta,t}}\in {{\mathbb{R}}}^{W}$ denotes the set of temporal nodes, ${E}_{t}^{{{{\mathcal{M}}}}_{\zeta,t}}\subseteq {V}_{t}^{{{{\mathcal{M}}}}_{\zeta,t}}\times {V}_{t}^{{{{\mathcal{M}}}}_{\zeta,t}}$ characterizes the set of causal transmission edges and ${S}_{t}^{{{{\mathcal{M}}}}_{\zeta,t}}\in {{\mathbb{R}}}^{W\times d}$ is the dynamic feature matrix. The adjacency matrix ${A}_{t}^{{{{\mathcal{M}}}}_{\zeta,t}}\in {{\mathbb{R}}}^{W\times W}$ is regularized via the physical constraint matrix ${M}^{{{{\mathcal{M}}}}_{\zeta,t}}\in {{\mathbb{R}}}^{W\times W}$, ultimately converging to the system’s natural temporal structure ${{{\mathcal{A}}}}^{{{{\mathcal{M}}}}_{\zeta,t}}={{\mathbb{E}}}_{t\sim T}[{A}_{t}^{{{{\mathcal{M}}}}_{\zeta,t}}]$.

Definition of multivariate coupling correlation: by revealing the endogenous correlation patterns among multiple variables during the steady-state operation of the information system, an explanatory benchmark for coupling anomaly causation is established. At time t, the coupling topology graph is defined as ${G}_{t}^{{{{\mathcal{M}}}}_{\zeta,s}}({V}_{t}^{{{{\mathcal{M}}}}_{\zeta,s}},{E}_{t}^{{{{\mathcal{M}}}}_{\zeta,s}},{({S}_{t}^{{{{\mathcal{M}}}}_{\zeta,s}})}^{\top })$, where ${V}_{t}^{{{{\mathcal{M}}}}_{\zeta,s}}\in {{\mathbb{R}}}^{N}$ denotes the set of coupling nodes, ${E}_{t}^{{{{\mathcal{M}}}}_{\zeta,s}}\subseteq {V}_{t}^{{{{\mathcal{M}}}}_{\zeta,s}}\times {V}_{t}^{{{{\mathcal{M}}}}_{\zeta,s}}$ characterizes the set of steady-state association edges and ${({S}_{t}^{{{{\mathcal{M}}}}_{\zeta,s}})}^{\top }\in {{\mathbb{R}}}^{N\times d}$ is the transposed feature matrix. Its adjacency matrix ${A}_{t}^{{{{\mathcal{M}}}}_{\zeta,s}}\in {{\mathbb{R}}}^{N\times N}$ is structurally guided via the physical constraint matrix ${M}^{{{{\mathcal{M}}}}_{\zeta,s}}\in {{\mathbb{R}}}^{N\times N}$, ultimately converging to the system’s natural coupling structure ${{{\mathcal{A}}}}^{{{{\mathcal{M}}}}_{\zeta,s}}={{\mathbb{E}}}_{t\sim T}[{A}_{t}^{{{{\mathcal{M}}}}_{\zeta,s}}]$.

Finally, the initial multivariate coupling correlation, the temporal dependency and their respective constraint matrices are defined as follows:

23 $${M}^{\zeta,t}={A}_{0}^{{{{\mathcal{M}}}}_{\zeta,t}}=\left(\begin{array}{cccc}0 & 1 & \cdots & 1\\ 0 & 0 & \cdots & 1\\ \vdots & \ddots & \ddots & \vdots \\ 0 & \cdots & 0 & 0\end{array}\right)\in {{{\mathcal{M}}}}^{W\times W},{M}^{\zeta,s}={A}_{0}^{{{{\mathcal{M}}}}_{\zeta,s}}=\left(\begin{array}{cccc}0 & 1 & \cdots & 1\\ 0 & 0 & \cdots & 1\\ \vdots & \ddots & \ddots & \vdots \\ 0 & \cdots & 0 & 0\end{array}\right)\in {{{\mathcal{M}}}}^{N\times N},$$

where the diagonal elements of the constraint matrix are set to zero, aiming to promote information interaction among different sensors along the temporal dimension. More importantly, this constraint matrix can directly accommodate systems with known natural topologies, such as the line topology in power systems or the road topology in transportation systems, by simply replacing the matrix parameters to fit such scenarios. This property further enhances the reliability of natural topological learning.

#### Adaptive hyperbolic graph structure discovery

Dynamic embedding modeling in hyperbolic space: adaptive hyperbolic graph structure discovery dynamically reveals the multi-view topological properties of an information system under normal operating modes, identifies the system’s topological steady state and serves as a reference benchmark for explaining anomaly causes. Based on the perspectives of multivariate temporal dependency (t) and multivariate coupling correlation (s), HDNN is employed to dynamically adjust the curvature for data embedding, thereby improving embedding quality and reducing data noise perturbations. The formulation is defined as follows:

24 $${F}_{t}^{{{{\mathcal{M}}}}_{\zeta,{{\mathcal{D}}}}}={{{\rm{HDNN}}}}^{{{{\mathcal{M}}}}_{\zeta,{{\mathcal{D}}}}}\left({\Vert }_{i=0}^{i=|{{{\mathcal{M}}}}_{\zeta,{{\mathcal{D}}}}|}{\exp }_{{{\boldsymbol{o}}}}^{{{{\mathcal{M}}}}_{\zeta,{{\mathcal{D}}}}}\left(\left(0,{\left({X}_{t}^{{{{\mathcal{M}}}}_{\zeta,{{\mathcal{D}}}}}\right)}_{i}\right)\right),{\theta }^{{{{\mathcal{M}}}}_{\zeta,{{\mathcal{D}}}}}\right),{{\mathcal{D}}}\in \{t,s\}.$$

where ${F}_{t}^{{{{\mathcal{M}}}}_{\zeta,{{\mathcal{D}}}}}$ denotes the embedded feature at time t in the representation space ${{\mathcal{M}}}$ with curvature ζ and perspective ${{\mathcal{D}}}$. The term ${\left({X}_{t}^{{{{\mathcal{E}}}}_{\zeta,{{\mathcal{D}}}}}\right)}_{i}$ represents the initial row vector feature of ${{\mathcal{D}}}$; under the temporal perspective t, ${X}_{t}^{{{{\mathcal{M}}}}_{\zeta,t}}={S}_{i}^{{{{\mathcal{E}}}}_{\zeta }}\in {{\mathbb{R}}}^{N\times M}$ and $|{{{\mathcal{M}}}}_{\zeta,t}|=W$; under the coupling perspective s, ${X}_{t}^{{{{\mathcal{M}}}}_{\zeta,s}}={({S}_{i}^{{{{\mathcal{E}}}}_{\zeta }})}^{\top }\in {{\mathbb{R}}}^{W\times N}$ and $|{{{\mathcal{M}}}}_{\zeta,s}|=N$. The parameter ${\theta }^{{{{\mathcal{M}}}}_{\zeta,{{\mathcal{D}}}}}$ corresponds to the learnable model parameters for different perspectives.

Topology optimization under physical constraints: to improve the compatibility between the perspective topological attribute ${{{\mathcal{A}}}}_{t}^{{{{\mathcal{M}}}}_{\zeta,{{\mathcal{D}}}}}$ and the perspective feature ${F}_{t}^{{{{\mathcal{M}}}}_{\zeta,{{\mathcal{D}}}}}$, a physical constraint mask matrix ${M}^{\zeta,{{\mathcal{D}}}}$ is introduced to prune and optimize the natural adjacency matrix. Feature-driven topology fine-tuning is then performed via HGCN:

25 $${Z}_{t}^{{{{\mathcal{M}}}}_{\zeta,{{\mathcal{D}}}}}={{{\rm{HGCN}}}}^{{{{\mathcal{M}}}}_{\zeta,{{\mathcal{D}}}}}\left({V}_{t}^{{{{\mathcal{M}}}}_{\zeta,{{\mathcal{D}}}}},{{{\mathcal{A}}}}^{{{{\mathcal{M}}}}_{\zeta,{{\mathcal{D}}}}}\odot {M}^{\zeta,{{\mathcal{D}}}},{F}_{t}^{{{{\mathcal{M}}}}_{\zeta,{{\mathcal{D}}}}},{\theta }^{{{{\mathcal{M}}}}_{\zeta,{{\mathcal{D}}}}}\right),$$

26 $$({A}_{t}^{{{{\mathcal{M}}}}_{\zeta,{{\mathcal{D}}}}})_{{ij}}={\left[1+{e}^{-{d}^{{{{\mathcal{M}}}}_{\zeta,D}}\left(({Z}_{t}^{{{{\mathcal{M}}}}_{\zeta,{{\mathcal{D}}}}})_{i},({Z}_{t}^{{{{\mathcal{M}}}}_{\zeta,{{\mathcal{D}}}}})_{j}\right)/\zeta }\right]}^{-1}.$$

Incremental steady-state topology solving: the natural structure of different perspectives ${{{\mathcal{A}}}}^{{{{\mathcal{M}}}}_{\zeta,{{\mathcal{D}}}}}\in {{\mathbb{R}}}^{l\times l}$(where $l=|{{{\mathcal{M}}}}_{\zeta,{{\mathcal{D}}}}|$) is defined as a function matrix, which represents the limit convergence of the dynamic adjacency matrix during the training process.

27 $${{{\mathcal{A}}}}^{{{{\mathcal{M}}}}_{\zeta,{{\mathcal{D}}}}}=\left(\begin{array}{cccc}{{\mathrm{lim}}}_{t\to \infty }({A}_{t}^{{{{\mathcal{M}}}}_{\zeta,{{\mathcal{D}}}}})_{{0,0}} & {{\mathrm{lim}}}_{t\to \infty }({A}_{t}^{{{{\mathcal{M}}}}_{\zeta,{{\mathcal{D}}}}})_{{0,1}} & \cdots & {{\mathrm{lim}}}_{t\to \infty }({A}_{t}^{{{{\mathcal{M}}}}_{\zeta,{{\mathcal{D}}}}})_{0,l}\\ {{\mathrm{lim}}}_{t\to \infty }({A}_{t}^{{{{\mathcal{M}}}}_{\zeta,{{\mathcal{D}}}}})_{{\mathrm{1,0}}} & {{\mathrm{lim}}}_{t\to \infty }({A}_{t}^{{{{\mathcal{M}}}}_{\zeta,{{\mathcal{D}}}}})_{{\mathrm{1,1}}} & \cdots & {{\mathrm{lim}}}_{t\to \infty }({A}_{t}^{{{{\mathcal{M}}}}_{\zeta,{{\mathcal{D}}}}})_{1,l}\\ \vdots & \ddots & \ddots & \vdots \\ {{\mathrm{lim}}}_{t\to \infty }({A}_{t}^{{{{\mathcal{M}}}}_{\zeta,{{\mathcal{D}}}}})_{l,0} & \cdots & {{\mathrm{lim}}}_{t\to \infty }({A}_{t}^{{{{\mathcal{M}}}}_{\zeta,{{\mathcal{D}}}}})_{l,l-1} & {{\mathrm{lim}}}_{t\to \infty }({A}_{t}^{{{{\mathcal{M}}}}_{\zeta,{{\mathcal{D}}}}})_{l,l}\end{array}\right),$$

where each element of ${{{\mathcal{A}}}}^{{{{\mathcal{M}}}}_{\zeta,{{\mathcal{D}}}}}$ is the converged value of the local topological attribute ${\left({A}_{t}^{{{{\mathcal{M}}}}_{\zeta,{{\mathcal{D}}}}}\right)}_{{ij}}$ over the training process as t→T, with the proof detailed in Supplementary Equation 9 is abbreviated as ${{{\mathcal{A}}}}^{{{{\mathcal{M}}}}_{\zeta,{{\mathcal{D}}}}}={[{{\mathrm{lim}}}_{t\to \infty }({A}_{t}^{{{{\mathcal{M}}}}_{\zeta,{{\mathcal{D}}}}})_{{ij}}]}_{l\times l}$. Although Supplementary Theorems 3, 4, and 5 all indicate convergence under long observation windows, establishing a one-to-one correspondence between the topological attributes of different datasets and their convergence functions remains challenging. Therefore, deep learning is first leveraged to dynamically learn the topological approximation function f(⋅) for local topological attribute nodes, defined as follows:

28 $$({{\mathcal{A}}}^{{{\mathcal{M}}}_{\zeta,{{\mathcal{D}}}}})_{{ij}}\approx f\left(\{{A}_{{ij}}^{{{{\mathcal{M}}}}_{\zeta,{{\mathcal{D}}}}}\}_{t=0}^{t=T},\{{{{\boldsymbol{\theta }}}}^{{{{\mathcal{M}}}}_{\zeta,{{\mathcal{D}}}}}\}_{t=0}^{t=T}\right),$$

where ${{{\boldsymbol{\theta }}}}^{{{{\mathcal{M}}}}_{\zeta,{{\mathcal{D}}}}}$ denotes the learnable parameters of the topological approximation function. Due to the influence of data fluctuations during the learning process, the learned natural structure exhibits structural oscillations under different data distributions. Numerical solving of the natural structure is therefore performed again based on expectations and the physical constraint matrix is imposed to guide it toward convergence, as defined below:

29 $$({{{\mathcal{A}}}}^{{{{\mathcal{M}}}}_{\zeta,{{\mathcal{D}}}}})_{{ij}}={{\mathbb{E}}}_{t\sim T}\left[({A}_{t}^{{{{\mathcal{M}}}}_{\zeta,{{\mathcal{D}}}}}\odot {M}^{{{{\mathcal{M}}}}_{\zeta,{{\mathcal{D}}}}})_{{ij}}\right]=\frac{1}{T}\mathop{\sum }\limits_{i=1}^{T}({A}_{t}^{{{{\mathcal{M}}}}_{\zeta,{{\mathcal{D}}}}}\odot {M}^{{{{\mathcal{M}}}}_{\zeta,{{\mathcal{D}}}}})_{{ij}},$$

where $({{{\mathcal{A}}}}^{{{{\mathcal{M}}}}_{\zeta,{{\mathcal{D}}}}})_{{ij}}$ denotes the weight of the final stable natural system topology; ${{\mathbb{E}}}_{t\sim T}[\cdot ]$ denotes the expectation operation over the full time series; ${A}_{t}^{{{{\mathcal{M}}}}_{\zeta,{{\mathcal{D}}}}}$ is the topological adjacency matrix learned at a single time step; ${M}^{{{{\mathcal{M}}}}_{\zeta,{{\mathcal{D}}}}}$ denotes the physical constraint matrix; and ⊙ denotes the element-wise multiplication operation. By integrating global temporal information via temporal expectation averaging and correcting structural deviations caused by data fluctuations through the physical constraint matrix, this formula effectively eliminates topological oscillation induced by single-time-step data disturbance and achieves stable convergence of the learned natural topology.

It is found that the expectation-based data solving process leads to a significant increase in computer memory resource consumption due to the storage of historical local structures. Therefore, the solution of this process is optimized as follows:

30 $${{{\mathcal{A}}}}_{k+1}^{{{{\mathcal{M}}}}_{\zeta,{{\mathcal{D}}}}}=\frac{1}{k}\mathop{\sum }\limits_{i=1}^{k}{A}_{i}^{{{{\mathcal{M}}}}_{\zeta,{{\mathcal{D}}}}},k={\mathrm{1,2}},\cdots {{{\mathcal{A}}}}_{k}^{{{{\mathcal{M}}}}_{\zeta,{{\mathcal{D}}}}}=\frac{1}{k-1}\mathop{\sum }\limits_{i=1}^{k-1}{A}_{i}^{{{{\mathcal{M}}}}_{\zeta,{{\mathcal{D}}}}},k={\mathrm{2,3}},\cdots,$$

Therefore, ${A}_{k+1}^{{{{\mathcal{M}}}}_{\zeta,{{\mathcal{D}}}}}$ is solved recursively using ${A}_{k}^{{{{\mathcal{M}}}}_{\zeta,{{\mathcal{D}}}}}$:

31 $${{{\mathcal{A}}}}_{k+1}^{{{{\mathcal{M}}}}_{\zeta,{{\mathcal{D}}}}}	=\frac{1}{k}\mathop{\sum }\limits_{i=1}^{k}{A}_{i}^{{{{\mathcal{M}}}}_{\zeta,{{\mathcal{D}}}}}=\frac{1}{k}\left(\mathop{\sum }\limits_{i=1}^{k-1}{A}_{i}^{{{{\mathcal{M}}}}_{\zeta,{{\mathcal{D}}}}}+{A}_{k}^{{{{\mathcal{M}}}}_{\zeta,{{\mathcal{D}}}}}\right)=\frac{1}{k}\left((k-1){{{\mathcal{A}}}}_{k}^{{{{\mathcal{M}}}}_{\zeta,{{\mathcal{D}}}}}+{A}_{k}^{{{{\mathcal{M}}}}_{\zeta,{{\mathcal{D}}}}}\right)\\ 	={{{\mathcal{A}}}}_{k}^{{{{\mathcal{M}}}}_{\zeta,{{\mathcal{D}}}}}-\frac{1}{k}\left({{{\mathcal{A}}}}_{k}^{{{{\mathcal{M}}}}_{\zeta,{{\mathcal{D}}}}}-{A}_{k}^{{{{\mathcal{M}}}}_{\zeta,{{\mathcal{D}}}}}\right).$$

This recursive form reduces memory usage from O(T) to O(1), requiring only three historical state variables to be maintained. The final steady-state topology ${{{\mathcal{A}}}}^{{{{\mathcal{M}}}}_{\zeta,{{\mathcal{D}}}}}={{{\mathcal{A}}}}_{k}^{{{{\mathcal{M}}}}_{\zeta,{{\mathcal{D}}}}}\odot {M}^{{{{\mathcal{M}}}}_{\zeta,{{\mathcal{D}}}}}$ serves as a reference benchmark for the system’s normal operating mode, providing an interpretable topological comparison basis for anomaly cause analysis. Furthermore, since the computation of k during the solving process requires memory space for three historical topological structures, the multi-view micro-memory unit of the HAO model consists of three units.

#### Multi-level signal parsing attention mechanism (MSCD)

Due to the presence of complex and diverse signal forms in multivariate data, such as periodic signals, level signals and sparse signals, traditional multilayer perceptrons struggle to effectively capture different signal features across multiple scales, leading to insufficient fitting performance with the original signals. To address this, a multi-level signal parsing attention mechanism is proposed, which dynamically adjusts the number of neurons according to the scale characteristics of the data block (${S}_{t}\in {{{\mathcal{M}}}}^{N\times W}$), so as to accurately capture the data features of multivariate variables. The specific definition is as follows:

32 $${\hat{S}}_{t}=\mathop{\sum }\limits_{l=1}^{L}{w}_{l}\times {F}_{t},{w}_{l}=\frac{{e}^{{w}_{l}}}{{\sum }_{l=1}^{j}{e}^{{w}_{j}}},$$

where L denotes the number of layers for signal parsing. The terms ${w}_{1}\in {{{\mathcal{M}}}}^{N\times W},{w}_{2}\in {{{\mathcal{M}}}}^{N+W},{w}_{3}\in {{{\mathcal{M}}}}^{N},{w}_{4}\in {{{\mathcal{M}}}}^{W}$ and ${w}_{5}\in {{{\mathcal{M}}}}^{C}$ represent constant scales determined by the dynamic perception mechanism of the multi-level signal parsing attention mechanism, used to set the number of neurons.

### Joint optimization objective

The HAO model achieves joint optimization of multivariate data reconstruction and multi-view low-coupling system steady-state structure discovery through multi-task collaborative learning on the hyperbolic manifold. Its core architecture comprises an adaptive hyperbolic pre-training framework and a natural structure discovery module, which overcome the limitations of traditional Euclidean space modeling via hierarchical representation learning and dynamic structure decoupling mechanisms in the hyperbolic geometric space.

Adaptive hyperbolic pre-training adopts a data geometry-driven strategy, leveraging dynamic adaptation of the curvature of Riemannian manifolds to achieve optimal alignment with the data’s topological properties, thereby enhancing the HAO model’s data reconstruction capability. This reconstruction process is defined as follows:

33 $${\hat{S}}_{t}^{{{{\mathcal{M}}}}_{\zeta }}={{\rm{MSCD}}}\left({\log }_{{{\boldsymbol{o}}}}^{{{{\mathcal{M}}}}_{\zeta }}\left({{{\rm{HDNN}}}}^{{{{\mathcal{M}}}}_{\zeta }}\left({Z}_{t}^{{{{\mathcal{M}}}}_{\zeta,t}}\cdot {({Z}_{t}^{{{{\mathcal{M}}}}_{\zeta,s}})}^{\top }\right)\right)\parallel {S}_{t}^{{{{\mathcal{M}}}}_{\zeta }}\right)\in {{\mathbb{R}}}^{N\times W},$$

where the adaptive hyperbolic pre-training framework multiplies the temporal feature matrix ${Z}_{t}^{{{{\mathcal{M}}}}_{\zeta,t}}$ by the spatial coupling matrix ${Z}_{t}^{{{{\mathcal{M}}}}_{\zeta,s}}$, which reduces the computational complexity from $O({NWd}{\prime} )$ to $O((N+W)d{\prime} )$, thereby effectively alleviating the curse of dimensionality^65^. Meanwhile, the hyperbolic logarithmic mapping ${\log }_{{{\boldsymbol{o}}}}^{{{{\mathcal{M}}}}_{\zeta }}$ projects the latent space representations onto the tangent space and concatenates them with the original signal ${S}_{t}^{{{{\mathcal{M}}}}_{\zeta }}$ at the feature level, which forms a gradient-stable pathway to suppress representation degradation in deep networks^66^.

Natural structure discovery is based on the hyperbolic graph convolutional network. By implementing a dynamic matching mechanism between the geometric topological properties of the data and the node neighborhoods, it aims to enhance the ability to discover latent structures. This dynamic discovery process is defined as follows:

34 $${Z}_{t}^{{{{\mathcal{M}}}}_{\zeta,l,{{\mathcal{D}}}}}	={\sigma }^{{\otimes }^{{{{\mathcal{M}}}}_{{\zeta }_{l-1},{\zeta }_{l}}}}\left({{{\rm{HGCN}}}}^{{{{\mathcal{M}}}}_{\zeta,l,{{\mathcal{D}}}}}\left({{{\rm{HDNN}}}}^{{{{\mathcal{M}}}}_{\zeta,l,{{\mathcal{D}}}}}({S}_{t}^{{{{\mathcal{M}}}}_{\zeta,{{\mathcal{D}}}}}),{{{\mathcal{A}}}}_{l}^{{{{\mathcal{M}}}}_{\zeta,{{\mathcal{D}}}}}\odot {M}^{{{{\mathcal{M}}}}_{\zeta,{{\mathcal{D}}}}}\right)\right),{{\mathcal{D}}}{{\mathscr{\in }}}\{t,s\},\\ {{{\mathcal{A}}}}_{l+1}^{{{{\mathcal{M}}}}_{\zeta,{{\mathcal{D}}}}}	={{{\mathcal{A}}}}_{l}^{{{{\mathcal{M}}}}_{\zeta,{{\mathcal{D}}}}}-\frac{1}{l}\left({{{\mathcal{A}}}}_{l}^{{{{\mathcal{M}}}}_{\zeta,{{\mathcal{D}}}}}-{A}_{t}^{{{{\mathcal{M}}}}_{\zeta,l,{{\mathcal{D}}}}}\right),$$

where natural structure discovery achieves the adaptive evolution of the system’s steady-state topological structure ${{{\mathcal{A}}}}^{{{{\mathcal{M}}}}_{\zeta,{{\mathcal{D}}}}}$ through two mechanisms: a dynamic adjacency decay mechanism, where the hierarchical coefficient 1/l progressively suppresses noise in the initial adjacency matrix ${{{\mathcal{A}}}}_{l}$ and a mask constraint mechanism, where the Hadamard product ${A}_{t}\odot {M}_{t}$ controls the strength of cross-perspective interactions. The curvature ζ is dynamically adjusted during training to ensure that the degree of bending in the geometric space matches the hierarchical structure of the data.

Joint optimization objective of the HAO model: the objective is to unify data reconstruction, natural structure discovery and geometric parameter learning into a multi-task constrained problem:

35 $$\begin{array}{c}{\min }_{\varTheta,\zeta,\left\{{{{\mathcal{A}}}}^{{{\mathcal{D}}}}\right\},{{\mathcal{D}}}{{\in }}\left\{t,s\right\}}\underbrace{{{||}{S}_{t}^{{{{\mathcal{M}}}}_{\zeta }}-{\hat{S}}_{t}^{{{{\mathcal{M}}}}_{\zeta }}{||}}_{{d}^{{{{\mathcal{M}}}}_{\zeta }}}^{2}}_{{{\rm{I}}}}+{\lambda }_{1}\underbrace{{\sum }_{{{\mathcal{D}}}{{\in }}\left\{t,s\right\}}{\sum }_{l=1}^{L}{{||}\left({{{\mathcal{A}}}}_{l}^{{{{\mathcal{M}}}}_{\zeta,{{\mathcal{D}}}}}-{A}_{t}^{{{{\mathcal{M}}}}_{\zeta,{{\mathcal{D}}},l}}\right)\odot {M}^{{{{\mathcal{M}}}}_{\zeta,{{\mathcal{D}}}}}{||}}_{{d}^{{{{\mathcal{M}}}}_{\zeta }}}^{2}}_{{{\rm{II}}}} \\+{\lambda }_{2}\underbrace{{\sum }_{{{\mathcal{D}}}{{\in }}\left\{t,s\right\}}({{||}{Z}_{t}^{{{{\mathcal{M}}}}_{\zeta,{{\mathcal{D}}}}}{||}}_{{d}^{{{{\mathcal{M}}}}_{\zeta }*}}+{{||}{M}^{{{{\mathcal{M}}}}_{\zeta,{{\mathcal{D}}}}}{||}}_{1,\varGamma })}_{{{\rm{III}}}}+{\lambda }_{3}\underbrace{{\sum }_{k=1}^{K}{{||}{\nabla }_{{\zeta }_{k}}{\log }_{{{\bf{o}}}}^{{{{\mathcal{M}}}}_{{\zeta }_{k}}}{||}}_{{{\rm{HS}}}({{{\mathcal{M}}}}_{\zeta })}}_{{{\rm{IV}}}} \\+{\lambda }_{4}\underbrace{{{||}\varTheta {||}}_{{{\mathcal{G}}}({{{\mathcal{M}}}}_{\zeta })}}_{{{\rm{V}}}} s.t.\,\underbrace{{{\langle }}{\pi }_{{{{\mathcal{M}}}}_{\zeta }}\left({S}_{t}^{{{{\mathcal{D}}}}_{i}}\right),{\pi }_{{{{\mathcal{M}}}}_{\zeta }}({S}_{t}^{{{{\mathcal{D}}}}_{j}}){{{\rangle }}}_{{{{\mathcal{T}}}}_{{{\bf{o}}}}{{{\mathcal{M}}}}_{\zeta }}{{\preceq }}0}_{{{\rm{VI}}}}\left(\forall {{{\mathcal{D}}}}_{i}\ne {{{\mathcal{D}}}}_{j}\right){,}\underbrace{{{{\mathcal{A}}}}_{l}^{{{{\mathcal{M}}}}_{\zeta,{{\mathcal{D}}}}}={{\mathbb{E}}}_{t{{\sim }}{{\mathcal{T}}}}\left[{A}_{t}^{{{{\mathcal{M}}}}_{\zeta,{{\mathcal{D}}},l}}\right]}_{{{\rm{VII}}}}{{,}}\zeta > 0,\end{array}$$

where ${{\rm{I}}}$ denotes the geodesic reconstruction loss; ${{\rm{II}}}$ measures the degree of variation of the natural structure ${{{\mathcal{A}}}}_{l}^{{{{\mathcal{M}}}}_{\zeta,{{\mathcal{D}}}}}$ obtained at the l-th iteration, evaluated at time t as ${A}_{l}^{{{{\mathcal{M}}}}_{\zeta,{{\mathcal{D}}},l}}$, wich is subject to the constraint imposed by the physical constraint matrix ${M}^{{{{\mathcal{M}}}}_{\zeta,{{\mathcal{D}}}}}$; ${{\rm{III}}}$ represents the hyperbolic low-rank constraint, together with $\Vert \cdot {\Vert }_{1,\varGamma }$, which enforces sparsity suppression on the off-diagonal elements of the physical constraint matrix across domains; ${{\rm{IV}}}$ denotes curvature gradient stability, where Riemannian metric computation for the HS norm is defined as ${{||}{\nabla }_{\zeta }{\log }_{{{\bf{o}}}}{||}}_{{{\rm{HS}}}({{{\mathcal{M}}}}_{\zeta })}:={[{\sum }_{i,j}{(\frac{\partial }{\partial \zeta }{\log }_{{{\bf{o}}}}^{{{{\mathcal{M}}}}_{\zeta }}({x}^{({ij})}))}^{2}]}^{1/2}$; ${{\rm{V}}}$ stands for geometric parameter regularization, where its computation is defined as ${{||}\varTheta {||}}_{{{\mathcal{G}}}({{{\mathcal{M}}}}_{\zeta })}:={[{\sum }_{k=1}^{d}\zeta \cdot {\cosh }^{2}(\sqrt{\zeta }{{||}{\theta }^{(k)}{||}}_{{d}^{{{{\mathcal{M}}}}_{\zeta }}}){{||}{\theta }^{(k)}{||}}_{{d}^{{{{\mathcal{M}}}}_{\zeta }}}^{2}]}^{1/2}$. Among the constraints, ${{\rm{VI}}}$ imposes low-coupling characteristics between different perspectives, while ${{\rm{VII}}}$ captures the expectation of historical local structures of the natural structure over l iterations.

To address the two core characteristics of multivariate temporal data, namely temporal dependency and inter-variable coupling, a joint optimization framework based on deep learning is proposed. By using a curvature-adaptive mechanism to dynamically regulate gradient dependencies, the framework transforms the original optimization objective in Eq. 35 into the following differentiable form:

36 $$\begin{array}{c}{\min }_{\varTheta,\zeta,\{{{\mathcal{A}}}^{{{\mathcal{D}}}},{{\mathcal{D}}}\in{t,s\}}}\underbrace{{{||}{S}_{t}^{{{{\mathcal{M}}}}_{\zeta }}-{\hat{S}}_{t}^{{{{\mathcal{M}}}}_{\zeta }}{||}}_{{d}^{{{{\mathcal{M}}}}_{\zeta }}}^{2}}_{{{{\mathcal{L}}}}_{{Rec}}:{ReconstructionLoss}} \\+{\lambda }_{1}{\sum }_{{{\mathcal{D}}}{{\in }}\{{t,s\}}{\sum }_{l=1}^{L}\underbrace{{{||}\left({{{\mathcal{A}}}}_{l}^{{{{\mathcal{M}}}}_{\zeta,{{\mathcal{D}}}}}-{A}_{t}^{{{{\mathcal{M}}}}_{\zeta,{{\mathcal{D}}},l}}\right)\odot {M}^{{{{\mathcal{M}}}}_{\zeta,{{\mathcal{D}}}}}{||}}_{{d}^{{{{\mathcal{M}}}}_{\zeta }}}^{2}}_{{{{\mathcal{L}}}}_{{Struct}}:{StructuralLoss}}} \\+{\lambda }_{2}\underbrace{{{||}\varTheta {||}}_{{{\mathcal{G}}}({{{\mathcal{M}}}}_{\zeta })}}_{{{{\mathcal{L}}}}_{\Pr }:{ParameterRegularization}}\\ s.t.{{{\mathcal{A}}}}_{l}^{{{{\mathcal{M}}}}_{\zeta,{{\mathcal{D}}}}}={{\mathbb{E}}}_{t{{\sim }}{{\mathcal{T}}}}\left[{A}_{t}^{{{{\mathcal{M}}}}_{\zeta,{{\mathcal{D}}},l}}\right],\zeta > 0.\end{array}$$

To solve the joint optimization problem formulated in Eq. 36, a hierarchical gradient updating mechanism is designed. The total gradient can be expressed as the sum of three components:

37 $${\nabla }_{\{\varTheta,\zeta,{{\mathcal{A}}}\}}{{\mathcal{L}}}=\underbrace{\frac{\partial {{{\mathcal{L}}}}_{{{\rm{Rec}}}}}{\partial \{\cdot \}}}_{{ReconstructGradient}}+{\lambda }_{1}\underbrace{\frac{\partial {{{\mathcal{L}}}}_{{{\rm{Struct}}}}}{\partial \{\cdot \}}}_{{StructuralGradient}}+{\lambda }_{2}\underbrace{\frac{\partial {{{\mathcal{L}}}}_{\Pr }}{\partial \{\cdot \}}}_{{RegularizationGradient}}.$$

The geometric parameters Θ of the HAO model are updated as follows:

38 $${\varTheta }^{(l+1)}={\varTheta }^{(l)}-{\eta }_{\varTheta }\left[\frac{\partial {d}^{{{{\mathcal{M}}}}_{\zeta }}{({S}_{t},{\hat{S}}_{t})}^{2}}{\partial \varTheta }+{\lambda }_{2}{\nabla }_{\varTheta }\Vert \varTheta {\Vert }_{{{\mathcal{G}}}({{{\mathcal{M}}}}_{\zeta })}\right],$$

where the gradient of the geometric regularization term is computed via the chain rule:

39 $${\nabla }_{\varTheta }\Vert \varTheta {\Vert }_{{{\mathcal{G}}}({{{\mathcal{M}}}}_{\zeta })}=\frac{\partial \Vert \varTheta {\Vert }_{{{\mathcal{G}}}}}{\partial {{\mathcal{G}}}}\cdot \frac{\partial {{\mathcal{G}}}({{{\mathcal{M}}}}_{\zeta })}{\partial \zeta }\cdot \frac{\partial \zeta }{\partial \varTheta }.$$

The curvature ζ is adaptively updated using Riemannian gradient descent:

40 $${\zeta }^{(l+1)}={\zeta }^{(l)}\odot \exp \left(-{\eta }_{\zeta }\left(\frac{\partial {d}^{{{{\mathcal{M}}}}_{\zeta }}{({S}_{t},{\hat{S}}_{t})}^{2}}{\partial \zeta }+{\lambda }_{1}\frac{\partial \Vert \varDelta {{\mathcal{A}}}{\Vert }^{2}}{\partial \zeta }\right)\right),$$

where the exponential map ensures compliance with the constraint ζ>0.

For the update of the natural structure matrix ${{{\mathcal{A}}}}^{{{\mathcal{D}}}}$, a hybrid strategy that integrates gradient-based solving with numerical methods is adopted. First, according to Eqs. 26 and 34 and leveraging the matching mechanism between hierarchical data structures and neighborhood configurations, the local structure with respect to metadata is generated, denoted as ${A}_{t}^{{{{\mathcal{M}}}}_{\zeta,{{\mathcal{D}}},l}}$. Second, the local structure is updated using a gradient-based solving approach.

41 $${A}_{t}^{{{{\mathcal{M}}}}_{\zeta,{{\mathcal{D}}},l+1}}={A}_{t}^{{{{\mathcal{M}}}}_{\zeta,{{\mathcal{D}}},l}}\odot {M}^{{{{\mathcal{M}}}}_{\zeta,{{\mathcal{D}}}}}-\eta \left[\frac{\partial {d}^{{{{\mathcal{M}}}}_{\zeta }}{({S}_{t},{\hat{S}}_{t})}^{2}}{\partial {A}_{t}^{{{{\mathcal{M}}}}_{\zeta,{{\mathcal{D}}},l}}}+{\lambda }_{1}\frac{\partial \Vert \varDelta {{\mathcal{A}}}{\Vert }^{2}}{\partial \zeta }+{\lambda }_{2}{\nabla }_{\varTheta }\Vert \varTheta {\Vert }_{{{\mathcal{G}}}({{{\mathcal{M}}}}_{\zeta })}\right],$$

where η denotes the learning rate. Subsequently, based on the local structure, the natural structural component is obtained by solving Eq. 31.

Finally, to establish the convergence proof for the joint optimization objective, the following assumptions are introduced: (1) Lipschitz continuity: The loss function ${{\mathcal{L}}}(\varTheta,\zeta,{{\mathcal{A}}})$ satisfies $\Vert \nabla {{\mathcal{L}}}(x)-\nabla {{\mathcal{L}}}(y){\Vert }_{{{\mathcal{G}}}}\le L\Vert x-y{\Vert }_{{{\mathcal{G}}}},\,\forall x,y\in {{{\mathcal{M}}}}_{\zeta }$ on the parameter space ${{\mathcal{G}}}({{{\mathcal{M}}}}_{\zeta })$, with the curvature-dependent constant defined as ${L}_{\zeta }={\sup }_{\zeta > 0}L({{{\mathcal{M}}}}_{\zeta })$. (2) Gradient boundedness: There exists a constant B>0 such that ${\mathbb{E}}[\Vert {\nabla }_{{{\mathcal{A}}}}{{\mathcal{L}}}{\Vert }^{2}]\le {B}^{2}$. Based on these assumptions, Supplementary Theorem 2, Lemma 1, and Lemma 2 are derived.

### Multivariate anomaly detection

#### Topological difference metric

By comparing the geometric deformation discrepancy between the system’s natural topology (${{\mathcal{A}}}$) and the predicted topology (A), anomalous relationships and associated sensor pairs can be identified. First, ${{\mathcal{A}}}$ and A are sparsified and vectorised as follows:

42 $${{{\mathcal{A}}}}_{{ij}}=\left\{\begin{array}{cc}{{{\mathcal{A}}}}_{{ij}} & {if}{{{\mathcal{A}}}}_{{ij}}\le {T}_{{up}}\\ 0 & {otherwise}\end{array}\right.,{A}_{{ij}}=\left\{\begin{array}{cc}{A}_{{ij}} & {if}{A}_{{ij}}\le {T}_{{up}}\\ 0 & {otherwise}\end{array}\right.,$$

where ${T}_{{up}}$ is the topological relationship judgment threshold, employed to dynamically filter those relationships that are beneficial for quantifying topological discrepancies, while also reducing the size and improving the efficiency of the candidate relationship set. Next, the sparsified topological matrices are transformed into vector form as follows:

43 $$\vec{{{\mathcal{A}}}}=	\left[{{{\mathcal{A}}}}_{1,1},{{{\mathcal{A}}}}_{1,2},\cdots,{{{\mathcal{A}}}}_{1,W},{{{\mathcal{A}}}}_{2,1},{{{\mathcal{A}}}}_{2,2},\cdots,{{{\mathcal{A}}}}_{2,W},\cdots {{{\mathcal{A}}}}_{N,1},{{{\mathcal{A}}}}_{N,2},\cdots,{{{\mathcal{A}}}}_{N,W}\right],\\ \vec{A}=	 \left[{A}_{1,1},{A}_{1,2},\cdots,{A}_{1,W},{A}_{2,1},{A}_{2,2},\cdots,{A}_{2,W},\cdots {A}_{N,1},{A}_{N,2},\cdots,{A}_{N,W}\right],$$

Finally, the geometric topological structures for comparison are selected based on the candidate relationships K:

44 $$({C}_{{{\mathcal{A}}}})_{{ij}}=\left\{\begin{array}{cc}1 & {if}{{{\mathcal{A}}}}_{{ij}}\in {{\rm{TopK}}}(\vec{{{\mathcal{A}}}},K)\\ 0 & {otherwise}\end{array}\right.,({C}_{A})_{{ij}}=\left\{\begin{array}{cc}1 & {if}{A}_{{ij}}\in {{\rm{TopK}}}(\vec{A},K)\\ 0 & {otherwise}\end{array}\right.,$$

where under s-relational topological comparison, K=N; under t-relational topological comparison, K=W. Based on the obtained topological relationships and inspired by the Jaccard similarity, a topological discrepancy metric grounded in the Jaccard similarity is devised, defined as follows:

45 $${J}_{T}({C}_{{{\mathcal{A}}}},{C}_{A})=1-\frac{|{C}_{{{\mathcal{A}}}}\cap {C}_{A}|}{|{C}_{{{\mathcal{A}}}}\cup {C}_{A}|},$$

where ${C}_{{{\mathcal{A}}}}$ and ${C}_{{{\bf{A}}}}$ denote the candidate relationship sets of natural and predictive structures after ${{\rm{TopK}}}(\cdot )$ sparsification, respectively. This metric ensures that the resulting anomaly interpretation originates from global perturbations of the system’s endogenous structure, rather than heuristic speculation based on superficial local correlations.

#### Multivariate anomaly spatio-temporal coordinate localization

Multivariate anomaly spatio-temporal coordinate localization is achieved by pinpointing anomalous coordinates through topological deformation. Meanwhile, to mitigate the potential misguidance of model reconstruction performance on anomaly coordinate computation, joint multivariate anomaly spatio-temporal coordinate localization is performed using spatial coordinates (${L}_{{{\boldsymbol{s}}}}$), temporal coordinates (${L}_{{{\boldsymbol{t}}}}$) and reconstructed coordinates (${L}_{{{\boldsymbol{r}}}}$). For a given data block ${S}_{t-W+1}^{t}\in {{{\mathcal{M}}}}^{N\times W}$, its potential fine-grained anomaly label is defined as $L={{{\boldsymbol{0}}}}^{N\times W}$, as detailed below:

46 $${{\boldsymbol{s}}}	=\left\{\lfloor N/{{{\rm{argmax}}}}_{s}|\vec{{{{\mathcal{A}}}}_{s}}-\vec{{A}_{s}}|\rfloor,N\%{{{\rm{argmax}}}}_{s}|\vec{{{{\mathcal{A}}}}_{s}}-\vec{{A}_{s}}|\right\},{L}_{{{\boldsymbol{s}}}}=1,\\ {{\boldsymbol{t}}}	=\left\{{{\lfloor }}W/{{{\rm{argmax}}}}_{t}|\vec{{{{\mathcal{A}}}}_{t}}-\vec{{A}_{t}}|{{\rfloor }},W\%{{{\rm{argmax}}}}_{t}|\vec{{{{\mathcal{A}}}}_{t}}-\vec{{A}_{t}}|\right\},{L}_{{{\boldsymbol{t}}}}=1,\\ {{\boldsymbol{r}}}	=\left\{{{{\rm{argmax}}}}_{s,t}|{S}_{t-W+1}^{t}-{\hat{S}}_{t-W+1}^{t}|\right\},{L}_{{{\boldsymbol{r}}}}=1,\\ L	={L}_{{{\boldsymbol{s}}}}\odot {L}_{{{\boldsymbol{t}}}}+{L}_{{{\boldsymbol{r}}}},$$

where % denotes the modulo operation. ${L}_{{{\boldsymbol{s}}}}$ represents multivariate coupling anomaly labeling: rows correspond to sensor pairs exhibiting anomalies, with the corresponding entry set to 1 when an anomaly occurs. ${L}_{{{\boldsymbol{t}}}}$ denotes multivariate temporal anomaly labeling: columns correspond to time pairs exhibiting anomalies, with the corresponding entry set to 1 upon anomaly occurrence. ${L}_{{{\boldsymbol{r}}}}$ denotes multivariate data reconstruction anomaly labeling: sensors at anomalous timestamps serve as anomalous coordinates, with the corresponding position set to 1 when an anomaly is present.

#### Multivariate anomaly recognition

Multivariate anomaly recognition is performed as follows. First, a sliding window of width W is applied to continuously sample the observational data ${{\bf{X}}}\in {{\mathbb{R}}}^{T\times N}$, generating sequential data blocks ${{\mathcal{X}}}\in {{\mathbb{R}}}^{T\times W\times N}$. Then, anomalies are identified by comparing the observed data ${S}_{t}^{{{{\mathcal{M}}}}_{\zeta }}\subseteq {{\mathcal{X}}}$ (where t≤T) with the predicted data ${\hat{S}}_{t}^{{{\mathcal{M}}}\zeta }$, based on both numerical deviations and topological discrepancies. Using the sparse nature of anomaly distributions, points located within sparsely identified regions are forcibly labeled as anomalous. This step is justified by the fact that anomalous spatio-temporal coordinates obtained via topological discrepancy localization exhibit greater reliability in terms of interpretability. The procedure is detailed below:

47 $${J}_{T}({C}_{{{{\mathcal{A}}}}_{s}},{C}_{{A}_{s}})=\left\{\begin{array}{cc}1,& {if}{J}_{T}({C}_{{{{\mathcal{A}}}}_{s}},{C}_{{A}_{s}}) > {\mu }_{{{\boldsymbol{s}}}}\pm \gamma \cdot {\sigma }_{{{\boldsymbol{s}}}}\\ 0,& {otherwise}\end{array}\right.,$$

where ${\mu }_{{{\boldsymbol{s}}}}$ and ${\sigma }_{{{\boldsymbol{s}}}}$ denote the mean and standard deviation of the coupling topological discrepancy, respectively and γ($0\le \gamma \le 3$) is a hyperparameter that controls the extent of the anomalous region. Finally, the anomaly decision function ${{\bf{A}}}(\cdot )$ is defined as follows:

48 $${{\bf{A}}}(({S}_{t})_{i})=\left\{\begin{array}{cc}1,&{if}{{\rm{Max}}}\bigg(|({S}_{t}^{{{{\mathcal{M}}}}_{\zeta }})_{i}-({\hat{S}}_{t}^{{{{\mathcal{M}}}}_{\zeta }})_{i}|,{J}_{T}\left(\left\{\left({{{\mathcal{A}}}}^{{{{\mathcal{M}}}}_{\zeta,{{\mathcal{D}}}}},{A}_{t}^{{{{\mathcal{M}}}}_{\zeta,{{\mathcal{D}}}}}\right)|{{\mathcal{D}}}{{\mathscr{\in }}}\left\{s,t\right\}\right\}\right)\bigg)\le \tau \\ 0,&{otherwise}\end{array}\right.,$$

where ${{\bf{A}}}(\cdot )$ is a binary anomaly decision function and τ is a predefined decision threshold. Within the function, the maximum of two discrepancy terms is computed: first, the absolute difference between the observed and predicted values of the i-th sensor at the current time step t, given by $\left|({S}_{t}^{{{\mathcal{M}}}\zeta })_{i}-({\hat{S}}_{t}^{{{\mathcal{M}}}\zeta })_{i}\right|$; second, the topological structural discrepancy under multiple views, ${J}_{T}(\cdot )$ which quantifies the difference between the natural structure and the predicted structure. The system’s anomalous state is then determined dynamically via the following expression:

49 $${{\rm{Max}}}\bigg(\left|({S}_{t}^{{{{\mathcal{M}}}}_{\zeta }})_{i}-({\hat{S}}_{t}^{{{{\mathcal{M}}}}_{\zeta }})_{i}\right|,{J}_{T}\left(\left\{\left({{{\mathcal{A}}}}^{{{{\mathcal{M}}}}_{\zeta,{{\mathcal{D}}}}},{A}_{t}^{{{{\mathcal{M}}}}_{\zeta,{{\mathcal{D}}}}}\right){{\rm{| }}}{{\mathcal{D}}}\in \{s,t\}\right\}\right)\bigg).$$

This mechanism adaptively identifies both the sources and locations of anomalies, which arise from either sensor value reconstruction anomalies or topological structural anomalies. When the maximum of these two discrepancy measures does not exceed the threshold τ, the system is classified as normal (output 1); otherwise, it is classified as anomalous (output 0).

## Supplementary information

Supplementary Information

Transparent Peer Review File

## Data Availability

All benchmark datasets utilized in this paper are publicly available online, and their corresponding access URLs are provided below: ASD (https://github.com/zhhlee/InterFusion/tree/main/data/processed), SMAP & MSL, SMD (https://github.com/NetManAIOps/OmniAnomaly/tree/master/ServerMachineDataset), SMAP&WADI (https://itrust.sutd.edu.sg/itrust-labs_datasets/), PSM, MSDS (https://github.com/snedelkoski/multi-source-observability-dataset), GPT, Power System and Gas Pipeline (https://sites.google.com/a/uah.edu/tommy-morris-uah/ics-data-sets), CICIDS (https://www.unb.ca/cic/datasets/ids-2017.html), SKAB, SWAN (https://bitbucket.org/gsudmlab/workspace/projects/FP), GECCO.

## Code availability

The source code of this work can be accessed via the GitHub repository at https://github.com/StructureMan/HAO or the Zenodo repository at https://zenodo.org/records/21018195.
