# Supplementary material for: Hyperbolic adaptive spatial-aware multivariate time series anomaly detection
Source: Nat Commun. 2026 Jul 27;17:9116. doi: 10.1038/s41467-026-75881-1 (PMC13518828; doi:10.1038/s41467-026-75881-1)
Supplement: Supplementary file 1 [file 41467_2026_75881_MOESM1_ESM.pdf]

Hyperbolic Adaptive Spatial-Aware Multivariate Time Series Anomaly Detection

Supplementary Information

Contents

1 Related Work.. 1
1.1 Multivariate Anomaly Detection Methods.. 1
1.2 Graph Structure Discovery and Learning.. 2
1.3 Multivariate Anomaly Cause Interpretation.. 7
1.4 Multivariate Anomaly Detection Evaluation... 9
2 Symbol Definitions.. 10
3 Relevant Theorems and Lemmas.. 11
4 Dataset... 13
5 Experimental Setup.. 17
5.1 Data Preprocessing.. 17
5.2 Dataset Partitioning.. 17
5.3 Evaluation Metrics.. 17
5.4 Small-Sample Hybrid Dynamic Threshold Determination Algorithm(SSA)............. 19
5.5 Parameter Settings.. 19
5.6 Covariate Analysis and Statistical Methods... 20
5.7 Comparison Models... 20
5.8 Complexity Analysis.. 25
6 Confusion Matrix.. 26
7 Comparison Experiments on ROC and P-R Curves.. 26
8 Comparative Experiments on Curvature Variation... 27
9 Case Study.. 27
Supplementary References... 30

1 Related Work

1.1 Multivariate Anomaly Detection Methods

In traditional machine learning-based anomaly detection, two core assumptions are commonly adopted: That anomalous events are low probability characteristics and tend to be located in low density regions of the data distribution. Based on these premises, researchers have developed theoretical frameworks for identifying anomalous data points via probability density estimation or clustering analysis[1][2]. Representative examples include the Isolation Forest(iForest) algorithm[3], which employs a path isolation mechanism; the Local Outlier Factor(LOF) algorithm[4], which relies on local density comparisons; and the DBSCAN algorithm[5], which integrates density-based clustering algorithms. To address the specific characteristics of time series data, extended methods have been proposed, such as the MERLIN algorithm[6], based on dynamic subsequence matching and the MPdist algorithm[7], which employs piecewise distance measurement. However, these conventional methods are highly sensitive to data distribution forms, sample sizes and hyperparameter settings; even minor data perturbations or parameter shifts can lead to significant degradation in detection performance. With the advent of deep learning, data-driven feature representation learning methods have gradually overcome the limitations of manual feature engineering, markedly improving model robustness and generalisation capability.

Early deep learning-based anomaly detection approaches were predominantly framed within supervised learning paradigms, essentially casting anomaly detection as a binary classification problem. Discriminatural boundaries are learned through the labeled positive and negative samples. For instance, DNN-based and CNN-based models[8][9] leverages multi-layer nonlinear transformations or convolutional operations to extract high-dimensional features, dynamically optimizing decision boundary parameters via loss functions and data labels. Autoencoder(AE) and variational autoencoder(VAE) variants strengthen the separability of anomalous samples during feature reconstruction by jointly optimizing reconstruction errors and classification losses[10][11]. Generative Adversarial Networks(GANs)[12] implicitly learn the distribution of normal data through adversarial interplay between generator and discriminator, thereby constructing precise anomaly decision boundaries. Additionally, some researchers incorporate Transformer or RNN architectures to fuse long-range dependencies and historical variation features in data, which enhances model robustness[13][14][15][16]. However, the heavy reliance of such methods on the labelled data restricts their large-scale deployment in industrial scenarios.

At present, multivariate anomaly detection is primarily based on unsupervised learning frameworks, whose core idea is to quantify anomalies by comparing residuals between observed data and model predictions. Examples include the ImDiffusion model[17] based on probabilistic diffusion, time series modelling approaches such as OmniAnomaly and InterFusion[18][19][20] and convolutional time series neural networks like MSCRED[21] and CAE-M[22]. While these methods can effectively detect multivariate anomalies, they lack explicit modelling of anomalous relationships. Consequently, researchers have employed information aggregation mechanisms based on graph neural networks to dynamically model multivariate spatio-temporal coupling relationships; examples include MTAD-GAT[23], GDN[24] and GRN[13]. These approaches learn each sensor's historical behaviours and construct inter-variable coupling relationships via metric learning. Although progress has been made in capturing multivariate spatio-temporal interactions, the deep fusion of spatio-temporal features introduces confounding effects on anomaly provenance. Meanwhile, modelling dynamic topological relationships among variables via information propagation dynamics has been constrained by geometric distortions in the data representation space, limiting the ability to uncover latent couplings or weak anomalies. Moreover, studies on multivariate anomaly causation interpretation, such as GSL-ST[25], TS-GAT[26] and GRN[13], generally rely on weight-driven explanatory mechanisms, which only analyze the anomalous sensors and their immediate neighbours and lack a clear reference benchmark for anomaly interpretation.

Based on the current state of research, studies on dynamic relationship modelling for multivariate anomaly causation remain constrained by core challenges such as the rigidity of preset structures, geometric distortion in data representation and conflation of anomaly interpretation dimensions. The compounding effects of these challenges make it difficult to establish a reliable reference benchmark for multivariate anomaly causation interpretation.

1.2 Graph Structure Discovery and Learning

Addressing the research challenges outlined in Supplementary Section 1.1, the accurate modeling of dynamic relationships from different perspectives of anomaly causation interpretation is central to multivariate anomaly causation interpretation. In the field of Graph Neural Networks(GNNs)[27], this relationship mining task is termed Graph Structure Learning(GSL)[28], whereas in multivariate anomaly detection it is referred to as Graph Structure Discovery (GSD), as shown in Supplementary Figure 1.

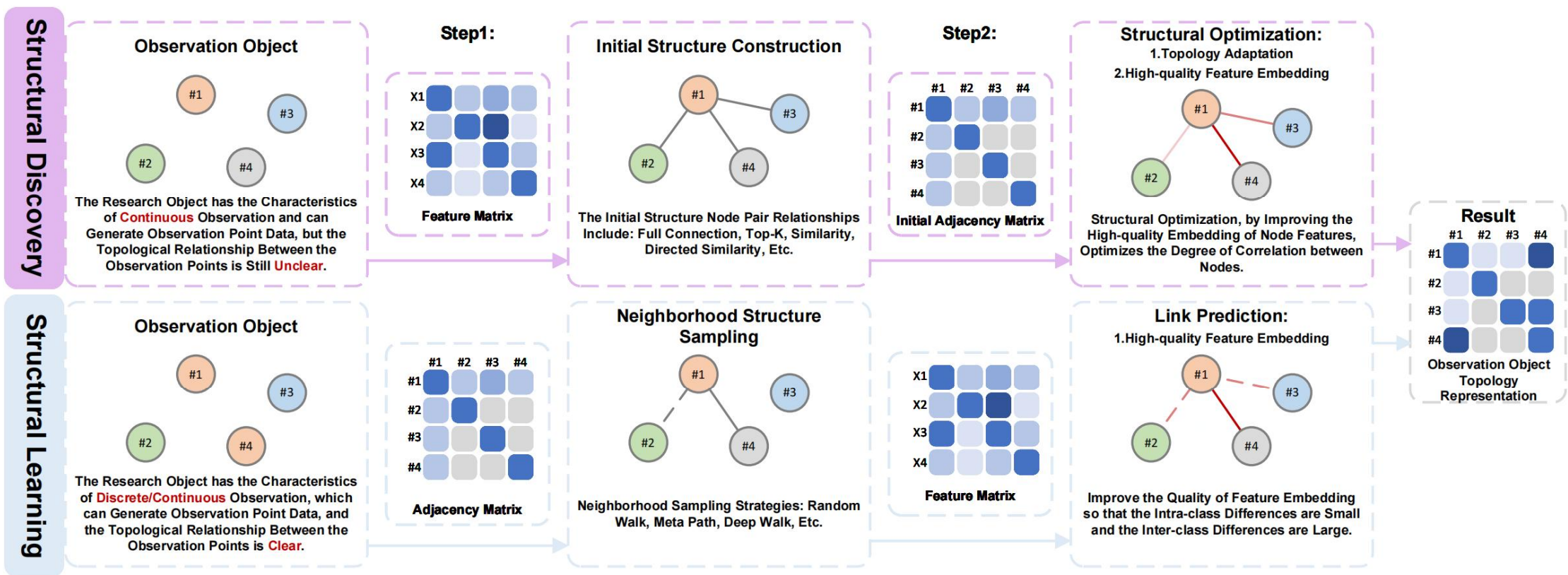

Supplementary Figure 1. Core differences between GSD and GSL. GSD starts from the feature matrix, aiming to construct an initial topology through node correlation measures such as mutual information and dynamically optimizes the adjacency matrix, thus belonging to a data driven structure generation method. In contrast, GSL takes the adjacency matrix as a structural prior and achieves higher-order embeddings through neighbourhood sampling, random walks or attention mechanisms[29], adhering to the logic of structure guided feature representation. At the data adaptation level, GSD must iteratively optimize to approximate the natural data distribution, yet faces generalization bottlenecks due to structural agnosticism. GSL, relying on structural knowability, explicitly fits the distribution of the adjacency matrix to support heterogeneous network modelling. Regarding supervision mechanisms, GSD predominantly operates in an unsupervised manner, for example, using reconstruction loss, whereas GSL often utilizes explicit structural labels or implicit constraints. Fundamentally, the two constitute a cognitive dichotomy between dynamic topology inference and static structure utilization.

1.2.1 Graph Structure Discovery

Graph structure discovery, as a key preprocessing step in multivariate anomaly detection, focuses on resolving latent interaction patterns among variables from high-dimensional time series data. Unlike graph learning methods that rely on known topologies, this task constructs an initial adjacency matrix in a data-driven manner. For example, graph structure discovery methods based on similarity measurement functions, such as GDN[24] and GRN[13], assess the strength of node correlations using cosine similarity. In contrast, DCRNN[30] establishes weight relationships under the principle of distance decay through a parameterized Gaussian kernel function. For graph structure discovery based on composite indicators, STADN[31] constructs a multi-dimensional distance function by integrating correlation coefficients, similarity scores and spatial distance. Regarding graph structure discovery based on symmetry constraints, HST-GNN[32] captures asymmetric interaction relationships by designing a direction-aware function. Similarly, GSL-GL[25] generates a directed adjacency matrix using the directional function and activation function from HST-GNN, effectively addressing the challenge of

modeling unidirectional causal relationships in real-world scenarios. As research into graph structure discovery progresses, dynamic correlation modeling has become a crucial direction. For instance, MUTANT[33] utilizes the Pearson Correlation Coefficient to characterize linear dependencies, while DGINet[34] innovatively introduces a spatio-temporal attention mechanism, achieving time-varying adaptability of correlation strength through learnable parameter matrices.

However, beyond numerous refined relationship modelling approaches, MTAD-GAT[23] adopts an unconstrained strategy for multivariate coupling relationships, presuming that connections exist between all variable pairs and using a fully connected graph as its initial topology. Through message passing and aggregation mechanisms in Graph Neural Networks (e.g., GCN[35], GAT[36]), it dynamically adjusts edge weight coefficients to update topological relationships. Nevertheless, the fully connected paradigm may introduce misleading noisy edge information, impairing the accuracy of subsequent continual learning[37]. To address this challenge, researchers have introduced the $\mathrm{TopK}(\cdot)$ strategy[24][13][31], which selectively retains the K edges with the highest correlation strength to construct a sparse graph structure[38]. However, this operation may overlook potential coupling relationships. To resolve this trade-off, efforts have shifted toward exploring physics-constrained modeling grounded in domain knowledge[39][40], including the exponential decay modeling of temporal dependencies[33] and the design of unidirectional transmission mechanisms for coupling relationships[25]. Nevertheless, existing methods still face several issues: first, the effectiveness of alternatives to the fully connected paradigm lacks rigorous theoretical validation and the mechanisms by which different connection patterns affect the geometric properties of embedding spaces remain unclear. Second, the adaptability of dynamic topology updating mechanisms to natural data characteristics is still not well understood, which can easily lead to structural distortion. Finally, the uncertainty in multivariate coupling relationships constitutes a major theoretical barrier to the lossless construction of natural graph structures, directly limiting the interpretability of anomaly causation analysis in industrial scenarios.

1.2.2 Graph Structure Learning

Graph structure learning aims to automatically infer or optimize graph structural relationships from data, which plays a vital role in understanding the true connections between data points, predicting the evolution of graph structures and uncovering anomalous relationships within data[28]. Current graph structure learning methods can be categorized into four categories: **metric learning-based, optimization-based, generative model-based and unsupervised graph structure learning.**

Metric Learning-based Graph Structure Learning. This category of methods represents the edges in a network as a function of the node representations learned by graph neural networks. Existing GNNs employ a message-passing mechanism, but information propagation is confined to observed edges. The sparsity of the network results in many potentially informative node pairs failing to establish connections[27]. Consequently, AGCN[41] measures the kernel distance via a generalized Mahalanobis distance metric and then treats this kernel distance as a residual which is added to the original graph structure, subsequently updating the Laplacian matrix through GNNs. However, the Laplacian matrix obtained by AGCN through linear transformation tends to be overly dense and lacks a clear optimization objective. PTDNet[38] observes the presence of task-irrelevant noisy edges in the network, which interfere with node representation learning. Therefore, PTDNet directly supervises its structure learning process using the task loss. IDGL[42] demonstrates a mutually reinforcing relationship between graph structure and GNN learning. Based on the hypothesis that nodes with similar low-dimensional representations are more likely to be connected, IDGL proposes an iterative deep graph learning framework, which establishes a clear optimization objective for structure learning. Nevertheless, the graph structure generation method of IDGL, based on node similarity, still faces challenges such as high computational complexity, dense networks and a tendency to fall into local trivial solutions due to the lack of supervision.

Optimization-based Graph Structure Learning. This category of methods treats graph structure learning as the set of parameters indicating whether edges exist between nodes and performs direct parametric learning on these parameters.

ProGNN[43] observes that real-world graph structures exhibit properties such as low rank, sparsity and similarity of features among adjacent nodes and it leverages these properties to guide graph structure learning by directly optimizing the adjacency matrix and adopting an alternating optimization strategy to address the bi-level optimization problem. LDS[44] also follows the bi-level optimization paradigm, but the difference is that its inner level is optimized using the training set, while its outer level uses the validation set. These studies provide useful insights for the subsequent graph structure optimization in this work. However, both these methods rely on the labelled data for supervised learning, which limits their broad applicability in practical scenarios.

Generative model-based methods. GEN[45] notes that most existing graph structure learning approaches focus on exploiting the discriminative properties of graph data (i.e., node connectivity), while ignoring the interpretability of the network generation process. To tackle this issue, GEN adopts the core idea of the classical stochastic block model (SBM), a typical generative model for networks, in which edge formation mainly depends on node community membership: Nodes in the same community are more likely to be connected, while cross community connections are less probable. Based on, GEN proposes a new hypothesis: A given graph structure can be regarded as a random sample generated from some optimal graph structure. Experiments show that the generative modeling strategy can effectively strengthen intra-class connections and weaken inter-class links, thus improving the structural homogeneity of the network.

Unsupervised graph structure learning. Most of the aforementioned methods rely on supervised learning, where structure learning depends on label information. When labels are scarce, only edges near labeled nodes receive effective supervision, while edges far from labeled nodes cannot be learned adequately. To alleviate this issue, STABLE[46] focuses on distance-metric-based graph structure learning and finds that representations learned under supervision are susceptible to perturbations, making it difficult to generate high-quality structures. Consequently, STABLE proposes a two-stage unsupervised approach: first, contrastive learning is adopted for representation learning to obtain reliable embeddings; subsequently, graph refinement is performed by constructing the graph structure based on embedding similarities. SUBLINE[47] treats the learnable views generated by a learnable graph structure module and the original structure as anchor views, maximising mutual information between them to learn the structure and adopts a direct learning strategy that takes the entire graph information as parameters. HolE[48] observes that improving structural homogeneity can enhance clustering performance. It adopts an iterative updating mechanism similar to IDGL[42], alternately performing clustering and structure learning. In each iteration, HolE updates the structure to increase homogeneity according to the clustering results. PROSE[49] notes that existing graph structure learning methods do not account for the varying importance of different regions when learning the graph structure jointly. For example, in social networks, connections between celebrities should be prioritised. PROSE formalises the problem as a top-down partition of the original structure into hierarchical levels with increasing importance and learns the structure bottom-up to ensure that critical substructures are learned first.

In recent years, unsupervised graph structure learning has achieved considerable progress in the inference and optimization of static graph structures. However, when confronted with dynamic graphs, which represent a widely adopted flexible data structure for modeling systems with time-evolving nodes and edges and have been extensively applied in social network analysis, traffic forecasting, e-commerce recommendation and other relevant fields[48], its ability to handle dynamically varying node connections and features remains limited. Consequently, graph representation learning is emerging as a promising new framework for graph structure learning.

1.2.3 Graph Representation Learning

Graph representation learning aims to learn low-dimensional, dense vector representations of graph-structured data[50]. These learned representations allow the topological relationships between nodes to be constructed and measured through vector space operations, such as inner product similarity calculations[51]. This enables efficient simulation of the dynamics and

topological structure of dynamic graphs in Euclidean space. Research on dynamic graphs[52] can broadly be divided into discrete dynamic graph algorithms and continuous dynamic graph algorithms[53].

Discrete dynamic graph methods decompose the dynamic process into a sequence of equally spaced graph snapshots, approximating continuous dynamics through a temporal window of static subgraphs. These methods first encode the graph structure of each snapshot and then aggregate both the structural and temporal effects across snapshots, which use techniques such as matrix perturbation theory[54] Skip-Grams[55] or triadic closure[56]. To capture temporal dependencies between snapshots, Recurrent Neural Networks(RNNs) are widely employed to integrate temporal information into snapshot representations[57]. For example, GraphERT[58] combines a Transformer architecture with temporal random walks to learn both the structural and temporal dynamics of dynamic graph embeddings. However, these approaches share a common limitation: Dynamic information across snapshots is inevitably lost, making it difficult to effectively capture the truly continuous evolution of real-world networks.

Continuous dynamic graph methods take into account the continuous evolution of both nodes and edges. To address the issue of dynamic information loss between snapshots in discrete dynamic graphs, researchers directly model the continuous process of temporal graph evolution[59][60][61][62]. For example, snapshot-based DySAT[60] employs RNNs to aggregate node embeddings from different snapshots. Time encoding-based TGAT[63] extends GAT to dynamic graphs by introducing temporal encodings, endowing the model with the ability to capture temporal information. Random walk-based NeurTWs[64] performs walk sampling and develops a topic embedding method to capture spatio-temporal dynamics among irregularly sampled temporal nodes on continuous dynamic graphs, without relying on explicit temporal encodings. Memory module-based TGN[62] integrates a memory module with a node encoder to update node representations and conduct graph structure learning. Sequential model-based SelfRGNN[61] maps temporal graphs into a general Riemannian space, which utilizes three types of spaces including hyperbolic space to learn temporal evolution relations. Although SelfRGNN involves hyperbolic space, it focuses on latent geometric changes and its topology encoding is still based on Euclidean space.

Although the above studies demonstrate that dynamic graphs can effectively capture spatio-temporal relationships between nodes, they face a dual dilemma in multivariate anomaly causation scenarios: first, uncertainty in prior structures may render existing knowledge ineffective[65]; second, the implicit correlation between feature evolution and structural changes increases the difficulty of interpretation. In addition, current methods rely on Euclidean space for modelling complex topologies, which is prone to geometric distortion.

1.2.4 Hyperbolic Graph Representation Learning

Most existing time series modeling methods adopt Euclidean space embedding, yet their geometric characteristic of uniform expansion pose inherent limitations. The volume of Euclidean space grows polynomially with radius, which cannot match the hierarchical and exponentially expanding inherent structure of real industrial time series systems[66]. This dimension growth mechanism leads to obvious geometric embedding distortion. Especially in dynamic temporal graph scenarios, it easily results in insufficient capture of long-range dependencies, deviation of dynamic evolution features and degradation of sparse structure representation. To address such structural defects, recent studies have introduced hyperbolic geometric space with negative curvature characteristics[53]. Its unique exponential volume expansion mechanism achieves natural geometric isomorphism with hierarchical topological structures of complex systems, providing a more suitable modeling foundation for this task. As shown in Supplementary Figure 2, hyperbolic space follows distinctive geometric expansion rules, and its volume conforms to $V(r) \propto e^{\zeta r}$, where ζ denotes the curvature parameter. The expansion intensity of the space can be adaptively adjusted by tuning the curvature value. It can be clearly observed from Supplementary Figure 2 that data points are densely distributed in Euclidean space with weakly distinguishable relational features, whereby numerous sparse, weak and abnormal structural connections are concealed by dense clusters[67]. In contrast, as the absolute value of

curvature in hyperbolic space increases, the geometric distances between samples expand evidently, and the distinguishability of hierarchical structures as well as subtle abnormal connections is greatly improved. From the perspective of metric geometric distortion theory, hyperbolic embedding is capable of fitting complex hierarchical structures with low distortion. Existing theoretical studies have proven that embedding finite δ-hyperbolic structures into hyperbolic space $\mathcal{H}^d$ yields multiplicative distortion bounded by $O(\delta \log n)$ [68][69]. When embedding identical hierarchical structures into Euclidean space, however, the lower bound of distortion increases polynomially with the number of nodes as $\Omega(n^{1/d})$. This demonstrates that Euclidean embedding inevitably distorts the inherent hierarchical distances of networks and artificially compresses the spatial distances of edge nodes and sparse structures. Consequently, numerous sparse abnormal nodes are crowded into dense normal clusters, making subtle anomalies unnoticeable and undetectable. In comparison, hyperbolic space effectively preserves hierarchical distance differences and mitigates structural embedding distortion, which theoretically accounts for the phenomenon that structural distinguishability is significantly enhanced with the increase in absolute curvature values shown in Supplementary Figure 2(a-c).

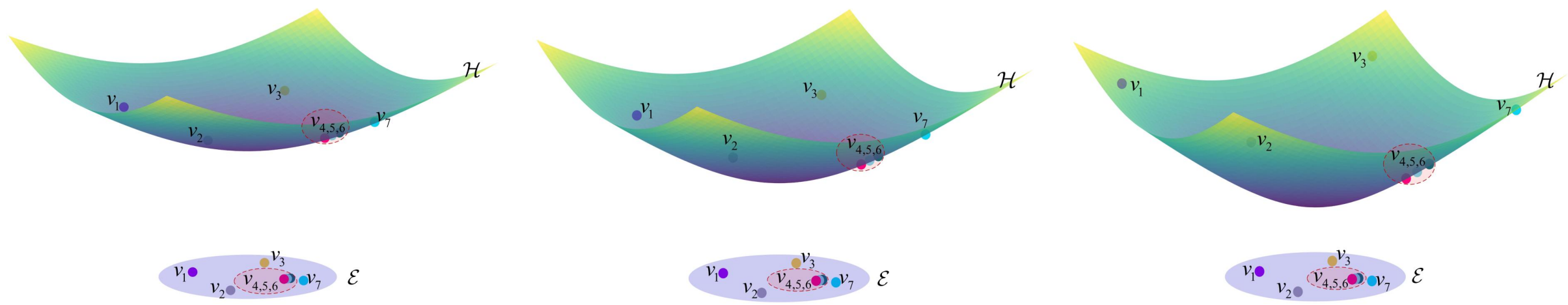

(a) $K=-1$ (b) $K=-2$ (c) $K=-4$

Supplementary Figure 2. Embedding representations under varying hyperbolic curvatures (K=-1, -2, -4). In Euclidean space, the relationships among data points (e.g., v_4, v_5, v_6) exhibit weak discriminability while displaying strong clustering, or community structure[70]. However, after being mapped to hyperbolic space, as the absolute value of the curvature increases, corresponding sequentially to subfigure (a) $K=-1$, (b) $K=-2$ and (c) $K=-4$, the geometric distances between these data points expand significantly. This indicates a systematic improvement in the discriminability of their natural relationships. Such enhanced differentiation of key relationships, especially anomalous relationships, not only facilitates more effective recognition of subtle anomaly types, but also provides finer and more robust evidence for uncovering the causes of unknown anomaly types.

Complex dynamic industrial networks generally exhibit tree-like hierarchical characteristics[68][69][71]. Given a branching factor b, the number of nodes at depth r grows exponentially as b^r. Constrained by its polynomial volume growth nature, Euclidean space fails to match such exponential structural expansion. It can only avoid node overlapping by continuously increasing embedding dimensions, which inevitably leads to severe dimensionality curse[72]. By virtue of its inherent exponential expansion property, hyperbolic space can accommodate tree-like hierarchical structures with arbitrary depths in low-dimensional spaces when the curvature parameter satisfies $b \approx e^{\zeta}$ [68]. This property enables the full retention of dynamic evolution details and sparse abnormal structures within multivariate time series data. More importantly, the curvature-aware gradient mechanism of hyperbolic space can accurately activate the representation learning of sparse abnormal nodes. Different from the isotropic and invariant gradient update rule in Euclidean space, hyperbolic Riemannian manifolds dynamically reshape the optimization landscape via metric tensors[69]. Within the tangent space $\mathcal{T}_x\mathcal{H}^{d,\zeta}$, Riemannian gradients adaptively scale the parameter updating magnitude according to spatial curvature[73]. For feature points located at spatial boundaries, i.e., hierarchical leaf nodes and sparse abnormal samples, the metric tensor automatically amplifies the gradient norm, which effectively alleviates the prevalent gradient vanishing problem in deep temporal graph models[74]. This mechanism enables the model to finely tune and reinforce the learning of weak anomalous patterns and sparse abnormal correlations, and remarkably improves the detection performance for subtle and concealed anomalies, which is fully consistent with the visualized distribution rules in Supplementary Figure 2(a-c).

Driven by these theoretical advantages, hyperbolic representation learning has developed rapidly and been widely adopted. Initially used for modeling complex networks, hyperbolic space has five isometric models: the hyperboloid model (also known as the Lorentz model), the Klein model, the Gans model, the Poincaré ball model, and the hemisphere model. Among these, the Poincaré ball model has been widely adopted due to its numerical stability[68]. Building upon these foundations, hyperbolic graph representation learning has achieved remarkable success. For instance, HGNN(Hyperbolic Graph Neural Network)[68] demonstrates superior effectiveness over traditional Euclidean models in graph classification tasks. HGAT[74] further proposes a graph convolution method incorporating hyperbolic attention mechanisms, which enables information propagation via gyrovector operations and applies it to practical scenarios such as node classification and clustering. HGCN[69] focuses on local structures, designing a local aggregation scheme that performs hyperbolic graph embedding within local tangent spaces and introducing learnable curvature parameters to adapt to diverse data characteristics.

Hyperbolic graph representation learning offers two important insights. First, when the natural structural properties of a system are unknown, performing node embedding in Euclidean space may introduce geometric distortion, making it difficult to uncover latent, subtle anomalous relationships. Second, the inherent geometric representation capacity of Riemannian manifolds suggests that relationship modeling can transcend the boundaries of traditional data-driven approaches, moving toward a more sophisticated and profound modeling paradigm based on data geometry, which exhibits greater potential than conventional dynamic graph relationship mining.

1.3 Multivariate Anomaly Cause Interpretation

Multivariate anomaly causation interpretation, as shown in Supplementary Figure 3, aims to ensure both the independence and reliability of explanatory dimensions: for a given analytical perspective, it must accurately characterize the causal associations within that perspective (e.g., feature importance or temporal dependency) while systematically decoupling interference from other explanatory dimensions (e.g., spatial correlations or group effects). Although inspired by dynamic graph representation learning, most studies improve the embedding quality of spatio-temporal features and pursue optimal representations of spatio-temporal relationships in multivariate data through joint representation learning[75][76][77]. Some researchers have instead constructed and learned these relationships separately, as exemplified by MTAD-GAT[23]. However, existing work lacks deep investigation into anomaly interpretation. Specifically, there is an absence of reference benchmarks for anomaly interpretation[78][79]. This section therefore focuses on analyzing relevant studies with potential for anomaly interpretation, particularly on constructing interpretability for temporal dependencies and multivariate coupling relationships.

Temporal dependency relationships describe the interactions between the operating states of a system at different time points. For example, literature [25] assumes that temporal dependencies follow an exponential decay distribution $e^{-\lambda t}$, while literature [31] presumes that they adhere to a strength-first principle. The construction of these multivariate temporal dependency relationships is typically based on statically predefined structures, combined with graph convolutional networks or attention mechanisms to update the strength of multivariate temporal dependencies. Literature [34] attempts to dynamically update temporal dependencies by integrating temporal features of multivariate data via Long Short-Term Memory(LSTM) networks, thereby achieving edge weight updates for temporal dependency relationships.

The reliability of multivariate anomaly causation interpretation is highly correlated with the rationality of the initially preset structure. Unreasonable presuppositions impose excessively rigid constraints on the natural structure-for instance, whether temporal dependencies are bidirectional or unidirectional and whether the exponential decay property truly reflects reality. As shown in Supplementary Figure 4, the exponential decay characteristic of temporal dependencies is only valid under short-term sampling windows. Meanwhile, the prevalence of community and hierarchical structures in multivariate coupling, as shown in Supplementary Figure 5, represents novel properties that motivate further investigation.

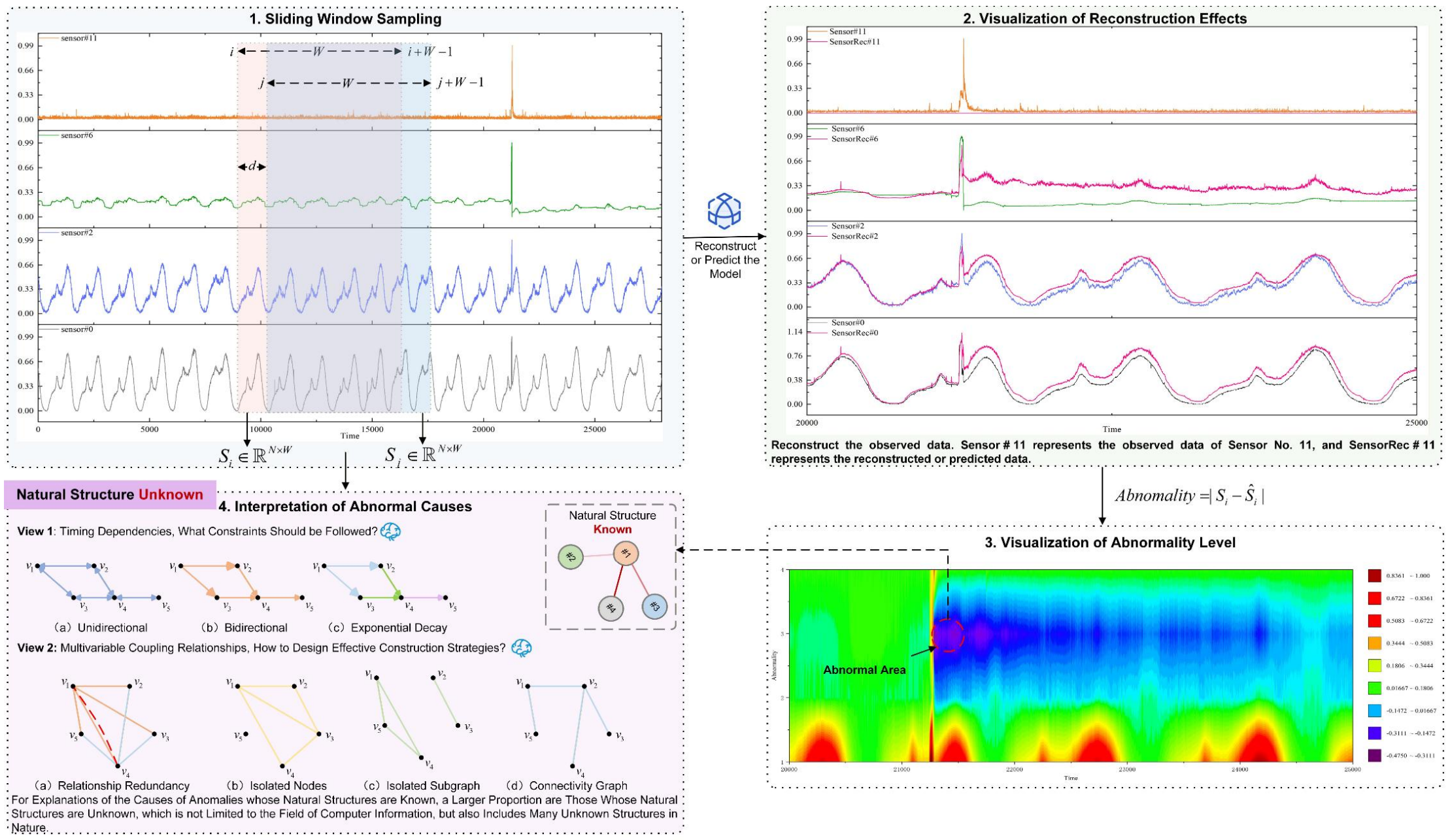

Supplementary Figure 3. Multivariate Anomaly Causation Interpretation Workflow[13][24][23][31]**.** Data preprocessing stage: The sliding window (W) technique is adopted to perform sliding sampling on monitoring data, where the sampling interval (d) is selected to ensure the continuity of data processing. Sampling is continuous when $d = 1$ and discrete when $d > 1$. **1. Model prediction stage:** A reconstruction-prediction model is used to reconstruct or predict current data based on historical operational patterns. **2. Anomaly detection and localization stage:** Predicted data are compared with actual observations to compute anomaly scores. Particular emphasis is placed on positions exhibiting strong positive or negative correlations within anomalous regions, enabling precise anomaly localization. **3. Anomaly causation interpretation stage:** Based on the spatial and temporal coordinates of the anomaly, the underlying causes are investigated in detail. Currently, anomaly causation interpretation can be divided into two categories according to whether the true physical topology is known or unknown, with the unknown topology case dominating real-world applications. However, research on reliable reference benchmarks for anomaly interpretation remains insufficient, which highlights the urgency and significance of this study.

Coupling relationships describe the mutual interactions between different variables. Compared with temporal dependency relationships, coupling relationships generally exhibit weaker directional characteristics. For example, literature [25][32] uses a directed similarity function to determine the directionality of coupling relationships. Literature [23] adopts a fully connected dense adjacency matrix to capture potential coupling relationships among variables, while literature [24][31][33] apply a $\mathrm{TopK}(\cdot)$ strategy to select the most strongly coupled variable pairs and construct a sparse adjacency matrix to capture these potential relationships. Although the fully connected approach may increase model complexity, it allows the model to explore and identify subtle yet influential patterns that may exist among sensors.

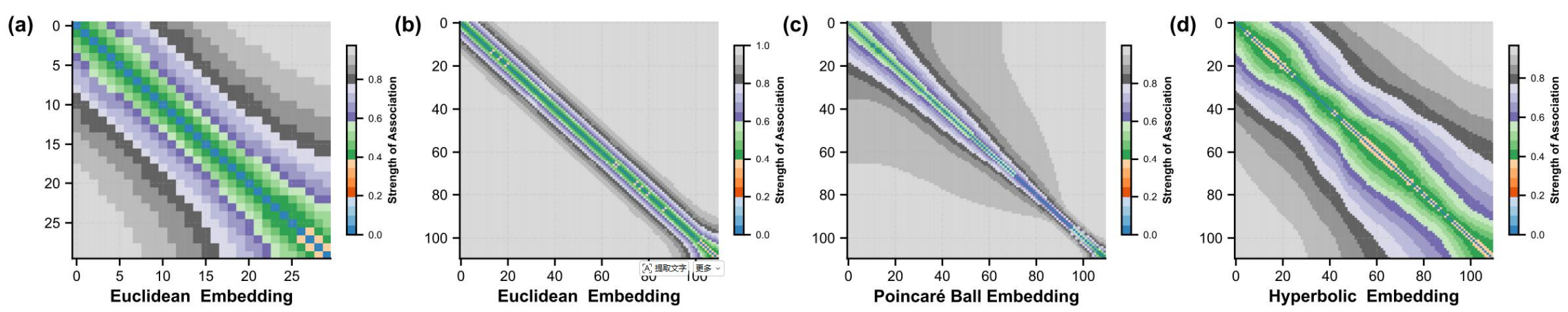

Supplementary Figure 4. Impact of observation window and spatial representation on temporal relationship parsing. Using the SMAP dataset[20] as the research object, we investigate the dual constraints imposed by the observation window

scale and spatial representation method on the parsing capability of temporal relationships. Specifically, as shown as **a**, **b** compare the parsing performance under Euclidean space embedding for medium-scale (30) and large-scale (110) observation windows, while as shown in **c**, **d** contrast the parsing differences between Poincaré ball embedding and hyperbolic space embedding under the large-scale (110) window. In Euclidean space, the large-scale observation window incorporates more data and covers a broader representation range, resulting in significantly better parsing performance than the medium-scale window. Under the large-scale window, non-Euclidean space embeddings, such as the Poincaré ball and hyperbolic space, can effectively capture the nonlinear and hierarchical complex associations in temporal data, demonstrating far higher parsing adaptability than Euclidean space. There exists a notable compatibility between the observation window scale and spatial representation method; only through their synergistic optimization can temporal relationships be parsed efficiently. Medium-scale windows are well-suited to Euclidean space embeddings, whereas large-scale windows require pairing with non-Euclidean embedding approaches.

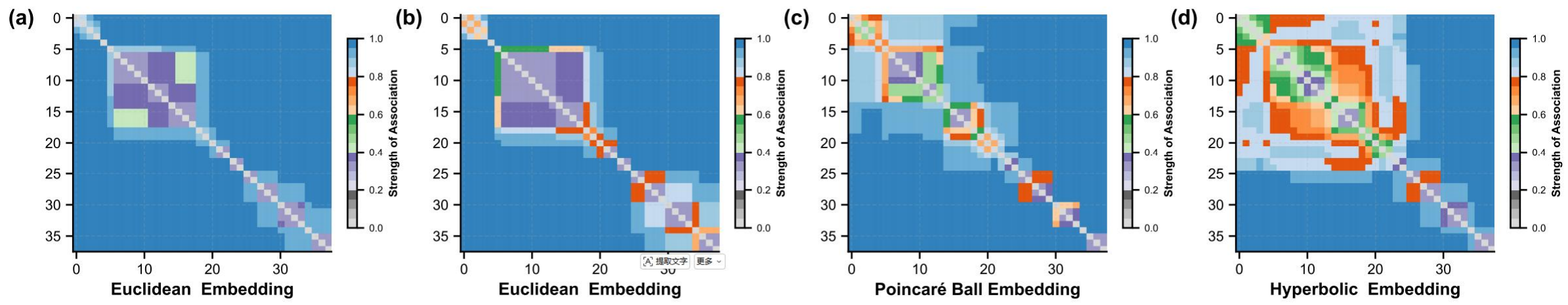

Supplementary Figure 5. Impact of observation windows and spatial embeddings on the analysis of sensor coupling relationships. Using the SWaT dataset as an example, we illustrate the dual constraints imposed by observation window size and spatial representation method on the ability to resolve sensor coupling relationships. Supplementary Figure 5(a) shows the result under Euclidean space embedding with an observation window of 5, which captures only local, short-term coupling associations between sensors. Supplementary Figure 5(b) shows the result under the same Euclidean embedding but with an extended observation window of 110, enabling integration of long-term temporal interactions and revealing global, long-range coupling patterns. Under a fixed observation window of 110, Supplementary Figure 5(c) shows the outcome of Poincaré ball space embedding, which accommodates the hierarchical structure of sensor coupling relationships and clearly distinguishes coupling associations between core and peripheral nodes. Supplementary Figure 5(d) shows the result of hyperbolic space embedding, which further enhances the representation of nonlinear, non-Euclidean coupling relations, allowing more accurate capture of weak and long-distance couplings between sensors. The spatial embedding distribution better matches the actual topology of the coupling network, confirming that hyperbolic space embedding offers superior adaptability over traditional Euclidean embedding for resolving complex sensor coupling relationships.

1.4 Multivariate Anomaly Detection Evaluation

Probability distribution-based threshold determination algorithms: The Peaks Over Threshold (POT) method based on Extreme Value Theory[81] can effectively estimate the occurrence probability of anomalous events without requiring assumptions about the underlying data distribution, even when dealing with highly complex raw data. For example, literature[32] adopts a parameterized Generalized Pareto Distribution (GPD) to fit the tail of the extreme value probability distribution for dynamic threshold selection τ, defined as follows:

$$\tau \simeq \tau_0 \frac{\hat{\beta}}{\hat{\gamma}} \cdot ((\frac{q \cdot h}{l})^{-\hat{\gamma}} - 1), \tag{1}$$

where $\hat{\beta}$ and $\hat{\gamma}$ are the estimates of β and γ obtained via the maximum likelihood method, q denotes the expected probability that the anomaly score is below the threshold, s_c is the lower quantile of the GPD and h is the length of the anomaly score.

$$F(s_c) = P(\tau_0 - s_t | s_t > \tau_0) \sim (1 + \frac{\gamma \cdot s}{\beta})^{-\frac{1}{\gamma}}, \tag{2}$$

where s_t represents the anomaly score at a given time and τ_0 is the initially set anomaly score threshold. β and γ are the shape and scale parameters of the GPD, respectively. Although the POT algorithm performs well in terms of accuracy, it is relatively complex in parameter setting and has certain limitations in terms of generality.

Numerical aggregation-based threshold determination algorithms[13][21][24]: These methods determine anomaly decision thresholds through numerical aggregation. Compared with POT-based algorithms, this approach can identify thresholds that achieve an optimal performance balance under fewer parameter constraints and it is defined as follows:

$$\tau_t = \max_i s_i\,(t), \tag{3}$$

where the overall anomaly value at time t is denoted as τ_t, which is obtained by aggregating the sensor anomalies s_i using the maximum function. This method can effectively reduce the interference of hyperparameters on model accuracy; however, its robustness decreases when facing data concept drift.

Based on the summary of related work, data geometry-driven modelling is considered to be an important direction for future multivariate anomaly causation interpretation. This direction differs from dynamic graph relationship modelling that relies on historical prior topological knowledge and also from existing dynamic evolution analysis that fuses multivariate spatio-temporal features in Euclidean space. The aim of this work is to uncover the system's natural, invariant topological properties, which constitute its natural structure. This natural structure reflects the endogenous relationships of the system under normal operating conditions and serves as a key reference benchmark for understanding the causes of anomalies.

2 Symbol Definitions

To enhance the readability and comprehensibility of this paper, the notation and component abbreviations used throughout the text are standardized. The specific conventions are summarized in Supplementary Table 1.

Supplementary Table 1. Notation Definitions.

No.	Symbol	Meaning
1	$\mathcal{E}$	Euclidean space.
2	$\mathcal{P}$	Poincaré ball space (a type of hyperbolic space).
3	$\mathcal{H}$	Hyperbolic space.
4	s	Multivariate coupled correlation perspective.
5	t	Multivariate temporal dependency perspective.
6	τ	Multivariate anomaly detection threshold; τ_{best} denotes the optimal threshold.
7	$\mathbf{X} \in \mathbb{R}^{T\times N}$	Multivariate time series data matrix, where T is the observation duration and N is the number of sensors or monitoring points.
8	$\mathcal{X} \in \mathbb{R}^{T\times W\times N}$	Multivariate data representation sampled using a sliding window (of length W).
9	$\{S_i^{\mathcal{M}_\zeta}\}_{i=t-W}^{t-1}$	Processed data sequence within the time window $[t-W, t-1]$, represented in the universal manifold space $\mathcal{M}_\zeta$.
10	$\mathcal{M} \in \{\mathcal{E},\mathcal{P},\mathcal{H}\}$	Universal manifold space representation (covering Euclidean, Poincaré ball, and hyperbolic spaces).
11	$\mathbf{W}^{\mathcal{M}_\zeta}, \boldsymbol{x}^{\mathcal{M}_\zeta}$	Matrix and vector representations in the universal manifold space $\mathcal{M}_\zeta$.
12	$\mathcal{D} \in \{s,t\}$	Multivariate integrated perspective representation (s for coupled correlation, t for temporal dependency).
13	$K = -\dfrac{1}{\zeta^2}$	Curvature of the universal manifold space $\mathcal{M}_\zeta$.
14	$\mathcal{T}_x\mathcal{M}, \mathcal{M} \in \{\mathcal{E},\mathcal{P},\mathcal{H}\}$	Tangent space of the universal manifold space $\mathcal{M}$ at point $\boldsymbol{x}$.
15	$g_x^{\mathcal{M}}, \mathcal{M} \in \{\mathcal{E},\mathcal{P},\mathcal{H}\}$	Riemannian metric of the universal manifold space $\mathcal{M}$ at point $\boldsymbol{x}$.
16	$\mathcal{M}_\zeta^n, \mathcal{M} \in \{\mathcal{E},\mathcal{P},\mathcal{H}\}$	A specifically instantiated n-dimensional manifold space with curvature $K = -1/\zeta^2$.
17	$d^{\mathcal{M}_\zeta}(\boldsymbol{x},\boldsymbol{y}), \mathcal{M} \in \{\mathcal{E},\mathcal{P},\mathcal{H}\}$	Distance between points $\boldsymbol{x}$ and $\boldsymbol{y}$ in the manifold space $\mathcal{M}_\zeta$.
18	$\log_{\boldsymbol{x}}^{\mathcal{M}_\zeta}(\boldsymbol{y}), \mathcal{M} \in \{\mathcal{E},\mathcal{P},\mathcal{H}\}$	The logarithmic map at base point $\boldsymbol{x}$, mapping point $\boldsymbol{y}$ on the manifold space $\mathcal{M}_\zeta$ to the tangent space $\mathcal{T}_x\mathcal{M}$.
19	$\exp_{\boldsymbol{x}}^{\mathcal{M}_\zeta}(\boldsymbol{v}), \mathcal{M} \in \{\mathcal{E},\mathcal{P},\mathcal{H}\}$	The exponential map at base point $\boldsymbol{x}$, mapping vector $\boldsymbol{v}$ in the tangent space $\mathcal{T}_x\mathcal{M}$ back to the manifold space $\mathcal{M}_\zeta$.
20	$P_{\boldsymbol{x}\to\boldsymbol{y}}^{\mathcal{M}_\zeta}(\boldsymbol{v})$	Vector transport in the manifold space $\mathcal{M}_\zeta$, moving vector $\boldsymbol{v}$ from point $\boldsymbol{x}$ to point $\boldsymbol{y}$.
21	$\oplus^\zeta, \otimes^\zeta$	Generalized addition and multiplication operations in the manifold space $\mathcal{M}_\zeta$.

22	$\sigma^{\otimes^{\mathcal{M}_{\zeta_{l-1},\zeta_l}}}$	Non-linear activation function in the manifold space $\mathcal{M}_{\zeta}$, where l denotes the model layer number (connecting layers with curvatures ζ_{l-1} and ζ_l respectively).
23	$\mathcal{A}^{\mathcal{M}_{\zeta,\mathcal{D}}} \in \{s,t\}, \mathcal{M} \in \{\mathcal{E},\mathcal{P},\mathcal{H}\}$	Natural structures identified under the multivariate integrated perspective $\mathcal{D} \in \{s,t\}$ in the manifold space $\mathcal{M}_{\zeta}$. For simplicity, when the manifold space is fixed, it is also denoted as $\mathcal{A}_{\mathcal{D}}$.
24	$A_t^{\mathcal{M}_{\zeta,\mathcal{D}}}, \mathcal{D} \in \{s,t\}, \mathcal{M} \in \{\mathcal{E},\mathcal{P},\mathcal{H}\}$	Local structure of the multivariate integrated perspective D in the manifold space $\mathcal{M}_{\zeta}$ at time t.
25	$\widetilde{\mathcal{A}}_{rc}^{\mathcal{M}_{\zeta,\mathcal{D}}}, \mathcal{D} \in \{s,t\}, \mathcal{M} \in \{\mathcal{E},\mathcal{P},\mathcal{H}\}$	Anomaly root cause relationships identified under the multivariate integrated perspective D in the manifold space $\mathcal{M}_{\zeta}$.
26	$G_t^{\mathcal{M}_{\zeta,\mathcal{D}}}(V_t^{\mathcal{M}_{\zeta,\mathcal{D}}}, E_t^{\mathcal{M}_{\zeta,\mathcal{D}}}, S_t^{\mathcal{M}_{\zeta,\mathcal{D}}})$, $\mathcal{D} \in \{s,t\}, \mathcal{M} \in \{\mathcal{E},\mathcal{P},\mathcal{H}\}$	Graph representation of the local structure under the multivariate integrated perspective D in the manifold space $\mathcal{M}_{\zeta}$ at time t, consisting of vertex set V_t, edge set E_t and node features S_t.
27	$M^{\mathcal{M}_{\zeta,\mathcal{D}}} \in \{s,t\}, \mathcal{M} \in \{\mathcal{E},\mathcal{P},\mathcal{H}\}$	Physical constraint matrix under the multivariate integrated perspective D in the manifold space $\mathcal{M}_{\zeta}$, used to suppress illegal connections.
28	$\mathcal{D} - \mathrm{HDNN}(\cdot), \mathcal{D} \in \{s,t\}$	Multiview linear transformation layer under the universal manifold space.
29	$\mathcal{D} - \mathrm{HGCN}(\cdot), \mathcal{D} \in \{s,t\}$	Multiview attention graph convolution layer under the universal manifold space.
30	$\mathrm{HSD}(\cdot)$	Multiview harmonic structure difference calculation under the universal manifold space.

3 Relevant Theorems and Lemmas

Theorem 1 (Isometric Isomorphism): Let $\mathcal{M}$ be a d-dimensional smooth connected Riemannian manifold equipped with a pseudo-Riemannian metric g of signature ν. Then, for any point $\boldsymbol{x} \in \mathcal{M}$, there exists an open neighbourhood $U \subset \mathcal{M}$ and a smooth map $\phi: U \to \mathbb{R}^{d,\nu}$ such that the differential of ϕ at $\boldsymbol{x}$, $\mathrm{d}\boldsymbol{\phi}_{\boldsymbol{x}}: \mathcal{T}_{\boldsymbol{x}}\mathcal{M} \to \mathcal{T}_{\phi(\boldsymbol{x})}\mathbb{R}^{d,\nu}$ is an isometric linear isomorphism.

Proof: First, by the Cartan–Dieudonné theorem[82], for any point $\boldsymbol{x} \in \mathcal{M}$, there exists a local coordinate system $(U; x^1, \ldots, x^d)$ such that the metric tensor g can be diagonalised into its standard form at $\boldsymbol{x}$:

$$g_{\boldsymbol{x}} = \mathrm{diag}(\underbrace{+1, \ldots, +1}_{\nu}, \underbrace{-1, \ldots, -1}_{d-\nu}), \tag{4}$$

where the number of negative signs ν matches the metric signature of the target pseudo-Euclidean space $\mathbb{R}^{d,\nu}$. Next, the mapping $\phi: U \to \mathbb{R}^{d,\nu}$ is constructed, with $\phi(x^1, \ldots, x^d) = (x^1, \ldots, x^d) \in \mathbb{R}^{d,\nu}$. Its differential at $\boldsymbol{x}$, $\mathrm{d}\boldsymbol{\phi}_{\boldsymbol{x}}$, maps the tangent vector $\boldsymbol{v} = v^i \partial_i|_{\boldsymbol{x}} \in \mathcal{T}_{\boldsymbol{x}}\mathcal{M}$ to $\mathrm{d}\boldsymbol{\phi}_{\boldsymbol{x}}(\boldsymbol{v}) = (v^1, \ldots, v^d) \in \mathcal{T}_{\phi(\boldsymbol{x})}\mathbb{R}^{d,\nu}$. Finally, the preservation of the pseudo-Riemannian inner product is verified. For any $\boldsymbol{u}, \boldsymbol{v} \in \mathcal{T}_{\boldsymbol{x}}\mathcal{M}$, the following holds:

$$g_{\mathcal{M}}(\boldsymbol{u}, \boldsymbol{v}) = \sum_{i=1}^{\nu} u^i v^i - \sum_{j=\nu+1}^{d} u^j v^j = \eta(\mathrm{d}\boldsymbol{\phi}_{\boldsymbol{x}}(\boldsymbol{u}), \mathrm{d}\boldsymbol{\phi}_{\boldsymbol{x}}(\boldsymbol{v})), \tag{5}$$

where $\eta = \mathrm{diag}(+1^{\nu}, -1^{d-\nu})$ is the standard metric of $\mathbb{R}^{d,\nu}$, indicating that if $\mathrm{d}\boldsymbol{\phi}_{\boldsymbol{x}}$ is an isometric linear map, then the Jacobian matrix of ϕ, $J_{\phi} = \partial x^i / \partial x^j$ is identically the $d \times d$ identity matrix over U, so that $\det(J_{\phi}) \equiv 1 \neq 0$. By the inverse function theorem, ϕ is a local diffeomorphism in some neighbourhood of $\boldsymbol{x}$. In summary, ϕ realises a local isometric embedding from $\mathcal{M}$ into $\mathbb{R}^{d,\nu}$.

Theorem 2 (Convergence of Geometric Parameters $\boldsymbol{\Theta}$): With the step size chosen as $\eta_{\Theta} = \frac{1}{L_{\Theta}\sqrt{T}}$, after T iterations it holds that:

$$\frac{1}{T}\sum_{k=1}^{T} \| \nabla_{\Theta}\mathcal{L}^{(k)} \|^2 \leq \frac{2L_{\Theta}(\mathcal{L}^{(0)} - \mathcal{L}^*)}{\sqrt{T}} + \frac{B^2}{\sqrt{T}}, \tag{6}$$

where $\mathcal{L}^*$ denotes the global optimum.

Brief Proof: By constructing a Lyapunov function[83] $V_k = \|\Theta^{(k)} - \Theta^*\|^2$ and applying the law of cosines in Riemannian geometry:

$$V_{k+1} \leq V_k - 2\eta_{\Theta}\langle \nabla\mathcal{L}, \Theta^{(k)} - \Theta^* \rangle + \eta_{\Theta}^2 L_{\Theta}^2 V_k, \tag{7}$$

the result follows directly by combining this with Jensen's inequality.

Theorem 3 (Lindeberg–Lévy Central Limit Theorem[84]): Let $\xi_1, \xi_2, \cdots, \xi_n, \cdots$ be a sequence of independent and identically distributed (i.i.d.) random variables satisfying $E\xi_k = \mu, D\xi_k = \sigma^2$. Define the standardized sum as $\zeta_n = \frac{1}{\sigma\sqrt{n}}\sum_{k=1}^{n}(\xi_k - \mu)$. If $0 < \sigma^2 < \infty$, the limiting distribution of ζ_n satisfies:

$$\lim_{n\to\infty} P\{\zeta_n < x\} = \frac{1}{\sqrt{2\pi}}\int_{-\infty}^{x} e^{-\frac{t^2}{2}} dt, \tag{8}$$

this theorem ensures the asymptotic normality of multivariate time series reconstruction errors in iterative model optimization and verifies that such errors converge stably in line with Gaussian distribution. It provides an essential theoretical prerequisite for stable hyperbolic embedding parameter updating and steady iterative optimization of spatio-temporal topological structures.

Theorem 4 (Bernoulli's Law of Large Numbers[84]): Let μ_n denote the occurrence frequency of event A within n independent Bernoulli trials and p represent the occurrence probability of event A in a single trial. For any $\varepsilon > 0$, the following formula holds:

$$\lim_{n\to\infty} P\left\{\left|\frac{\mu_n}{n} - p\right| < \varepsilon\right\} = 1, \tag{9}$$

the law of large numbers reveals the statistical stability of event frequency in repeated trials. In this work, it provides theoretical support for sliding window sampling of multivariate time series and statistical estimation of anomaly probability. It explains that continuous window sampling enables the model to capture stable statistical features of spatio-temporal structures and acquire reliable anomaly probability estimation, and effectively eliminates interference brought by random errors from single-window sampling.

Theorem 5 (Gärtner–Ellis inequality[84]): Let $\{\xi_i\}$ be a sequence of independent random variables with $D\xi_i = \sigma_i^2 < \infty, (i = 1,2,\cdots,\infty)$ and $\{C_n\}$ be a non-increasing positive constant sequence. For any positive integers m, n satisfying $m < n$ and arbitrary $\varepsilon > 0$, we have:

$$P\left\{\max_{m\le j\le n}\left|\sum_{i=1}^{j}(\xi_i - E\xi_i)\right| \ge \varepsilon\right\} \le \frac{1}{\varepsilon^2}\left(C_m^2\sum_{j=1}^{m}\sigma_j^2 + \sum_{j=m+1}^{n} C_j^2\sigma_j^2\right), \tag{10}$$

this theorem constrains the range of cumulative errors generated in hyperbolic embedding updating and incremental topology iteration. It strictly proves that the parameter updating error of the adaptive curvature module achieves bounded convergence during iterative training, which provides core theoretical guarantee for the convergence and stability of the entire adaptive hyperbolic learning framework.

Lemma 1 (Geometrically Preserving Message Aggregation): For any representation space $\mathcal{M}_\zeta$, given a node feature set $\{x_i\}_{i=1}^{n} \subset \mathcal{M}_\zeta$, the message aggregation operator $\Phi_{\mathcal{M}_\zeta}: \mathcal{M}_\zeta^n \to \mathcal{M}_\zeta$ must satisfy:

$$d^{\mathcal{M}_\zeta}\left(\Phi_{\mathcal{M}_\zeta}(\{x_i\}), \Phi_{\mathcal{M}_\zeta}(\{y_i\})\right) \le \sum_{i=1}^{n} w_i\, d^{\mathcal{M}_\zeta}(x_i, y_i), \tag{11}$$

where w_i denotes the weight coefficient and $d^{\mathcal{M}_\zeta}(\cdot,\cdot)$ is the geodesic distance in the $\mathcal{M}_\zeta$ space. In Euclidean space, this condition can be satisfied by a linearly weighted average, which admits a closed-form solution:

$$\Phi_{\mathcal{E}}(\{x_i\}) = \sum_{i=1}^{n} w_i x_i \quad \left(w_i \ge 0, \sum w_i = 1\right). \tag{12}$$

However, in the hyperbolic space $\mathcal{H}$, the negative curvature of the manifold causes geodesics to diverge exponentially and thus the weighted averaging operation no longer admits a closed-form solution.

Lemma 2 (Curvature Update Stability): When the curvature update step size satisfies $\eta_\zeta < \frac{2}{\kappa_{\max}}$ (where $\kappa_{\max}$ denotes the maximum sectional curvature), the sequence $\{\zeta^{(k)}\}$ fulfills:

$$\|\zeta^{(k+1)} - \zeta^*\| \le \rho\|\zeta^{(k)} - \zeta^*\|, \quad \rho = 1 - \frac{\eta_\zeta \lambda_{\min}}{2}, \tag{13}$$

where $\lambda_{\min}$ is the minimum eigenvalue of the Hessian matrix $\nabla^2 \mathcal{L}(\zeta^*)$.

4 Dataset

The experimental section of this study utilizes 16 publicly available datasets (Supplementary Table 2), covering various domains such as server cluster failures[18], Mars rover malfunctions[20] and natural gas pipeline intrusions[85]. Considering the diversity of source scenarios for these datasets, anomaly types are broadly categorized into point anomalies, regional anomalies and sequential anomalies[86]. To facilitate the shows of how these anomalies manifest in univariate (Supplementary Figure 6) and multivariate (Supplementary Figure 7) settings, as well as the challenges involved in their recognition, detailed schematic examples are provided. Additionally, Supplementary Table 2 records the types of anomalies contained in each dataset.

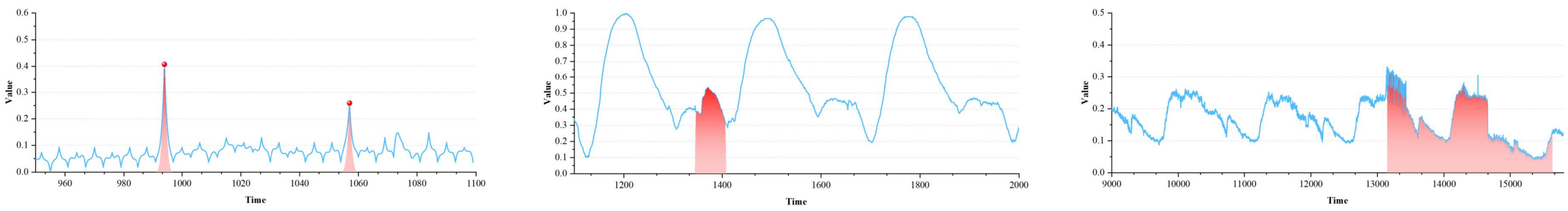

(a) Univariate Point-level Anomaly (b) Univariate Regional Anomal (c) Univariate Sequence Anomaly

Supplementary Figure 6. Univariate Anomaly Types. (a) Univariate point anomaly refers to a monitoring value at a specific time point *t* that significantly deviates from those at other time points. Such anomalies can be subdivided into those above or below the average level, but the core characteristic is a difference from the average. In pattern sequences, anomalous points often appear as extreme values. Sometimes, these outliers may be noise. Since anomalies can affect analytical results, they must be detected and removed. However, in some cases, they may also represent critical events, such as cyberattacks or sensor malfunctions. **(b)** Univariate regional anomaly refers to sensor behavior within a time interval Δt that markedly differs from historical trends. This includes trend-type anomalies (e.g., Bias, Drift within the region), characterized by a preserved trend but an overall shift away from the standard level; and more challenging anomalies (e.g., Erratic, Stuck, Spike within the region), marked by sharp fluctuations or sustained constant values. These anomalies are typically associated with gradual equipment degradation and failure or changes in application service patterns. **(c)** Univariate sequence anomaly denotes a deviation of the data pattern from historical patterns starting at time point *t*. Its manifestations resemble the harder-to-handle anomalies within regions (e.g., Erratic, Stuck, Spike), but pose higher risks because they may lead to long-term shifts in service patterns, rendering detection models based on historical priors ineffective.

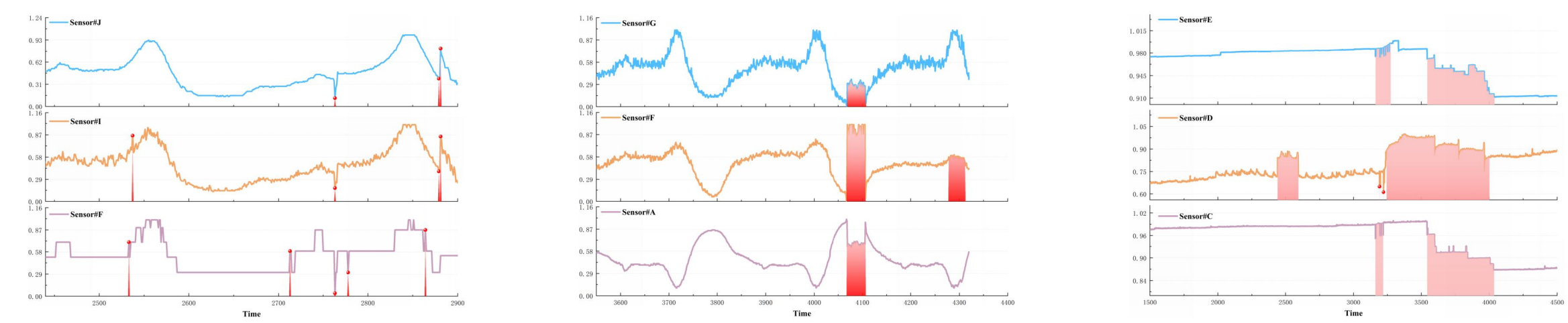

(a) Multivariate Point-level Anomaly (b) Multivariate Regional Anomaly (c) Multivariate Sequence Anomaly

Supplementary Figure 7. Three types of multivariate time series anomalies. Unlike univariate anomalies, the core of multivariate anomalies lies in the combined behavior of multiple variables that collectively reflecting the overall health status of the system. This makes the analysis more complex, requiring researchers to go beyond focusing solely on the historical patterns of individual variables and instead to consider both the interdependencies and mutual influences among variables. **(a)** In multivariate point anomalies, the key to judgment is whether multiple monitoring variables simultaneously exhibit significant deviations at a specific time point *t*. Interactions or competitive relationships among variables may cause

weaker point anomalies to be masked or overlooked, even though such subtle point anomalies can sometimes be manifestations of representative events, for example, the transient points are in the GECCO dataset[87], where water quality indicators are affected by instantaneous or periodic additions of purification agents. Moreover, when a single sensor shows multiple anomalous points while others exhibit few, the model must precisely distinguish true anomalies from spurious ones, which poses a serious challenge to anomaly detection systems. **(b)** Multivariate regional anomalies are defined as significant deviations of system behavior from normal operating patterns within a time window Δt. For multivariate data with clear periodicity, anomalies, which includes complex ones such as Erratic, Stuck and Spike, can be effectively detected through historical pattern modeling, data reconstruction and discrepancy analysis. However, the complexity of multivariate data arises from its temporal dependencies, inter-variable coupling correlations and the spatio-temporal dynamic evolution of these relationships, all of which hinder precise monitoring of such anomalies. Taking Sensor *F* shown in **(b)** as an example, its first anomaly showed a similar sequence deviation pattern to other sensors, whereas the second exhibited a markedly different pattern. This inconsistency makes it difficult to determine whether the anomaly stems from a change in service mode or from equipment degradation. Such difficulties in identifying the root cause of anomalies constitute a common challenge that existing research must overcome. **(c)** Multivariate sequence anomalies are considered one of the greatest challenges in the field of multivariate anomaly monitoring. The absence of periodic characteristics in the temporal dependency relationships of multivariate data makes it very difficult to extract regular patterns and detect anomalous deviations accordingly. In addition, the occurrence of Erratic phenomena in multivariate sequence data disrupts the continuous dynamic evolution of inter-variable coupling correlations. Meanwhile, the locally adaptive nature of spatio-temporal dynamic relationships often fails to capture such long-term and subtle drifts in anomalous patterns. More importantly, point anomalies or regional anomalies occurring on a single sensor can also significantly impact the stability of anomaly monitoring models, thereby further increasing the difficulty of anomaly detection.

Supplementary Table 2 Dataset Overview. This table quantifies the complexity of anomaly detection tasks using the Difficulty index, where higher values indicate greater detection difficulty.

No.	Dataset	Dataset Background Description	Statistical Features					Anomaly Types			*D*
			Train	Test	*D*	*A*	*E*	*P*	*R*	*S*	
1	ASD[18]	The Application Server Dataset (ASD) is a service cluster dataset collected by a team from Tsinghua University from a large internet company. The dataset contains 45 days of observation data (uniformly distributed at 5-minute intervals), consisting of 30 days of normal operation data and 15 days of data containing anomalies. It includes 19 metrics describing server status, covering key aspects such as CPU, memory, network and virtual machines.	102331	51840	19	0.0461	12	√	√	-	3
2	MSL[20]	Mars Science Laboratory (MSL) is a dataset provided by NASA, comprising sensor and actuator data from the Mars rover itself.	58317	73729	55	0.1048	27	√	-	√	2
3	SMAP[20]	Soil Moisture Active Passive (SMAP) is a dataset provided by NASA, containing soil sample data and remote sensing information collected by the Mars rover.	140825	444035	25	0.1285	55	√	√	-	2
4	SMD[19]	The Server Machine Dataset (SMD) is operational data of a computer cluster collected by a team from Tsinghua University from a large internet company. The dataset records the continuous resource utilization of a compute cluster composed of 28 servers over a period of five weeks.	708405	708420	38	0.0415	28	√	-	-	1
5	SWaT[80]	The Secure Water Treatment (SWaT) system is a water treatment operational testbed. Its data collection lasted for 11 consecutive days, during which the system operated around the clock. During the final four days of the collection process in 2016, a total of 36 attacks were recorded. Attack points included sensors (e.g., water level sensors, flow meters, etc.) and actuators (e.g., valves, pumps, etc.).	496800	449919	51	0.2344	1	√	√	-	1
6	PSM[88]	The Pooling Server Metrics (PSM) dataset originates from the eBay e-commerce platform	14990	14990	26	0.0795	1	√	√	√	2

		and collects internal data from multiple application server nodes. The dataset consists of 13 weeks of normal operation data and 8 weeks of data containing anomalies.									
7	MSDS[89]	The Multi-Source Distributed System (MSDS) is a complex distributed system testbed developed by Technische Universität Berlin. Machine metrics are collected from physical nodes using Glances, including indicators such as CPU, memory and load. Application logs are aggregated via OpenStack services, consolidating logs from all services running on the physical nodes. Distributed tracing captures information such as HTTP requests, service latency, item associations and the duration of different segments of service requests (including RPC and database layer processing times) to identify workflows.	29286	29286	10	0.7225	1	√	-	√	3
8	WADI[90]	The Water Distribution (WADI) dataset records a continuous 16-day operation process, of which 14 days are in normal operation and 2 days are in attack scenario states. The data collection covers all network traffic, sensor readings and actuator statuses.	1048571	172801	123	0.0599	1	√	√	-	3
9	Synthetic[21]	The Synthetic dataset is a synthetic data generation method proposed by researchers at the University of Notre Dame to simulate the operational conditions of real-world power plants. This method selects sine or cosine functions based on random seeds and incorporates time delays, frequency variations and random Gaussian noise to generate time series. Using this approach, 30 time series (each containing 20,000 data points) are generated and 5 shock-like anomalies with durations of 30, 60 and 90 time units are randomly injected, respectively.	10000	10000	30	0.048	1	√	-	-	1
10	GPT[85]	The Gas Pipeline Testbed (GPT) is a simulated typical SCADA test system created by Gao Wei et al., comprising components such as MTUs, RTUs and HMIs. This system controls air pumps, pressure sensors, relief valves and electromagnetic relief valves, with pipeline pressures ranging from 0 – 20 PSI and a maximum allowable pressure of 22 PSI. The system operates in three modes: low-pressure mode (~0.1 PSI), medium-pressure mode (~10 PSI, range 9 – 11 PSI) and high-pressure mode (~20 PSI, range 18 – 22 PSI). Pressures exceeding 22 PSI or transitions between operating modes are considered anomalous.	14119	14119	26	0.0118	1	-	√	-	3
11	Power System[91]	The Power System Datasets were created through collaboration between Udam Adhikari and colleagues and Raymond Boggs and Justin Beaver from Oak Ridge National Laboratory (ORNL). These datasets contain measurement data from electric power transmission systems under normal, disturbance, control and cyber-attack conditions. The data types include synchrophasor measurements, as well as log information from the Snort intrusion detection system, simulated control panels and relays.	1282	5224	127	0.6609	1	√	-	√	3
12	Gas Pipeline[92]	The Gas Pipeline Datasets were developed through a collaboration between Justin Beaver and Raymond Boggs at Oak Ridge National Laboratory (ORNL). In this effort, the team from Michigan State University (MSU) was responsible for providing the raw data logs, while the ORNL team handled the formatting of these logs into usable datasets.	37706	48510	26	0.5165	1	-	√	-	3
13	CICIDS[93]	CICIDS 2017 (Canadian Institute for Cybersecurity Intrusion Detection System 2017) is a key network intrusion detection system (IDS) research dataset released by the Canadian Institute for Cybersecurity (CIC). Data collection spanned five working days (July 3 – 7, 2017). Monday served as a normal traffic day, while Tuesday through Friday involved the injection of various attacks, including FTP/SSH brute force, DoS, Heartbleed, web attacks, port scanning, botnets and DDoS. Attacks were	97718	191033	78	0.0103	1	√	√	√	3

		carried out in both the morning and afternoon periods on Tuesday through Friday.									
14	SKAB[94]	The Skoltech Anomaly Benchmark (SKAB) is an anomaly detection benchmark designed for industrial scenarios, aimed at evaluating the performance of single-point (outlier) and collective (change point) anomaly detection algorithms. SKAB comprises sensor data from 35 independent simulated industrial devices (such as motors and water pumps). These multivariate time series data are acquired from sensors on a testbed, including industrial metrics such as timestamps, motor current and post-circulation pressure of water pumps.	9401	1146	8	0.3517	1	√	√	-	3
15	SWAN[95]	The SWAN dataset was created by Georgia State University in Atlanta, USA and is derived from photospheric vector magnetograms in the Space-weather HMI Active Region Patches (SHARP) series. It compiles multivariate time series observational data from 4,098 solar active regions between May 2010 and December 2018 and records 51 predictive parameters.	60000	60000	38	0.326	1	√	-	√	3
16	GECCO[87]	The GECCO dataset was provided by the SPOTeven laboratory during the 2018 Industrial Challenge. It consists of measurement data from key nodes in the water supply system of the Thuringian Remote Water Supply Company, which are used for water quality monitoring, such as the outlets of water treatment plants and the inlets and outlets of water towers. The data record water quality and operational parameters every minute. Any change in water quality indicators (chlorine dioxide content, pH value, oxidation-reduction potential, conductivity, turbidity) is considered an event. Changes in operational data (water flow rate, water temperature) may be associated with water quality changes but do not constitute events on their own.	69260	69260	9	0.0105	1	√	-	√	3
Statistical metrics include Dimension (*D*), Anomaly Rate (*A*), and Entity (*E*); anomaly types cover Point (*P*), Regional (*R*), and Sequential (*S*); *D* denotes Difficulty Level.											

Currently, the main challenges in multivariate anomaly detection stem from the complexity of multivariate data in dynamic scenarios. To enhance detection performance, models need to more deeply integrate spatio-temporal joint features of multivariate data and accurately characterize their coupling relationships, with the aim of improving data reconstruction capability and achieving more effective anomaly discrimination. However, existing studies still present several issues worthy of deep consideration: First, although spatio-temporal feature fusion significantly strengthens the model's representational capacity for multivariate data, it also introduces confusion in the explanatory dimensions of anomaly causes. Second, the characterization of multivariate spatio-temporal coupling relationships largely relies on prior structural knowledge and employs graph representation learning methods, which include the hyperbolic representation learning discussed in this work, to mine spatio-temporal dynamics[70]. For example, literature [78] uses the output of topology estimation (NTP) in grid Energy Management Systems (EMS) as a reference topology. Yet, such reliance on known prior structures notably diminishes the model's generalization ability in dynamic or unknown scenarios. Furthermore, the core focus of this study is the analysis of anomaly causes, an aspect that is notably absent from existing research. To tackle these problems, this study proposes a unified joint learning framework that integrates data reconstruction, natural structure discovery and geometric parameter adjustment, which aims to effectively address the aforementioned challenges.

5 Experimental Setup

5.1 Data Preprocessing

To enhance the model's ability to capture latent patterns in the data and ensure the stability of the training process, this study sequentially performs data sampling and normalization operations. To preserve the complete contextual information around the moment an anomaly occurs, a sliding window strategy is adopted to sample the observed data. Specifically, the original

observation data $X \in \mathbb{R}^{N \times T}$ is continuously sampled by sliding a window of size W, resulting in a dataset $\mathcal{X} \in \mathbb{R}^{T \times N \times W}$ that contains the anomaly context at each time step, with each data element $S_i \in \mathcal{X}$ serving as the basic unit for the model to learn anomaly patterns. In addition, a data normalization method is introduced to eliminate scale differences among different features in the raw data. This operation not only accelerates model convergence but also enables direct comparison of coupling relationships and sparsity characteristics between different features, thereby simplifying subsequent anomaly analysis procedures. The definition is as follows:

$$\mathcal{X}_{\text{norm}} = \frac{\mathcal{X} - \mathcal{X}_{\text{min}}}{\mathcal{X}_{\text{max}} - \mathcal{X}_{\text{min}}}, \tag{14}$$

where $\mathcal{X}_{\text{norm}}$ denotes the normalized result, $\mathcal{X}$ is the original data, $\mathcal{X}_{\text{min}}$ is the minimum value of $\mathcal{X}$ and $\mathcal{X}_{\text{max}}$ is the maximum value of $\mathcal{X}$.

5.2 Dataset Partitioning

As shown in Supplementary Table 2 lists the datasets used for model training. The training dataset contains monitoring data of the observation targets under normal operating modes, while the test dataset covers scenarios with unknown system operating states, including both normal and anomalous mode data. The test and validation sets are constructed using a specific partitioning method, with the data partition ratio denoted as $\beta = 90\%$ and the anomaly ratio in the original test data denoted as γ. The procedure is as follows:

(1) Initial Data Splitting: The normal and anomalous data from the original test dataset are each split according to the ratio β. Both normal and anomalous data are divided into 10% and 90% portions, respectively.

(2) Test Set Construction: From the 10% portion of the normal data and the 10% portion of the anomalous data, samples are drawn according to the original test dataset's normal-to-anomalous ratio (i.e., γ) to form a new test set. For example, if the original test dataset has a normal-to-anomalous sample ratio of 1000:10, then the new test set will contain 100 samples from the 10% normal portion and 1 sample from the 10% anomalous portion (or scaled accordingly).

(3) Validation Set Construction: The remaining 90% portion of the normal data and the 90% portion of the anomalous data are combined and samples are similarly drawn according to the original test dataset's normal-to-anomalous ratio (i.e., γ) to form a new validation set.

5.3 Evaluation Metrics

Anomaly recognition refers to evaluating the model's performance in identifying anomalous points or segments. The assessment metrics for this task are defined based on the confusion matrix, as shown in Supplementary Table 3, which illustrates the correspondence between model predictions and ground-truth labels[43]. Multivariate time series anomaly detection suffers from severe class imbalance, which tends to overestimate the overall model performance and fails to reflect practical anomaly detection capability. To mitigate evaluation bias caused by imbalanced data distribution, this study adopts the inverse class frequency weighting strategy to optimize weighted Precision(Pre), Recall(Rec) and F1-score[24][96], defined as follows:

$$\text{Pre}_w = \frac{\sum_{i \in \text{TP}} s_i}{\sum_{i \in \text{TP}} s_i + \sum_{i \in \text{FP}} s_i}, \text{Rec}_w = \frac{\sum_{i \in \text{TP}} s_i}{\sum_{i \in \text{TP}} s_i + \sum_{i \in \text{FN}} s_i}, \text{F1}{-}\text{score}_w = 2 \cdot \frac{\text{Pre}_w \cdot \text{Rec}_w}{\text{Pre}_w + \text{Rec}_w}, \tag{15}$$

where s_i denotes the inverse frequency weight assigned to each sample. Let N_0 be the number of normal samples, N_1 be the number of anomalous samples, and the total sample size is $N = N_0 + N_1$. Specifically, normal samples with larger proportion are assigned low weight $s_i = w_1 = N_1/N$, while sparse anomalous samples are assigned high weight $s_i = w_0 = N_0/N$ to balance their evaluation importance.

Furthermore, evaluation results derived from fixed thresholds are highly sensitive to threshold selection. To address this issue, we adopt Volume Under the Surface of ROC (VUS_ROC) for comprehensive model evaluation[97]. Given a

monotonically increasing threshold sequence $Th = [Th_0, Th_1, ..., Th_N]$ satisfying $0 = Th_0 < Th_1 < ... < Th_N = 1$, and a buffer length sequence $\mathcal{L} = [\ell_0, \ell_1, ..., \ell_L]$ satisfying $0 = \ell_0 < \ell_1 < ... < \ell_L = \ell$, the VUS_ROC is defined as follows:

$$\text{VUS\_ROC} = \frac{1}{4}\sum_{w=1}^{L}\sum_{k=1}^{N} \Delta^{(k,w)} \cdot \Delta^{w}, \tag{16}$$

where $\Delta^{(k,w)} = \Delta^{k}_{TPR_{\ell_w}} \cdot \Delta^{k}_{FPR_{\ell_w}} + \Delta^{k}_{TPR_{\ell_{w-1}}} \cdot \Delta^{k}_{FPR_{\ell_{w-1}}}, \Delta^{k}_{FPR_{\ell_w}} = FPR_{\ell_w}(Th_k) - FPR_{\ell_w}(Th_{k-1}), \Delta^{k}_{TPR_{\ell_w}} = TPR_{\ell_w}(Th_{k-1}) + TPR_{\ell_w}(Th_k), \Delta^{w} = |\ell_w - \ell_{w-1}|$.

Anomaly Diagnosis is a finer-grained detection task than anomaly recognition, aimed at determining the specific sensors responsible for the anomaly[23]. The hit rate is selected to quantify the model's anomaly diagnosis performance[14], defined as follows:

$$\text{HitRate@}K = \frac{1}{N}\sum_{i=1}^{N} \mathbb{I}\,(i, K), \tag{17}$$

where N denotes the number of test samples and $\mathbb{I}(i, K)$ is an indicator function. For the i-th test sample, $\mathbb{I}(i, K) = 1$ if the ground-truth anomalous sensor is among the $\text{TopK}(\cdot)$ anomalous sensors predicted by the model; otherwise, $\mathbb{I}(i, K) = 0$.

In addition, the normalized discounted cumulative gain (NDCG) is adopted to evaluate the quality of the model-predicted list of anomalous sensors[14], defined as follows:

$$\text{NDCG@}K = \frac{\text{DCG@}K}{\text{IDCG@}K}, \quad \text{DCG@}K = \sum_{i=1}^{K} \frac{2^{rel_i} - 1}{\log_2(i+1)}, \quad \text{IDCG@}K = \sum_{i=1}^{|REL|} \frac{2^{rel_i} - 1}{\log_2(i+1)}, \tag{18}$$

where DCG measures the cumulative gain of the $\text{TopK}(\cdot)$ elements in the ranked results, which incorporates a positional discount. rel_i denotes the anomaly score of the i-th element and $\log_2(i+1)$ serves as the positional discount factor, which penalizes results that appear later in the ranking. IDCG is the DCG value obtained from the ideal ranking of all ground-truth anomalies, i.e., with the true anomalies placed at the top positions. $|REL|$ indicates the number of ground-truth anomalies. A larger NDCG value signifies higher quality of the model-predicted anomalous list.

Anomaly Cause Interpretation extends anomaly diagnosis by aiming to identify the underlying causes of anomalies. Traditional methods $\text{TopK}(\mathcal{N}_i)$ tend to explain anomaly causes based on the contribution of the sensor set $\mathcal{N}_i$ associated with the anomalous sensor i[13][24][96]. In contrast, the proposed method explains anomaly causes by contrasting the structural variation differences between the natural structure and the predicted structure of multivariate data and visualizing these differences, rather than relying on conventional local influence analysis. The anomalous structural relationship is defined as follows:

$$\widetilde{\mathcal{A}}^{\mathcal{M}_{\zeta,\mathcal{D}}}_{rc} = 1\{k \in \text{TopK}(\text{vec}(\mathcal{A}^{\mathcal{M}_{\zeta,\mathcal{D}}}), K)\}, r = k/\sqrt{|\text{vec}(\mathcal{A}^{\mathcal{M}_{\zeta,\mathcal{D}}})|}, c = k\%\sqrt{|\text{vec}(\mathcal{A}^{\mathcal{M}_{\zeta,\mathcal{D}}})|}, \tag{19}$$

where $\text{vec}(\cdot)$ denotes the operation of serializing structural information, $|\cdot|$ returns the number of elements and r, c are the row and column indices of $\text{TopK}(\text{vec}(\mathcal{A}^{\mathcal{M}_{\zeta,\mathcal{D}}}), K)$. $\text{TopK}(\text{vec}(\mathcal{A}^{\mathcal{M}_{\zeta,\mathcal{D}}}), K)$ extracts globally high-correlation sensor relationships. If the serialized position index k corresponds to a candidate sensor, then $\widetilde{\mathcal{A}}^{\mathcal{M}_{\zeta,\mathcal{D}}}_{rc} = 1$; otherwise, $\widetilde{\mathcal{A}}^{\mathcal{M}_{\zeta,\mathcal{D}}}_{rc} = 0$. Next, view position information is generated for each sensor, defined as follows:

$$(x, y)_i = \left(\cos\left(\frac{i}{2\pi\sqrt{|\text{vec}(\mathcal{A}^{\mathcal{M}_{\zeta,\mathcal{D}}})|}}\right), \sin\left(\frac{i}{2\pi\sqrt{|\text{vec}(\mathcal{A}^{\mathcal{M}_{\zeta,\mathcal{D}}})|}}\right)\right), i \in \{0, 1, \cdots, |\mathcal{M}_{\zeta,\mathcal{D}}|\}. \tag{20}$$

5.4 Small-Sample Hybrid Dynamic Threshold Determination Algorithm(SSA)

The SSA is designed based on the dataset partitioning strategy proposed in this study, which integrates structural difference metrics to achieve efficient and robust anomaly evaluation. First, the error between the predicted behavior and the observed behavior of sensor i at time t is taken as the anomaly score, which is defined as follows:

$$\mathrm{Err}_i(t) = |(S_t^{\mathcal{M}_\zeta})_i - (\hat{S}_t^{\mathcal{M}_\zeta})_i|. \tag{21}$$

Next, considering that excessive deviation of any sensor's error value relative to other sensors may impair the accuracy of anomaly detection[24], a robust normalization method, which is more resilient to outliers, is applied to process the error value of each sensor. The definition is as follows:

$$a_i(t) = \frac{\mathrm{Err}_i(t) - \tilde{\mu}_i}{\tilde{\sigma}_i}, \tag{22}$$

where $\tilde{\mu}_i$ and $\tilde{\sigma}_i$ denote the median and the interquartile range of IQR^2, respectively. Next, to compute the global anomaly value at time t, the multi-view discrepancy values are aggregated using a structure-enhanced maximum function, defined as follows:

$$\mathrm{A}(t) = \mathrm{Max}\left(a_i(t), J_T\left(\left\{\left(\mathcal{A}^{\mathcal{M}_{\zeta,\mathcal{D}}}, A_t^{\mathcal{M}_{\zeta,\mathcal{D}}}\right) | \mathcal{D} \in \{s,t\}\right\}\right)\right). \tag{23}$$

Finally, a mixed sampling strategy for positive and negative samples from the training data is adopted to thoroughly explore the numerical characteristics embedded in historical anomaly data, thereby enhancing the robustness of anomaly detection. The determination of the optimal threshold τ_{best} for SSA is defined as follows:

$$\tau_{best} = \underset{\tau}{\mathrm{argmax}} \sum_{i=1}^{N-1} \frac{\mathrm{FPR}_t - \mathrm{FPR}_{t+1}}{2} \times (\mathrm{TPR}_t + \mathrm{TPR}_{t+1}), \tag{24}$$

where N denotes the number of anomalous values in the set $\{\mathrm{A}(t)\}$. The false positive rate $\mathrm{FPR} = \left\{\frac{FP}{TN+FP} | \tau_t\right\}$ and the true positive rate $\mathrm{TPR} = \left\{\frac{TP}{TP+FN} | \tau_t\right\}$ are both computed based on $\tau_t \in \{\mathrm{A}(t)\}$. Finally, the value of τ_t corresponding to the maximum of $\sum_{i=1}^{N-1} \frac{\mathrm{FPR}_t - \mathrm{FPR}_{t+1}}{2} \times (\mathrm{TPR}_t + \mathrm{TPR}_{t+1})$ is selected as the optimal threshold τ_{best} for multivariate anomaly detection.

Furthermore, considering the substantial performance impact of data scale N (under varying sample partitioning ratios) on the search process for τ_{best}, whose original time complexity is $O(N)$, a dynamic search strategy is designed: first, sort the anomaly value sequence $\{\mathbf{A}(t)\}$; second, adaptively adjust the search path for τ_{best} based on the direction of the evaluation metric F1-score's change gradient, whether it is increasing or decreasing. This strategy reduces the time complexity to $O(\log N)$, achieving a significant improvement in search efficiency.

5.5 Parameter Settings

Model Training Optimizer: The AdamW optimize[98] is used for model training.
Learning Rate Adjustment: A step scheduler is adopted to adjust the learning rate, with an initial learning rate of 0.01 and a step size of 5[99].
Multi-level Signal Parsing Attention Mechanism: The default number of constant neurons is set to 32. When the reconstructed signal contains sparse level signals, specifically, when the number of transition signals in the observed data is ≤ 3, additional layers of signal parsing should be added and the number of neurons should be adjusted to 64. This configuration effectively addresses scenarios involving the reconstruction of highly sparse signals.
Experimental Environment: All model training and experiments were conducted on a system configured as follows: an Intel Xeon(R) Platinum 8470Q processor, 110 GB RAM, an NVIDIA RTX PRO 6000 GPU and the Ubuntu 22.04 operating system. All models were trained using the PyTorch-2.0.0+cu117 library[100].

5.6 Covariate Analysis and Statistical Methods

All covariates that may influence model performance and experimental outcomes were systematically tested, controlled, and reported in this study. These covariates are categorized into three groups: data-level covariates, model-level covariates, and experimental design covariates.

Data-level covariates included the sliding window size ($W \in \{5,15,30,70,90,110\}$), multivariate dimensionality (sensor count, ranging from 8 to 127), anomaly difficulty level (low/ medium/ high), anomaly type (point, regional, sequential), and sequence length. Min–max normalization was applied to all features, as detailed in Section Dataset. The sliding window size was varied for sensitivity analysis but fixed to an optimal value for the main experiments.

Model-level covariates comprised three embedding spaces (Euclidean, Poincaré ball, Hyperbolic), the adaptive curvature parameter ($K = -1/\zeta^2$) in Hyperbolic space, hidden dimension, learning rate, AdamW optimizer with step scheduling, core model components (HDNN, MSCD, AHGSD, SSA, TS, SS), and anomaly scoring algorithms (STS, POT, TTS, NSTS). Hyperparameters were tuned via grid search and fixed across all datasets to ensure fair comparison. Additionally, ablation studies were conducted by systematically removing individual components to quantify their contributions.

Experimental design covariates included the validation set ratio (ranging from 0.1 to 0.9), label condition (original labels vs. corrected labels), evaluation metrics (F1-score, Precision, Recall, AUC, AP, VUS_ROC, HitRate@600%, NDCG@600%), and five independent repeated runs. All experimental settings and computational environments were standardized to ensure reproducibility and unbiased comparison across models.

All statistical comparisons were performed using two-sided paired *t*-tests . Results are reported as mean ± standard deviation (SD), test statistics, degrees of freedom, exact p-values, 95% confidence intervals (CI), and effect sizes (Cohen's d). Model stability was quantified using standard deviation and the coefficient of variation.

5.7 Comparison Models

Based on the comparative analysis of multivariate anomaly detection models presented in SupplementaryTable 3, existing studies still exhibit notable limitations in methodological innovation and interpretability, highlighting the necessity of the proposed HAO-series models.

Limitations in Structural Modeling and Dynamic Adaptability. Existing methods generally rely on static graph structures or predefined topological relationships, such as the parallel attention layers of the MTAD-GAT model and the similarity embeddings of the GDN model. Consequently, they struggle to adapt to the dynamically evolving correlations of complex systems. Taking the MERLIN model as an example, although it addresses the sensitivity to subsequence length in univariate time series through a dynamic search range, its core mechanism remains univariate local pattern matching. It fails to model the coupling effects between multivariate variables, such as physical constraints or temporal dependencies. Furthermore, MERLIN lacks the capability for explicit spatial topology modeling. Approaches such as the historical inheritance strategy of the TS-GAT model and the independent variable assumption of the MERLIN model result in a rigid structure during the inference phase, rendering them unable to capture dynamic endogenous correlations. To address these limitations, the proposed HAO model introduces an adaptive hyperbolic graph structure discovery algorithm based on hyperbolic space embedding. It uses the law of large numbers to drive autonomous topological evolution and incorporates physical laws as dynamic constraints. This enables the HAO model to continuously mine the endogenous relationships of complex systems, effectively breaking through the rigid limitations of preset structures.

Data Geometric Representation Distortion. Mainstream models (e.g., OmniAnomaly, CAE-M) primarily perform feature embedding in Euclidean space, overlooking the natural geometric properties of the data. For example, the MERLIN model

uses z-normalized Euclidean distance to measure subsequence discrepancies, but its linear metric cannot capture the nonlinear manifold structure of multivariate joint distributions. Although the TranAD model leverages self-attention mechanisms to capture long-range dependencies, it fails to address geometric distortions of high-dimensional data in Euclidean space. Such methods suffer from reduced anomaly sensitivity due to geometric distortion. In contrast, the HAO model proposed in this study introduces a dynamic adjustment mechanism for the curvature of Riemannian manifolds. By adaptively optimizing the geometric attributes of the embedding space, it significantly enhances the discriminability of cross-variable anomaly patterns.

Interpretability Dimension Coupling Defects. Existing interpretation methods suffer from anomaly attribution confusion due to coupled spatio-temporal features, such as the spatio-temporal joint modeling in STADN and the sequence benchmark deviation in MERLIN. The MERLIN model takes subsequences as analytical units, which only reflects local deviation magnitudes and thus fails to reveal cross-variable cooperative anomalies, nor does it possess fine-grained root cause localization capability. Although dynamic graph methods such as GDN achieve node-level localization, they do not decouple the contributions of temporal and structural factors. In contrast, the HAO model employs a spatio-temporal feature independent learning architecture to forcibly decouple multidimensional representations and designs a bimodal scoring system based on geometric and numerical discrepancies. This enables independent parsing of spatio-temporal anomalies and enhances the credibility of anomaly cause interpretation.

Insufficient Adaptability in Small-Sample Scenarios. Threshold selection methods, such as the statistical property adaptation in MERLIN and the validation-set tuning in GDN, rely heavily on data distribution assumptions and they are prone to failure in small-sample scenarios. The MERLIN model estimates thresholds based on univariate subsequence distance statistics, without accounting for multivariate joint distribution characteristics; moreover, estimation errors in μ and σ are accumulated under small-sample condition. Preset threshold methods such as MAD-GAN exhibit poor robustness when facing distribution shifts. In contrast, the small-sample dynamic threshold determination algorithm proposed in this study for the HAO model significantly improves detection stability in data-scarce scenarios through randomized sample composition and dynamic sparsification of the natural graph.

Supplementary Table 3. Description of Multivariate Anomaly Detection Comparison Models.

Number	Method	Threshould Selection(τ)	Model Description	Interpretation Method	Relesed Year
1	MERLIN[6]	I	MERLIN is a parameter-free algorithm designed to efficiently discover anomalous subsequences (discords) of arbitrary length in large-scale time series archives. Its core innovation addresses the sensitivity of traditional time series discord methods to subsequence length parameters by dynamically adjusting the search scope and automatically exploring all possible subsequence lengths to identify the optimal anomaly. This method is based on an improved DRAG algorithm and employs a two-stage candidate set screening and optimization strategy. First, it adaptively sets the distance threshold r by predicting the statistical properties (mean μ and standard deviation σ) of discord distances for adjacent lengths, gradually scanning from the minimum to the maximum length to avoid redundant computations. Second, it uses z-normalized Euclidean distance to measure subsequence dissimilarity, taking the distance between each subsequence and its nearest non-self-matching neighbor as the anomaly score—the higher the score, the greater the likelihood of an anomaly.	A	2021
2	OmniAnomaly[19]	II	OmniAnomaly identifies anomalies by learning robust representations of time series to capture their normal patterns. It integrates Gated Recurrent Units (GRUs) and Variational Autoencoders (VAEs), incorporating key techniques such as stochastic variable connections and planar normalization flows (Planar NF). GRUs are employed to capture complex temporal dependencies within the time series, while VAEs learn latent representations of the data through stochastic variables. Planar NF assists in modeling non-Gaussian posterior distributions, thereby enhancing the robustness of the learned representations. The anomaly score is determined by computing the reconstruction probability of the input data — i.e., the model's ability to reconstruct the input data based on its latent representation. A	A	2019

			lower reconstruction probability indicates a greater deviation of the observed values from the normal pattern and thus a higher likelihood of an anomaly.		
3	MAD-GAN[12]	III	MAD-GAN captures spatio-temporal correlations in data by integrating Generative Adversarial Networks (GANs) with Long Short-Term Memory Recurrent Neural Networks (LSTM-RNNs). The MAD-GAN architecture employs LSTM-RNNs to construct both the generator (G) and discriminator (D): The generator learns the distribution of normal data through adversarial training, while the discriminator distinguishes between real and generated samples. During anomaly detection, the model leverages both the reconstruction capability of the generator and the classification capability of the discriminator. Specifically, it first optimizes latent space mapping to reconstruct test samples and computes reconstruction residuals; simultaneously, it obtains the discriminative loss from the discriminator. Finally, an anomaly score is constructed by combining these two components with a weighted sum.	B	2019
4	MSCRED[21]	IV	MSCRED characterizes the system state at different time steps by constructing multi-scale (resolution) signature matrices, uses convolutional encoders to capture spatial correlations among time series and combines an attention-based ConvLSTM network to learn temporal dependency patterns. The decoder generates a residual matrix by reconstructing the signature matrix and the anomaly score is determined by computing the Frobenius norm of the residual matrix (i.e., the reconstruction error)—the larger the residual, the higher the likelihood of an anomaly.	B	2019
5	MTAD-GAT[23]	II	MTAD-GAT is designed to address the limitation of existing methods that fail to explicitly model inter-variable dependencies. The model deploys two parallel graph attention layers — feature-oriented and time-oriented — to capture dynamic relationships among multiple variables and temporal dependencies, respectively. It further extracts local and long-term temporal features by integrating 1D convolution and GRU layers. For anomaly scoring, a joint optimization strategy is adopted, fusing outputs from both the prediction model (next-step prediction error based on RMSE) and the reconstruction model (reconstruction probability based on VAE). The final anomaly score is generated through a weighted summation of these components, enabling precise detection of entity-level anomalies.	A	2020
6	GDN[24]	IV	GDN is designed to address the challenges of modeling complex inter-sensor relationships and detecting anomalies in multivariate time series data. The method is implemented through four core components. First, it generates a unique feature representation for each sensor via sensor embedding vectors. Second, it constructs a dependency graph among sensors based on embedding similarity using graph structure learning. Third, it employs a graph attention mechanism to aggregate neighbor information and predict future sensor behavior, where the attention weights dynamically reflect the varying influence of different sensors. Finally, it calculates normalized errors between predicted and actual values to produce anomaly scores, followed by smoothing with a sliding window and threshold-based anomaly determination. The anomaly scores integrate local deviations across individual sensors and are aggregated via a max function to obtain a global anomaly score, thereby enabling interpretable detection of both system-level and sensor-level anomalies.	D	2021
7	CAE-M[22]	V	CAE-M is designed to address the challenges of modeling complex spatio-temporal dependencies and mitigating noise interference in multi-sensor time series data. The model adopts a dual-branch architecture. First, a deep Convolutional Autoencoder (CAE) combined with a Maximum Mean Discrepancy (MMD) penalty term is used to extract low-dimensional spatial features, suppressing the interference of noisy data in learning normal patterns. Second, a memory network—comprising a bidirectional LSTM with an attention mechanism and an autoregressive model — is employed in parallel to capture both nonlinear and linear temporal dependencies in the time series. The anomaly score is computed via a composite objective function that jointly optimizes three components: The reconstruction error (from the CAE output), the prediction error (from the memory network output) and the distribution alignment loss (MMD). During the testing phase, the total error of a sample is compared against a dynamic threshold derived from the distribution of training data, enabling fine-grained anomaly determination.	A	2021
8	TranAD[14]	II	TranAD is designed to address the performance bottlenecks of traditional methods under conditions of high data volatility, scarce labels and stringent real-time requirements. Its core architecture adopts an encoder－decoder structure, utilizing a multi-head self-attention mechanism to capture long-term temporal dependencies and incorporates a two-stage adversarial training	A	2022

			strategy. In the first stage, a standard Transformer generates an initial reconstructed sequence and the reconstruction error is computed as a focusing score. In the second stage, this score is used as a self-conditioning input to guide the model in paying enhanced attention to potential anomaly regions, thereby amplifying the anomaly signal. The anomaly score is calculated as a weighted average of the outputs from the two stages—combining the initial reconstruction error and the corrected error after adversarial training. Finally, extreme value theory (POT) is applied to determine anomalies via a dynamic threshold. By leveraging the self-attention mechanism for parallel processing, TranAD significantly improves detection efficiency, while meta-learning (MAML) is employed to optimize generalization performance in few-shot scenarios.		
9	TimesNet[101]	I	TimesNet simultaneously captures the intraperiod and interperiod variations of time series by transforming 1D sequential data into structured 2D tensors and adopting parameter-efficient 2D inception blocks. Specifically, the model first adaptively discovers multiple dominant periods via Fast Fourier Transform (FFT) on the frequency domain, then reshapes the 1D time series into a set of 2D tensors to disentangle and unify intraperiod variations within each cycle and interperiod variations across different cycles. It then utilizes a shared inception block with multi-scale 2D kernels to extract comprehensive temporal 2D variations from the reshaped tensors, and fuses the multi-period representations adaptively according to the amplitude weights derived from FFT.<br>TimesNet employs a residual stacked architecture with repeated TimesBlocks as its backbone, enabling end-to-end representation learning for general time series analysis tasks. For forecasting, imputation, classification, and anomaly detection, the model generates task-specific outputs through tailored projection heads, and identifies anomalies or prediction deviations based on reconstruction or forecasting errors, achieving state-of-the-art performance across five mainstream time series analysis tasks in a unified framework.	A	2022
10	Anomaly Transformer (ATrans)[102]	I	ATrans simultaneously models the prior-association and series-association of time series by reforming the vanilla self-attention into a novel two-branch Anomaly-Attention mechanism. Specifically, the model first generates prior-association using a learnable Gaussian kernel to reflect the adjacent-concentration inductive bias of time points, and computes series-association via standard self-attention to capture global temporal dependencies from the raw sequence. It then calculates the Association Discrepancy as the symmetric KL divergence between these two distributions, which serves as an inherent distinguishable criterion between normal and anomalous time points.<br>The anomaly score is computed by jointly fusing the normalized negative Association Discrepancy and the reconstruction error through element-wise multiplication, which amplifies the separability between normal and anomalous samples. Finally, the model is optimized under a minimax strategy to alternately adjust the prior-association and series-association, further enhancing the discriminability of anomaly scores and enabling accurate unsupervised detection and localization of anomalies in multivariate time series.	A	2023
11	STADN[31]	VI	STADN simultaneously captures the spatial and temporal dependencies of sensor data by integrating Graph Attention Networks (GAT) and Long Short-Term Memory networks (LSTM). Specifically, the model first constructs a relationship graph among sensors and uses GAT to aggregate information from neighboring nodes to capture spatial dependencies. It then employs LSTM to learn both short-term and long-term behaviors of the time series, thereby mining temporal dependencies. The anomaly score is computed based on prediction error—i.e., the absolute difference between the predicted value and the actual observed value for each sensor at each time point. An error refinement module is used to reconstruct the prediction error, enlarging the score gap between normal and anomalous instances. Finally, the anomaly scores are normalized and aggregated via a max function to obtain the anomaly score for each time point, which is used for detecting and locating anomalies.	D	2023
12	GRN[13]	IV	GRN is designed to address the issues of insufficient anomaly detection performance, poor interpretability and gradient vanishing/exploding in industrial control systems. Methodologically, GRN learns node features through sensor embedding vectors, constructs a sensor association graph structure using an attention mechanism and combines Gated Recurrent Units (GRUs) to capture both long-term and short-term temporal dependencies in time series—where the reset and update gates of GRUs help alleviate gradient-related problems. For anomaly scoring, the model generates single-point anomaly scores by	D	2023

			comparing the error between predicted and actual observed values (after normalization). These scores are smoothed using a moving average and then compared against a threshold to determine anomalies. Meanwhile, root cause localization is achieved by maximizing node-level anomaly scores, thereby balancing detection performance with interpretability.		
13	DCdetector[103]	I	DCdetector simultaneously learns discriminative permutation-invariant representations for time series anomaly detection by employing a dual-branch contrastive structure with dual attention operated on multi-scale patched sequences. Specifically, the model first normalizes input multivariate time series via instance normalization and adopts channel-independent patching to divide each dimension into patches, then constructs patch-wise attention to model dependencies across patches and in-patch attention to capture relations within patches under multi-scale settings, and uses upsampling to restore two branches to the same temporal length. It optimizes the network purely by contrastive loss based on symmetric KL divergence between the patch-wise and in-patch representations with stop-gradient for asynchronous training, avoiding model collapse thanks to the intrinsic asymmetric design of dual branches. The anomaly score is computed as the symmetric KL divergence between representations from the two branches, where anomalies yield significantly higher discrepancy values than normal points. Finally, time points with anomaly scores exceeding a predefined threshold are judged as anomalies, enabling effective and robust unsupervised detection and localization for both point-wise and pattern-wise anomalies in time series.	A	2023
14	DGINet[34]	II	DGINet is designed to address the problem of anomaly detection in high-dimensional, high-noise time series within the Industrial Internet, particularly targeting false positives and false negatives caused by delayed sensor responses and the neglect of static feature associations. The model integrates a Dynamic Graph Attention Network (D-GAT) and Informer. D-GAT is used to capture the dynamic associations of sensor features under different temporal states, generating dynamic feature graphs and a Variational Autoencoder (VAE) is employed for sequence reconstruction. Meanwhile, the Informer module analyzes long-term temporal dependencies, identifying key time points as candidate anomaly attack points and combines GRU to extract temporal information from the dynamic feature graphs for prediction. The anomaly score is calculated by fusing the prediction error (Root Mean Square Error, RMSE) and the reconstruction probability and anomalies are finally determined through threshold comparison.	D	2024
15	TS-GAT[26]	IV	TS-GAT is designed to address the issue of continuity loss caused by independently updating graph structures when modeling dynamic relationships among sensors in existing methods. The model is implemented through three core components: 1) A time series encoder (LSTM + Mask layer) that extracts temporal features of sensors and generates embedding representations; 2) A sustainably updatable graph structure learning module, which dynamically adjusts the adjacency matrix through similarity constraint loss and a thresholding strategy, ensuring that historical information is inherited during iterative updates of the graph structure; 3) A prediction decoder (causal convolution) that reconstructs normal patterns by combining sensor embeddings and graph attention representations. The anomaly score is computed using Graph Deviation Scoring (GDC): The prediction error is first standardized by the median and interquartile range and then the maximum value across multiple sensors is taken as the final score. If the score exceeds a predefined threshold, the instance is identified as anomalous.	D	2024
16	HAO(E) / HAO(P) (Ours)	VII	Our proposed HAO model is designed to systematically address four core challenges in explainable anomaly cause analysis: Limited model flexibility due to rigid preset structural constraints, difficulty in identifying anomalous relationships caused by distorted geometric representations of data, contradictory anomaly attributions resulting from multi-dimensional feature confusion and insufficient credibility of manually annotated anomalies. To tackle these issues, HAO constructs a unified multi-task learning framework that deeply explores the above problems through the collaborative optimization of data reconstruction, dynamic structure discovery and geometric parameter learning. Methodologically, HAO encompasses three key innovations. First, it designs a high-precision data reconstruction algorithm based on a multi-level signal parsing attention mechanism, which incorporates geometric priors to reliably learn underlying data patterns and lays the foundation for subsequent analyses. Second, it innovatively proposes an adaptive hyperbolic graph structure discovery method with dynamic optimization in the embedding space. This method not only drives the autonomous evolution of the network topology	E	-

	HAO(H)			to mine intrinsic system relationships but also introduces physical laws as dynamic constraints, thereby enhancing interpretability while preserving the Natural characteristics of the structure. Third, it decouples multi-dimensional features through a spatiotemporal feature-independent learning architecture, avoiding information mixing. In the anomaly detection stage, HAO adopts a bimodal fusion scoring strategy that combines geometric differences (analyzing Natural spatiotemporal structural variations) and numerical differences (based on reconstruction error), supplemented by an anomaly spatiotemporal localization mechanism. Finally, based on the spatio-temporal coordinates of anomaly events, fine-grained correction of low-quality labels is performed, establishing a unified evaluation benchmark for comparative models. Further, by integrating a task-oriented natural topology sparsification dynamic thresholding algorithm, HAO ultimately achieves highly reliable anomaly detection, diagnosis and causal explanation.		

a. Threshould Selection Methods: **I.** By predicting the statistical properties (mean μ and standard deviation σ) of discord distances for adjacent lengths, the distance threshold r is adaptively set. **II.** Peaks-Over-Threshold. **III.** Predefined Value. **IV.** The threshold is defined as $\tau=\beta\cdot \max\{s(t)_{valid}\}$, where $s(t)_{valid}$ denotes the anomaly scores during the validation period and $\beta\in[1,2]$ is set to maximize the F1-score on the validation set. **V.** Decision Threshold (THR). **VI.** Regularity Score Ratio. **VII.** Small-Sample Hybrid Dynamic Threshold Determination Algorithm .
b. Anomaly Interpretation Methods: **A.** Sequence Baseline Deviation. **B.** Matrix Baseline Deviation. **C.** Multivariate Coupled Graph Relationships. **D.** Multivariate Spatio-Temporal Feature Joint Optimization with Dynamically Learned Fusion Graph. **E.** Multivariate Coupled Relationships and Independently Learned Temporal Dependencies.

5.8 Complexity Analysis

The theoretical analysis of spatio-temporal complexity indicators for anomaly detection models is shown in Supplementary Table 4. The three variant models HAO(E), HAO(P), and HAO(H) proposed in this study adopt a unified complexity architecture with a time complexity of $O(N^2d+L^2d+N^2c)$ and a space complexity of $O(N^2+L^2+Nd+Ld)$. Specifically, N^2d and L^2d represent the basic computational costs of multivariate spatial coupled graph convolution and temporal dimension graph convolution, respectively. The additional N^2c overhead is derived from the adaptive curvature distance calculation and dynamic adjacency structure updating module in Euclidean, Poincaré, and hyperbolic geometric spaces. The spatial overhead of the model is mainly dominated by the spatial-temporal adjacency matrix storage terms N^2 and L^2, resulting in a controllable overall complexity level and stable computational cost.

Supplementary Table 4 Time and Space Complexity Comparison of Models.

Time Complexity	Space Complexity
$O(LNd+Ld^2)$	$O(Nd+d^2)$
$O(LNd+Ld^2)$	$O(Nd+d^2)$
$O(LN^2k^2d)$	$O(N^2d)$
$O(LN^2d+Ld^2)$	$O(N^2+Nd+d^2)$
$O(LN^2d)$	$O(N^2+Nd)$
$O(LN^2k^2d)$	$O(N^2d)$
$O(L^2Nd)$	$O(L^2+LNd)$
$O(L^2Nd)$	$O(L^2+LNd)$
$O(LNdk^2)$	$O(LNd)$
$O(LN^2d+Ld^2)$	$O(N^2+Nd+d^2)$
$O(LN^2d+Ld^2)$	$O(N^2+Nd+d^2)$
$O(L^2Nd)$	$O(L^2+LNd)$
$O(LN^2d+Ld^2)$	$O(N^2+Nd+d^2)$
$O(Ld^2+LN^2d)$	$O(N^2+Nd+d^2)$
$O(N^2d+L^2d+N^2c)$	$O(N^2+L^2+Nd+Ld)$

Symbol Definitions: L denotes the sliding window length; N denotes the number of variables; d denotes the hidden dimension; c denotes the compression dimension in HAO; k denotes the convolution kernel size.

In contrast, Transformer-based models including TranAD, ATrans, and DCdetector present a time complexity of $O(L^2Nd)$ that increases quadratically with sequence length, leading to a sharp surge in computational cost and high computing consumption in long-sequence industrial scenarios. The spatial-temporal feature decoupling architecture designed in this study confines the quadratic temporal computational overhead to an independent temporal subnetwork, effectively addressing the computational explosion problem caused by long-sequence inputs and significantly improving the model's adaptability to long time-series tasks. Although mainstream graph temporal models such as GDN and MTAD-GAT achieve

the same $O(N^2d)$ space complexity level as the proposed model, they lack curvature-aware geometric modeling capabilities and fail to explore the deep latent structures of data. The minor $O(N^2c)$ computational overhead introduced in this study supports the core functions of multi-geometric-space adaptive modeling and hierarchical structure capture, achieving an optimal trade-off between model detection accuracy and computational efficiency.

6 Confusion Matrix

Supplementary Table. 5 Binary Classification Confusion Matrix.

	The Model Predicts a Positive Class(P)	The Model Predicts a Negative Class(N)
Reality is a Positive Class(T)	True Positive (TP)	False Negative (FN)
Reality is a Negative Class(F)	False Positive (FP)	True Negative (TN)

7 Comparison Experiments on ROC and P-R Curves

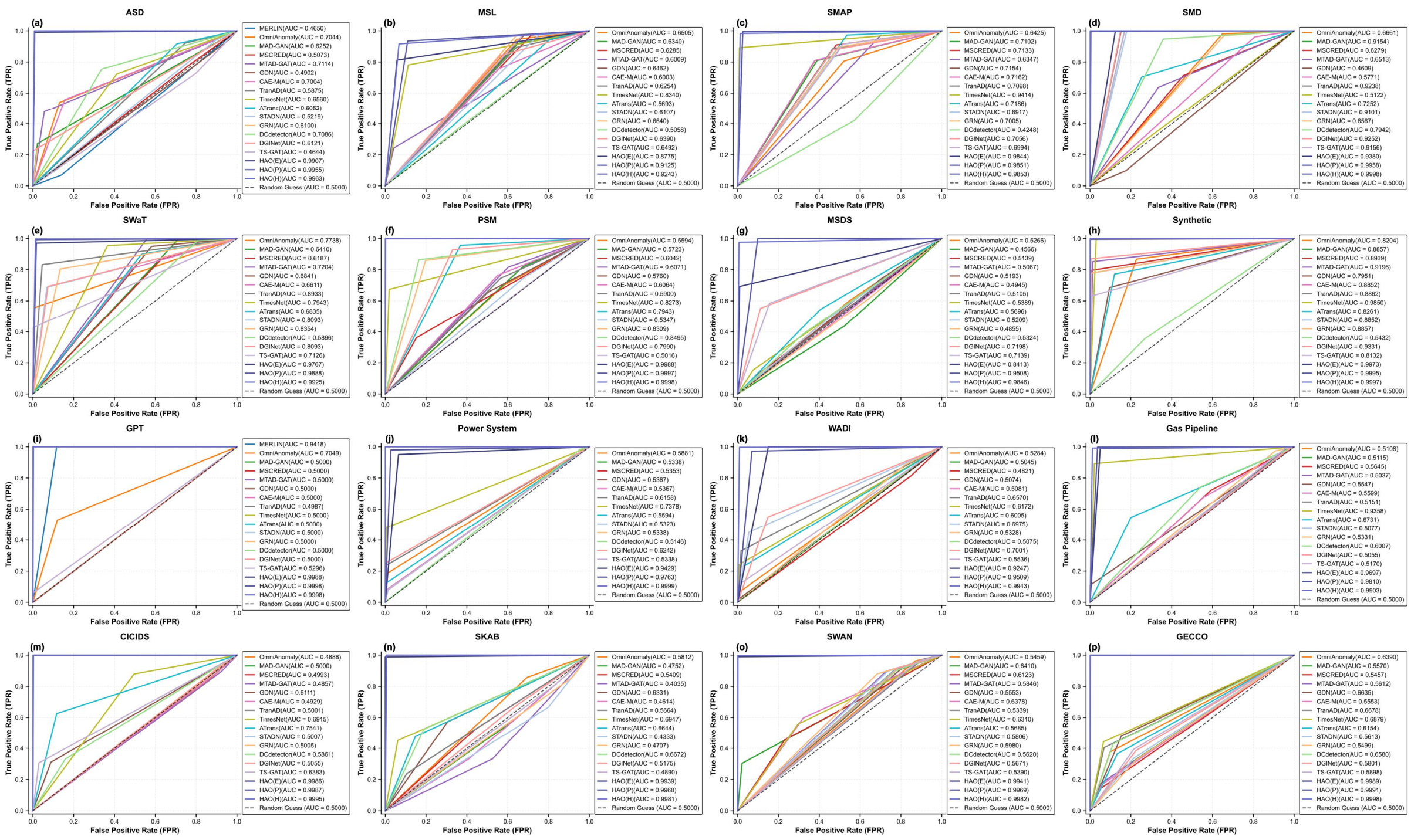

Supplementary Figure 8. Comparison of ROC Performance of Multivariate Anomaly Detection Models Across Multiple Datasets. a, ASD. **b**, MSL. **c**, SMAP. **d**, SMD. **e**, SWaT. **f**, PSM. **g**, MSDS. **h**, Synthetic. **i**, GPT. **j**, Power System. **k**, WADI. **l**, GAS Pipeline. **m**, CICIDS. **n**, SKAB. **o**, SWAN. **p**, GECCO.

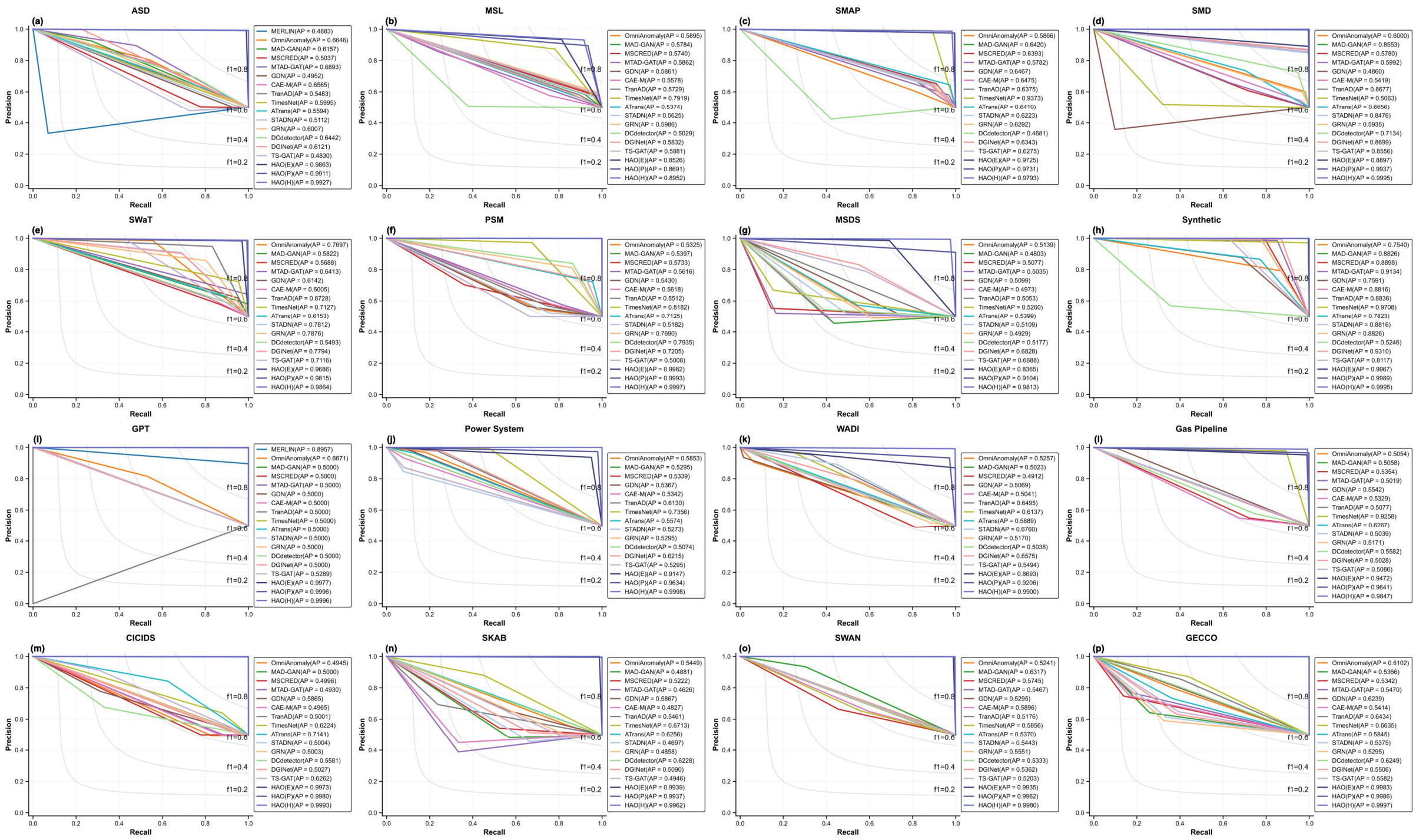

Supplementary Figure 9. Comparison of P–R Curves for Multivariate Anomaly Detection Models. a, ASD. **b**, MSL. **c**, SMAP. **d**, SMD. **e**, SWaT. **f**, PSM. **g**, MSDS. **h**, Synthetic. **i**, GPT. **j**, Power System. **k**, WADI. **l**, GAS Pipeline. **m**, CICIDS. **n**, SKAB. **o**, SWAN. **p**, GECCO.

8 Comparative Experiments on Curvature Variation

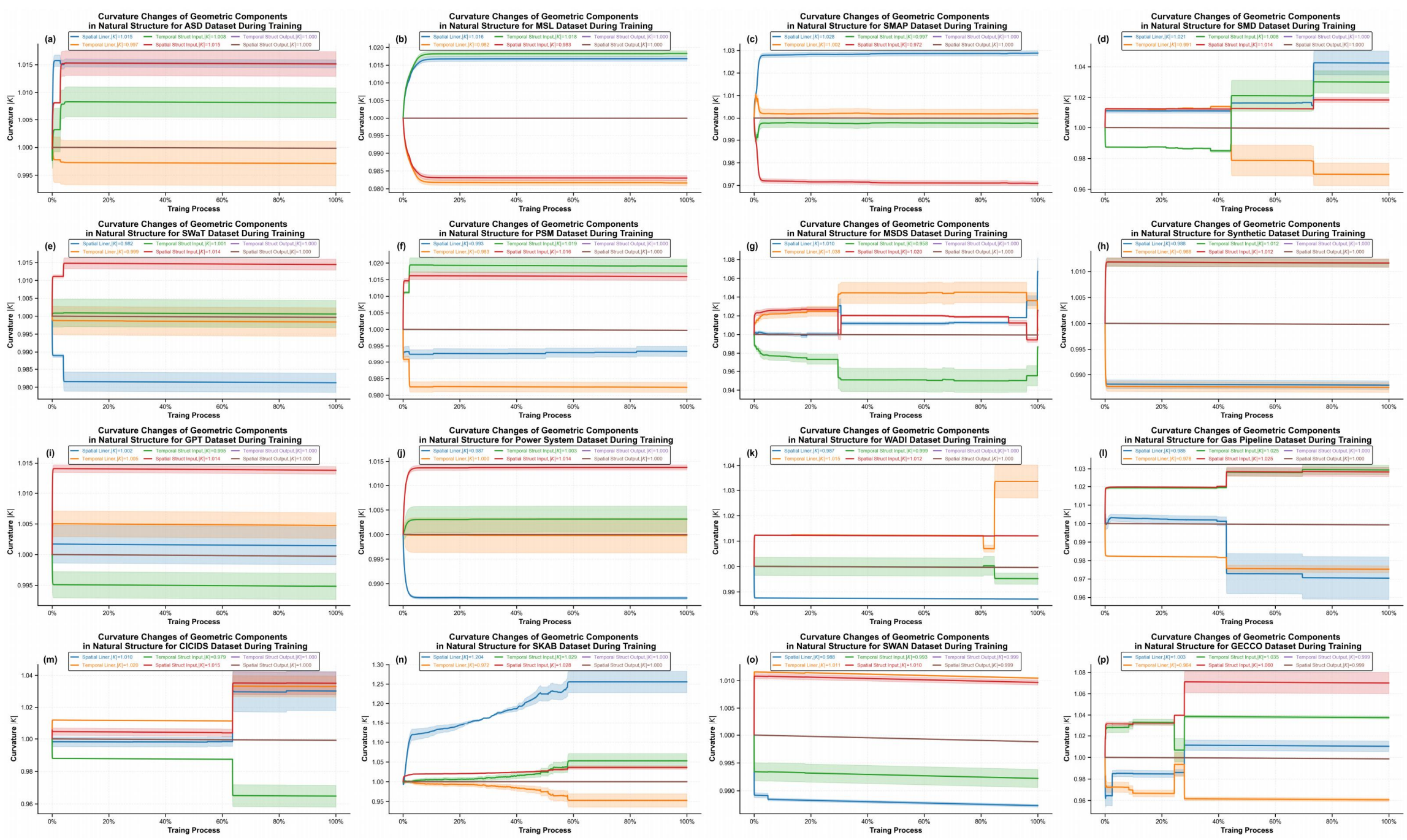

Supplementary Figure 10. Training Stability and Curvature Variation Analysis of the HAO Model. a, ASD. **b**, MSL. **c**, SMAP. **d**, SMD. **e**, SWaT. **f**, PSM. **g**, MSDS. **h**, Synthetic. **i**, GPT. **j**, Power System. **k**, WADI. **l**, GAS Pipeline. **m**, CICIDS. **n**, SKAB. **o**, SWAN. **p**, GECCO.

9 Case Study

For the task of multivariate anomaly detection, this study has demonstrated that natural structures exhibit great potential in anomaly recognition, diagnosis and cause interpretation. However, their module collaboration mechanisms, especially the underlying principles for systematic cause interpretation, remain unclear to date. This study takes the water treatment system[80] as the research object and implements a complete pipeline of anomaly recognition, diagnosis and interpretation, which aims to reveal the natural mechanism of the proposed method, as shown in Supplementary Figure 11.

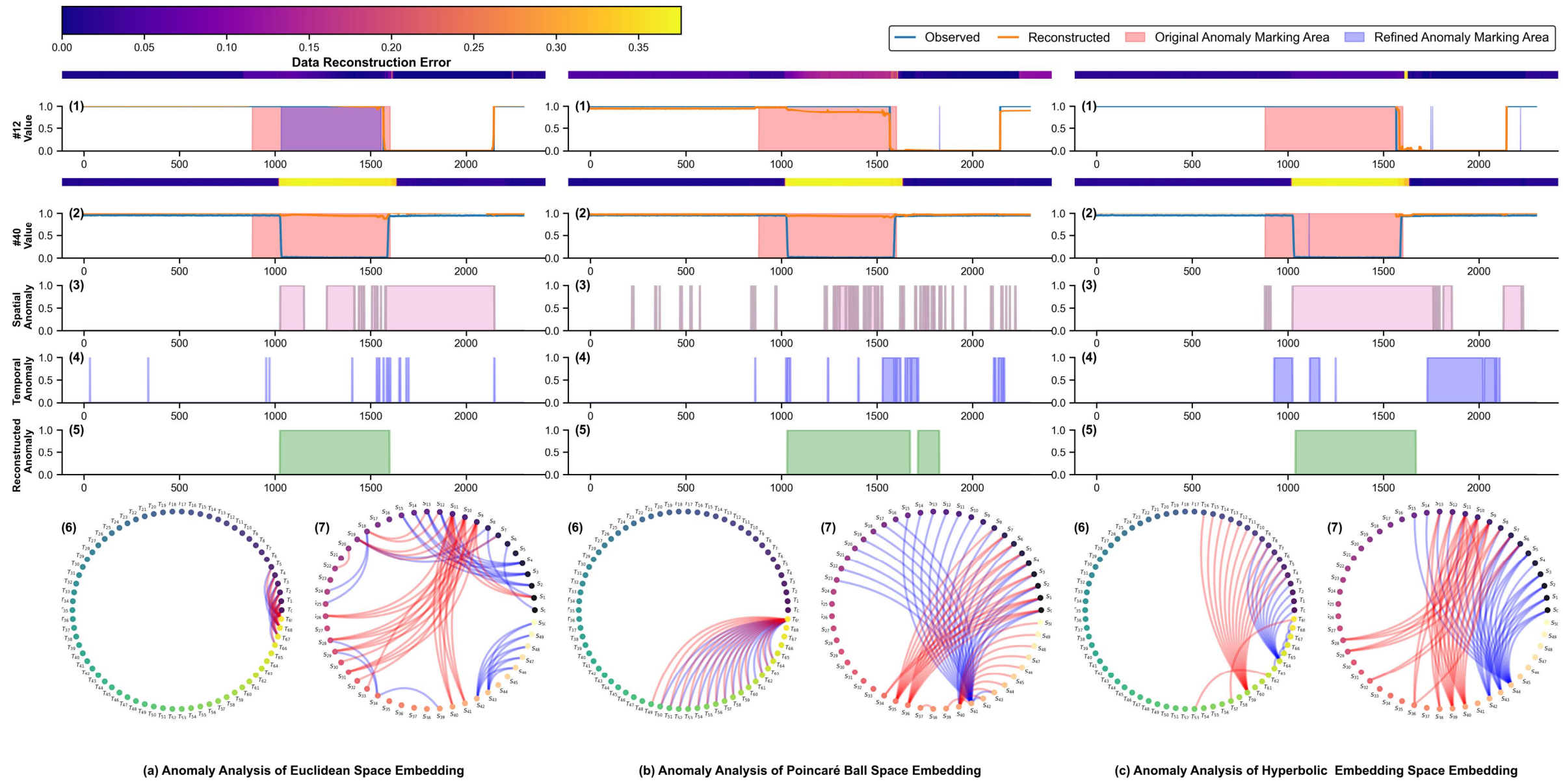

Supplementary Figure 11. Case Study of Anomaly Recognition, Diagnosis and Interpretation on the SWaT Industrial Dataset. It showns a case study focusing on a cyberattack event that occurred between 12:00:55 and 12:04:10 on December 28, 2015, where anomalies were generated in the timestamp range from 12500 to 14800. Two sensors, sensor 12 and sensor 40, were selected for detailed examination. **a**, Anomaly analysis of euclidean space embedding. **a(1)** and **a(2)** show the observed and reconstruction data of the 12th and 40th sensors from the SWaT dataset in Euclidean space. **a(3)** shows spatial anomaly in Euclidean space. **a(4)** shows temporal anomaly in Euclidean space. **a(5)** shows reconstructed anomaly in Euclidean space. **a(6)** shows temporal anomaly interpretation graph in Euclidean space. **a(7)** shows coupled anomaly interpretation graph in Euclidean space. **b**, Anomaly analysis of Poincaré Ball space embedding. **b(1)** and **b(2)** show the observed and reconstruction data of the 12th and 40th sensors from the SWaT dataset in Poincaré Ball space. **b(3)** shows spatial anomaly in Poincaré Ball space. **b(4)** shows temporal anomaly in Poincaré Ball space. **b(5)** shows reconstructed anomaly in Poincaré Ball space. **b(6)** shows temporal anomaly interpretation graph in Poincaré Ball space. **b(7)** shows coupled anomaly interpretation graph in Poincaré Ball space. **c**, Anomaly analysis of Hyperbolic space embedding. **c(1)** and **c(2)** show the observed and reconstruction data of the 12th and 40th sensors from the SWaT dataset in Hyperbolic space. **c(3)** shows spatial anomaly in Hyperbolic space. **c(4)** shows temporal anomaly in Hyperbolic space. **c(5)** shows reconstructed anomaly in Hyperbolic space. **c(6)** shows temporal anomaly interpretation graph in Hyperbolic space. **c(7)** shows coupled anomaly interpretation graph in Hyperbolic space.

The multivariate anomaly detection results of the HAO-series models on SWaT are shown in Supplementary Figure 11(a, b, c). The reconstructed sequence of Sensor 40 deviates significantly within the timestamp range 1000–1700 and its reconstruction error exhibits an anomalous pattern as shown in Supplementary Figure 11(a(5), b(5), c(5)), which indicates that the HAO model can effectively identify potential operational deviations based on historical patterns. The fine-grained label correction results shown in Supplementary Figure 11(a(1)) are highly consistent with manual annotation. In contrast, the Poincaré ball space (Supplementary Figure 11(b(1))) and hyperbolic space (Supplementary Figure 11(b(1))) do not individually mark anomalies in the timestamp range 1000–1700, which suggests that the anomaly may be triggered by collaborative disturbances across multiple sensors rather than the isolated attack timestamps. The anomaly annotation

distribution in hyperbolic space (Supplementary Figure 11(c(1,2))) further corroborates the existence of clustered anomalies. From the perspective of cause localization, the multivariate coupling structure anomalies shown in Supplementary Figure 11(a(3), b(3), c(3)) reveal that sensor attacks are the main inducement. Compared with the Euclidean space (Supplementary Figure 11(a(3))), hyperbolic space exhibits a certain delay in temporal anomaly localization, whereas the Poincaré ball space and hyperbolic space (Supplementary Figure 11(b(3), c(3))) capture anomaly signals earlier, which demonstrates the advantages of non-Euclidean geometric representations in characterizing nonlinear correlations among variables. The sustained deviation at the end of the anomaly-marked interval indicates the existence of a cascading effect among SWaT system sensors, which makes potential anomalies difficult to identify in a timely manner under the Euclidean framework. This also verifies the close relationship between spatial representation capability and anomaly relationship analysis. In the temporal dimension, the dynamic variations of multivariate coupling anomalies (Supplementary Figure 11(c(3))) and temporal anomaly trajectories (Supplementary Figure 11(c(4))) demonstrate that the attacked sensors destroy the temporal consistency of the original operational model, thereby inducing pattern drift in downstream sensors. This chain reaction is particularly significant in hyperbolic space, which suggests that multivariate anomaly detection should consider both spatial representation selection and cross-variable temporal dependency modeling to improve the perception and interpretive validity for complex attack scenarios.

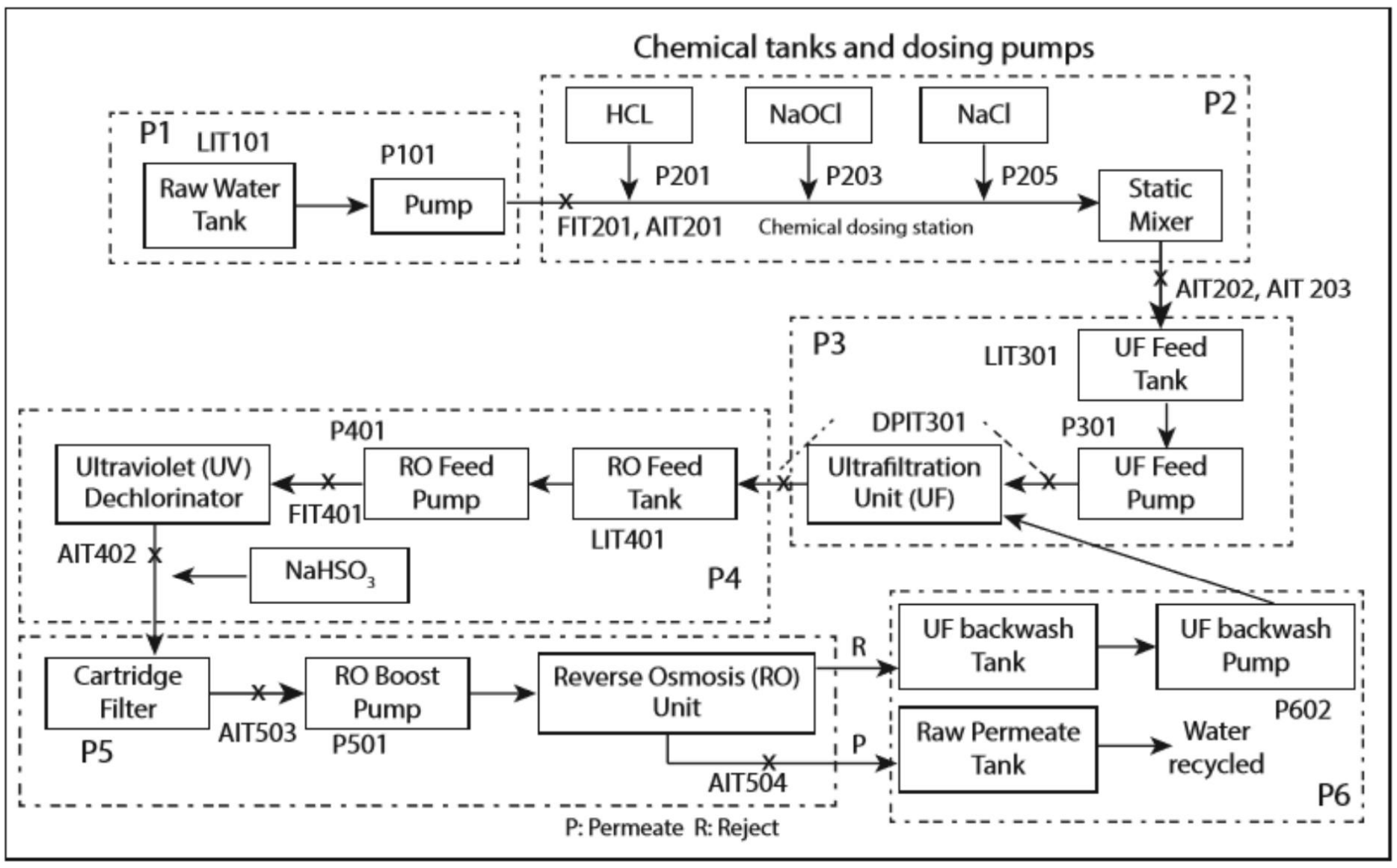

Supplementary Figure 12. Component Layout Diagram of the SWaT Water Treatment Plant[80]

Supplementary Table. 6 Sensor Numbers and Descriptions for SWaT Anomaly Cause Interpretation[80]

No	Name	Type	Description
6	AIT-202	Sensor	PH analyser; Measures HCl level.
7	AIT-203	Sensor	ORP analyser; Measures NaOCl level.
8	FIT-201	Sensor	Flow Transmitter; Control dosing pumps.
9	MV-201	Actuator	Motorized valve; Controls water flow to the UF feed water tank.
10	P-201	Actuator	Dosing pump; NaCl dosing pump.
11	P-202(backup)	Actuator	Actuator&Dosing pump; NaCl dosing pump.
12	P-203	Actuator	Dosing pump; HCl dosing pump.
13	P-204(backup)	Actuator	Actuator & Dosing pump; HCl dosing pump.

To further determine the anomaly causes, this study focuses on the anomaly interpretation graphs with the most significant spatio-temporal anomalies within the timestamp range 1000–1700, as shown in Supplementary Figure 11(a(6, 7), b(6, 7), c(6, 7)). In the Euclidean space, the temporal anomaly interpretation graph (Supplementary Figure 11(a(6))) shows that the temporal dependency relationship is a concomitant phenomenon, while the coupling anomaly interpretation graph exhibits a partial concomitant phenomenon, in which sensors $S_{1,9-13,19}$ show significant anomalies and correlation relationships, which points out the direction for subsequent localization. The temporal anomaly interpretation in the Poincaré ball space (Supplementary Figure 11(b(6))) presents similar results to Supplementary Figure 11(a(6)), which indicates that temporal

anomalies are not the main cause, although weak anomalies in the temporal dimension are detected. The coupling anomaly analysis further reveals that the anomalies of $S_{1,9-13,19}$ are closely related to sensor S_{41}, which suggests a cascading effect transmitted from upstream to downstream sensors. The temporal anomaly interpretation graph in the hyperbolic space (Supplementary Figure 11(c(6))) shows a significant lag from T_{59} to T_{64} in the anomaly occurrence time, which indicates that this attack primarily targets specific types of environmental detection sensors. The coupling anomaly interpretation graph (Supplementary Figure 11(c(7))) further confirms that S_{6-13} are the main potential attack sensors in this anomaly. In summary, both the accuracy of anomaly relationship analysis and the ability of spatio-temporal anomaly decoupling are significantly enhanced with the improvement of data representation capability. To finally determine the core source of this attack event, the functional characteristics of the relevant sensors are analyzed by combining the component distribution map of the SWaT water treatment plant (Supplementary Figure 12) and the system sensor information table (Supplementary Table 6). The anomaly attack occurred from 12:00:55 to 12:04:10 on 2015-12-28, and sensor AIT-202 was attacked and its initial state value which is greater than 7.05 is manipulated to 6. The HAO model not only identifies the initial attack point and direct anomalies, but also accurately locates the abnormal responses of downstream components by mining cross-regional anomaly connections S_{7-13}, thereby revealing the complete propagation path and systematic impact of the attack in the entire physical system. This result provides strong empirical support for capturing the potential, complex anomalies with spatial propagation characteristics.

Supplementary References

[1] Epanechnikov, V. A. Non-parametric estimation of a multivariate probability density. *Theory Probab. Its Appl.* **14**(1), 153-158 (1969).

[2] Yin, C., Zhang, S., Wang, J. and Kim, J.-U. An improved K-means using in anomaly detection. In *Proc. First Int. Conf. Comput. Intell. Theory, Syst. Appl. (CCITSA)*, 129-132 (2015).

[3] Xu, H., Pang, G., Wang, Y. and Wang, Y. Deep isolation forest for anomaly detection. *IEEE Trans. Knowl. Data En.* **35**, 12591-12604 (2023).

[4] Breunig, M. M., Kriegel, H.-P., Ng, R. T. and Sander, J. LOF: identifying density-based local outliers. In *Proc. ACM SIGMOD Int. Conf. Manag. Data*, 93-104 (2000).

[5] Arlia, D. and Coppola, M. Experiments in parallel clustering with DBSCAN. In *Proc. Euro-Par Int. Conf. Parallel Process.* 326-331 (2001).

[6] Nakamura, T. et al. Merlin: parameter-free discovery of arbitrary length anomalies in massive time series archives. In *Proc. IEEE Int. Conf. Data Min. (ICDM)*, 1190-1195 (2020).

[7] Gharghabi, S. et al. An ultra-fast time series distance measure to allow data mining in more complex real-world deployments. *Data Min. Knowl. Dis.* **34**, 1104-1135 (2020).

[8] Zhang, Q. et al. Lightweight and accurate DNN-based anomaly detection at edge. *IEEE Trans. Parall Distrib. Syst.* **33**, 2927-2942 (2022).

[9] Zhu, B. et al. ADFormer: generalizable few-shot anomaly detection with dual CNN-transformer architecture. *IEEE Trans. Instrum. Meas.* **74**, 1-16 (2025).

[10] Yao, Y. et al. SVD-AE: An asymmetric autoencoder with SVD regularization for multivariate time series anomaly detection. *Neural Networks*. **170**, 535-547 (2024).

[11] Zhang, X. et al. Inter-layer explainable variational autoencoder model for multivariate time series anomaly detection. Eng. *Appl. Artif. Intell*. *159*, 111585 (2025).

[12] Li, D. et al. MAD-GAN: multivariate anomaly detection for time series data with generative adversarial networks. In *Proc. Int. Conf. Artif. Neural Netw. (ICANN)*, 703-716 (2019).

[13] Tang, C., Xu, L., Yang, B., Tang, Y. & Zhao, D. GRU-based interpretable multivariate time series anomaly detection in industrial control system. *Comput. Secur.* **127**, 103094 (2023).

[14] Tuli, S. et al. TranAD: Deep transformer networks for anomaly detection in multivariate time series data. In *Proc. VLDB Endow.* **15**, 1201-1214 (2022).

[15] Ma, M., Han, L. and Zhou, C. BTAD: a binary transformer deep neural network model for anomaly detection in multivariate time series data. *Adv. Eng. Inform.* **56**, 101949 (2023).

[16] Luo, W., Liu, W. and Gao, S. A revisit of sparse coding based anomaly detection in stacked RNN framework. In *Proc. IEEE Int. Conf. Comput. Vis. (ICCV)*, 341-349 (2017).

[17] Chen, Y. et al. ImDiffusion: imputed diffusion models for multivariate time series anomaly detection. In *Proc. VLDB Endow.* **17**, 359-372 (2023).

[18] Li, Z. et al. Multivariate time series anomaly detection and interpretation using hierarchical inter-metric and temporal embedding. In *Proc. ACM SIGKDD Conf. Knowl. Discov. Data Min.* (2021).

[19] Su, Y. et al. Robust anomaly detection for multivariate time series through stochastic recurrent neural network. In *Proc. ACM SIGKDD Conf. Knowl. Discov. Data Min.* 2828-2837 (2019).

[20] Hundman, K. et al. Detecting spacecraft anomalies using LSTMs and nonparametric dynamic thresholding. In *Proc. ACM SIGKDD Int. Conf. Knowl. Discov. Data Min.* 387-395 (2018).

[21] Zhang, C. et al. A deep neural network for unsupervised anomaly detection and diagnosis in multivariate time series data. In *Proc. AAAI Conf. Artif. Intell.* **33**, 1409-1416 (2019).

[22] Zhang, Y. et al. Unsupervised deep anomaly detection for multi-sensor time-series signals. *IEEE Trans. Knowl. Data En.* **35**(2), 2118-2132 (2021).

[23] Zhao, H. et al. Multivariate time-series anomaly detection via graph attention network. In *Proc. IEEE Int. Conf. Data Min. (ICDM)*, 841-850 (2020).

[24] Deng, A. & Hooi, B. Graph neural network-based anomaly detection in multivariate time series. In *Proc. AAAI Conf. Artif. Intell.* **35**, 4027-4035 (2021).

[25] Zheng, Y. et al. Correlation-aware spatial-temporal graph learning for multivariate time-series anomaly detection. *IEEE Trans. Neur Net. Lear. Syst.* **35**, 11802-11816 (2024).

[26] Zhang, W. et al. A graph attention network-based model for anomaly detection in multivariate time series. *J. Supercomput.* **80**, 8529-8549 (2024).

[27] Wu, Z. et al. A comprehensive survey on graph neural networks. *IEEE Trans. Neural Netw. Learn. Syst.* **32**, 4-24 (2020).

[28] Zhao, J. et al. Heterogeneous graph structure learning for graph neural networks. In *Proc. AAAI Conf. Artif. Intell.* **35**, 4697-4705 (2021).

[29] Xia, F. et al. Random walks: A review of algorithms and applications. *IEEE Trans. Em. Top. Comput. I.* **4**, 95-107 (2019).

[30] Li, Y. G, Yu, R., Shahabi, C. and Liu, Y. Diffusion convolutional recurrent neural network: data-driven traffic forecasting. In *Proc. Int. Conf. Learn. Represent. (ICLR)* (2018).

[31] Tian, Z. et al. Anomaly detection using spatial and temporal information in multivariate time series. *Sci. Rep.* **13**, 4400 (2023).

[32] Jiang, Y. et al. Multivariable time series anomaly detection using heuristic spatio-temporal graph neural network (in Chinese). *Sci. Sin. Inform.* **53**, 1784-1801 (2023).

[33] Shi Y, et al. Robust anomaly detection for multivariate time series through temporal GCNs and attention-based VAE. *Knowl. Based. Syst* **275**, 110725 (2023).

[34] Huang, X. H. et al. Multivariate time series anomaly detection via dynamic graph attention network and Informer. *Appl. Intell.* **54**, 7636-7658 (2024).

[35] Kipf, T. N. and Welling, M. Semi-supervised classification with graph convolutional networks. In *Proc. Int. Conf. Learn. Represent. (ICLR)* (2017).

[36] Veličković, P. et al. Graph attention networks. In *Proc. Int. Conf. Learn. Represent. (ICLR)* (2018).

[37] Silva, T. C. *Machine Learning in Complex Networks: Modeling, Analysis, and Applications.* Berlin: Springer (2012).

[38] Luo, D. et al. Learning to drop: robust graph neural network via topological denoising. In *Proc. ACM Int. Conf. Web Search Data Min.*, 779-787 (2021).

[39] Zhang, Y., Yang, C., Huang, K. and Li, Y. Intrusion detection of industrial internet-of-things based on reconstructed graph neural networks. *IEEE Trans. Netw. Sci. Eng.* **10**, 2894-2905 (2022).

[40] Yuan, H. et al. DG-Mamba: robust and efficient dynamic graph structure learning with selective state space models. In *Proc. AAAI Conf. Artif. Intell.* **39**, 22272-22280 (2025).

[41] Li, R. et al. Adaptive graph convolutional neural networks. In *Proc. AAAI Conf. Artif. Intell.* **32**, (2018).

[42] Chen, Y., Wu, L. and Zaki, M. Iterative deep graph learning for graph neural networks: better and robust node embeddings. *Adv. Neural Inf. Process. Syst.* **33**, 19314-19326 (2020).

[43] Jin, W., Ma, Y., Liu, X., Tang, X., Wang, S. and Tang, J. Graph structure learning for robust graph neural networks. In *Proc. ACM SIGKDD Int. Conf. Knowl. Discov. Data Min.*, 66-74 (2020).

[44] Franceschi, L. et al. Learning discrete structures for graph neural networks. In *Proc. PMLR.*, **97**, 1972-1982(2019).

[45] Wang, R. J. et al. Graph structure estimation neural networks. In *Proc. Web Conf.*, 342-353 (2021).

[46] Li, K. et al. Reliable representations make a stronger defender: unsupervised structure refinement for robust GNN. In *Proc. ACM SIGKDD Int. Conf. Knowl. Discov. Data Min.*, 925-935 (2022).

[47] Liu, Y. X. et al. Towards unsupervised deep graph structure learning. In *Proc. Web Conf.*, 1392-1403 (2022).

[48] Gu, M. et al. Homophily-enhanced structure learning for graph clustering. In *Proc. ACM Int. Conf. Inf. Knowl. Manag.*, 577-586 (2023).

[49] Wang, H. Z. et al. PROSE: graph structure learning via progressive strategy. In *Proc. ACM SIGKDD Int. Conf. Knowl. Discov. Data Min.*, 2337-2348 (2023).

[50] Khoshraftar, S. and An, A. A survey on graph representation learning methods. *ACM Trans. Intel. Syst. Tec.* **15**, 1-55 (2024).

[51] Li, Y. et al. MCGNet+: an improved motor imagery classification based on cosine similarity. Brain Inf. 9, 3 (2022).

[52] Zheng, Y., Yi, L. & Wei, Z. A survey of dynamic graph neural networks. *Front. Comput. Sci*. 19, 196323 (2025).

[53] Xu, Y. Y. et al. Scalable and effective temporal graph representation learning with hyperbolic geometry. *IEEE Trans. Neural Netw. Learn. Syst.* (2024).

[54] Li, J. D., Dani, H., Hu, X., Tang, J. L, Chang, Y. and Liu, H. Attributed network embedding for learning in a dynamic environment. In *Proc. ACM Conf. Inf. Knowl. Manag.*, 387-396 (2017).

[55] Du, L., Wang, Y., Song, G., Lu, Z. and Wang, J. S. Dynamic network embedding: an extended approach for skip-gram based network embedding. In *Proc. Int. Joint Conf. Artif. Intell.*, 2086-2092 (2018).

[56] Zhou, L. K, Yang, Y., Ren, X., Wu, F. and Zhuang, Y. T. Dynamic network embedding by modeling triadic closure process. In *Proc. AAAI Conf. Artif. Intell.*, Article 71, 571-578 (2018).

[57] Goyal, P., Chhetri, S. R. and Canedo, A. Dyngraph2vec: capturing network dynamics using dynamic graph representation learning. *Knowl.-Based Syst.* **187**, 104816 (2020).

[58] Beladev, M., Katz, G., Rokach, L., Singer, U. and Radinsky, K. GraphERT—transformers-based temporal dynamic graph embedding. In *Proc. ACM Int. Conf. Inf. Knowl. Manag.*, 68-77 (2023).

[59] Kumar, S., Zhang, X. K. and Leskovec, J. Predicting dynamic embedding trajectory in temporal interaction networks. In *Proc. ACM SIGKDD Int. Conf. Knowl. Discov. Data Min.*, 1269-1278 (2019).

[60] Sankar, A., Wu, Y. H., Gou, L., Zhang, W. and Yang, H. DySAT: deep neural representation learning on dynamic graphs via self-attention networks. In *Proc. ACM Int. Conf. Web Search Data Min.*, 519-527 (2020).

[61] Sun, L., Ye, J. D, Peng, H. and Yu, P. S. A self-supervised Riemannian GNN with time varying curvature for temporal graph learning. In *Proc. ACM Int. Conf. Inf. Knowl. Manag.*, 1827-1836 (2022).

[62] Rossi, E. et al. Temporal graph networks for deep learning on dynamic graphs. In *Proc. ICML Workshop Graph Represent. Learn.* (2020).

[63] Xu, D., Ruan, C., Korpeoglu, E., Kumar, S. and Achan, K. Inductive representation learning on temporal graphs. In *Proc. Int. Conf. Learn. Represent.* (2020).

[64] Jin, M., Li, Y. F. and Pan, S. R. Neural temporal walks: motif-aware representation learning on continuous-time dynamic graphs. In *Proc. Adv. Neural Inf. Process. Syst.* **35**, 19874-19886 (2022).

[65] Liu, Y., Pan, S., Wang, Y. G. et al. Anomaly detection in dynamic graphs via transformer. *IEEE Trans. Knowl. Data En.* **35**, 12081-12094 (2021).

[66] Krioukov, D., Papadopoulos, F., Kitsak, M., Vahdat, A. and Boguná, M. Hyperbolic geometry of complex networks. *Phys. Rev. E* **82**, 036106 (2010).

[67] Fu, X.C. et al. ACE-HGNN: Adaptive Curvature Exploration Hyperbolic Graph Neural Network. In *Proc. Int. Conf. Data Min.*, 111-120 (2021).

[68] Nickel, M. and Kiela, D. Learning continuous hierarchies in the Lorentz model of hyperbolic geometry. In *Proc. Int. Conf. Mach. Learn.*, 3776-3785 (2018).

[69] Chami, I. et al. Hyperbolic graph convolutional neural networks. In *Proc. Adv. Neural Inf. Process. Syst.* **32** (2019).

[70] Ranshous, S. et al. Anomaly detection in dynamic networks: a survey. *Wires. Comput. Stat.* **7**, 223-247 (2015).

[71] Ravasz, E. et al. Hierarchical organization in complex networks. *Phys. Rev. E* **67**, 026112-026119(2003).

[72] Dai, J. D. et al. A hyperbolic-to-hyperbolic graph convolutional network. In *Proc. IEEE/CVF Conf. Comput. Vis. Pattern Recognit.* (2021).

[73] Peng, W., Varanka, T., Mostafa, A., Shi, H. & Zhao, G. Hyperbolic deep neural networks: a survey. *IEEE. Trans. Pattern. Anal.* **44**, 10023-10044 (2022).

[74] Gulcehre, C. et al. Hyperbolic attention networks. In *Proc. Int. Conf. Learn. Represent.* (2019).

[75] Pan, K., Palensky, P. and Esfahani, P. M. From static to dynamic anomaly detection with application to power system cyber security. *IEEE Trans. Power Syst.* **35**, 1584-1596 (2019).

[76] Ren, H., Ye, Z. and Li, Z. Anomaly detection based on a dynamic Markov model. *Inform. Sciences.* **411**, 52-65 (2017).

[77] Nakayama, H. et al. A dynamic anomaly detection scheme for AODV-based mobile ad hoc networks. *IEEE Trans. Veh. Technol.* **58**, 2471-2481 (2008).

[78] Li, S., Pandey, A., Hooi, B. et al. Dynamic graph-based anomaly detection in the electrical grid. *IEEE Trans. Power Syst.* **37**, 3408-3422 (2021).

[79] Noble, J. & Adams, N. Real-time dynamic network anomaly detection. *IEEE Intell. Syst.* **33**, 5-18 (2018).

[80] Mathur, A. P. et al. SWaT: a water treatment testbed for research and training on ICS security. In *Proc. Int. Workshop Cyber-phys. Syst. Smart Water Netw. (CySWater)* 31-36 (2016).

[81] Siffer A, et al. Anomaly detection in streams with extreme value theory. In *Proc. 23rd ACM SIGKDD Int. Conf. Knowl. Discov. Data Min.* 1067-1075 (2017).

[82] Gallot, S., Hulin, D. and Lafontaine, J. Riemannian geometry. Berlin: Springer-Verlag, 1990.

[83] Massera, J. L. On Liapounoff's conditions of stability. *Ann. Math.* **50**, 705 – 721 (1949).

[84] Kallenberg, O. Foundations of Modern Probability. New York: Springer, 1997.

[85] Morris, T. and Gao, W. Industrial control system network traffic data sets to facilitate intrusion detection system research. In *Proc. Critical Infrastructure Protection VIII*, **14**, ISBN 978-3-662-45354-4 (2014).

[86] Li, G. & Jung, J. J. Deep learning for anomaly detection in multivariate time series: approaches, applications, and challenges. *Inform. Fusion.* **91**, 93 – 102 (2023).

[87] Moritz, S., Rehbach, F., Chandrasekaran, S., Rebolledo, M. & Bartz-Beielstein, T. GECCO Industrial Challenge 2018 Dataset: a water quality dataset for the "Internet of Things: Online Anomaly Detection for Drinking Water Quality" competition at the Genetic and Evolutionary Computation Conference 2018, Kyoto, Japan. *Zenodo* **3884398** (2018).

[88] Abdulaal, A. et al. Practical approach to asynchronous multivariate time series anomaly detection and localization. In *Proc. 27th ACM SIGKDD Int. Conf. Knowl. Discov. Data Min.* 2485 – 2494 (2021).

[89] Nedelkoski, S. et al. Multi-source distributed system data for AI-powered analytics. In *Proc. European Conference on Service-Oriented and Cloud Computing* 161 – 176 (Springer, 2020).

[90] Chuadhry, M. A., Palleti, V. R. and Mathur, A. P. WADI: a water distribution testbed for research in the design of secure cyber physical systems. In *Proc. 3rd Int. Workshop Cyber-Phys. Syst. Smart Water Netw.* 25 – 28 (2017).

[91] Pan, S., Morris, T. and Adhikari, U. Classification of disturbances and cyber-attacks in power systems using heterogeneous time-synchronized data. *IEEE Trans. Ind. Inform.* **11**, 650 – 662 (2015).

[92] Beaver, J. M., Borges-Hink, R. C. and Buckner, M. A. An evaluation of machine learning methods to detect malicious SCADA communications. In *Proc. 12th Int. Conf. Mach. Learn. Appl. (ICMLA)* **2**, 54 – 59 (2013).

[93] Sharafaldin, I., Lashkari, A. H. and Ghorbani, A. A. Toward generating a new intrusion detection dataset and intrusion traffic characterization. In *Proc. 4th Int. Conf. Inf. Syst. Secur. Privacy (ICISSP)*. (2018).

[94] Katser, I. D. and Kozitsin, V. O. Skoltech anomaly benchmark (SKAB). Kaggle (2020).

[95] Angryk, R. A. et al. Multivariate time series dataset for space weather data analytics. *Sci. Data* **7**, 227 (2020).

[96] Zheng, Y. et al. Correlation-aware spatial – temporal graph learning for multivariate time-series anomaly detection. *IEEE Trans. Neur Net. Lear. Syst.* **35**, 11802 – 11816 (2024).

[97] Paparrizos, J. et al. Volume under the surface: a new accuracy evaluation measure for time-series anomaly detection. In *Proc. VLDB Endow.* **15**, 2774 – 2787 (2022).

[98] Kingma, D.P. & Ba, J. Adam: a method for stochastic optimization. (2014).

[99] Saleh, N. & Mashaly, M. A dynamic simulation environment for container-based cloud data centers using ContainerCloudSim. In *Proc. Int. Conf. Intell. Comput. Inf. Syst.* 332 – 336 (2019).

[100] Paszke, A. et al. PyTorch: an imperative style, high-performance deep learning library. *Adv. Neural Inf. Process. Syst.* **32**, 8026 – 8037 (2019).

[101] Wu, H. et al. TimesNet: Temporal 2D-Variation Modeling for General Time Series Analysis. In *Proc. Int. Conf. Learn. Represent.* (2023).

[102] Xu, J. et al. Anomaly Transformer: Time Series Anomaly Detection with Association Discrepancy. In *Proc. Int. Conf. Learn. Represent.* (2022).

[103] Yang, Y. et al. DCdetector: Dual Attention Contrastive Representation Learning for Time Series Anomaly Detection. In *Proc. ACM SIGKDD Conf. Knowl. Discov. Data Min.* 1 – 17 (2023).
