# Supplementary material for: Hyperbolic adaptive spatial-aware multivariate time series anomaly detection
Source: Nat Commun. 2026 Jul 27;17:9116. doi: 10.1038/s41467-026-75881-1 (PMC13518828; doi:10.1038/s41467-026-75881-1)
Supplement: Supplementary file 2 [file 41467_2026_75881_MOESM2_ESM.pdf]

nature portfolio

# Peer Review File

# Hyperbolic Adaptive Spatial-Aware Multivariate Time Series Anomaly Detection

Corresponding Author: Professor Zhonglin Ye

**This file contains all reviewer reports in order by version, followed by all author rebuttals in order by version.**

Version 0:

Reviewer comments:

Reviewer #1

(Remarks to the Author)
The manuscript introduces the Hyperbolic Adaptive Spatial-Aware (HAO) model, which integrates adaptive curvature-based hyperbolic pretraining, graph structure learning, and mixed dynamic threshold determination to address challenges in anomaly detection.
These contributions are both innovative and well-executed, marking a significant advancement in multivariate anomaly detection tasks. The paper tackles several critical challenges, including handling high data volatility, overcoming rigid structural assumptions, and ensuring low-latency inference, which are vital for real-world industrial applications. The model is validated across six open-source datasets, achieving superior performance (e.g., >99.85% in F1, ACC, and AUC metrics). The improvements over baselines, including a 6% gain in AUC compared to the TranAD model, highlight its effectiveness. The proposal for cross-regional anomaly causality interpretation and structure-difference-based analysis offers a robust explanation of global system anomalies, a critical feature for practical applications.
Here are some suggestions for Improvement:
1. The manuscript’s formatting is inconsistent, particularly in the alignment of equations, headings, and figures. Standardizing these elements would significantly enhance readability and professionalism.
2. Some sections, especially those explaining hyperbolic transformations and graph structure learning, use overly complex sentences. Simplifying these explanations would make the content accessible to a broader audience.
3. While figures such as the framework diagram (Figure 4) and hyperbolic space illustrations (Figure 5) are informative, they could benefit from higher resolution and more detailed captions to improve interpretability.
4. While the method demonstrates strong performance, the paper could include a discussion on scalability for large-scale or dynamic datasets and its real-time deployment feasibility.
5. The framework is highly specialized for multivariate anomaly detection but could benefit from a discussion on potential applications in other domains, such as finance, healthcare, or cybersecurity.
6. The conclusion mentions potential improvements but lacks specific directions. Elaborating on extensions, such as integrating reinforcement learning or exploring domain adaptation techniques, would provide a clearer research trajectory.
7. While the manuscript uses standard metrics (AUC, F1, etc.), including additional metrics like precision-recall curves for imbalanced datasets would enrich the evaluation.
Dataset Details:
8. A more detailed discussion of the datasets, including their challenges and how the proposed model addresses them, would provide better context for the results.
Threshold Determination:
9. The Small Sample Dynamic Mixed Threshold Determination Algorithm (SSA) is a key innovation but is described briefly. Expanding on its implementation and parameter sensitivity would strengthen its contribution.

(Remarks on code availability)

Reviewer #2

(Remarks to the Author)
The multivariate anomaly detection is an interesting research problem and has great application value. The paper introduces hyperbolic graph learning into hierarchical structure mining of multivariate data and proposes a complete

multivariate anomaly detection framework for detection accuracy, interpretability, and robustness. Extensive experiments are conducted to verify the effectiveness and efficiency of this framework. But the paper still exists the following important problems:
1.Figure 1 does not provide valuable information and should be revised to show a comprehensible diagram.
2.The motivation of the work is not explicit. The paper does not clearly explain why the multivariate anomaly detection needs hyperbolic space.
3.In the introduction section, the paper should discuss the direct challenges of multivariate anomaly detection instead of focusing on natural data structures analysis.
4.The 2.1 section of related work part is not complete. Currently, multivariate anomaly detection has been widely studied but authors do not highlight the difference of the proposed method with existing methods.
5.The related work section lacks the outline of hyperbolic graph learning research. However, it has been used as the key technique for the proposed framework.
6.Whether is the AH-GSL effective for hyperbolic graph structure learning? Because the feature reconstruction optimization may not promote the adjacent structure match the hyperbolic structure characteristics. Here, authors should give more theory analysis.
7.There are many representation errors in this paper, such as the data scientific representation in Table 1, etc.
8.The proposed multivariate anomaly detection framework is complete, but the novelty of designed algorithm is limited, especially, the AH-PF framework greatly relies on existing hyperbolic graph learning models and makes small revision. In addition, the anomaly evaluation method is also simple.
9.The paper should perform the case study experiment to verify the effectiveness of learned hyperbolic graph structure.

(Remarks on code availability)
The code provides a readme file to introduce the implementation steps and gives datasets links.

Reviewer #3

(Remarks to the Author)
This manuscript presents a theoretical framework for multivariate anomaly detection and evaluates its performance on several datasets by comparing it against existing models. While the authors aim to introduce an innovative framework, the manuscript, in its current form, has significant limitations in terms of clarity, rigor, and novelty. Several issues should be addressed before consideration for publication.
1. The authors present Figure 1 as an illustration of "multivariate anomaly detection tasks." However, the figure is merely provided without an explanation in the introduction. The subsequent explanation of different phases—anomaly identification, anomaly diagnosis, and anomaly cause explanation—lacks a clear connection to the figure. This disjointed presentation may confuse readers.
2. The manuscript states: “However, existing studies generally adapt structures to data rather than data to structures, meaning that structures preset based on prior knowledge may not accurately reflect the natural data structure, thereby affecting the objectivity of anomaly detection and causality interpretation.” The distinction between "structures to data" and "data to structures" is unclear and requires a more precise explanation. If the authors are referring to dynamically evolving models adapting to data, numerous existing methodologies have already addressed this issue in anomaly detection. Below are some relevant references for the authors to refer to.
• Ranshous, Stephen, et al. "Anomaly detection in dynamic networks: a survey." Wiley Interdisciplinary Reviews: Computational Statistics 7.3 (2015): 223-247.
• Ren, H., Ye, Z., & Li, Z. (2017). Anomaly detection based on a dynamic Markov model. Information Sciences, 411, 52-65.
• Pan, Kaikai, Peter Palensky, and Peyman Mohajerin Esfahani. "From static to dynamic anomaly detection with application to power system cyber security." IEEE Transactions on Power Systems 35.2 (2019): 1584-1596.
• Li, Shimiao, et al. "Dynamic graph-based anomaly detection in the electrical grid." IEEE Transactions on Power Systems 37.5 (2021): 3408-3422.
• Noble, Jordan, and Niall Adams. "Real-time dynamic network anomaly detection." IEEE Intelligent Systems 33.2 (2018): 5-18.
• Nakayama, Hidehisa, et al. "A dynamic anomaly detection scheme for AODV-based mobile ad hoc networks." IEEE transactions on vehicular technology 58.5 (2008): 2471-2481.
• Liu, Yixin, et al. "Anomaly detection in dynamic graphs via transformer." IEEE Transactions on Knowledge and Data Engineering 35.12 (2021): 12081-12094.
3. The current version of the manuscript contains writing issues, including grammatical errors and unclear expressions, which hinder readability. The writing should be more fluid, precise, and aligned with academic standards. For instance, "…, where M represents the number of sensors, W represents the sampling window length."
4. The figures presented in the manuscript are inadequately explained. Instead of providing only a brief caption, the authors should offer detailed descriptions and analyses. For instance, in Figure 5, the results are not explicitly interpreted. The authors should clearly explain the insights derived from the figure and how it supports their claims. Without such explanation, the figure lacks scientific value and leaves readers uncertain about its intended message.
5. The experimental design is not well justified. In the Dataset Description section, training and test data proportions are specified. However, when conducting anomaly detection experiments, the authors state: “90% of the data is used for training all models, with the remaining 10% serving as a test set, to evaluate model performance.” Given that anomaly detection inherently deals with imbalanced data, where anomalous samples are significantly fewer than normal samples, using only 10% of the data for testing undermines the credibility of the results. A small test set may lead to biased evaluations, reducing the reliability of the reported performance. The authors should explain why they did not maintain the same train-test split ratio as described in the Dataset Description section. They should justify the choice of the test set size and its potential impact on

anomaly detection performance, especially in cases where anomalies are rare. A more balanced and statistically sound evaluation strategy should be considered to ensure the robustness and generalizability of the results.
6. The authors should increase the number of datasets (>15) to provide stronger empirical validation of their model's generalizability. The current dataset selection is insufficient to convincingly demonstrate the robustness of the proposed approach. Expanding the experimental setup with diverse datasets would enhance the manuscript's impact and credibility.
7. The manuscript does not clearly define how anomalies are labeled in the experimental datasets. The authors must specify how they determine which samples are normal and which are anomalous. In the absence of domain-specific knowledge, can anomalies be identified based on waveform characteristics, inter-variable relationships, amplitude thresholds, or other quantitative measures? A more detailed discussion is necessary to establish the validity of the anomaly labels used in the study.
8. The manuscript contains numerous abbreviations. So it is recommended that the authors include a table listing all abbreviations along with their full forms to improve readability and accessibility for a broader audience.

(Remarks on code availability)

Version 1:

Reviewer comments:

Reviewer #2

(Remarks to the Author)
Thank authors' response one by one for the comments. But these problems need to be solved before considering publication.
1.There are inconsistent representations in the revised manuscript, such as "To the best of
our knowledge, the hyperbolic adaptive pretraining framework, adaptive hyperbolic graph structure learning, cross-region anomaly interpretation," in the ABSTRACT section, etc.
2.There are format errors in the manuscript, such as the first author affiliation is not labeled in the manuscript, "coupling correlation relationship describes in the 9th page", etc.
3.The whole language representation needs to be improved.
4.Some unimportant sections should be removed in the manuscript, or they are represented by the Appendix section. This enables the research work focus on key contents.

(Remarks on code availability)

Reviewer #3

(Remarks to the Author)
1. From a system design and methodological integration standpoint, this paper presents a conceptually well-organized framework with potential for practical value. However, from the standpoint of theoretical depth or transformative methodological innovation, the work does not appear to reach a level of breakthrough that would justify publication in a high-impact venue such as Nature Communications. Additionally, its current implementation and experimental setup raise concerns about the validity and generalizability.
2. According to the description of "Data set division" on page 25, the experimental setup appears to have some critical limitations that warrant closer examination. The training set consists solely of normal data, while the test and validation sets are constructed from artificially partitioned and recombined abnormal samples. This process fragments originally continuous abnormal events into isolated points or patterns, which are then distributed across different subsets. Such manipulation severely disrupts the contextual integrity of the anomalies and impairs the model's ability to learn the temporal dynamics and structural patterns associated with real-world anomalies. More critically, this partitioning strategy breaks the chronological order of the time series, thereby violating the core assumption of time series modeling — that models are trained on past data to predict or detect future events. As a result, the evaluation no longer reflects a realistic deployment scenario and fails to simulate the continuity and temporal causality of anomaly occurrences in practical applications.
3. Although the proposed method is evaluated on 16 benchmark datasets, the results do not show a clear performance advantage in terms of the key evaluation metric, F1-score. More importantly, in many real-world applications such as security alerting, fault diagnosis, and medical anomaly detection, missed detections (false negatives) are often more critical than false alarms. In such contexts, Recall becomes a crucial metric. Unfortunately, the proposed method also underperforms in Recall compared to existing baseline models on most datasets, further undermining its practical effectiveness and competitiveness.
4. Regarding reproducibility, the GitHub repository currently lacks several components required for end-to-end replication. For instance, in main.py, modules such as src.merlin, src.pot, and src.NoPOT appear to be missing or inaccessible.

(Remarks on code availability)
Regarding reproducibility, the GitHub repository currently lacks several components required for end-to-end replication. For instance, in main.py, modules such as src.merlin, src.pot, and src.NoPOT appear to be missing or inaccessible.

Version 2:

Reviewer comments:

Reviewer #2

(Remarks to the Author)
Thanks for authors' revision. My concerns have been addressed.

(Remarks on code availability)

Reviewer #4

(Remarks to the Author)
This paper proposes a novel method for multivariate time series anomaly detection. The proposed method uses an adaptive hyperbolic pre-training framework to capture the relationship between different variates, and employs a graph structure discovery mechanism to automatically learn a graph structure that connects different variates, without replying on predefined structure. A multi-level signal parsing attention mechanism is also introduced to tackle insufficient model robustness caused by complex signals. Experiments are conducted to show the effectiveness of the proposed method.

The paper has several strong points:
1. The research problem is significant.
2. The empirical discussions are solid.
3. The proposed method is well designed.
4. The experiments are extensive.

Despite its merit, this paper still requires a minor revision. Detailed comments are as follows:
1. The claims of "first time" are too strong and should be toned down or supported with clearer comparisons. At least, you can mention "to the best of our knowledge" for some unsure claims.
2. The proposed framework integrates multiple components, but the core novelty is not clearly distinguished. I suggest the authors to highlight the most significant 1-2 novel points in introduction/abstract, and the rest points can be regarded as supplemental.
3. Why hyperbolic space is better for such learning problem? More detailed discussion, especially from a theoretical perspective, is expected.
4. Hyperbolic operations are computationally expensive, as well as the other complex components in the proposed method. In this case, a complexity analysis is expected to discuss the running efficiency of the proposed method. If possible, empirical results (e.g. run time comparison) are also preferred.
5. The claim of causal interpretation is not sufficiently justified and appears to rely on heuristic explanations. The correctness and reliability of the "explanations" should be further discussed.
6. The main figure (Figure 8) contains excessive details and dense textual annotations, which significantly affect its readability. It is recommended to simplify the textual content and improve the visual organization by clearly separating different functional regions (e.g., modules or stages). This would help readers better understand the overall framework.

(Remarks on code availability)
The codes look well organized.

Reviewer #5

(Remarks to the Author)
This paper proposes using hyperbolic geometry for multivariate time series anomaly detection (TSAD). The core intuition that sensor (or univariate TS) coupling networks exhibit hierarchical structure better captured by hyperbolic space than Euclidean space is reasonable. However, the execution diverges significantly from what the TSAD community has come to expect of rigorous work in this area. The strongest contributions in recent TSAD research share a common trait: methodological focus, center on one clear idea, validate it thoroughly and let the results speak. This manuscript takes the opposite approach, simultaneously introducing five components, a label correction framework, a new thresholding algorithm and an interpretability pipeline, spread across 42 pages. The sheer volume makes it difficult to isolate what actually drives the reported improvements.

The evaluation protocol does not align with current community standards. Since Wu & Keogh (2022) demonstrated that traditional point-adjust F1 creates an illusion of progress, the field has moved
toward more robust metrics: VUS (Paparrizos et al., VLDB 2022), range-based and affiliation-based measures (Huet et al., 2022). None of these appear here. More critically, the model corrects the
anomaly labels using its own learned topology, then evaluates all methods on those corrected labels. This circularity undermines the reported F1-scores of 0.99+, which are already unusually high
for datasets like WADI and CICIDS where the community consistently reports much lower perfs.
The benchmark suite itself omits the most prominent recent methods, including Anomaly Transformer,
DCdetector and TimesNet, while relying heavily on baselines from 2019.

The datasets used are individually well-known in the community, but the way they are handled raises questions. The artificial recombination of normal and anomalous data for test and validation
sets, rather than strict temporal splitting, contradicts standard practice for time series evaluation. The sliding window argument in the rebuttal preserves local continuity but does not address the global temporal ordering violation.

The theoretical content is presented with considerable formalism but limited depth. Classical results from probability theory are restated in the appendix without being formally connected to the
paper's specific convergence claims. The adaptive curvature, a key theoretical motivation, converges to the same fixed value of 1.0 across all datasets, a result that the paper reports but does not discuss, even though it directly contradicts the need for learning curvature at all.

The TSAD field has matured considerably in recent years. The community now values clean experimental protocols, honest baselines, appropriate metrics and focused contributions over architectural
complexity. This manuscript would benefit substantially from engaging with that direction: one clear contribution, properly measured, on well-established benchmarks, compared against current
state-of-the-art under identical conditions.

(Remarks on code availability)

Version 3:

Reviewer comments:

Reviewer #2

(Remarks to the Author)
The authors'responses have addressed my concerns.

(Remarks on code availability)

Reviewer #4

(Remarks to the Author)
I appreciate the authors for the detailed response. The current version of manuscript looks nice from my side. In this case, I suggest to accept this paper.

(Remarks on code availability)

**Open Access** This Peer Review File is licensed under a Creative Commons Attribution 4.0 International License, which

permits use, sharing, adaptation, distribution and reproduction in any medium or format, as long as you give appropriate credit to the original author(s) and the source, provide a link to the Creative Commons license, and indicate if changes were made.
In cases where reviewers are anonymous, credit should be given to 'Anonymous Referee' and the source.
The images or other third party material in this Peer Review File are included in the article's Creative Commons license, unless indicated otherwise in a credit line to the material. If material is not included in the article's Creative Commons license and your intended use is not permitted by statutory regulation or exceeds the permitted use, you will need to obtain permission directly from the copyright holder.
To view a copy of this license, visit https://creativecommons.org/licenses/by/4.0/

# REVISION OF MANUSCRIPT IN NATURE COMMUNICATIONS #NCOMMS-24-82438

## Explanation of Revisions

We are submitting a revised version of our paper, "Hyperbolic Adaptive Spatial Perception Multivariate Time Series Anomaly Detection" (ID: #NCOMMS-24-82438). We sincerely thank the reviewers for their valuable and constructive comments. We thoroughly revise the paper in accordance with these suggestions, provide detailed responses to all review comments, and add new analytical content to further strengthen our research. We hereby declare that this paper has not been published elsewhere in any form.

In order to ensure that the revisions made in response to editorial comments and feedback from reviewers are clear, standardized, and easy to review, the following is an explanation of how responses and revisions are marked:

- **Reviewer comment presentation:** We strictly quote the original comments of the reviewers and highlight them in black. For example: **The manuscript introduces the Hyperbolic Adaptive Spatial-Aware...**
- **Response Marking of responses to comments:** For each review comment, our response is uniformly marked in blue bold underlined format, labeled as: **Response 1 :**.
- **Explanation of modifications:** All revisions made to the text, whether content adjustments or wording optimizations, are marked in red bold underlined format, labeled as: **Revision 1 :** with a brief explanation of the key points of the revision.
- **Modification Location Identification:** To facilitate efficient review, we employ red bold formatting **[(Page X) Section Title]** to mark revision locations. Example: **[(Page 7) 2.2.4 Hypergraph Representation Learning]**.

## Reviewer #1 (Remarks to the Author):

The manuscript introduces the Hyperbolic Adaptive Spatial-Aware (HAO) model, which integrates adaptive curvature-based hyperbolic pretraining, graph structure learning, and mixed dynamic threshold determination to address challenges in anomaly detection. These contributions are both innovative and well-executed, marking a significant advancement in multivariate anomaly detection tasks. The paper tackles several critical challenges, including handling high data volatility, overcoming rigid structural assumptions, and ensuring low-latency inference, which are vital for real-world industrial applications. The model is validated across six open-source datasets, achieving superior performance (e.g., >99.85% in F1, ACC, and AUC metrics). The improvements over baselines, including a 6% gain in AUC compared to the TranAD model, highlight its effectiveness. The proposal for cross-regional anomaly causality interpretation and structure-difference-based analysis offers a robust explanation of global system anomalies, a critical feature for practical applications. Here are some suggestions for Improvement:

**Response :** We sincerely appreciate your valuable comments, which cover expression optimization, expansion of experimental data, and in-depth discussion of multiple application areas. These comments have significantly improved the quality of the paper. In this round of revisions, we have carefully addressed all comments. The main revisions are summarized below:

- We comprehensively unify the layout formats of mathematical formulas, titles, and charts in terms of centering and alignment standards. We redraw the hyperbolic space diagram (Fig.4) and add curvature annotations and geometric characteristic descriptions. For the HAO model framework diagram (Fig.7), we optimize the logical flow and key step identifiers. We add detailed legend descriptions to all charts and strengthen the correspondence between text and images.
- We simplify the theoretical expressions of the HAO model and expand our experiments. We reconstruct the model's theoretical component, simplifying the expressions of complex elements like hyperbolic transformations. We add 16 cross-domain datasets for testing, including those from industrial, IT, and space weather monitoring fields, with scales ranging from thousands to millions. We also supplement evaluation metrics for imbalanced datasets, such as P-R curves and AP values (Section 4.4.2). To validate the parameter sensitivity of the small-sample dynamic threshold mixing algorithm, we conduct two sets of comparative experiments. One set uses short, medium, and long observation windows ($W = \{5, 10, 30, 90, 200, 500\}$), while the other explores the impact of different small-sample partitioning ratios (0.1–0.9). Additionally, we perform hyperparameter experiments to jointly validate the algorithm's responsiveness to parameter changes.
- We expand the discussion section by adding the "multivariate natural structure and domain transfer" analysis (Section 4.6.3). We add to the discussion section expanding on the feasibility of real-time model deployment and memory optimization strategies (Section 5.2). We explore incremental learning as a possible technical breakthrough direction for graph structure discovery. We add a case study demonstrating the full chain of capabilities for anomaly identification, diagnosis, and explanation (Section 4.6.5).
- We strengthen methodological innovation by detailing the dynamic threshold mechanism of the SSA algorithm (Section 4.3). We add a natural structural convergence/stability quantification analysis (Section 4.6.2). We compare the threshold determination methods of seven comparison models (Table 3). We reveal unknown anomaly types through geometric pattern mining (Section 4.6.4).

1. The manuscript's formatting is inconsistent, particularly in the alignment of equations, headings, and figures. Standardizing these elements would significantly enhance readability and professionalism.

**Response 1:** Thank you very much for your valuable feedback. Regarding the issue of uniformity in manuscript formatting, we have conducted a comprehensive and thorough review and revision, focusing on the alignment of formulas, titles, and charts to ensure consistent and standardized formatting throughout the document. We have adjusted all mathematical formulas to the standard centered format, and we have strictly aligned charts and their accompanying text. Additionally, we have supplemented and improved the detailed legends for all charts to make the relationship between text and images clearer and more explicit. The following improvements have been made:

**Revision 1:** **[(Page 1) 1. Introduction]** We have deepened our understanding of the core tasks of anomaly detection in multivariate anomaly detection, namely anomaly identification, anomaly diagnosis, and anomaly interpretation, and provided a detailed explanation in Fig.1 to enrich the reader's understanding of these tasks.

**[(Page 5 $\sim$ 8) 2.2 Graph Structure Discovery and Learning]** Given that there are many points of confusion between graph structure learning and the natural structure discovery process we are researching, We add Fig.3 to explain the differences between graph structure discovery and graph structure learning, clearly defining the boundaries between the graph structure discovery method we are using and the graph structure learning framework. We highlight the uniqueness of our research method.

**[(Page 11 $\sim$ 12) 3. HAO Model]** Given the lack of theoretical exposition for Euclidean space $\mathcal{E}$ and Poincaré sphere space $\mathcal{P}$ in Version 1, we have restructured the theoretical part of the HAO model. We have provided a more complete and concise theoretical representation of the three core components in Version 1: hyperbolic linear transformation, hyperbolic graph convolution layer, and hyperbolic graph structure discovery, thereby reducing the difficulty of understanding the synergistic effects between these components.

**[(Page 21 $\sim$ 22) 4.1 Dataset]** We expand the anomaly detection dataset in Version 1 to cover 16 different application scenarios, including industrial control, IT operations, critical infrastructure, cybersecurity, scientific observation, as detailed in Table 1. In the experimental setup section, we reorganize the descriptions of the comparison models and further expand on the similarities and differences in their framework designs. We also detail the threshold determination methods and anomaly explanation methods for all comparison models to facilitate a multi-faceted comparison with our HAO model.

**[(Page 11 $\sim$ 20) 3. HAO Model, 4.2 Evaluation Metrics, 4.3 Experimental Setup]** We adopt a standardized formatting style for mathematical formulas, using centered alignment and separate lines to ensure consistency and readability.

2. Some sections, especially those explaining hyperbolic transformations and graph structure learning, use overly complex sentences. Simplifying these explanations would make the content accessible to a broader audience.

**Response 2:** We are very grateful to the reviewers for pointing out that some sentences in the theoretical part of the HAO model in Version 1 were too complex. We make significant changes to the content and expression of the HAO theory section. By reasonably organizing and expanding the content of the theoretical section and simplifying the description of the core component theory, we improve the overall integrity, rationality, and interpretability of the HAO model framework design.

**Revision 2:** **[(Page 11$\sim$20) 3. HAO Model]** We made the following improvements to the theoretical part of the HAO model framework:

- We simplify the overall description of the HAO model framework, focusing on explaining the HAO model framework design method and the challenges it addresses. In the explanatory section of Fig.7, we briefly describe the execution process of the HAO model framework and the coordination process between components. This enhances the reader's understanding of the overall process of the HAO model in anomaly detection tasks (Sections 3.0 and 3.1).
- The initial embedding part of multivariate data expands the mathematical definitions of distance measures, spatial mappings, and translations in the three geometric embedding representations of the HAO model in Euclidean space $\mathcal{E}$, Poincaré sphere space $\mathcal{P}$, and hyperbolic space $\mathcal{H}$, laying the foundation for the subsequent design of HAO model components (Section 3.2).
- We extend related theorems and lemmas to provide theoretical support for the discovery of natural structures in multivariable data spatio-temporal, component design, and HAO model optimization processes (Section 3.3).
- We simplify the theoretical explanations of the hyperbolic linear transformation, hyperbolic attention graph convolution layer, multivariate spatio-temporal graph construction and constrained initialization, adaptive hyperbolic graph structure discovery, joint optimization objective, and anomaly detection sections involved in the hyperbolic pre-training framework. We focus on the practical considerations of component design and core theoretical formula expressions (Section 3.4).

3. While figures such as the framework diagram (Figure 4) and hyperbolic space illustrations (Figure 5) are informative, they could benefit from higher resolution and more detailed captions to improve interpretability.

**Response 3:** We express our gratitude to the reviewers for their valuable comments on the inadequacies in the presentation of Fig.4 (HAO model framework) and Fig.5 (hyperbolic space diagram). To optimize the article's structure, we adjust the content layout accordingly. We move Fig.5 to Fig.4's position and Fig.4 to Fig.7's position to achieve a more reasonable content arrangement. We make the following improvements:

- For Fig.4, we redesigned the schematic diagram of data embedded in hyperbolic space under different curvatures and adjusted the presentation position of data point projections. We add annotations for different curvatures to more intuitively demonstrate the geometric advantages of hyperbolic space in capturing complex relationships and sensitive anomalies.
- For Fig.7, we logically organize and visually optimize the overall process of the HAO model, strengthen the explanation of key steps, and unify the identification specifications. This makes the operational logic of the model framework clearer and easier for readers to understand.

**Revision 3:** **[(Page 8) 2.2.4 Hyperbolic Graph Representation Learning]**

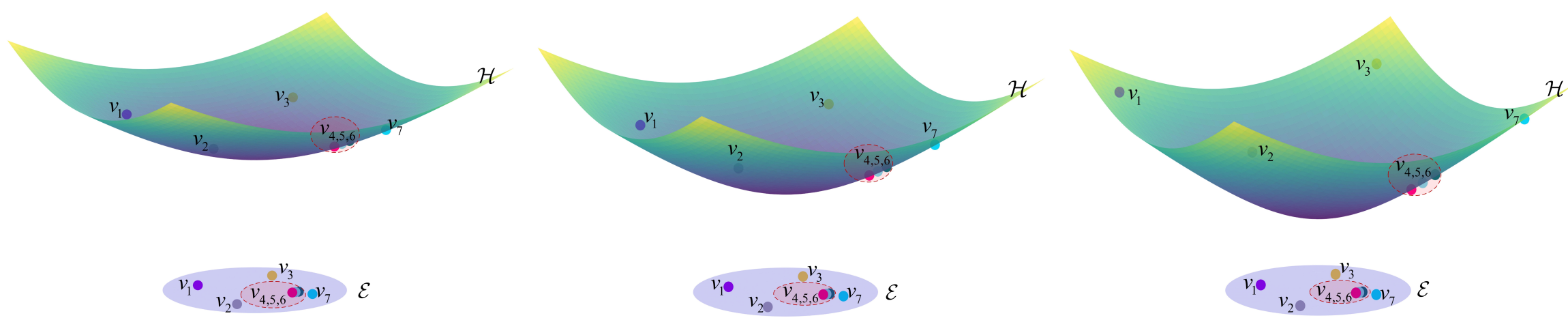

(a) $K = -1$ (b) $K = -2$ (c) $K = -4$

Figure 1: This paper presents the embedding representation of data points in hyperbolic spaces with different curvatures. In Euclidean space, the relationships between data points (such as $v_4$, $v_5$, $v_6$) are less distinguishable and exhibit strong clustering (or community structure) [1]. However, when mapped to hyperbolic space, as the absolute value of the curvature of the embedding space gradually increases (corresponding to subgraphs (a) $K = -1$, (b) $K = -2$, and (c) $K = -4$), the geometric distances between these data points significantly increase. This indicates that the distinguishability of their intrinsic relationships is improving overall. The enhanced distinguishability of these key relationships (especially anomalous relationships) not only enables more effective identification of weak anomaly types but, more importantly, provides more refined and robust evidence for revealing the causes of unknown anomaly types.

**[(Page 11 ∼ 12) 3. HAO Model]** The data geometry-driven hyperbolic adaptive spatial-aware multivariate time series anomaly detection model proposed in this paper (Fig.2) is a multi-task learning framework that unifies data reconstruction, structure discovery, and geometric parameter learning, aiming to address the following challenges:

(1) Reduce the rigidity of the predefined structure constraints. By applying physical constraints to different dimensions of the data and using more relaxed constraint conditions, we can minimize the interference of human prior knowledge on the predefined structure.

(2) Reduce data topology attribute distortion by optimizing the learning framework through a dual adaptive hyperbolic graph structure, multiple data representation spaces, adaptive curvature adjustment, and dynamic structural optimization to maximize the exploration of structural characteristics suitable for the data.

(3) Reduce confusion in explaining abnormal causes by optimizing the dual-channel structure mechanism, independently optimizing the temporal dependency relationships and multivariable coupling relationships of multivariable data, and improving the reliability of interpretation for different abnormal causes.

(4) Improve the efficiency of dynamic structure optimization. Through customized learning components, we can maximize the representation performance of different data representation spaces, reduce accuracy loss in feature space conversion, and enhance the efficiency of multivariate anomaly detection.

The HAO model incorporates several key components of the innovative design described in this paper, including adaptive hyperbolic space dynamic adjustment of data embedding quality, adaptive hyperbolic graph structure discovery to dynamically capture the natural structure of data, a small sample dynamic mixed anomaly threshold determination algorithm to improve the robustness of anomaly detection, and an efficient graph structure difference analysis strategies to efficiently explain the causes of anomalies across regions.

## References


[1] Ranshous, S. et al. Anomaly detection in dynamic networks: a survey. *Wires. Comput. Stat.* **7**, 223–247 (2015).


4. While the method demonstrates strong performance, the paper could include a discussion on scalability for large-scale or dynamic datasets and its real-time deployment feasibility.

**Response 4:** We thank the reviewers for their valuable comments on the scalability of the HAO model on large-scale/dynamic datasets and the feasibility of real-time deployment. We make the following improvements:

- We collect datasets ranging from thousands to millions of scales for comparative testing (see Section 4.4.4, "Analysis of Multi-scenario Adaptability and Spatio-temporal Complexity"). The experimental results show that the HAO model demonstrates good performance potential on large-scale datasets. At the same time, in terms of real-time deployment feasibility, the model exhibits high execution efficiency and low memory consumption, meeting basic deployment requirements.
- We conduct a theoretical discussion in response to reviewers' concerns about larger-scale sensor scenarios. The HAO model aims to reveal the underlying structure of the system, uncover intrinsic relationships within information systems, and capture spatio-temporal patterns in dynamic environments. Its working principle

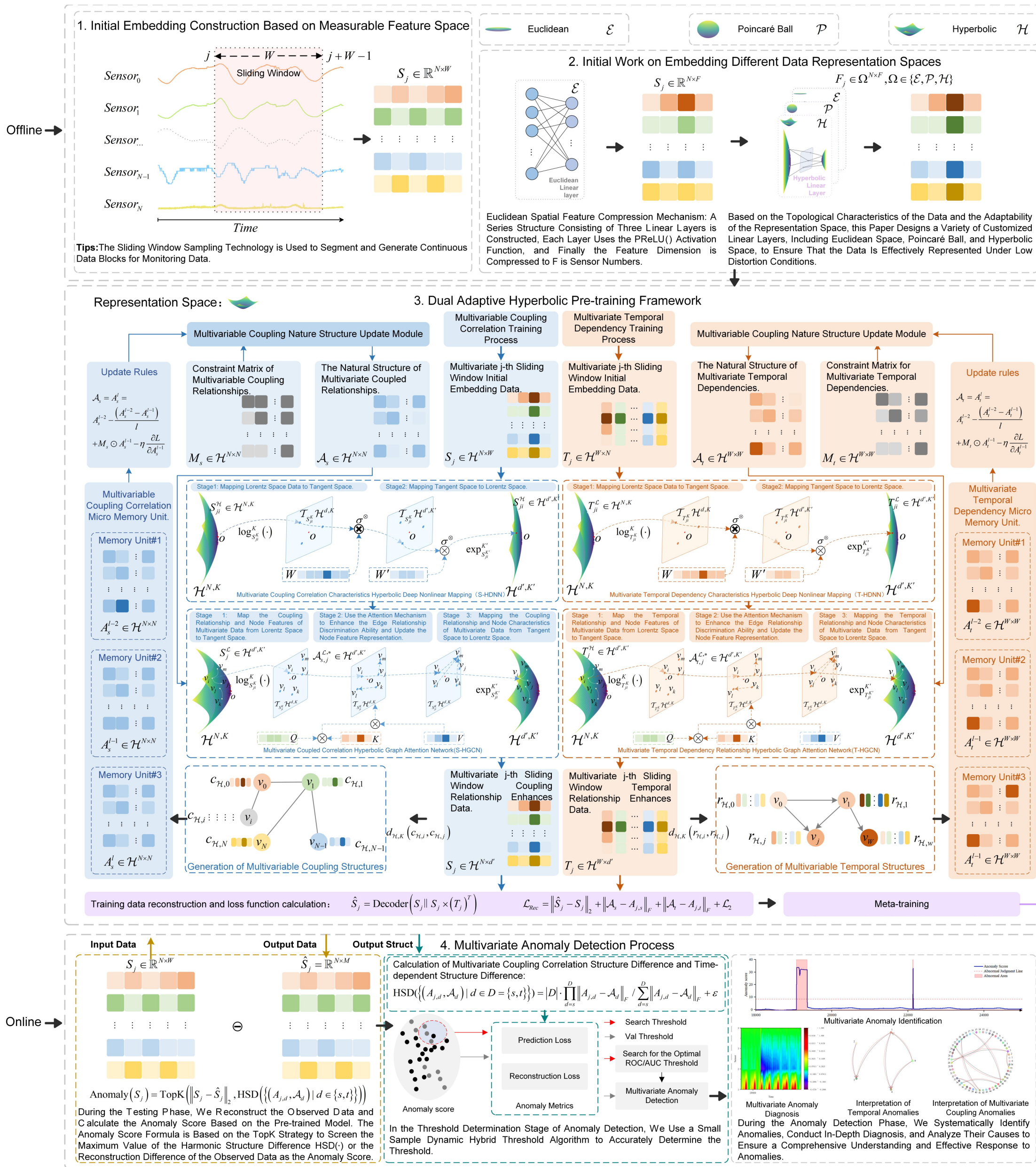


Figure 2: The HAO model achieves multivariable anomaly detection through a four-stage process. Taking hyperbolic space($\mathcal{H}$) embedding as an example: first, initial feature embedding is performed, where input features $S_j$ are generated by continuously sampling from the time-series data collected from the monitored object using a sliding window $W$; then, the data representation space is selected, where $S_j$ is input into a Euclidean space linear layer to enhance information density, and $\mathcal{E}$ (Euclidean space), $\mathcal{P}$ (Poincaré sphere space), or $\mathcal{H}$ (hyperbolic space) are chosen for feature initialization. The core stage is the adaptive hyperbolic pre-training framework (AH-PF): (1) We construct a multivariate coupling correlation matrix $\mathcal{A}_s$ and a temporal dependency matrix $\mathcal{A}_t$ in hyperbolic space, and fuse the physical constraint matrices $M_s$ and $M_t$ with the features $S_j$ and $T_j$ to form pre-training inputs; (2) We achieve curvature-adaptive representation enhancement through parallel hyperbolic deep linear transformation (HDNN) and graph attention neighbor aggregation layer (HGCN), dynamically adjusting curvature to optimize coupling and temporal embedding quality; (3) In the hyperbolic graph structure discovery stage, we fuse hierarchical and neighborhood structure generation to enhance the graph spectrum, introduce coupling/temporal dependency-based micro-memory units to suppress numerical mutations, and enhance the generalization capability of the interpretability dimension based on robust update rules; (4) We adopt a meta-training strategy, fine-tune based on data block topological features to maintain the stability of $\mathcal{A}_s$ and $\mathcal{A}_t$. In the final multivariate anomaly detection stage, the data to be inspected is processed by the AH-PF framework, and the natural structure of multiple interpretability dimensions is aggregated to generate prediction variants. An anomaly score is calculated using a dynamic threshold mixing algorithm and a joint loss function (reconstruction loss and harmonic structural difference loss) to complete anomaly identification, diagnosis, and root cause analysis. This framework significantly improves the multidimensional anomaly analysis capabilities of complex systems through spatial adaptive selection and hyperbolic curvature adjustment mechanisms.

requires analyzing the mutual influence between arbitrary sensors without prior knowledge. Based on this, the number of sensors and the spatial complexity required by the model theoretically grow linearly. It should be noted that while graph representation learning has made progress in predicting million-node links [1], this is often based on known topological structures. In contrast, real-world graph structure discovery typically lacks such prior knowledge. In cases of uncertain topological information, the node sampling strategy for link prediction introduces additional noise, which typically increases the memory resource requirements for graph

structure discovery tasks. Therefore, incremental learning of natural graph structures may be an important direction for future research.

## References

[1] Xu, Y. Y. et al. Scalable and effective temporal graph representation learning with hyperbolic geometry. *IEEE T. Neural Netw. Learn. Syst.* (2024).

**Revision 4: [(Page 21 ∼ 23) 4.1 Dataset]** The experimental analysis is based on a large-scale dynamic dataset covering thousands to millions of scales and different application scenarios.

**[(Page 32 ∼ 33) 4.4.4 Analysis of Multi-scenario Adaptability and Spatio-temporal Complexity]** We experimentally evaluate the adaptability, time complexity, and space complexity of different models when processing multi-scenario dynamic datasets, with test ranges covering data scales from thousands to millions (see Fig.13 for details).

**[(Page 52 ) 5.2 Discussion]** We conduct a supplementary discussion on the potential technical bottlenecks that may exist in the natural graph structure of the HAO model.

5. The framework is highly specialized for multivariate anomaly detection but could benefit from a discussion on potential applications in other domains, such as finance, healthcare, or cybersecurity.

**Response 5:** Thank you for suggesting that we explore the potential applications of the HAO model framework in other fields. The core of the HAO model framework lies in the discovery of system-natural structures. Our work focuses on utilizing these natural structures for anomaly detection, diagnosis, and cause explanation. Therefore, we are also interested in the potential value of intrinsic structures in other application areas, such as multivariate intrinsic structures and domain transfer, geometric properties of temporal dependencies, network intrusion target selection, and robust assessment of system operational states. Regarding these research topics, we provide a thorough discussion in the "Multivariate Anomaly Causality Interpretation" section of the main text.

**Revision 5: [(Page 42 ∼ 51) 4.6.2 Analysis of Multivariate Spatio-temporal natural Structure Characteristics, 4.6.3 Multivariate Natural Structure and Domain Transfer Analysis, 4.6.4 Multivariate Anomaly Causality Interpretation Graph, 4.6.5 Case Study Analysis]** To gain a deeper understanding of anomalies, we distill the complex and diverse anomaly phenomena into two core forms: time-series dependency anomalies and variable coupling correlation anomalies. The key value of the HAO model lies in its ability to automatically reveal the intrinsic natural structure of information systems, laying the analytical foundation for tracing the causes of these anomalies. Building on this, we utilize visualization analysis to deeply examine the three key characteristics of multivariate data: first, the convergence and stability of the intrinsic structure; second, the effectiveness and discernibility of hierarchical and community structures; third, empirical evidence of the universality of the geometric properties of the intrinsic structure and its domain transferability. Our goal is to uncover and interpret more potential, unknown, and complex abnormal geometric patterns through this series of analyses.

6. The conclusion mentions potential improvements but lacks specific directions. Elaborating on extensions, such as integrating reinforcement learning or exploring domain adaptation techniques, would provide a clearer research trajectory.

**Response 6:** Thank you very much for your valuable suggestions on domain-adaptive techniques in natural structure discovery. Based on your feedback, we expand the summary section, adjusting it from a single summary in Version 1 to the current two-part summary and discussion. In the summary, we focus on the key contributions of the HAO model; in the discussion, we explore several potential research directions that deserve attention in the future.

**Revision 6 : [(Page 51 ∼ 52) 5. Summary and Discussion]** 5.1 In the summary section, we further clarify the key contributions and performance of the HAO model based on the original work while incorporating a detailed discussion of the complete anomaly detection task. In the discussion section, we focus on future research, exploring several promising directions worth exploring, including model efficiency and real-time performance, interpretability of complex anomaly patterns, generalization under extreme small-sample and zero-sample conditions, and validation of domain transfer and generalization capabilities.

7. While the manuscript uses standard metrics (AUC, F1, etc.), including additional metrics like precision-recall curves for imbalanced datasets would enrich the evaluation.

**Response 7:** Thank you very much for your suggestions regarding the performance evaluation of the HAO model. We supplement Section 4.4 on multivariate anomaly detection with precision, recall, F1 score, ROC/AUC curve, and

P-R/AP curve [1] to evaluate the performance and generalization ability of the comparison model and the HAO model from a richer and more comprehensive perspective.

## References

[1] Powers, D.M.W. Evaluation: from precision, recall and F-measure to ROC, informedness, markedness & correlation. *J. Mach. Learn. Technol.* **2**, 37–63 (2011).

**Revision 7: [(Page 28 ∼ 35) 4.4.1 Analysis of Anomaly Identification Performance Based on ROC Curves and AUC Metrics, 4.4.2 Analysis of Anomaly Detection Performance Based on P-R Curves and Average Precision, 4.4.3 Analysis of Comparative Experimental Results, 4.4.5 Analysis of Multi-scenario Adaptability and Spatio-temporal Complexity]** To conduct a more comprehensive evaluation of the HAO model's performance, we add new subsections in Section 4.4: 4.4.1 provides an in-depth analysis of anomaly detection performance based on ROC curves and AUC metrics; Section 4.4.2 explores anomaly detection performance based on P-R curves and average precision; Section 4.4.3 presents the results of comparative experiments; Section 4.4.5 examines the model's multi-scenario adaptability and spatio-temporal complexity. These supplementary, multi-perspective evaluation experiments effectively validate the HAO model's reliable identification capabilities in multivariable anomaly detection tasks.

8. A more detailed discussion of the datasets, including their challenges and how the proposed model addresses them, would provide better context for the results.

**Response 8:** Thank you for your valuable suggestions regarding the challenges faced by anomaly detection datasets and the HAO model's response strategies. As is pointed out, the core challenges of anomaly detection datasets lie in the lack of standardized anomaly evaluation benchmarks and the absence of systematic summarization and generalization of the underlying causes of anomalies. We first conduct an initial classification of the datasets involved in this paper using the anomaly classification method proposed by [1]. Building on this, for the exploration of the underlying causes of anomalies, we innovatively adopt a method that analyzes the differences between the original structure and the predicted structure for explanation. Based on this, we make the following revisions:

- To improve the robustness of the HAO model to diverse anomalies, we expand the evaluation dataset from the original 6 to 16, ensuring broader application scenario coverage.
- Following the classification criteria of [1], we conduct a systematic analysis of 16 test datasets, elaborating on the univariate/multivariate point-level, regional, and sequence anomaly types present in them and discussing their inherent challenges (see Fig.8 and Fig.9 for specific analyses).
- Through comparative experiments (see Table 3), we evaluate the performance of the HAO model and the baseline model in handling the above three types of anomalies. Empirical results show that the HAO model's detection strategy based on data geometry not only significantly improves the recognition rate of point-level, regional, and sequence anomalies but also can discover potential anomaly patterns and geometric features. This strongly demonstrates the comprehensive advantages and leading position of the HAO model in the three key areas of anomaly recognition (Section 4.4), anomaly diagnosis (Section 4.5), and anomaly causality interpretation(Section 4.6).

## References

[1] Gen Li, Jason J. Jung, Deep learning for anomaly detection in multivariate time series: Approaches, applications, and challenges, Information Fusion, Volume 91, Pages 93-102, 2023.

**Revision 8: [(Page 21 ∼ 51) 4. Experiment]** We verify the superior performance of the HAO model in three experimental parts based on the data geometry - driven anomaly detection scheme: anomaly identification (Section 4.4), anomaly diagnosis (Section 4.5), and anomaly causality interpretation (Section 4.6).

9. The Small Sample Dynamic Mixed Threshold Determination Algorithm (SSA) is a key innovation but is described briefly. Expanding on its implementation and parameter sensitivity would strengthen its contribution.

**Response 9:** Thank you very much for recognizing the Small Sample Dynamic Hybrid Threshold Determination Algorithm (SSA). In response to your suggestions, we make the following modifications: first, we categorize SSA under the experimental settings section; second, we expand the data construction scheme for replacing large samples with small samples; and third, we optimize the efficiency of the SSA search process. To more comprehensively validate the performance of SSA, we expand the scope of comparison in our experiments. We divide data into sequences at ratios

ranging from 0.1 to 0.9 across 16 benchmark datasets and conduct threshold searches while also evaluating performance under short, medium, and long sliding windows ($W = \{5, 10, 30, 90, 200, 500\}$). After calculating the average $\overline{F1}$ scores for all window sizes and partition ratios, we find significant differences in anomaly detection performance across different embedding spaces. These improvements and subsequent experiments strongly validate the effectiveness of the SSA anomaly threshold determination algorithm.

**Revision 9:** **[(Page 25 $\sim$ 28) 4.3 Experimental Setup]** We provide detailed supplementary explanations on the specific implementation methods of the small sample dynamic mixed threshold determination algorithm.

**[(Page 33 $\sim$ 35) 4.4.6 Hyperparameter Experiments of the HAO Model]** In order to more comprehensively evaluate the small sample dynamic mixed anomaly threshold determination algorithm, we expand and improve its statistical characteristics and design and implement experiments to analyze the sensitivity of anomaly detection to data sample segmentation ratios. The experimental results not only verified the effectiveness of this algorithm in most anomaly detection scenarios.

## Reviewer #2 (Remarks to the Author):

The multivariate anomaly detection is an interesting research problem and has great application value. The paper introduces hyperbolic graph learning into hierarchical structure mining of multivariate data and proposes a complete multivariate anomaly detection framework for detection accuracy, interpretability, and robustness. Extensive experiments are conducted to verify the effectiveness and efficiency of this framework. But the paper still exists the following important problems:

**Response :** We sincerely appreciate your valuable feedback on the application of hyperbolic graph learning to the mining of multi-dimensional data hierarchies. In particular, your observation that feature reconstruction optimization may not effectively promote feature adaptation between adjacent structures and hyperbolic structures prompts us to re-evaluate the implementation of hyperbolic graph structure learning in the HAO model. We recognize that to enhance interpretability, the design of spatio-temporal structures should be separated from the feature reconstruction process. Accordingly, we restructure the hyperbolic graph structure discovery module of the HAO model to enhance the transparency of natural structure learning and ensure accurate identification of the inherent geometric characteristics of multivariate data, including community and hierarchical properties. The specific details of this significant revision are as follows:

- We supplement the related work section and emphasize the most significant difference between our work and existing methods—directly mining the system's natural topological attributes and explaining the causes of anomalies in an unsupervised framework. The related work section is now complete and provides comparison with existing methods.
- We review the development of graph structure learning, clearly define the difference between graph structure discovery and graph structure learning, and explain the background of hyperbolic graph representation learning and its intrinsic connection to our work. The relevant section lacked an overview of research on hyperbolic graph learning.
- We correct the concept of AH-GSL to "hyperbolic adaptive graph structure discovery" and deeply restructure the model framework, including lightweight framework design, definition of functional component generality across different embedding spaces, and validation of the validity of spatio-temporal relationship structures. We also supplement it with theoretical analysis and experimental verification.
- We significantly restructure the framework of the HAO model and design three complementary experimental sections centered on the core task of anomaly detection: anomaly identification, anomaly diagnosis, and anomaly causality interpretation. This greatly increases the assessment dimensions and optimizes the visualization of the results.
- We supplement the SWaT dataset with case studies, analyzing network attack incidents in detail and demonstrating the ability of the HAO model's modules to work together and provide in-depth explanations of the causes of anomalies.

In addition, we thoroughly review the entire text, corrected errors in expression, and supplement the data set and code acquisition methods to ensure the transparency and reproducibility of the research.

1. Figure 1 does not provide valuable information and should be revised to show a comprehensible diagram.

**Response 1:** Thank you for pointing out that Fig.1 was not effectively conveying the information. We completely redraw Fig.1 based on your suggestions. The new figure aims to clearly illustrate the full scope of the multivariable anomaly detection task, including the three key stages of anomaly identification, diagnosis, and root cause analysis, along with their primary characteristics. Most importantly, in the section on anomaly root cause interpretation, we not only critically examine the shortcomings of existing methods but also delve into their fundamental obstacles: namely, the true topological properties of the observed objects are often unknown, which makes constructing a reliable benchmark framework for anomaly root cause interpretation mechanisms a core challenge.

**Revision 1:** **[(Page 1) 1.0 Introduction]** With the continuous advancement of industrial IoT and information systems, production monitoring sensor [1] plays a key role in data collection and analysis in the production process, which is crucial for ensuring production safety and enhancing intelligent operation and maintenance [2]. The existing multivariate anomaly detection research task (Fig.3) in [3, 4, 5] still faces challenges such as the unknown true topology of the system and distortion of the data embedding space [6], confusing anomaly interpretation dimensions, and the dynamic scaling of sensor scale. In addition, the "interpretation comfort zone trap" of over-reliance on structural a priori severely limits the anomaly interpretation capability of dynamic scenarios [7]. Therefore, we aims to construct an open and adaptive explanation generalization mechanism. This mechanism aims to maintain explanation credibility and traceability accuracy when unknown anomalies emerge. It is not only an important exploration to realize industrial cognitive

intelligence from "anomaly recognition" to "causal insight" but also concerns the credibility of decision-making under complex working conditions.

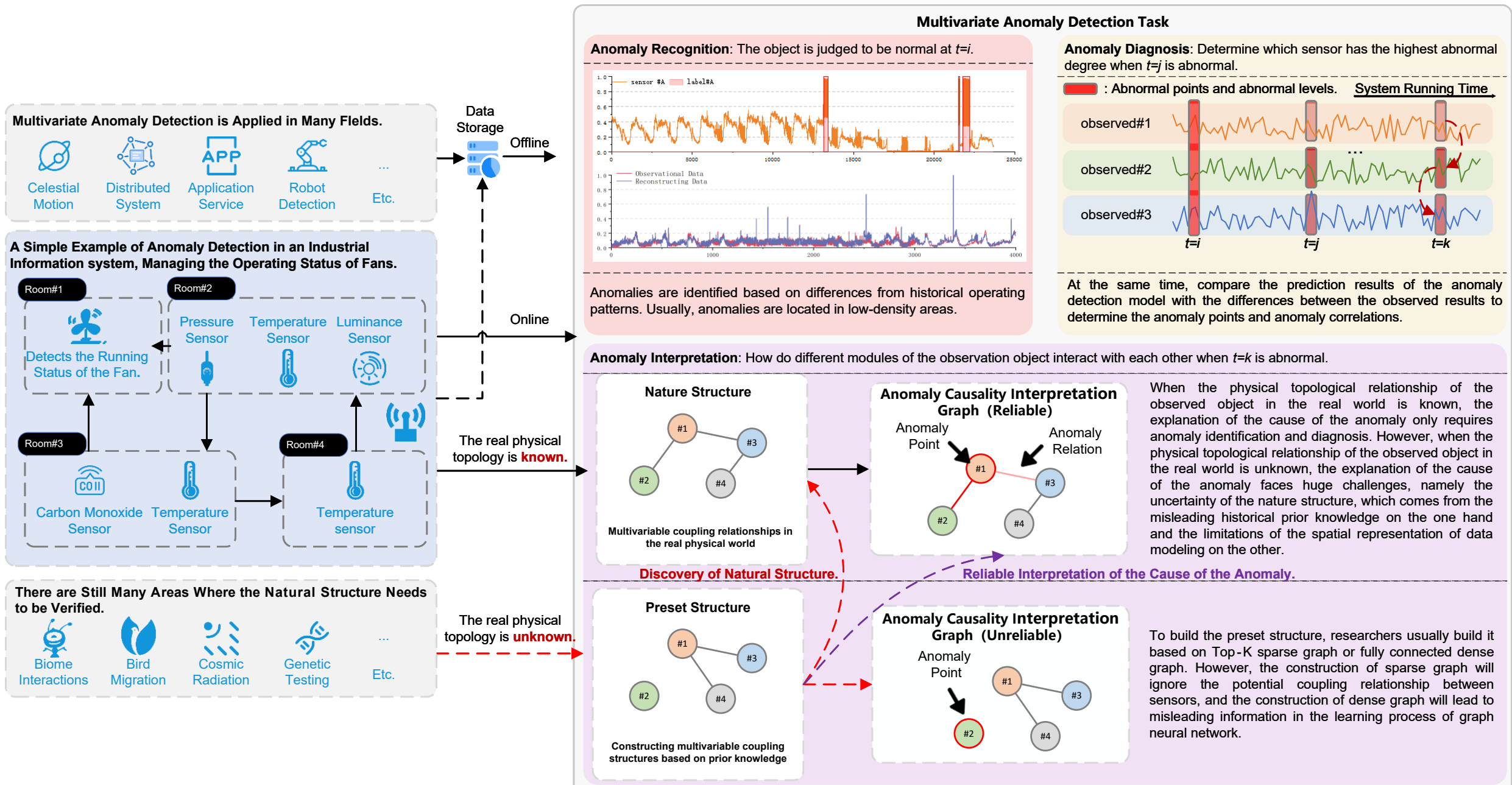


Figure 3: Currently, multivariate anomaly detection has evolved into a three-tiered detection framework [4]: At the detection layer (Anomaly Recognition), anomaly detection uses advanced pattern recognition algorithms to screen for anomalous segments from massive amounts of time series data; at the diagnosis layer (Anomaly Diagnosis), spatio-temporal dynamic relationship analysis is used to precisely locate the spatio-temporal coordinates and threshold conditions where anomalies occur; at the interpretation layer (Anomaly Interpretation), causal reasoning and knowledge graphs reveal the multi-level causal network behind anomalies. This three-tiered paradigm of "detection-diagnosis-interpretation" not only achieves a leap from observing phenomena to understanding their essence but also provides an actionable decision-making basis for continuously optimising production systems.

## References


[1] Sloane, M. et al. Materiality and risk in the age of pervasive AI sensors. *Nat. Mach. Intell.* **7**, 334–345 (2025).

[2] Tao, F., Zhang, H. & Zhang, C. Advancements and challenges of digital twins in industry. *Nat. Comput. Sci.* **4**, 169–177 (2024).

[3] Pazho, A. D. et al. A survey of graph-based deep learning for anomaly detection in distributed systems. *IEEE T. Knowl. Data En.* **36**, 1–20 (2024). https://doi.org/10.1109/TKDE.2023.3282898

[4] Li, G. & Jung, J. J. Deep learning for anomaly detection in multivariate time series: approaches, applications, and challenges. *Inform. Fusion.* **91**, 93–102 (2023).

[5] Ranshous, S. et al. Anomaly detection in dynamic networks: a survey. *Wires. Comput. Stat.* **7**, 223–247 (2015).

[6] Peng, W., Varanka, T., Mostafa, A., Shi, H. & Zhao, G. Hyperbolic deep neural networks: a survey. *IEEE. T. Pattern. Anal.* **44**, 10023–10044 (2022).

[7] Abhilash, P. M. et al. Towards next-gen smart manufacturing systems: the explainability revolution. *npj Adv. Manuf.* **1**, 8 (2024).


2. The motivation of the work is not explicit. The paper does not clearly explain why the multivariate anomaly detection needs hyperbolic space.

**Response 2:** Thank you for your valuable feedback, which pointes out that the paper was not sufficiently clear in explaining the research motivation and the necessity of applying hyperbolic space. We carefully reflect on these shortcomings and make targeted revisions. Therefore, in the first paragraph of Section 1.0 Introduction, we reorganize and strengthen the statement of research motivation, clearly stating that this study aims to construct an open and adaptive explanatory generalization mechanism. The core value of this mechanism lies in its ability to ensure the credibility of explanations and the accuracy of traceability even in the face of emerging unknown anomalies. We believe this is not

only a crucial step in advancing industrial cognitive intelligence from "anomaly detection" to "causal insight" but also a necessary pathway to enhance the reliability of decision-making in complex operational conditions.

Revision 2: **[(Page 1) 1.0 Introduction]** With the continuous advancement of industrial IoT and information systems, production monitoring sensor [1] plays a key role in data collection and analysis in the production process, which is crucial for ensuring production safety and enhancing intelligent operation and maintenance [2]. The existing multivariate anomaly detection research task (Fig.1) in [3, 4, 5] still faces challenges such as the unknown true topology of the system and distortion of the data embedding space [6], confusing anomaly interpretation dimensions, and the dynamic scaling of sensor scale. In addition, the “interpretation comfort zone trap” of over-reliance on structural a priori severely limits the anomaly interpretation capability of dynamic scenarios [7]. **Therefore, we aims to construct an open and adaptive explanation generalization mechanism. This mechanism aims to maintain explanation credibility and traceability accuracy when unknown anomalies emerge. It is not only an important exploration to realize industrial cognitive intelligence from “anomaly recognition” to “causal insight” but also concerns the credibility of decision-making under complex working conditions.**

## References


[1] Sloane, M. et al. Materiality and risk in the age of pervasive AI sensors. *Nat. Mach. Intell.* **7**, 334–345 (2025).

[2] Tao, F., Zhang, H. & Zhang, C. Advancements and challenges of digital twins in industry. *Nat. Comput. Sci.* **4**, 169–177 (2024).

[3] Pazho, A. D. et al. A survey of graph-based deep learning for anomaly detection in distributed systems. *IEEE T. Knowl. Data En.* **36**, 1–20 (2024). https://doi.org/10.1109/TKDE.2023.3282898

[4] Li, G. & Jung, J. J. Deep learning for anomaly detection in multivariate time series: approaches, applications, and challenges. *Inform. Fusion.* **91**, 93–102 (2023).

[5] Ranshous, S. et al. Anomaly detection in dynamic networks: a survey. *Wires. Comput. Stat.* **7**, 223–247 (2015).

[6] Peng, W., Varanka, T., Mostafa, A., Shi, H. & Zhao, G. Hyperbolic deep neural networks: a survey. *IEEE. T. Pattern. Anal.* **44**, 10023–10044 (2022).

[7] Abhilash, P. M. et al. Towards next-gen smart manufacturing systems: the explainability revolution. *npj Adv. Manuf.* **1**, 8 (2024).


3. In the introduction section, the paper should discuss the direct challenges of multivariate anomaly detection instead of focusing on natural data structures analysis.

**Response 3:** Thank you for your valuable feedback, which highlights the need for the introduction to focus on the direct challenges of multi - class anomaly detection rather than being limited to the analysis of natural data structures. We acknowledge that while understanding the foundational aspects of data structures is important, articulating the direct challenges faced is more effective in highlighting the necessity of the research. Given the core objective of this study - to develop a more generalized mechanism for explaining the causes of multivariate anomalies (**Response 2** ) - which heavily relies on reliable natural structure discovery, we emphasize the direct challenges faced in multivariable anomaly detection in Section 1.0 of the introduction to better establish the research foundation.

Revision 3: **[(Page 2 $\sim$ 3) 1.0 Introduction] Challenge 1: The true topology of the system is unknown.** In an open environment, the lack of explicit optimization objectives for real system topologies and supervision by domain experts makes it difficult to establish a system natural structure that conforms to the essence of industrial scenarios. This issue primarily manifests in static models built based on prior assumptions (e.g., $\epsilon$-radius, $k$-nearest neighbors) [2, 3] struggling to effectively adapt to data-natural topologies (such as nonlinear dependencies and time-varying relationships), resulting in elevated false positive rates and interpretive biases. Although some studies have adjusted parameters through online learning [4], the underlying topological structure remains rigid, failing to achieve a truly “data-adaptive structure.” These limitations are particularly pronounced in non-stationary data streams (e.g., progressive degradation monitoring of devices [5]) and systems with dense latent variables (e.g., detection of misinformation in social networks [6]). In recent years, researchers have explored various aspects such as temporal dependencies in multivariate data, sensor coupling, and heuristic learning [2, 4, 7, 8], and have developed adaptive topological dynamic evolution models. However, these models heavily rely on the true topological relationships of the system or a large amount of supervised information (such as nodes, edges, and graph features [9, 10, 11]) as explicit optimization objectives. The potential constraints imposed by prior knowledge as out the implicit structure of the data rather than truly adapting to the data structure, which makes it difficult to identify structural anomalies in dynamic scenarios effectively. The limitations of this type of research, especially in multivariate anomaly detection, are that the available topological optimization information is particularly scarce, prompting us to conduct research on the discovery of the natural structure of multivariate data without prior

knowledge constraints. We proposed an adaptive hyperbolic graph structure discovery method, providing a reliable reference benchmark and objective evidence to support the explanation of the causes of multivariate anomalies.

**Challenge 2: Data embedding space distortion.** The system's natural structure discovery is constrained by geometric distortion in the data representation space [1]. This distortion makes it difficult to effectively identify weak abnormal relationships. This phenomenon is detailed in Fig.4. Early research successfully analyzed structural features in physical systems using complex network theory [12, 13, 14], achieving significant breakthroughs in scenarios with explicit entity relationships, such as epidemic spread and computer virus propagation [15, 16, 17]. However, when the true system topology is unavailable, such network models based on physical assumptions are challenged in their applicability. In the field of multivariate anomaly detection, strategies such as stacking multi-head attention modules or deepening temporal networks [7, 18, 19] have been employed to enhance the ability to detect weak anomalies and model structural features. However, these approaches not only increase the computational burden of the model but also fail to address the fundamental bottleneck in feature representation. Meanwhile, in the field of graph representation learning [20], data-driven methods capture latent structural features, but Euclidean space embedding leads to severe geometric distortion. This distortion significantly reduces the discernibility of node relationships. Therefore, researchers have leveraged the superior geometric representation capabilities of hyperbolic space [21, 22] to improve data embedding quality. However, graph representation learning relies on network data with known topological relationships. These research boundaries prompt us to consider how to match the geometric characteristics of hyperbolic space with node neighborhood structures. This consideration leads to the proposal of an adaptive hyperbolic pre-training framework that significantly improves the ability to identify weak anomalies.

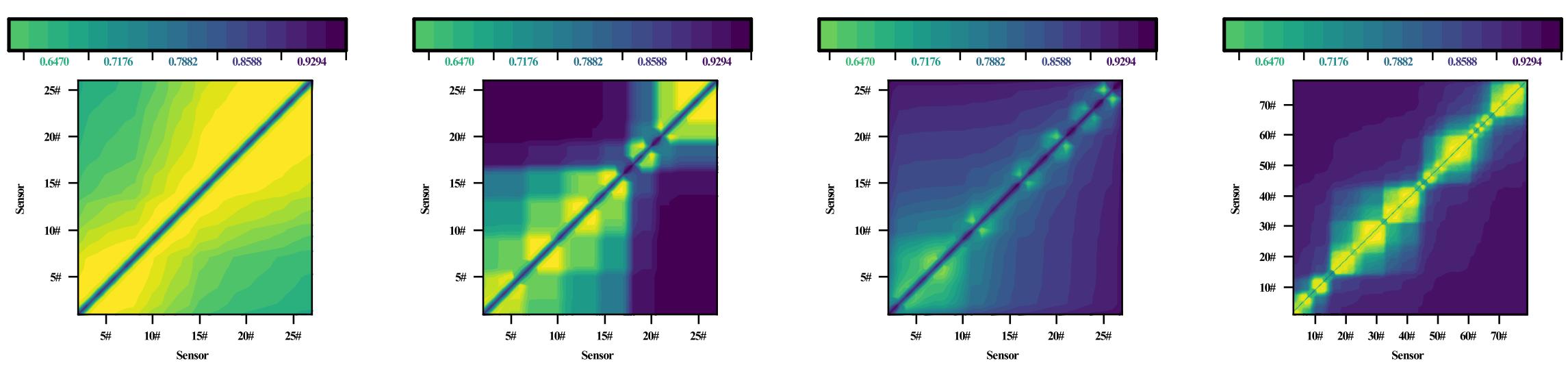


(a) The Natural Structure of the Application Server of the Our Model in $\mathcal{E}$ Mining

(b) The Natural Structure of the Application Server of the Our Model in $\mathcal{P}$ Mining

(c) The Natural Structure of the Application Server of the Our Model in $\mathcal{H}$ Mining

(d) The Natural Structure of Our Model for the Network Intrusion Detection System[23] in $\mathcal{E}$ Mining

Figure 4: By comparing the performance of our model in different data embedding spaces (Euclidean space $\mathcal{E}$, Poincaré sphere space $\mathcal{P}$, and hyperbolic space $\mathcal{H}$), we observed significant differences in the expression of the natural structure of APP server data [24]. As shown in Fig.4(a), the types of multivariate coupling relationships expressed in the $\mathcal{E}$ space are relatively simple and primarily concentrated in regions of low coupling intensity. In contrast, the $\mathcal{P}$ space in Fig.4(b) exhibits a richer variety of relationship types, along with distinct hierarchical and community structure characteristics [25], with clear relationship expressions. In the $\mathcal{H}$ space in Fig.4(c), the expression of hierarchical and community relationships is smoother. This indicates that as the geometric characteristics of the embedding space are enhanced, the geometric structure inherent in the data is more easily captured and expressed. However, it is worth noting that even in datasets where the geometric structure itself is not prominent (such as the network intrusion system in Fig.4(d)), by selecting an appropriate embedding space, the model can still uncover good hierarchical and community structures. This further indicates that the model's mining performance depends not only on the inherent geometric characteristics of the data but more critically on the reasonable adaptation of the data's geometric characteristics to those of the embedding space. Such adaptation is crucial for maximizing the model's ability to reveal the data's structural properties.

**Challenge 3: Confusion regarding the causes of anomalies.** Due to the joint learning strategy employed by existing multivariate anomaly detection models [2, 3, 7, 24] to characterize the spatio-temporal features of multivariate data, anomaly signals undergo coupled diffusion in both the spatio-temporal dimensions. Dynamic dependency features, which are subject to time-varying perturbations, and static coupling associations, which are constrained by spatial limitations, mutually penetrate each other, resulting in a causal masking effect. Existing anomaly causality interpretation studies primarily follow a structure-prior-driven framework [7, 8, 26, 27]: firstly, an initial topological structure is constructed based on association measures such as directed distance matrices, similarity matrices, or covariance matrices between node pairs. Secondly, edge weights are updated through a message aggregation mechanism to model the system's topological evolution. Finally, the temporal dynamic dependencies of nodes and their multivariate spatial coupling relationships are jointly represented and learned to complete data reconstruction and anomaly causation interpretation. In this process, the model traces anomalies based on node weights, with the implicit assumption that high-weight nodes necessarily correspond to core anomaly sources. However, this interpretation framework based on static structural isomorphism assumptions has serious shortcomings. The weight-driven explanation mechanism [28] cannot penetrate the surface correlation layer, essentially simplifying complex system anomalies into a node importance ranking problem, leading to causal confusion between heterogeneous anomaly sources—making it difficult to distinguish the fundamental differences between endogenous system weak anomaly accumulation (such as gradual equipment degradation) and exogenous sudden interference (such as malicious attacks or natural disasters). Although the two may exhibit similar

representations at the data reconstruction error level, their physical causes and mitigation strategies differ fundamentally. Therefore, we significantly reduced mutual interference between causality interpretation through a system topology adaptive discovery mechanism that independently learns spatio-temporal features.

**Challenge 4: Dynamic expansion of sensor scale.** In recent years, with the continuous expansion of the scale of industrial Internet of Things sensors, the timeliness and robustness of anomaly detection models have become core performance indicators. Existing data-driven Transformer or RNN architecture anomaly detection models [18, 26, 7] have a positive correlation between computing resources and sensor scale, significantly reducing model inference speed. Although the field of graph representation learning has achieved structural discovery of millions of nodes based on link prediction [22], the sampling or random walk strategies that rely on real topological relationships [29, 30] cannot serve as effective references for system topology discovery. Therefore, we propose a novel anomaly detection architecture driven by data geometry. Compared to additional memory units that store historical data change patterns to mine potential topological properties, the approach based on Riemannian manifolds to mine the geometric properties of data [31] requires fewer computational resources and is more conducive to lightweight model architecture design. Currently, the research framework for data geometric properties on Riemannian manifolds [32] primarily divides into two strategies: "manifold-tangent-manifold" mapping transformation and direct "manifold-to-manifold" end-to-end operations. However, these hyperbolic combination learning units face significant computational efficiency bottlenecks during training, primarily manifested as: 1) frequent conversions between hyperbolic space and approximate Euclidean space, significantly increasing computational overhead; 2) severely limiting the model's real-time inference response speed. More critically, unsupervised anomaly detection exhibits notable fragility when faced with sudden changes in sensor values: 1) dynamic thresholds exhibit severe fluctuations, disrupting monitoring continuity [33]; 2) insufficient adaptability to concept drift [24] and non-trivial sequence [34] fluctuations; 3) the anomaly scoring mechanism overly relies on normal pattern standards, leading to a significant reduction in system fault tolerance [35]. These defects collectively weaken the model's robustness in real-world industrial environments.

## References


[1] Peng, W., Varanka, T., Mostafa, A., Shi, H. & Zhao, G. Hyperbolic deep neural networks: a survey. *IEEE. T. Pattern. Anal.* **44**, 10023–10044 (2022).

[2] Deng, A. & Hooi, B. Graph neural network-based anomaly detection in multivariate time series. In *Proc. AAAI Conf. Artif. Intell.* **35**, 4027–4035 (2021).

[3] Khodayar, M. & Wang, J. Spatio-temporal graph deep neural network for short-term wind speed forecasting. *IEEE T. Sustain. Energ* **10**, 670–681 (2019).

[4] Jiang, Y. et al. Multivariable time series anomaly detection using heuristic spatio-temporal graph neural network (in Chinese). *Sci. Sin. Inform.* **53**, 1784–1801 (2023).

[5] Ou, S., Zhao, M., Wu, H., Zhang, Y. and Li, S. Machinery degradation trend prediction considering temporal distribution discrepancy between degradation stages. *Eng. Appl. Artif. Intell.* **131**, 107872 (2024).

[6] Baribi-Bartov, S. et al. Supersharers of fake news on Twitter. *Sci* **384**, 979–982 (2024).

[7] Tang, C., Xu, L., Yang, B., Tang, Y. & Zhao, D. GRU-based interpretable multivariate time series anomaly detection in industrial control system. *Comput. Secur.* **127**, 103094 (2023).

[8] Zheng, Y. et al. Correlation-aware spatial–temporal graph learning for multivariate time-series anomaly detection. *IEEE T. Neur Net. Lear. Syst.* **35**, 11802–11816 (2024).

[9] Li, S., Pandey, A., Hooi, B. *et al.* Dynamic graph-based anomaly detection in the electrical grid. *IEEE T. Power Syst.* **37**, 3408–3422 (2021).

[10] Noble, J. & Adams, N. Real-time dynamic network anomaly detection. *IEEE Intell. Syst.* **33**, 5–18 (2018).

[11] Liu, Y., Pan, S., Wang, Y. G. *et al.* Anomaly detection in dynamic graphs via transformer. *IEEE T. Knowl. Data En.* **35**, 12081–12094 (2021).

[12] Watts, D. et al. Collective dynamics of 'small-world' networks. *Nat* **393**, 440–442 (1998).

[13] Barabási, A. L. et al. Emergence of scaling in random networks. *Sci* **286**, 509–512 (1999).

[14] Beghini, F. *et al.* Gut microbiome strain-sharing within isolated village social networks. *Nat* **637**, 167-175 (2025).

[15] Michelle, K. et al. Drivers of epidemic dynamics in real time from daily digital COVID-19 measurements. *Sci* **385**, eadm8103 (2024).

[16] Yang, W. L. et al. Analysis and numerical simulation of computer virus propagation model based on limited resources. *Math. Comput. Simul.* **223**, 494-508 (2024).

[17] Asadi, E. et al. A clustered-LPSEIRS malware propagation model in complex networks. *Appl Netw Sci* **9**, 62 (2024).

[18] Tuli, S. et al. TranAD: Deep transformer networks for anomaly detection in multivariate time series data. In *Proc. VLDB Endow.* **15**, 1201–1214 (2022).

[19] Chen, Z., Chen, D., Zhang, X., Yuan, Z. and Cheng, X. Learning graph structures with transformer for multivariate time-series anomaly detection in IoT. *IEEE Internet Things J.* **9**, 9179–9189 (2022).

[20] Khoshraftar, S. and An, A. A survey on graph representation learning methods. *ACM T. Intel. Syst. Tec.* **15**, 1–55 (2024).

[21] Sun, L. et al. Hyperbolic variational graph neural network for modeling dynamic graphs. In *Proc. AAAI Conf. Artif. Intell.* **35** (2021).

[22] Xu, Y. Y. et al. Scalable and effective temporal graph representation learning with hyperbolic geometry. *IEEE T. Neural Netw. Learn. Syst.* (2024).

[23] Sharafaldin, I., Lashkari, A. H. and Ghorbani, A. A. Toward generating a new intrusion detection dataset and intrusion traffic characterization. In *Proc. 4th Int. Conf. Inf. Syst. Secur. Privacy (ICISSP)*. (2018).

[24] Li, Z. et al. Multivariate time series anomaly detection and interpretation using hierarchical inter-metric and temporal embedding. In *Proc. ACM SIGKDD Conf. Knowl. Discov. Data Min.* (2021).

[25] Adamic, L. A. et al. Search in power-law networks. *Phys. Rev. E* **64**, 046135 (2001).

[26] Zhang, W. et al. A graph attention network-based model for anomaly detection in multivariate time series. *J. Supercomput.* **80**, 8529–8549 (2024).

[27] Huang, X. H. et al. Multivariate time series anomaly detection via dynamic graph attention network and Informer. *Appl. Intell.* **54**, 7636–7658 (2024).

[28] Qu, H. B. et al. GNR: A universal and efficient node ranking model for various tasks based on graph neural networks. *Physica A* **632**, 129339 (2023).

[29] Yang, C. Y. et al. A meta-path graph-based graph homogenization framework for machine fault diagnosis. *Eng. Appl. Artif. Intel.* **121**, 105960 (2023).

[30] Xia, F. et al. Random walks: A review of algorithms and applications. *IEEE T. Em. Top. Comput. I.* **4**, 95–107 (2019).

[31] Nickel, M. et al. Poincaré embeddings for learning hierarchical representations. In *NeurIPS* **30**, 6338–6347 (2017).

[32] Dai, J. D. et al. A hyperbolic-to-hyperbolic graph convolutional network. In *Proc. IEEE/CVF Conf. Comput. Vis. Pattern Recognit.* (2021).

[33] Su, Y. et al. Robust anomaly detection for multivariate time series through stochastic recurrent neural network. In *Proc. ACM SIGKDD Conf. Knowl. Discov. Data Min.* 2828–2837 (2019).

[34] Wu, R. J. and Keogh, E. J. Current time series anomaly detection benchmarks are flawed and are creating the illusion of progress. *IEEE T. Knowl. Data En.* **35**, 2421–2429 (2021).

[35] Shi Y, et al. Robust anomaly detection for multivariate time series through temporal GCNs and attention-based VAE. *Knowl. Based. Syst* **275**, 110725 (2023).

4. The 2.1 section of related work part is not complete. Currently, multivariate anomaly detection has been widely studied but authors do not highlight the difference of the proposed method with existing methods.

**Response 4:** Thank you very much for pointing out that the relevant section in Section 2.1 needs improvement. The issue you raised is very critical: although existing research is extensive, most explanations of the causes of anomalies in multivariate systems rely on known system topologies. When this prior knowledge is lacking, the difficulty of explanation increases significantly. This is precisely the starting point of our work and the biggest difference from existing research — we are committed to directly mining the natural topological properties of systems and explaining the causes of anomalies within an unsupervised framework. Additionally, we take into account challenges such as embedding space distortion and structural rigidity constraints, which distinguish our approach from traditional methods based on Euclidean space. The latter often fail to capture micro - level anomaly relationships due to their neglect of relationship expressions under embedding distortion.

**Revision 4:** **[(Page 4 ∼ 5 ) 2.1 Multivariate Anomaly Detection Methods]** In traditional machine learning anomaly detection, two core assumptions are made: anomaly events have low probability characteristics and tend to be distributed in low-density areas of the data. Based on this, researchers have developed theoretical frameworks for identifying anomalous data points using probability density estimation or clustering analysis [7, 8]. Examples include the isolated

forest (iForest) algorithm based on path isolation mechanisms [9], the local outlier factor (LOF) algorithm utilizing local density comparisons [10], and the DBSCAN algorithm that integrates density-based clustering [11]. Given the unique characteristics of time-series data, additional methods have emerged, such as the MERLIN algorithm based on dynamic sub-sequence matching [12] and the MPdist algorithm using segmented distance metrics [13]. However, these traditional methods are highly sensitive to data distribution patterns, sample size, and hyperparameter settings, and even minor data perturbations or parameter shifts can lead to significant degradation in detection performance. With the development of deep learning technology, data-driven feature representation learning methods have gradually overcome the limitations of manual feature engineering, significantly improving the robustness and generalization capabilities of models.

Early deep learning anomaly detection primarily relied on supervised learning frameworks, which essentially modeled anomaly detection as a binary classification problem, learning decision boundaries through labeled positive and negative samples. For example, models based on DNNs and CNNs [14, 15] utilize multi-layer nonlinear transformations or convolutional operations to extract high-dimensional features, and dynamically learn decision boundary parameters based on loss functions and data labels. AE, VAE-based models jointly optimize reconstruction error and classification loss functions to enhance the ability to distinguish anomaly samples during feature reconstruction [16, 17]. GAN implicitly learns the distribution of normal data through the adversarial game between the generator and the discriminator, thereby constructing an accurate anomaly detection boundary [18]. Additionally, some researchers have integrated Transformer or RNN to fuse long-range dependencies and historical change features in the data, thereby improving the robustness of the model [19, 4, 2, 20]. However, the strong dependence of such methods on labeled data limits their large-scale application in industrial scenarios.

Currently, multivariate anomaly detection is based on an unsupervised learning framework, whose core idea is to quantify anomalies by comparing the residuals between observed data and model predictions. For example, the ImDiffusion model based on probabilistic diffusion [21], the time series modeling-based OmniAnomaly and InterFusion models [6, 5, 22], as well as the MSCRED model based on convolutional time-series neural networks [23] and the CAE-M model [24]. While these methods can effectively detect multivariate anomalies, they lack modeling of anomaly relationships. Therefore, researchers have utilized graph neural network information aggregation mechanisms to dynamically model multivariate spatio-temporal coupling relationships, such as the MTAD-GAT model [25] and GDN [1], GRN [2], etc., which learn the historical behavioral data of each sensor and construct coupling relationships between multiple variables based on metric learning methods. Although these methods have made progress in terms of multivariable spatio-temporal relationships, the deep integration of spatio-temporal features leads to confusion regarding the source of anomalies. Additionally, researchers have modeled the dynamic topological relationships among multiple variables using information propagation dynamics. However, geometric distortions in the data representation space limit the mining potential of such models, making it difficult to effectively uncover latent coupling relationships or weak anomalies. Furthermore, related studies on the multivariate anomaly causality interpretation, such as GSL-ST [3] and GRN [2] generally rely on weight-driven explanation mechanisms, which can only analyze anomalous sensors and their associated sensors, lacking a clear reference benchmark for anomaly interpretation.

Based on the current state of research, studies on dynamic relationship modelling of multivariate anomaly causes are still constrained by core challenges such as rigid, predefined structures, geometric distortion in data representation, and confusion in the dimensions of anomaly interpretation. The cumulative effect of these challenges makes it extremely difficult to construct a reliable reference benchmark for explaining multivariate anomaly causes.

## References


[1] Deng, A. & Hooi, B. Graph neural network-based anomaly detection in multivariate time series. In *Proc. AAAI Conf. Artif. Intell.* **35**, 4027–4035 (2021).

[2] Tang, C., Xu, L., Yang, B., Tang, Y. & Zhao, D. GRU-based interpretable multivariate time series anomaly detection in industrial control system. *Comput. Secur.* **127**, 103094 (2023).

[3] Zheng, Y. et al. Correlation-aware spatial–temporal graph learning for multivariate time-series anomaly detection. *IEEE T. Neur Net. Lear. Syst.* **35**, 11802–11816 (2024).

[4] Tuli, S. et al. TranAD: Deep transformer networks for anomaly detection in multivariate time series data. In *Proc. VLDB Endow.* **15**, 1201–1214 (2022).

[5] Li, Z. et al. Multivariate time series anomaly detection and interpretation using hierarchical inter-metric and temporal embedding. In *Proc. ACM SIGKDD Conf. Knowl. Discov. Data Min.* (2021).

[6] Su, Y. et al. Robust anomaly detection for multivariate time series through stochastic recurrent neural network. In *Proc. ACM SIGKDD Conf. Knowl. Discov. Data Min.* 2828–2837 (2019).

[7] Epanechnikov, V. A. Non-parametric estimation of a multivariate probability density. *Theory Probab. Its Appl.* **14**(1), 153–158 (1969).

[8] Yin, C., Zhang, S., Wang, J. and Kim, J.-U. An improved K-means using in anomaly detection. In *Proc. First Int. Conf. Comput. Intell. Theory, Syst. Appl. (CCITSA)*, 129–132 (2015).

[9] Xu, H., Pang, G., Wang, Y. and Wang, Y. Deep isolation forest for anomaly detection. *IEEE T. Knowl. Data En.* **35**, 12591–12604 (2023).

[10] Breunig, M. M., Kriegel, H.-P., Ng, R. T. and Sander, J. LOF: identifying density-based local outliers. In *Proc. ACM SIGMOD Int. Conf. Manag. Data*, 93–104 (2000).

[11] Arlia, D. and Coppola, M. Experiments in parallel clustering with DBSCAN. In *Proc. Euro-Par Int. Conf. Parallel Process.* 326–331 (2001).

[12] Nakamura, T. et al. Merlin: parameter-free discovery of arbitrary length anomalies in massive time series archives. In *Proc. IEEE Int. Conf. Data Min. (ICDM)*, 1190–1195 (2020).

[13] Gharghabi, S. et al. An ultra-fast time series distance measure to allow data mining in more complex real-world deployments. *Data Min. Knowl. Dis.* **34**, 1104–1135 (2020).

[14] Zhang, Q. et al. Lightweight and accurate DNN-based anomaly detection at edge. *IEEE T. Parall Distrib. Syst.* **33**, 2927–2942 (2022).

[15] Zhu, B. et al. ADFormer: generalizable few-shot anomaly detection with dual CNN-transformer architecture. *IEEE T. Instrum. Meas.* **74**, 1–16 (2025).

[16] Xiang, P., Ali, S., Jung, S. K. and Zhou, H. Hyperspectral anomaly detection with guided autoencoder. *IEEE T. Geosci. Remote Sens.* **60**, 1–18 (2022).

[17] Lin, S. et al. Anomaly detection for time series using VAE-LSTM hybrid model. In *Proc. IEEE Int. Conf. Acoust. Speech Signal Process. (ICASSP)*, 4322–4326 (2020).

[18] Li, D. et al. MAD-GAN: multivariate anomaly detection for time series data with generative adversarial networks. In *Proc. Int. Conf. Artif. Neural Netw. (ICANN)*, 703–716 (2019).

[19] Ma, M., Han, L. and Zhou, C. BTAD: a binary transformer deep neural network model for anomaly detection in multivariate time series data. *Adv. Eng. Inform.* **56**, 101949 (2023).

[20] Luo, W., Liu, W. and Gao, S. A revisit of sparse coding based anomaly detection in stacked RNN framework. In *Proc. IEEE Int. Conf. Comput. Vis. (ICCV)*, 341–349 (2017).

[21] Chen, Y. et al. ImDiffusion: imputed diffusion models for multivariate time series anomaly detection. *Proc. VLDB Endow.* **17**, 359–372 (2023).

[22] Hundman, K. et al. Detecting spacecraft anomalies using LSTMs and nonparametric dynamic thresholding. In *Proc. ACM SIGKDD Int. Conf. Knowl. Discov. Data Min.* 387–395 (2018).

[23] Zhang, C. et al. A deep neural network for unsupervised anomaly detection and diagnosis in multivariate time series data. In *Proc. AAAI Conf. Artif. Intell.* **33**, 1409–1416 (2019).

[24] Zhang, Y. et al. Unsupervised deep anomaly detection for multi-sensor time-series signals. *IEEE T. Knowl. Data En.* **35**(2), 2118–2132 (2021).

[25] Zhao, H. et al. Multivariate time-series anomaly detection via graph attention network. In *Proc. IEEE Int. Conf. Data Min. (ICDM)*, 841–850 (2020).

5. The related work section lacks the outline of hyperbolic graph learning research. However, it has been used as the key technique for the proposed framework.

**Response 5:** Thank you very much for your valuable comments on the overview of hyperbolic graph learning research in Section 2. We deeply recognize that to better support our research, we must more clearly explain the development of hyperbolic graph representation learning and its intrinsic connection to our work, especially its unique value in addressing anomaly detection challenges. Based on this, we make the following adjustments:

- It is important to distinguish the "graph structure discovery" task proposed in this paper from traditional "graph structure learning" and "hypergraph structure learning". While the latter are important research directions, their applications often rely on prior knowledge of the system's topological structure. However, our work focuses precisely on the more challenging scenario where prior topological properties are completely unknown, aiming to discover the natural graph structure of data in an unsupervised manner. Therefore, existing graph structure learning methods, whether based on Euclidean space or hyperbolic space, cannot be directly applied to our "graph structure discovery" task.

- To better understand the background of hyperbolic graph learning, we review the origins of graph structure learning [1]. Graph structure learning originates from the link prediction task in graph neural networks, with early research primarily focusing on topological representations of static graphs and employing various techniques such as metric learning, optimization - based learning, generative models, and unsupervised methods. However, these early studies largely rely on known topological properties for supervised learning, which limits their large - scale application. Later, with the rise of dynamic graphs (whose topological properties are typically known), graph representation learning gains widespread attention in the field of graph neural networks (GNNs) [2] and further diversifies into discrete dynamic graph learning and continuous dynamic graph learning. Although graph representation learning has made significant progress, its methods cannot be directly applied to our graph structure discovery task. This is because the essence of graph representation learning remains link prediction within a supervised framework with known topological structures, whereas the core of our work is unsupervised structure mining under completely unknown topological properties.
- The rise of hyperbolic graph learning originates from an important discovery in the field of graph representation learning: researchers observe that hyperbolic spaces, with their unique geometric structure, are more effective than traditional Euclidean spaces at accommodating complex relationships with hierarchical or recursive characteristics, and their inherent curvature helps improve the model's ability to recognize relationship patterns [3]. Building on this advantage, a series of hyperbolic graph learning methods emerge, such as HDNN, HGCNN, HGAT, and HGCN [4, 5, 6], attracting widespread attention from the academic community. The superior characteristics exhibited by hyperbolic spaces are not only widely recognized in the field of graph representation learning but also provide key insights for our current research.

## References


[1] Zhao, J. et al. Heterogeneous graph structure learning for graph neural networks. *Proc. AAAI Conf. Artif. Intell.* **35**, 4697–4705 (2021).

[2] Wu, Z. et al. A comprehensive survey on graph neural networks. *IEEE T. Neural Netw. Learn. Syst.* **32**, 4–24 (2020).

[3] Nickel, M. and Kiela, D. Learning continuous hierarchies in the Lorentz model of hyperbolic geometry. In *Proc. Int. Conf. Mach. Learn.*, 3776–3785 (2018).

[4] Nickel, M. et al. Poincaré embeddings for learning hierarchical representations. In *NeurIPS* **30**, 6338–6347 (2017).

[5] Gulcehre, C. et al. Hyperbolic attention networks. In *Proc. Int. Conf. Learn. Represent.* (2019).

[6] Chami, I. et al. Hyperbolic graph convolutional neural networks. *Adv. Neural Inf. Process. Syst.* **32** (2019).


**Revision 5: [(Page 5 $\sim$ 9) 2.2 Graph Structure Discovery and Learning, 2.2.1 Graph Structure Discovery, 2.2.2 Graph Structure Learning, 2.2.3 Graph Representation Learning, 2.2.4 Hyperbolic Graph Representation Learning]** Section 2.2 aims to clearly define and elaborate on the two related yet distinct concepts of graph structure discovery and graph structure learning. The content of this section is organized as follows: firstly, in Section 2.2.1, we explicitly distinguish the fundamental differences between graph structure discovery (the unsupervised task of uncovering natural structures, which is the focus of this paper) and graph structure learning (typically conducted under known topologies), laying the groundwork for subsequent discussions. Secondly, Section 2.2.2 reviews the development history of graph structure learning and identifies the technical challenges commonly faced in this field. Subsequently, Section 2.2.3 further explores the origins of graph representation learning, focusing on the fundamental differences between it and the graph structure discovery task we are addressing, as well as the unique challenges this entails. Finally, in Section 2.2.4, we focus on hyperbolic graph representation learning, tracing its developmental origins and elucidating how its unique properties provide key insights for the research work in this paper.

6. Whether is the AH-GSL effective for hyperbolic graph structure learning? Because the feature reconstruction optimization may not promote the adjacent structure match the hyperbolic structure characteristics. Here, authors should give more theory analysis.

**Response 6:** Thank you very much for your valuable feedback on the Hyperbolic Adaptive Graph Structure Learning (AH-GSL) method. We carefully consider the critical issue you raised regarding the potential limitations of feature reconstruction optimization in aligning adjacent structural features with hypersurface structural features and conduct a theoretical reconstruction accordingly. As you aptly observe, our previous approach of using feature reconstruction strategies to separate multivariate spatio-temporal coupling relationships indeed exhibits significant limitations in terms of interpretability. Therefore, to effectively reduce the interference of multivariate temporal dependencies and coupling relationships, enhance the adaptability of neighborhood structures to their geometric properties under hyperbolic

space embedding, and particularly focus on improving model interpretability, we comprehensively restructure the HAO model's overall framework, as detailed in Fig.4. This reconstruction encompasses multiple aspects, including lightweight framework design, theoretical definitions of the universality of functional components across different embedding spaces, and comprehensive validation of the effectiveness of spatio-temporal relationship structures. **Once again, we sincerely thank you for your valuable feedback, which plays a crucial role in improving the overall quality of our paper.** Our specific modification contents are as follows:

- To more accurately reflect our research work, we revise and expand the core concepts. First, we correct the concept of "Hyperbolic Adaptive Graph Structure Learning" (AH-GSL) proposed in Version 1 and revise it to "Hyperbolic Adaptive Graph Structure Discovery" (AH-GSD) (for detailed explanations, please refer to **Response 2.4** and **Response 2.5**). This modification aims to more accurately express the core functionality of our model in discovering the inherent structure of a system from data under unknown prior topological conditions rather than merely "learning" existing graph structures.
- We define and design a general form for the core components shared by multivariate data in different embedding spaces (Euclidean space $\mathcal{E}$, Poincaré sphere space $\mathcal{P}$, Hyperbolic space $\mathcal{H}$), such as linear transformation mechanisms, message aggregation mechanisms, and attention mechanisms. This work aims to ensure that these key modules can be flexibly applied across spaces, laying the foundation for subsequent processing.
- Based on the aforementioned general components and the graph structure discovery process proposed in this paper, we redesign the HAO model framework (see Fig.4 for details). The HAO model is a data geometry-driven, hyperbolic adaptive space-aware multivariate time series anomaly detection model. Its core lies in constructing a unified multi-task learning framework that integrates data reconstruction, structure discovery, and geometric parameter learning.

  The core design of this framework is its two-stage natural structure discovery optimization process. Firstly, it uses a multi-perspective relationship generation component in general space (from the perspectives of temporal dependencies and multivariable coupling), combined with its general distance measurement function and curvature adjustment mechanism, to dynamically adapt to the geometric characteristics of multivariable data and generate relationships. Secondly, through incremental topological optimization, based on probabilistic statistical principles, these multi-perspective feature relationships are consolidated from the perspective of the frequency of their occurrence. Finally, we uniformly define data reconstruction, structure discovery, and geometric parameter learning as a multi-task joint optimization problem.
- The experimental validation section (Section 4.6) provides an in-depth analysis of the natural structure formed by multivariate data temporal dependencies and multivariate coupling. We evaluate the stability of its convergence process and carefully examine its structural geometric characteristics, such as hierarchical structure, community structure, and unique temporal decay structure. Additionally, we assess the potential of this method to transcend its current application scenarios and explore its potential value in other fields.

**Revision 6: [(Page 11 $\sim$ 20) 3. HAO Model, 3.1 Symbol Conventions, 3.2 Initial Embedding of Multivariate Data, 3.2 Adaptive Hyperbolic Pre-training Framework, 3.3 Joint Optimization Objective]** Section 3 provides a detailed introduction to the HAO model framework. Firstly, we redraw the model framework diagram and streamline the description of the execution process to enhance clarity. In Section 3.1, we establish general definitions for basic operations in the Euclidean space $\mathcal{E}$, the Poincaré sphere space $\mathcal{P}$, and the Hyperbolic space $\mathcal{H}$, along with related theorems and lemmas, laying the groundwork for subsequent work. Based on this, Section 3.2 delves into the core components of the model, including hyperbolic linear transformations, hyperbolic graph attention convolutional layers, multivariate spatio-temporal graph construction, and constrained initialization, and adaptive hyperbolic graph structure discovery. We reorganize and refine the theoretical framework and optimize the presentation. Finally, Section 3.3 proposes a joint optimization objective, integrating data reconstruction, structure discovery, and geometric parameter learning into a unified multi-task learning framework for a solution.

7. There are many representation errors in this paper, such as the data scientific representation in Table 1, etc.

**Response 7:** Thank you very much for pointing out the numerous errors in the paper. We have thoroughly checked the entire text. We are especially grateful for your pointing out the inaccuracies in Table 1, which we have revised.

**Revision 7: [(Page 21 $\sim$ 23) 4.1 Dataset]** The experimental part of this paper used 16 publicly available datasets (Table 3), covering multiple different fields such as server cluster failures [5], Mars rover failures [6], and natural gas pipeline intrusions [13]. Given the diversity of the dataset's source scenarios, we have broadly categorized the anomaly types into point-level anomalies, regional anomalies, and sequence anomalies [4]. To facilitate the discussion of how these anomalies manifest and the challenges of identifying them in both univariate (Fig.8) and multivariate (Fig.9) scenarios, we provide detailed illustrations. Additionally, we have recorded the anomaly types included in this dataset in Table 3.

Table 1: Dataset Overview.

| Number | Dataset | Dataset Background Description | Statistical Features | | | | | Anomaly Types | | |
|---|---|---|---|---|---|---|---|---|---|---|
| | | | Train | Test | Dimensions | Anomalies | Entity | Point | Regional | Sequence |
| 1 | ASD [5] | Application Server Dataset (ASD) is a dataset collected by a team at Tsinghua University from the service cluster of a large internet company. The dataset contains 45 days of observation data (uniformly distributed at 5-minute intervals), divided into 30 days of data with no anomalies and 15 days of data containing anomalies. The dataset includes 19 metrics describing server status, covering key aspects such as CPU, memory, network, and virtual machines. | 102331 | 51840 | 19 | 0.0461 | 12 | ✓ | ✓ | - |
| 2 | MSL [6] | Mars Science Laboratory (MSL) is sensor and actuator data from the Mars rover provided by NASA. | 58317 | 73729 | 55 | 0.1048 | 27 | ✓ | - | ✓ |
| 3 | SMAP [6] | Soil Moisture Active Passive (SMAP) is soil sample data and remote sensing information data provided by NASA's Mars rover. | 140825 | 444035 | 25 | 0.1285 | 55 | ✓ | ✓ | - |
| 4 | SMD [7] | Server Machine Dataset (SMD) is a collection of operational data from a large internet company's computer cluster, compiled by a team from Tsinghua University. The dataset records the continuous resource utilization of a computing cluster consisting of 28 servers over a five-week period. | 708405 | 708420 | 38 | 0.0415 | 28 | ✓ | - | - |
| 5 | SWaT [8] | Secure Water Treatment (SWaT) system is a water treatment operation testing platform. Data collection lasted for 11 days, with the system operating around the clock. During the last four days of the 2016 collection process, a total of 36 attacks were recorded, targeting sensors (such as water level sensors and flow meters) and actuators (such as valves and pumps). | 496800 | 449919 | 51 | 0.2344 | 1 | ✓ | ✓ | - |
| 6 | PSM [9] | Pooling Server Metrics (PSM) dataset originates from the eBay shopping website and collects internal data from multiple application server nodes. The dataset consists of 13 weeks of normal operating data and 8 weeks of data containing anomalies. | 14990 | 14990 | 26 | 0.0795 | 1 | ✓ | ✓ | ✓ |
| 7 | MSDS [10] | Multi-Source Distributed System (MSDS) is a complex distributed system testing platform created by the Technical University of Berlin. Machine metrics are collected using Glances to gather metrics from physical nodes (such as CPU, memory, and load). Application logs are aggregated through OpenStack services to collect logs from all services running on physical nodes. Distributed tracing identifies workflows by capturing information such as HTTP requests, service latency, project associations, and the duration of different service request segments (including RPC and DB layer timing). | 29286 | 29286 | 10 | 0.7225 | 1 | ✓ | - | ✓ |
| 8 | WADI [11] | Water Distribution (WADI) dataset records 16 days of continuous operation, including 14 days of normal operation and 2 days of attack scenarios. Data collection covers all network traffic, sensor readings, and actuator statuses. | 1048571 | 172801 | 123 | 0.0599 | 1 | ✓ | ✓ | - |
| 9 | Synthetic [12] | Synthetic is a synthetic data method proposed by researchers at the University of Notre Dame to simulate the operational conditions of real power plants. The method is based on randomly selecting sine or cosine functions using a random seed and adding time delays, frequency changes, and random Gaussian noise to generate time series. Using this method, we generated 30 time series (each containing 20,000 data points) and randomly injected 5 shock-like outliers with durations of 30, 60, and 90 time units. | 10000 | 10000 | 30 | 0.048 | 1 | ✓ | - | - |
| 10 | GPT [13] | Gas Pipeline Testbed (GPT) was created by Gao Wei et al. to simulate a typical SCADA test system, including components such as MTU, RTU, and HMI. The system controls air pumps, pressure sensors, pressure relief valves, and electromagnetic pressure relief valves, with a pipeline pressure range of 0-20 PSI and a maximum acceptable pressure of 22 PSI. The system operates in three modes: low-pressure mode (approximately 0.1 PSI), medium-pressure mode (approximately 10 PSI, range 9-11 PSI), and high-pressure mode (approximately 20 PSI, range 18-22 PSI). Pressure exceeding 22 PSI or the system being in a mode transition state is considered an anomaly. | 14119 | 14119 | 26 | 0.0118 | 1 | - | ✓ | - |
| 11 | Power System [14] | Power System Datasets were created by Utam Adhikari et al. in collaboration with Raymond Boggs and Justin Beever from Oak Ridge National Laboratory (ORNL). They contain measurement data from power transmission systems under normal, disturbed, controlled, and cyberattack conditions. Data types include synchronous phasor measurements, as well as log information from the Snort intrusion detection system, simulated control panels, and relays. | 1282 | 5224 | 127 | 0.6609 | 1 | ✓ | - | ✓ |
| 12 | Gas Pipeline [15] | Gas Pipeline Datasets were created through a collaboration between Justin Beever and Raymond Boggs at Oak Ridge National Laboratory (ORNL). The Michigan State University (MSU) team was responsible for providing the raw data logs, while the ORNL team formatted these logs into usable datasets. | 37706 | 48510 | 26 | 0.5165 | 1 | - | ✓ | - |
| 13 | CICIDS [16] | CICIDS 2017 (Canadian Institute for Cybersecurity Intrusion Detection System 2017) is a critical network intrusion detection system (IDS) research dataset released by the Canadian Institute for Cybersecurity (CIC). Data collection took place over five working days (July 3 to July 7, 2017). Monday was a normal traffic day, while Tuesday through Friday involved the injection of various attacks, including FTP/SSH brute-force attacks, DoS attacks, Heartbleed exploits, web attacks, port scans, botnet activity, and DDoS attacks. These attacks were executed during both morning and afternoon sessions from Tuesday through Friday. | 97718 | 191033 | 78 | 0.01029 | 1 | ✓ | ✓ | ✓ |
| 14 | SKAB [17] | Skoltech Anomaly Benchmark (SKAB) is an anomaly detection benchmark designed for industrial scenarios that evaluate the performance of single-point (outlier) and collective (change point) anomaly detection algorithms. SKAB contains sensor data from 35 independent simulated industrial devices (motors, pumps, etc.). These multivariate time series data sources originate from sensors on test benches and include industrial indicators such as time, motor current, and pump backflow pressure. | 9401 | 1146 | 8 | 0.3517 | 1 | ✓ | ✓ | - |
| 15 | SWAN [18] | SWAN dataset was created by Georgia State University in Atlanta, Georgia, USA, and extracted from the Solar Hemisphere Vector Magnetogram (SHARP) series of space weather HMI active region patches. It compiles multivariate time series observation data from 4,098 solar active regions between May 2010 and December 2018 and records 51 predictive parameters. | 60000 | 60000 | 38 | 0.326 | 1 | ✓ | - | ✓ |
| 16 | GECCO [19] | GECCO dataset was provided by the SPOTeven laboratory during the 2018 Industrial Challenge. GECCO consists of measurement data collected by Thuringia Remote Water Supply Company to monitor water quality at critical nodes in its water supply system (such as water plant outlets and water tower inlets/outlets), recording water quality data and operational data every minute. Any changes in water quality parameters (chlorine dioxide concentration, pH value, oxidation-reduction potential, conductivity, turbidity) are considered events. Changes in operational data (water flow rate, water temperature) may be associated with water quality changes but do not constitute events themselves. | 69260 | 69260 | 9 | 0.0105 | 1 | ✓ | - | ✓ |

**Multivariate Dataset Anomaly Detection Difficulty Ranking: ⋆⋆⋆: #6, #13. ⋆⋆: #1, #2, #3, #5, #7, #8, #11, #14, #15, #16. ⋆: #4, #9, #10 ,#12.**

Currently, the main challenges in multivariable anomaly detection stem from the complexity of multivariable data in dynamic scenarios. To improve detection performance, models need to more deeply integrate the spatio-temporal joint features of multivariable data and accurately represent their coupling relationships, thereby enhancing data reconstruction capabilities and achieving more significant anomaly identification. However, existing research still faces several issues worthy of further consideration. Firstly, while spatio-temporal feature fusion significantly enhances the model's ability to represent multivariable data, it also introduces confusion in the dimension of anomaly causality interpretation. Secondly, the representation of multivariable spatio-temporal coupling relationships heavily relies on prior structural knowledge (e.g., [20] uses the output of topological estimation (NTP) from a grid energy management system (EMS) as a reference topology), and graph representation learning methods (including hyperbolic representation learning discussed in this paper) are employed to uncover spatio-temporal dynamic relationships [21]. However, this reliance on known prior structural knowledge significantly reduces the model's generalization ability in dynamic or unknown scenarios. Finally, this is the core research focus of this paper, anomaly causality analysis is precisely what is lacking in existing research. To address this, this paper proposes a unified learning framework that integrates data reconstruction, natural structure discovery, and geometric parameter adjustment, aiming to explore the aforementioned issues effectively.

## References


[1] Li, Z. et al. Multivariate time series anomaly detection and interpretation using hierarchical inter-metric and temporal embedding. In *Proc. ACM SIGKDD Conf. Knowl. Discov. Data Min.* (2021).

[2] Hundman, K. et al. Detecting spacecraft anomalies using LSTMs and nonparametric dynamic thresholding. In *Proc. ACM SIGKDD Int. Conf. Knowl. Discov. Data Min.* 387–395 (2018).

[3] Morris, T. and Gao, W. Industrial control system network traffic data sets to facilitate intrusion detection system research. In *Critical Infrastructure Protection VIII*, **14**, ISBN 978-3-662-45354-4 (2014).

[4] Li, G. & Jung, J. J. Deep learning for anomaly detection in multivariate time series: approaches, applications, and challenges. *Inform. Fusion.* **91**, 93–102 (2023).

[5] Li, Z. et al. Multivariate time series anomaly detection and interpretation using hierarchical inter-metric and temporal embedding. In *Proc. ACM SIGKDD Conf. Knowl. Discov. Data Min.* (2021).

[6] Hundman, K. et al. Detecting spacecraft anomalies using LSTMs and nonparametric dynamic thresholding. In *Proc. ACM SIGKDD Int. Conf. Knowl. Discov. Data Min.* 387–395 (2018).

[7] Su, Y. et al. Robust anomaly detection for multivariate time series through stochastic recurrent neural network. In *Proc. ACM SIGKDD Conf. Knowl. Discov. Data Min.* 2828–2837 (2019).

[8] Mathur, A. P. et al. SWaT: a water treatment testbed for research and training on ICS security. In *Proc. Int. Workshop Cyber-phys. Syst. Smart Water Netw. (CySWater)* 31–36 (2016).

[9] Abdulaal, A. et al. Practical approach to asynchronous multivariate time series anomaly detection and localization. In *Proc. 27th ACM SIGKDD Int. Conf. Knowl. Discov. Data Min.* 2485–2494 (2021).

[10] Nedelkoski, S. et al. Multi-source distributed system data for AI-powered analytics. In *European Conference on Service-Oriented and Cloud Computing* 161–176 (Springer, 2020).

[11] Chuadhry, M. A., Palleti, V. R. and Mathur, A. P. WADI: a water distribution testbed for research in the design of secure cyber physical systems. In *Proc. 3rd Int. Workshop Cyber-Phys. Syst. Smart Water Netw.* 25–28 (2017).

[12] Zhang, C. et al. A deep neural network for unsupervised anomaly detection and diagnosis in multivariate time series data. In *Proc. AAAI Conf. Artif. Intell.* **33**, 1409–1416 (2019).

[13] Morris, T. and Gao, W. Industrial control system network traffic data sets to facilitate intrusion detection system research. In *Critical Infrastructure Protection VIII*, **14**, ISBN 978-3-662-45354-4 (2014).

[14] Pan, S., Morris, T. and Adhikari, U. Classification of disturbances and cyber-attacks in power systems using heterogeneous time-synchronized data. *IEEE Trans. Ind. Inform.* **11**, 650–662 (2015).

[15] Beaver, J. M., Borges-Hink, R. C. and Buckner, M. A. An evaluation of machine learning methods to detect malicious SCADA communications. In *Proc. 12th Int. Conf. Mach. Learn. Appl. (ICMLA)* **2**, 54–59 (2013).

[16] Sharafaldin, I., Lashkari, A. H. and Ghorbani, A. A. Toward generating a new intrusion detection dataset and intrusion traffic characterization. In *Proc. 4th Int. Conf. Inf. Syst. Secur. Privacy (ICISSP)*. (2018).

[17] Katser, I. D. and Kozitsin, V. O. Skoltech anomaly benchmark (SKAB). Kaggle (2020). https://doi.org/10.34740/KAGGLE/DSV/1693952.

[18] Angryk, R. A. et al. Multivariate time series dataset for space weather data analytics. *Sci. Data* **7**, 227 (2020).

[19] Steffen Moritz, Frederik Rehbach, Sowmya Chandrasekaran, Margarita Rebolledo, and Thomas Bartz-Beielstein. 2018. GECCO Industrial Challenge 2018 Dataset: a water quality dataset for the "Internet of Things: Online Anomaly Detection for Drinking Water Quality" competition at the Genetic and Evolutionary Computation Conference 2018, Kyoto, Japan (2018)).

[20] Li, S., Pandey, A., Hooi, B. *et al.* Dynamic graph-based anomaly detection in the electrical grid. *IEEE T. Power Syst.* **37**, 3408–3422 (2021).

[21] Ranshous, S. et al. Anomaly detection in dynamic networks: a survey. *Wires. Comput. Stat.* **7**, 223–247 (2015).

8. The proposed multivariate anomaly detection framework is complete, but the novelty of designed algorithm is limited, especially, the AH-PF framework greatly relies on existing hyperbolic graph learning models and makes small revision. In addition, the anomaly evaluation method is also simple.

**Response 8:** We sincerely appreciate your insightful comments highlighting the limited novelty of the hyperbolic pre-training framework (AH-PF) in algorithm design and pointing out its reliance on existing hyperbolic graph learning models with only minor modifications. We take the critical issues raised in Reviewer 6 very seriously. Inspired by your insights and suggestions, we significantly restructure the framework of the HAO model (**Response 6**) and thoroughly validate its feasibility through experimental testing. It is worth noting that this restructuring still adheres to the core design principles of natural structure discovery outlined in Version 1.

We thoroughly revise the entire experimental section to address the shortcomings of the original experimental evaluation. We carefully design three complementary experimental modules centered on the core task of anomaly detection. Section 4.4 focuses on anomaly identification, introducing multiple key metrics, including ROC/AUC and P-R/AP, and testing them on a broader range of industrial and academic datasets. Section 4.5 delves into anomaly diagnosis, introducing a

comprehensive evaluation of anomaly source tracking and model reconstruction capabilities. Section 4.6 is dedicated to explaining the causes of anomalies, adding detailed analyses of geometric features such as spatio-temporal structural convergence, community, and hierarchical characteristics, and prospectively exploring their application potential across fields such as aerospace and space observation. In each section, we significantly expand the evaluation dimensions and optimize the visualization of results to ensure a comprehensive and multi-faceted presentation of the HAO model's advanced performance and broad prospects in the field of anomaly detection.

**Revision 8:** **[(Page 11 $\sim$ 20) 3. HAO Model]** Regarding the restructuring and optimization of the HAO model framework, we have provided a complete description of the modifications in Section **Response 6**.

**[(Page 28 $\sim$ 36) 4.4. Multivariate Anomaly Recognition]** To comprehensively evaluate the performance and characteristics of the HAO model, we significantly expand the experimental section, adding seven new analyses: first, we conduct an in-depth analysis of anomaly detection performance using ROC curves and AUC metrics (Section 4.4.1); second, we assess anomaly detection effectiveness using P-R curves and average precision (Section 4.4.2); next, we conduct detailed comparative experiments (Section 4.4.3); then, we examine the model's multi-scenario adaptability and spatio-temporal efficiency (Section 4.4.4); subsequently, we validate the effectiveness of each component through ablation experiments (Section 4.4.5); additionally, we explore the impact of hyperparameters (Section 4.4.6); finally, we conduct a specialized analysis of the model's adaptive curvature learning mechanism (Section 4.4.7).

**[(Page 36 $\sim$ 41) 4.5. Multivariate Anomaly Diagnosis]** To more comprehensively evaluate the model's anomaly detection capabilities, we expand our experiments to include three targeted analyses: first, we evaluate the model's overall anomaly detection performance (Section 4.5.1); second, we investigate its ability to identify the source of anomalies (Section 4.5.2); and finally, we examine the model's effectiveness in detecting anomalies through data reconstruction (Section 4.5.3).

**[(Page 42 $\sim$ 48) 4.6. Multivariate Anomaly Causality Interpretation]** To further explore the intrinsic characteristics of multivariate data and the effectiveness of the model, we expand the experimental section. The newly added experiments include: analyzing the convergence of the natural structure of multivariate spatio-temporal data (Section 4.6.1), exploring its structural characteristics (Section 4.6.2), evaluating the performance of this structure in domain transfer (Section 4.6.3), constructing a multivariate anomaly causality interpretation graph to enhance interpretability (Section 4.6.4), and verifying the practical application value of the model through specific case studies (Section 4.6.5).

9. The paper should perform the case study experiment to verify the effectiveness of learned hyperbolic graph structure.

**Response 9:** Thank you very much for your interest in the practical application potential of the HAO model. We specifically highlight the significant advantages of the HAO model in anomaly detection tasks within the hyperbolic embedding space. Taking the SWaT dataset [1] as an example, which represents a water treatment system, we analyze the cyberattack incident that occurred between 13:10:10 and 13:26:13 on December 28, 2015. The analysis process includes: starting with anomaly identification (Fig.5), transitioning to precise anomaly diagnosis (Fig.6), delving into the explanation of anomaly causes (Fig.7), and utilizing the sensor description table (Table 2) to clarify the roles of key parameters. This end-to-end case analysis effectively demonstrates the HAO model's ability to coordinate across modules and provide a deep explanation of the causes of anomalies.

## References

[1] Mathur, A. P. et al. SWaT: a water treatment testbed for research and training on ICS security. In *Proc. Int. Workshop Cyber-phys. Syst. Smart Water Netw. (CySWater)* 31–36 (2016).

**Revision 9 :** **[(Page 48 $\sim$ 51) 4.6.5 Case Study Analysis]** Our prior research has established the HAO model's significant potential in anomaly detection, diagnosis, and causal interpretation. Nevertheless, the systematic collaboration mechanisms among its internal modules for achieving comprehensive anomaly attribution remain unexplored. This study therefore investigates a cyberattack incident (13:10:10-13:26:13, December 28, 2015) in the SWaT water treatment system [1], executing a four-phase analytical framework: (1) anomaly identification (Fig.5), (2) diagnostic analysis (Fig.6), (3) root cause attribution (Fig.7), and (4) critical sensor parameter documentation (Table 2). This end-to-end demonstration elucidates the HAO model's integrated module coordination mechanisms for anomaly interpretation.

First, based on the anomaly detection results shown in Fig.5, the HAO model successfully identified the attack behavior targeting the water treatment system. Specifically, at timestamp 9394 (as shown in Fig.6(d)), the model first detected a significant anomaly signal, which was mainly manifested as a sharp jump in the multivariate coupling structure difference value. This abnormal state persisted until the system behavior returned to normal around timestamp 10300. Notably, during the entire abnormal period, the differences in the temporal dependency structure remained relatively stable without significant disturbances. This pattern characteristic (significant coupling structure abnormalities while

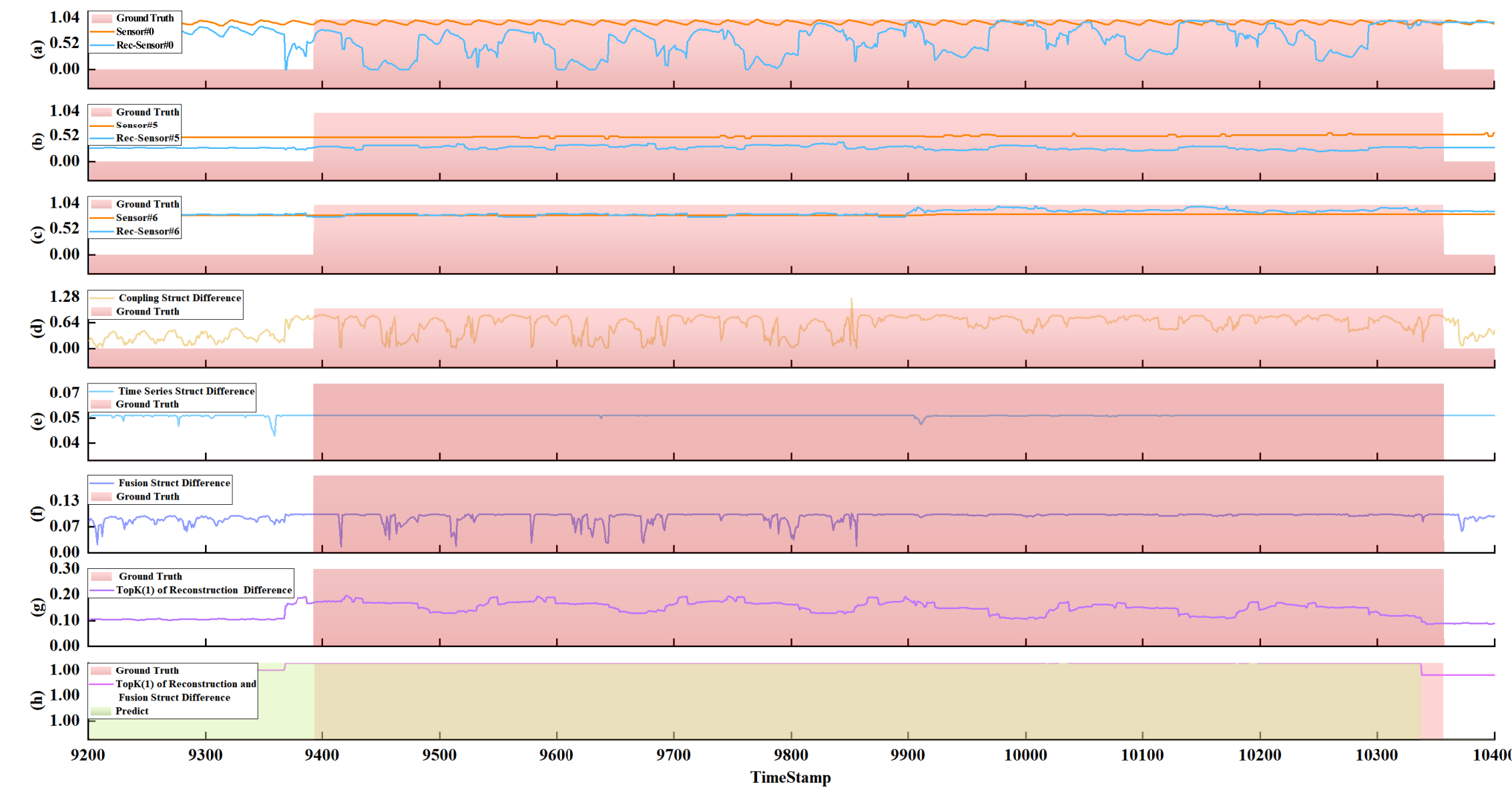


Figure 5: Analysis of Anomaly Source in the $\mathcal{H}$ Space.

temporal dependencies remain stable) strongly suggests that the primary target of this attack was the sensor system rather than long-term factors such as equipment aging. Further analysis revealed that the reconstruction results for Sensor 0 exhibited significant deviations during the anomaly period. Based on the three key pieces of evidence—the sharp increase in coupled structure anomalies, the stability of the temporal structure, and the significant reconstruction deviations in Sensor 0—the HAO model determined the attack onset time to be timestamp 9394. This determination was six time steps earlier than the actual annotated anomaly onset time, demonstrating the model's advantage in early attack detection.

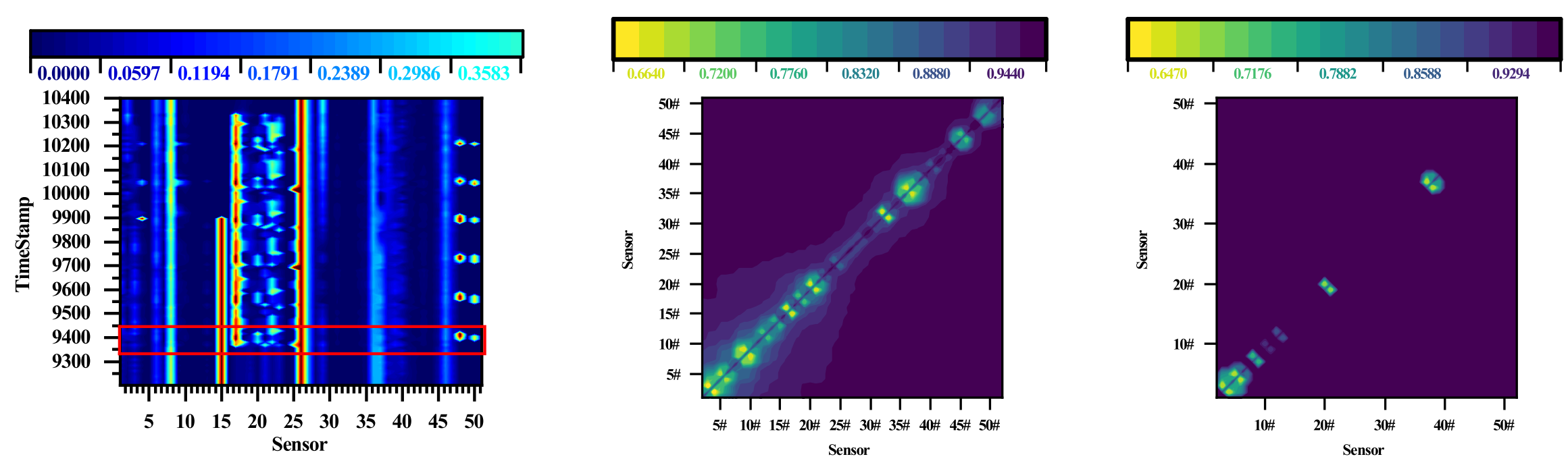


(a) Reconstruction Difference Graph of the SWaT Dataset

(b) Multivariate Coupling Natural Structure of SWaT

(c) Multivariate Coupled Predictive Structure for SWaT

Figure 6: Anomaly Source Localization.

For enhanced anomaly diagnosis, we examined reconstruction discrepancies within the timestamp range 9200-10400 (Fig.6(a)). Analysis revealed that at timestamp 9394, Sensor 17's reconstruction exhibited sudden significant deviation concurrently with similar anomalies in Sensors 45-50. Based on this finding, we compared and analyzed the natural structure of the SWaT system in normal mode (Fig.6(b)) with the predicted structure at timestamp 9394 (Fig.6(c)). The comparison demonstrated supplementary relational alterations in the predicted structure specifically, connections absent in the natural topology. Since structural comparison alone cannot ascertain root causes, we computed structural residuals between predicted and natural structures, applied key-relationship filtering, and derived an interpretable residual spectrum (Fig.7(a)). This spectrum distinctly identifies three anomaly signatures:

(1) **Local community structure deviation:** The local community formed by sensors 15, 16, 17, 18, 19, 20, 21, 22, and 24 exhibits significant deviation from the normal behavior model (natural structure) in terms of coupling patterns. This deviation strongly suggests that the application scenario or operating status of the subsystem has changed at abnormal times.

(2) **Cross-regional abnormal connections:** More notably, the graph clearly shows "atypical cross-regional connections" between sensors 17 and 15 and sensors 47 and 49. This abnormal connection pattern, combined with the inherent linear workflow characteristics of the water treatment system, suggests that abnormal conditions occurring upstream (such as in the regions where sensors 17 and 15 are located) may be transmitted through the system's internal workflow, ultimately triggering a chain reaction of abnormal conditions in downstream regions (such as those where sensors 47 and 49 are located).

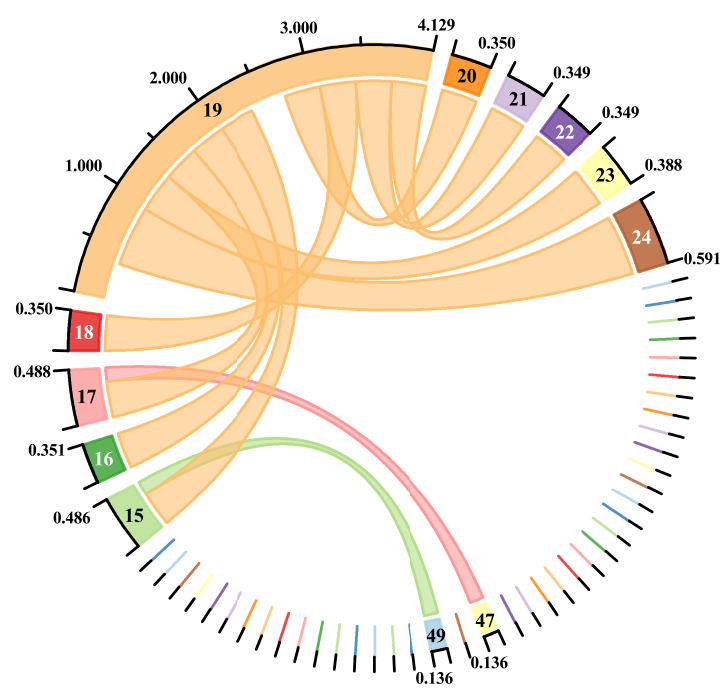


(a) SWaT Coupled Correlation anomaly Interpretation Difference Graph

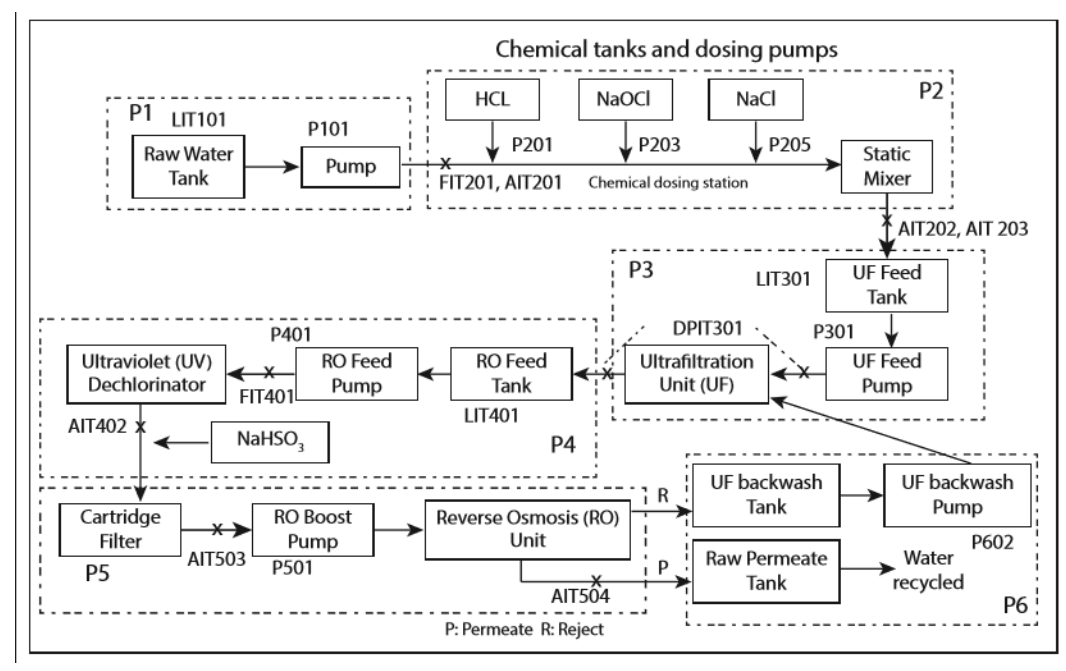


(b) SWaT Water Treatment Plant Component Distribution Graph [1]

Figure 7: Attack Analysis and Source Explanation on SWaT Dataset.

Table 2: SWaT Anomaly Attribution: Sensor Indexing and Descriptions [1].

| No | Name | Type | Description |
|---|---|---|---|
| 15 | P-205 | Actuator | Dosing pump; NaOCl dosing pump. |
| 16 | P-206(backup) | Actuator | Dosing pump; NaOCl dosing pump. |
| 17 | DPIT-301 | Sensor | Wash process Differential pressure indicating transmitter, Controls the backwash process. |
| 18 | FIT-301 | Sensor | Flow meter; Measures the flow of water in the UF stage. |
| 19 | LIT-301 | Sensor | Level Transmitter; UF feed water tank level. |
| 20 | MV-301 | Actuator | Motorized Valve; Controls UF-Backwash process. |
| 21 | MV-202 | Actuator | Motorized Valve; Controls water from UF process to DeChlorination unit. |
| 22 | MV-303 | Actuator | Motorized Valve; Controls UF-Backwash drain. |
| 23 | MV-304 | Actuator | Motorized Valve; Controls UF drain. |
| 24 | P-301(backup) | Actuator | UF feed Pump; Pumps water from UF feed water tank to RO feed water tank via UF filtration. |
| 47 | PIT-503 | Sensor | Pressure meter;RO reject pressure. |
| 49 | F-601 | Actuator | Pump; Pumps water from RO permeate tank to raw water tank (not used for data collection). |

To ultimately determine the core anomaly source, We analyze the functional characteristics of the relevant sensors based on the system sensor information table (Table 2). Results indicate that sensor 15 functions as an execution unit, whereas sensor 17 operates as a data acquisition unit. From an attack feasibility perspective, compromising data acquisition units (e.g., sensor 17) demonstrates higher operational feasibility and stealth than directly attacking physical execution units. Furthermore, the residual spectrum reveals cross-regional anomalous connections between sensor 17 and downstream execution units (19–24), conclusively identifying sensor 17 as the core anomaly source.

The underlying logic is that sensor 17 serves as a data acquisition unit, and any malicious tampering with the values it reports can mislead the control system, causing the service mode of downstream execution units (19, 20, 21, 22, 23, 24) that rely on this input to undergo unexpected and significant changes. Conversely, if the anomaly originates from the 15th execution unit itself since its function is limited to performing specific operations, it is unlikely to actively trigger a service mode transition across the entire subsystem (which includes multiple execution units). The control mechanism of the water treatment system dictates that the decision to switch service modes is based on sensor feedback data. Therefore, as a critical data source, tampering with the values reported by sensor 17 is the most direct and reasonable cause of the downstream chain reaction of anomalies. According to the anomaly attack logs recorded by the SWaT system, this attack occurred between 13:10:10 and 13:26:13 on December 28, 2015. The attacker maliciously tampered with the readings of sensor 17 (DPIT-301), specifically by altering the normal output value (below 40 kPa) to an abnormally high value (above 40 kPa).

Comprehensive analysis reveals that Fig.6(a) clearly shows significant data reconstruction anomalies within the range of sensor numbers 40 to 50. This phenomenon is highly consistent with the cross-regional anomalies revealed in Fig.7(a) (manifested as abnormal connections between sensor 17 and sensor 47, and sensor 15 and sensor 49). By conducting spatial localization analysis using the physical distribution map of the SWaT water treatment plant components shown in Fig.7(b), it can be determined that sensor 47 and execution unit 49 are both located downstream of the initial attack target (sensor 17). This spatial correlation reveals the propagation path of the attack's impact: malicious tampering with the upstream sensor 17 (data acquisition unit) triggers a chain reaction through the system's inherent workflow, causing abnormal increases in the monitored values of downstream sensor 47 and further triggering abnormal execution behavior of downstream actuator 49.

Most importantly, the HAO model not only successfully identified the initial attack point (sensor 17) and direct anomalies (reconstruction deviation), but also revealed the complete propagation chain and systemic impact of the attack across the entire physical system by mining cross-regional anomaly connections (17→47, 15→49) and locating downstream component anomalies (increased value of 47, execution anomaly of 49). The discovery of these interconnected phenomena provides strong empirical evidence supporting the HAO model's ability to capture potential, complex anomalies with spatial propagation characteristics.

## References


[1] Mathur, A. P. et al. SWaT: a water treatment testbed for research and training on ICS security. In *Proc. Int. Workshop Cyber-phys. Syst. Smart Water Netw. (CySWater)* 31–36 (2016).


### Reviewer #2 (Remarks on code availability):

The code provides a readme file to introduce the implementation steps and gives datasets links. The code provides a readme file to introduce the implementation steps and gives datasets links.

**Response :** Thank you very much for pointing out the issues related to the code description in our work. We have made the necessary improvements. The latest modified source code has been uploaded to GitHub for everyone to use. In addition, we have provided detailed information on how to obtain each anomaly detection dataset to ensure the transparency and reproducibility of the research.

**Revision :** **[(Page 53 ) Data Availability ]**The benchmark datasets used in this study are all publicly available on the Internet and can be accessed through the following URLs: **ASD**: `https://github.com/zhhlee/InterFusion/tree/main/data/processed`. **SMAP&MSL**:`https://www.kaggle.com/datasets/patrickfleith/nasa-anomaly-detection-dataset-smap-msl`. **SMD**:`https://github.com/NetManAIOps/OmniAnomaly/tree/master/ServerMachineDataset`. **SMAP&WADI**: `https://itrust.sutd.edu.sg/itrust-labs_datasets/`. **PSM**:`https://github.com/eBay/RANSynCoders/tree/main/data`. **MSDS**:`https://github.com/snedelkoski/multi-source-observability-dataset`. **GPT&Power System&Gas Pipeline**:`https://sites.google.com/a/uah.edu/tommy-morris-uah/ics-data-sets`. **CI-CIDS**:`https://www.unb.ca/cic/datasets/ids-2017.html`. **SKAB**:`https://github.com/waico/SkAB`. **SWAN**:`https://bitbucket.org/gsudmlab/workspace/projects/FP`. **GECCO**:`https://www.spotseven.de/gecco/gecco-challenge/gecco-challenge-2018`.

**[(Page 53) Code Availability ]**All the code required for reproducing the results of this study, along with the source code for the pip packages, is available under the MIT license in the GitHub repository at `https://github.com/StructureMan/HAO`.

## Reviewer #3 (Remarks to the Author):

This manuscript presents a theoretical framework for multivariate anomaly detection and evaluates its performance on several datasets by comparing it against existing models. While the authors aim to introduce an innovative framework, the manuscript, in its current form, has significant limitations in terms of clarity, rigor, and novelty. Several issues should be addressed before consideration for publication.

**Response :** We sincerely appreciate your valuable and constructive feedback. As you accurately point out, we are fully aware that the manuscript indeed has shortcomings in terms of clarity, rigor, and novelty. As a result, we conduct a thorough self - reflection and extensively research the current state of related studies. This process further solidifies our belief that data-geometry-driven modeling approaches will be a crucial direction for explaining the causes of multivariate anomalies in the future. These approaches fundamentally differ from dynamic graph relationship modeling that relies on historical prior topological knowledge, as well as existing dynamic evolutionary analysis paths that integrate multivariate spatio-temporal features based on Euclidean space. Our core work focuses on uncovering the intrinsic, unchanging topological properties of a system—its "natural structure." This structure reflects the endogenous relationships within the system during normal operation and serves as a critical reference point for understanding the causes of anomalies. Therefore, we propose a data geometry - driven HAO model under an unsupervised framework, aiming to establish an interpretable generalization mechanism with open adaptability. This ensures that even when faced with unknown anomalies, the credibility of explanations and the accuracy of root cause analysis are maintained. This represents a significant exploration of the transition of industrial cognitive intelligence from "anomaly detection" to "causal insight" and directly impacts the reliability of decision-making in complex operational conditions. The core improvements of this revision are summarized as follows:

- We add more detailed explanations and key step analyses to help readers clearly understand our research challenges, core motivations, and model framework design ideas. This improves the clarity of the charts.
- We optimize the loose structure of the theoretical Version 1, carefully categorize the types, scales, and anomaly types of the datasets used in the experimental evaluation, and greatly expand the evaluation indicators for model performance. In particular, for multivariate anomaly detection tasks, we conduct more detailed and in-depth experimental analysis of key links such as identification, diagnosis, and cause interpretation.
- We conduct an in-depth analysis of the fundamental differences between graph structure learning and the graph structure discovery method we propose, pointing out the limitations of graph structure learning under the existing supervised framework, such as the lack of depth in multivariable anomaly causality interpretation research and the lack of reliable explanation benchmarks due to over-reliance on data spatio-temporal dynamic relationship mining. Based on this, we comprehensively restructure the overall framework of the HAO model for discovering the natural spatio-temporal structure of multivariate data and validate its effectiveness through experiments.

1. The authors present Figure 1 as an illustration of "multivariate anomaly detection tasks." However, the figure is merely provided without an explanation in the introduction. The subsequent explanation of different phases—anomaly identification, anomaly diagnosis, and anomaly causality interpretation—lacks a clear connection to the figure. This disjointed presentation may confuse readers.

**Response 1 :** Thank you for pointing out the issues with Fig.1. On the one hand, the figure caption does not clearly explain the various stages of multivariate anomaly detection (anomaly identification, anomaly diagnosis, and anomaly causality interpretation). On the other hand, these explanations lack a clear correspondence with the content of the chart, which can cause misunderstandings. In response to this feedback, we thoroughly revise Fig.1. The new figure not only more clearly defines the core objectives of each detection stage but also explicitly highlights the current limitations of research in the field of multivariable anomaly causality interpretation.

**Revision 1: [(Page 1) 1.0 Introduction]**With the continuous advancement of industrial IoT and information systems, production monitoring sensor [1] plays a key role in data collection and analysis in the production process, which is crucial for ensuring production safety and enhancing intelligent operation and maintenance [2]. The existing multivariate anomaly detection research task (Fig.8) in [3, 4, 5] still faces challenges such as the unknown true topology of the system and distortion of the data embedding space [6], confusing anomaly interpretation dimensions, and the dynamic scaling of sensor scale. In addition, the “interpretation comfort zone trap” of over-reliance on structural a priori severely limits the anomaly interpretation capability of dynamic scenarios [7]. Therefore, we aims to construct an open and adaptive explanation generalization mechanism. This mechanism aims to maintain explanation credibility and traceability accuracy when unknown anomalies emerge. It is not only an important exploration to realize industrial cognitive intelligence from “anomaly recognition” to “causal insight” but also concerns the credibility of decision-making under complex working conditions.

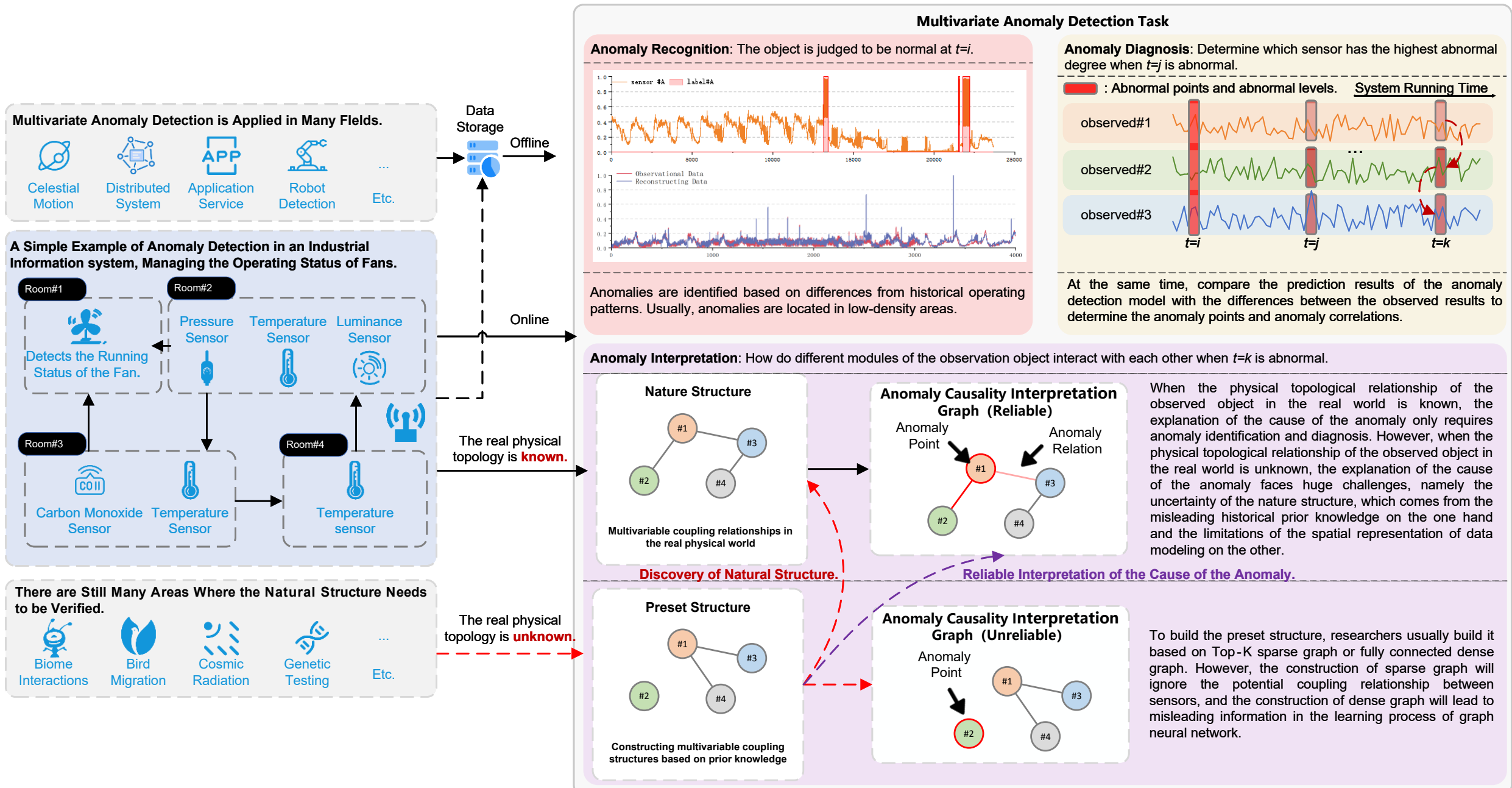


Figure 8: Currently, multivariate anomaly detection has evolved into a three-tiered detection framework [4]: At the detection layer (Anomaly Recognition), anomaly detection uses advanced pattern recognition algorithms to screen for anomalous segments from massive amounts of time series data; at the diagnosis layer (Anomaly Diagnosis), spatio-temporal dynamic relationship analysis is used to precisely locate the spatio-temporal coordinates and threshold conditions where anomalies occur; at the interpretation layer (Anomaly Interpretation), causal reasoning and knowledge graphs reveal the multi-level causal network behind anomalies. This three-tiered paradigm of "detection-diagnosis-interpretation" not only achieves a leap from observing phenomena to understanding their essence but also provides an actionable decision-making basis for continuously optimising production systems.

## References

[1] Sloane, M. et al. Materiality and risk in the age of pervasive AI sensors. *Nat. Mach. Intell.* **7**, 334–345 (2025).

[2] Tao, F., Zhang, H. & Zhang, C. Advancements and challenges of digital twins in industry. *Nat. Comput. Sci.* **4**, 169–177 (2024).

[3] Pazho, A. D. et al. A survey of graph-based deep learning for anomaly detection in distributed systems. *IEEE T. Knowl. Data En.* **36**, 1–20 (2024). https://doi.org/10.1109/TKDE.2023.3282898

[4] Li, G. & Jung, J. J. Deep learning for anomaly detection in multivariate time series: approaches, applications, and challenges. *Inform. Fusion.* **91**, 93–102 (2023).

[5] Ranshous, S. et al. Anomaly detection in dynamic networks: a survey. *Wires. Comput. Stat.* **7**, 223–247 (2015).

[6] Peng, W., Varanka, T., Mostafa, A., Shi, H. & Zhao, G. Hyperbolic deep neural networks: a survey. *IEEE. T. Pattern. Anal.* **44**, 10023–10044 (2022).

[7] Abhilash, P. M. et al. Towards next-gen smart manufacturing systems: the explainability revolution. *npj Adv. Manuf.* **1**, 8 (2024).

2. The manuscript states: "However, existing studies generally adapt structures to data rather than data to structures, meaning that structures preset based on prior knowledge may not accurately reflect the natural data structure, thereby affecting the objectivity of anomaly detection and causality interpretation." The distinction between "structures to data" and "data to structures" is unclear and requires a more precise explanation. If the authors are referring to dynamically evolving models adapting to data, numerous existing methodologies have already addressed this issue in anomaly detection. Below are some relevant references for the authors to refer to.

## References

[1] Ranshous, Stephen, et al. "Anomaly detection in dynamic networks: a survey." Wiley Interdisciplinary Reviews: Computational Statistics 7.3 (2015): 223-247.

[2] Ren, H., Ye, Z., & Li, Z. (2017). Anomaly detection based on a dynamic Markov model. Information Sciences, 411, 52-65.

[3] Pan, Kaikai, Peter Palensky, and Peyman Mohajerin Esfahani. "From static to dynamic anomaly detection with application to power system cyber security." IEEE Transactions on Power Systems 35.2 (2019): 1584-1596.

[4] Li, Shimiao, et al. "Dynamic graph-based anomaly detection in the electrical grid." IEEE Transactions on Power Systems 37.5 (2021): 3408-3422.

[5] Noble, Jordan, and Niall Adams. "Real-time dynamic network anomaly detection." IEEE Intelligent Systems 33.2 (2018): 5-18.

[6] Nakayama, Hidehisa, et al. "A dynamic anomaly detection scheme for AODV-based mobile ad hoc networks." IEEE transactions on vehicular technology 58.5 (2008): 2471-2481.

[7] Liu, Yixin, et al. "Anomaly detection in dynamic graphs via transformer." IEEE Transactions on Knowledge and Data Engineering 35.12 (2021): 12081-12094.

**Response 2:** Thank you very much for accurately pointing out two key issues in our work: first, the blurred conceptual boundary between "structure adapts to data" and "data adapts to structure"; second, the universality of dynamic adaptive data models in existing research. We reflect deeply on these issues.

Regarding the first question, we recognize that this confusion between the two concepts may indeed lead to misunderstandings. We now clearly distinguish between the two: "structure adapts to data" refers to the process of discovering the intrinsic topological properties of data under conditions of no prior knowledge (unsupervised), such as through measuring the relationships between data points or performing cluster analysis, for example, the process of discovering the molecular structure of DNA or the structure of atomic nuclei. However, due to limitations in observational techniques and data representation methods, this process is prone to omitting potential relationships, such as distortions that occur when embedding geometric relationships in Euclidean space. "Data adapts to structure" refers to the process of classifying unknown relationships using known characteristics of data points (such as friend/stranger relationships in social networks) within a supervised framework. Dynamic graph relationship learning is a prominent area of research in this field, which employs clustering core ideas and homogeneity principles to perform structural learning through link prediction. Therefore, our research is firmly grounded in "structure adapts to data" rather than "data adapts to structure." This fundamental difference in starting points endows our graph structure discovery method with stronger generalization capabilities. It enables it to be applied to more unknown anomaly detection scenarios and achieve reliable explanations of anomaly causes. A detailed argumentation of this point has been presented in **Response 1**.

Regarding your second question, many existing methods achieve dynamic adaptation to data in anomaly detection (based on the references you provided, we conduct a detailed comparative analysis in the main text). We acknowledge this, but believe that existing methods have the following limitations, prompting us to explore new paths:

- Challenges in Topological Relationship Mining: Reviewing the core framework of existing multivariate anomaly detection models (see Section 2.1), most rely on dynamic graph learning methods aimed at capturing the spatio-temporal dynamic relationships in multivariate data. However, in practical applications, especially when dealing with large-scale networks, sampling methods such as random walks and meta-paths based on prior topological properties often fail. More importantly, a significant characteristic of the multivariable anomaly detection field is the widespread absence of topological attributes, which significantly reduces the effectiveness of directly applying dynamic graph methods to this field (see Section 2.2 for details).
- The challenge of explaining the causes of anomalies: Although capturing dynamic spatio-temporal relationships can improve data reconstruction quality and is beneficial for anomaly detection and diagnosis based on residuals, it is difficult to answer the fundamental question of "why anomalies occur." Research finds that existing models for dynamically adaptive data either lack in-depth analysis of the causes of anomalies, focusing solely on improving feature representation through dynamic relationships, or they only provide local explanations based on anomaly weights or rely on capturing relationships from historically adjacent time points, which are unreliable in cases of topological property anomalies (see Sections 2.2.1 and 2.3 for details).

Based on a summary of the limitations of existing research, we deeply recognize that a reliable reference benchmark is crucial for explaining the causes of anomalies. To this end, we define this benchmark as the "natural structure" and have designed a discovery framework around it, completing theoretical analysis and experimental testing to verify its feasibility and reliability in explaining the causes of anomalies.

It is worth noting that in the experimental phase, the spatio-temporal natural structure of multivariate data also reveals several interesting phenomena, such as exposing temporal chaotic characteristics and demonstrating the hierarchy and community structure of multivariate coupling (Section 4.6.2), providing a reference for domain knowledge transfer

(Section 4.6.3). These findings further validate the unique value of our work and its breakthrough in overcoming the limitations of existing research.

**Revision 2: [(Page 1) 1. Introduction] Challenge 1: The true topology of the system is unknown.** In an open environment, the lack of explicit optimization objectives for real system topologies and supervision by domain experts makes it difficult to establish a system natural structure that conforms to the essence of industrial scenarios. This issue primarily manifests in static models built based on prior assumptions (e.g., $\epsilon$-radius, $k$-nearest neighbors) [1, 2] struggling to effectively adapt to data-natural topologies (such as nonlinear dependencies and time-varying relationships), resulting in elevated false positive rates and interpretive biases. Although some studies have adjusted parameters through online learning [3], the underlying topological structure remains rigid, failing to achieve a truly "data-adaptive structure." These limitations are particularly pronounced in non-stationary data streams (e.g., progressive degradation monitoring of devices [4]) and systems with dense latent variables (e.g., detection of misinformation in social networks [5]). In recent years, researchers have explored various aspects such as temporal dependencies in multivariate data, sensor coupling, and heuristic learning [1, 3, 6, 7], and have developed adaptive topological dynamic evolution models. However, these models heavily rely on the true topological relationships of the system or a large amount of supervised information (such as nodes, edges, and graph features [8, 9, 10]) as explicit optimization objectives. The potential constraints imposed by prior knowledge as out the implicit structure of the data rather than truly adapting to the data structure, which makes it difficult to identify structural anomalies in dynamic scenarios effectively. The limitations of this type of research, especially in multivariate anomaly detection, are that the available topological optimization information is particularly scarce, prompting us to conduct research on the discovery of the natural structure of multivariate data without prior knowledge constraints. We proposed an adaptive hyperbolic graph structure discovery method, providing a reliable reference benchmark and objective evidence to support the explanation of the causes of multivariate anomalies.

## References


[1] Deng, A. & Hooi, B. Graph neural network-based anomaly detection in multivariate time series. In *Proc. AAAI Conf. Artif. Intell.* **35**, 4027–4035 (2021).

[2] Khodayar, M. & Wang, J. Spatio-temporal graph deep neural network for short-term wind speed forecasting. *IEEE T. Sustain. Energ* **10**, 670–681 (2019).

[3] Jiang, Y. et al. Multivariable time series anomaly detection using heuristic spatio-temporal graph neural network (in Chinese). *Sci. Sin. Inform.* **53**, 1784–1801 (2023).

[4] Ou, S., Zhao, M., Wu, H., Zhang, Y. and Li, S. Machinery degradation trend prediction considering temporal distribution discrepancy between degradation stages. *Eng. Appl. Artif. Intell.* **131**, 107872 (2024).

[5] Baribi-Bartov, S. et al. Supersharers of fake news on Twitter. *Sci* **384**, 979–982 (2024).

[6] Tang, C., Xu, L., Yang, B., Tang, Y. & Zhao, D. GRU-based interpretable multivariate time series anomaly detection in industrial control system. *Comput. Secur.* **127**, 103094 (2023).

[7] Zheng, Y. et al. Correlation-aware spatial–temporal graph learning for multivariate time-series anomaly detection. *IEEE T. Neur Net. Lear. Syst.* **35**, 11802–11816 (2024).

[8] Li, S., Pandey, A., Hooi, B. *et al.* Dynamic graph-based anomaly detection in the electrical grid. *IEEE T. Power Syst.* **37**, 3408–3422 (2021).

[9] Noble, J. & Adams, N. Real-time dynamic network anomaly detection. *IEEE Intell. Syst.* **33**, 5–18 (2018).

[10] Liu, Y., Pan, S., Wang, Y. G. *et al.* Anomaly detection in dynamic graphs via transformer. *IEEE T. Knowl. Data En.* **35**, 12081–12094 (2021).


3. The current version of the manuscript contains writing issues, including grammatical errors and unclear expressions, which hinder readability. The writing should be more fluid, precise, and aligned with academic standards. For instance, "..., where M represents the number of sensors, W represents the sampling window length."

**Response 3:** Thank you for pointing out the writing issues in Version 1, such as grammatical errors and unclear expressions, which indeed affected the readability of the article. We take this matter very seriously and conduct a thorough grammatical correction and expression review of the entire text. We recognize that academic papers should strive for fluency and accuracy while strictly adhering to academic norms. To this end, we not only correct specific grammatical errors but also optimize numerous expressions. For example, we rewrite phrases like "where M represents the number of sensors and W represents the length of the sampling window," which were somewhat loose in their explanation, into a more concise and academically appropriate form to enhance overall readability and professionalism.

**Revision 3:** **[(Page 1 ∼ 51 ) Main Text]** We have conducted a comprehensive review and revision of the entire text to address all issues related to grammar, unclear expressions, and loose sentence structures.

4. The figures presented in the manuscript are inadequately explained. Instead of providing only a brief caption, the authors should offer detailed descriptions and analyses. For instance, in Figure 5, the results are not explicitly interpreted. The authors should clearly explain the insights derived from the figure and how it supports their claims. Without such explanation, the figure lacks scientific value and leaves readers uncertain about its intended message.

**Response 4:** Thank you for pointing out the serious lack of data interpretation in Version 1. We deeply recognize the impact this has on the clarity of the paper. To address this, we invest a great deal of effort in reanalyzing and summarizing each chart and experimental result in the paper, striving to provide sufficient and clear explanations for each piece of data. We are particularly sorry for the issue you raised regarding Fig.5, which did not clearly express its intended meaning. We thoroughly revise this figure, making significant adjustments to element labeling, content explanation, and information organization, with the aim of eliminating ambiguity and ensuring that readers can accurately understand our research approach and findings. We sincerely appreciate your assistance in improving the quality of our paper.

**Revision 4:** **[(Page 7) 2.2.4 Hyperbolic Graph Representation Learning]**

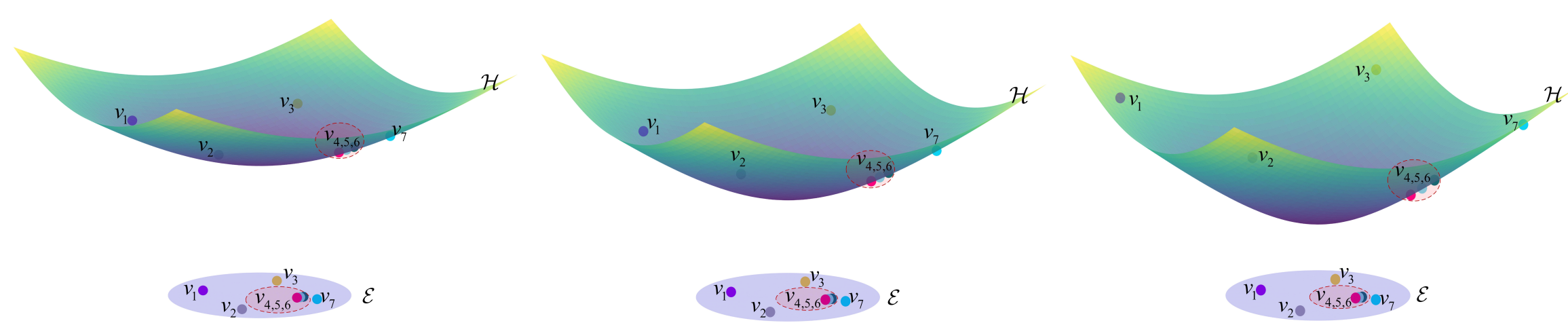

(a) $K = -1$ (b) $K = -2$ (c) $K = -4$

Figure 9: This paper presents the embedding representation of data points in hyperbolic spaces with different curvatures. In Euclidean space, the relationships between data points (such as $v_4, v_5, v_6$) are less distinguishable and exhibit strong clustering (or community structure) [1]. However, when mapped to hyperbolic space, as the absolute value of the curvature of the embedding space gradually increases (corresponding to subgraphs (a) $K = -1$, (b) $K = -2$, and (c) $K = -4$), the geometric distances between these data points significantly increase. This indicates that the distinguishability of their intrinsic relationships is improving overall. The enhanced distinguishability of these key relationships (especially anomalous relationships) not only enables more effective identification of weak anomaly types but, more importantly, provides more refined and robust evidence for revealing the causes of unknown anomaly types.

## References


[1] Ranshous, S. et al. Anomaly detection in dynamic networks: a survey. *Wires. Comput. Stat.* **7**, 223–247 (2015).


5. The experimental design is not well justified. In the Dataset Description section, training and test data proportions are specified. However, when conducting anomaly detection experiments, the authors state: "90% of the data is used for training all models, with the remaining 10% serving as a test set, to evaluate model performance." Given that anomaly detection inherently deals with imbalanced data, where anomalous samples are significantly fewer than normal samples, using only 10% of the data for testing undermines the credibility of the results. A small test set may lead to biased evaluations, reducing the reliability of the reported performance. The authors should explain why they did not maintain the same train-test split ratio as described in the Dataset Description section. They should justify the choice of the test set size and its potential impact on anomaly detection performance, especially in cases where anomalies are rare. A more balanced and statistically sound evaluation strategy should be considered to ensure the robustness and generalizability of the results.

**Response 5:** Thank you very much for pointing out the shortcomings in the rationality argument of our experimental design. In response to this valuable feedback, we have made systematic modifications in three dimensions: comprehensiveness of data coverage, clarity of division strategy, and hyperparameter robustness verification.

- We focus on improving the comprehensiveness and representativeness of the dataset. Considering that anomaly detection is essentially a problem of handling imbalanced datasets (Section 4.1), we carefully designed a diverse dataset covering 16 scenarios with anomaly rates ranging from 0.01 to 0.7 in different application scenarios. This design aims to more comprehensively evaluate the generalization performance of the model in the real world and ensure the reliability of the results.
- We clearly and thoroughly describe the dataset partitioning strategy. In the experimental setup section (Section 4.3), we provide a clear explanation of the use of the training set, validation set, and test set. We

particularly emphasize the common partitioning approach in multivariate anomaly detection tasks: typically, we use one dataset representing the normal operating patterns of the information system for pre-training to learn normal behavior patterns, while using another dataset containing both normal and abnormal operating patterns to evaluate the model's anomaly detection performance and threshold optimization. We provide a detailed explanation of the construction process of this partitioning strategy, enhancing the transparency of the experimental design.

- We expand the test range of the data sample division ratio to cover a wider range from 0.1 to 0.9 in the hyper-parameter experiment section (Section 4.4.6). Additionally, we systematically examine the model's stability performance under different observation window lengths ($W = \{5, 10, 30, 90, 200, 500\}$). Furthermore, we statistically analyze the overall robustness of different data splitting ratios across multiple datasets, further validating the reasonableness and stability of the model configuration.

**Revision 5:** **[(Page 21 $\sim$ 23) 4.1 Dataset]** The experimental part of this paper used 16 publicly available datasets (Table 2), covering multiple different fields such as server cluster failures [5], Mars rover failures [2], and natural gas pipeline intrusions [3]. Given the diversity of the dataset's source scenarios, we have broadly categorized the anomaly types into point-level anomalies, regional anomalies, and sequence anomalies [4]. To facilitate the discussion of how these anomalies manifest and the challenges of identifying them in both univariate (Fig.10) and multivariate (Fig.11) scenarios, we provide detailed illustrations. Additionally, we have recorded the anomaly types included in this dataset in Table 3.

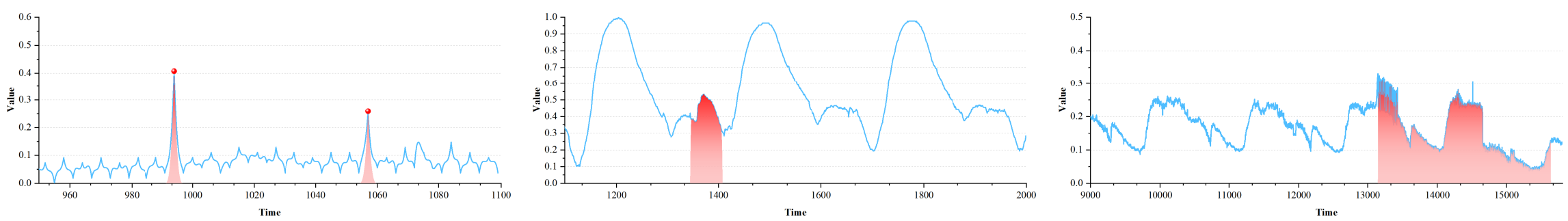


(a) Univariate Point-level Anomaly (b) Univariate Regional Anomaly (c) Univariate Sequence Anomaly

Figure 10: Types of univariate anomalies. (a) Univariate point-level anomalies refer to monitoring values at a specific time point $t$ significantly different from those at other time points. Such anomalies can be further classified as above or below the average level, but the core characteristic is that they differ from the average level. In pattern sequences, anomalous points often manifest as extreme values. Sometimes, these anomalous values may be noise. Since anomalies can affect analysis results, they must be detected and removed. However, such anomalies may also represent important events like hacking attacks or sensor failures. (b) Univariate regional anomalies refer to situations where sensor performance deviates significantly from historical trends within a time interval $\Delta t$. This includes trend-related anomalies (such as Bias or Drift within the region), which manifest as a trend that persists but deviates from the standard level, and more challenging anomalies (such as Erratic, Stuck, or Spike within the region), which manifest as abrupt fluctuations or sustained constancy in numerical values. These anomalies are typically associated with gradual degradation/failure of equipment or changes in application service patterns. (c) Univariate sequence anomalies refer to significant deviations in data patterns from historical patterns starting from time point $t$. Their manifestations are similar to those of difficult-to-handle anomalies within a region (e.g., Erratic, Stuck, Spike). However, they pose higher risks because they may lead to long-term changes in service patterns, thereby rendering detection models based on historical prior information ineffective.

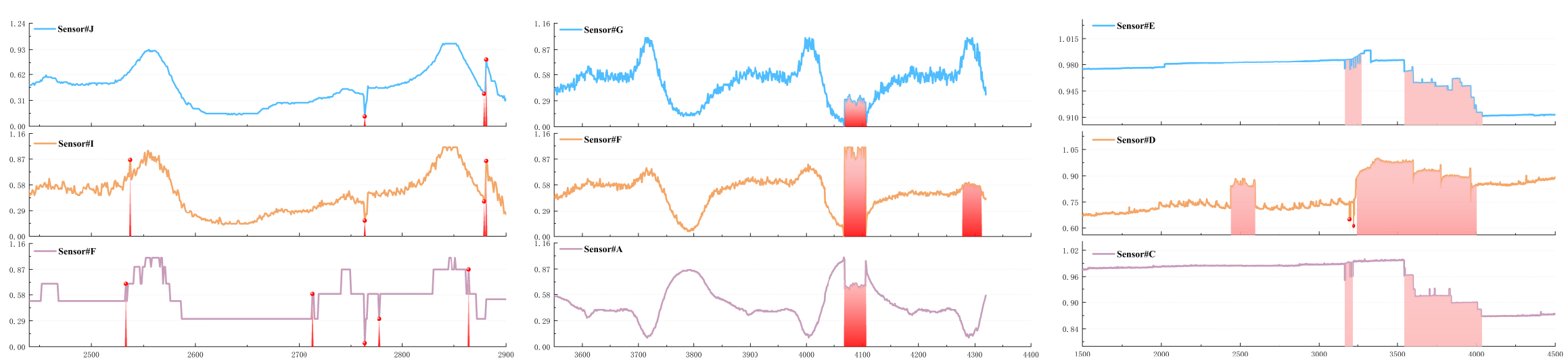


(a) Multivariate Point-level Anomaly (b) Multivariate Regional Anomaly (c) Multivariate Sequence Anomaly

Figure 11: Unlike univariate anomalies, the core of multivariate anomalies lies in the combined performance of multiple variables, which collectively reflect the overall health of the system. This makes the analysis more complex, requiring researchers to go beyond focusing on the historical patterns of individual variables and instead consider the interdependencies and interactions between variables simultaneously. (a) In multivariate point-level anomalies, the key to judgment lies in whether multiple monitoring variables all exhibit significant anomalies at a specific time point $t$. Interactions or competitive relationships between variables may cause weaker point-level anomalies to be more easily masked or overlooked, even though these subtle point-level anomalies may precisely reflect certain representative events (e.g., the instantaneous points in the GECCO [7] dataset where water quality indicators are influenced by the instantaneous/periodic addition of purifying elements). Additionally, when a single sensor exhibits multiple point anomalies while other sensors have few anomalies, the model must accurately distinguish between true anomalies and false anomalies, posing a significant challenge to anomaly detection systems. (b) Multivariate regional anomalies are defined as significant deviations from normal operating patterns within a time window $\Delta t$. For multivariate data with obvious periodicity, anomalies can be effectively detected through historical pattern modeling, data reconstruction, and difference analysis, including handling complex anomalies such as Erratic, Stuck, and Spike. However, the complexity of multivariate data lies in its temporal dependency, the coupling relationships between variables, and the spatio-temporal dynamic evolution of these relationships, all of which hinder our current ability to monitor such anomalies precisely. Taking Sensor #F in Fig.11(b) as an example, the first anomaly exhibited a similar sequence difference pattern to other sensors, while the second anomaly showed significant differences. This inconsistency in behavior makes it difficult to accurately determine whether a change in service mode or device degradation caused the anomaly. This difficulty in identifying the fundamental cause of anomalies constitutes a common challenge that existing research must overcome. (c) Multivariate sequence anomalies are considered one of the greatest challenges currently facing the field of multivariate anomaly detection. Due to the potential absence of periodic characteristics in the temporal dependencies of multivariate data, it becomes extremely difficult to extract regular patterns from the data and identify anomalous differences based on them. Additionally, the erratic phenomena observed in multivariate sequence data can disrupt the sustained dynamic evolution of coupling relationships between variables. Furthermore, the local adaptability of spatio-temporal dynamic relationships often fails to capture such long-term, subtle drift patterns of anomalies. More importantly, point-level or regional anomalies occurring on a single sensor can significantly impact the stability of anomaly detection models, thereby further increasing the difficulty of anomaly detection.

**[(Page 25) 4.3 Experimental Setup]Data set division:** Table 3 shows the dataset used for model training. The training dataset contains monitoring data of observed objects in normal mode, while the test dataset covers scenarios where the system operating status is unknown, including both normal mode data and abnormal mode data. We construct the test set and validation set using a specific data partitioning method, where the data partitioning ratio is denoted as $\beta = 90\%$, and the abnormal ratio of the original test data is denoted as $\gamma$. The steps are as follows:

(1) Preliminary data partitioning: The normal data and abnormal data in the original test dataset are partitioned according to the $\beta$ ratio. That is, the normal data and abnormal data are divided into 10% and 90% parts, respectively.

(2) Constructing the test set: Samples are drawn from 10% of the normal data and 10% of the abnormal data, according to the normal/abnormal ratio (i.e., $\gamma$) of the original test data set, to construct a new test set. For example, if the normal/abnormal sample ratio in the original test dataset is 1000:10, the new test set will be constructed by sampling 100 samples from the 10% portion of normal data and 1 sample from the 10% portion of abnormal data (or scaled according to this ratio).

(3) Constructing the validation set: The remaining 90% of normal data and 90% of abnormal data are combined, and samples are extracted according to the normal/abnormal ratio (i.e., $\gamma$) of the original test data set to construct a new validation set.

**[(Page 34 $\sim$ 35) 4.4.6 Hyperparameter Experiments of the HAO Model]** Analysis of hyperparameter experiments for the HAO model indicates that the small-sample threshold determination algorithm significantly improves the robustness of anomaly detection by selecting a small number of representative samples from large-scale data for threshold calibration. To investigate the impact of different data partitioning ratios on embedding space performance, we designed experiments on 16 benchmark datasets: data is sequentially partitioned at ratios ranging from 0.1 to 0.9, followed by threshold search, while validation is conducted under sliding window conditions $W = \{5, 10, 30, 90, 200, 500\}$. By calculating the average $\overline{F1}$ scores for all window sizes across different segmentation ratios, we found significant differences in anomaly detection performance across different embedding spaces.

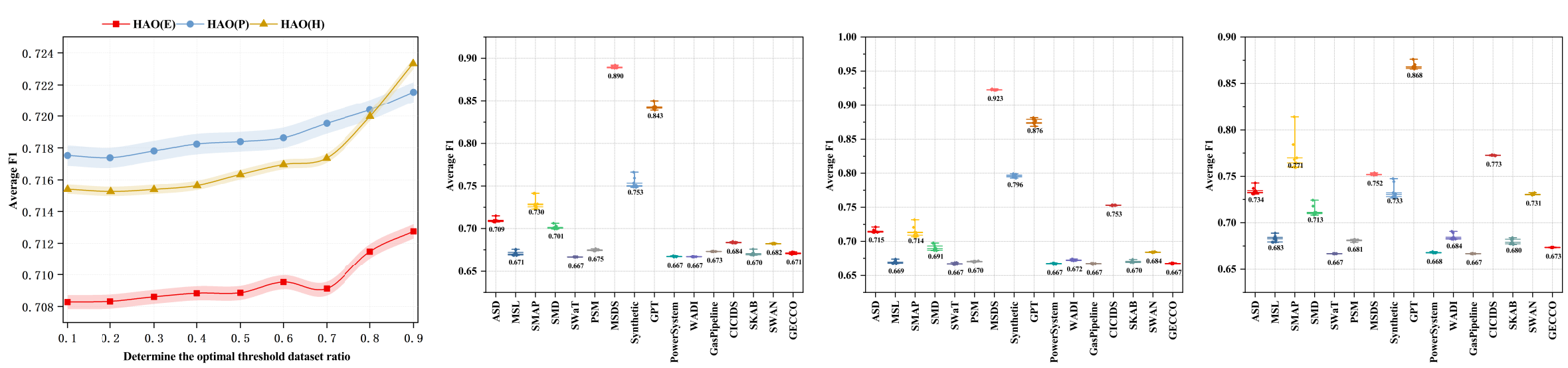


(a) HAO (b) HAO(E) (c) HAO(P) (d) HAO(H)

Figure 12: The Impact of Different Validation Set Split Ratios on HAO Model Performance.

The experimental results show in (Fig.12(a)) that in the $\mathcal{E}$ and $\mathcal{P}$ embedding spaces, when the threshold search data ratio is in the range of 0.1 to 0.7, the fluctuation amplitude of the $\overline{F1}$ score is approximately 0.001, but when the ratio rises to around 0.9, the fluctuation amplitude expands to 0.04. In contrast, the $\mathcal{H}$ space exhibits a fluctuation of 0.007 at the 0.9 ratio, indicating its stronger ability to identify weak anomalies. This characteristic enables it to precisely distinguish subtle anomalies among a large number of anomalous samples. The overall performance is characterized by HAO(E) < HAO(H) < HAO(P), where the Poincaré space exhibits the most significant numerical fluctuations, followed by the Euclidean space and the hyperbolic space being the most stable. This validates the effectiveness of the small-sample threshold algorithm in most anomaly detection scenarios and also demonstrates that adjusting the data partitioning ratio can accommodate higher precision detection requirements.

Regarding the overall robustness of the validation dataset partitioning ratio (Fig.12(b)-(d)), most datasets remain stable in all three embedding spaces. However, in the SMAP dataset (Fig.12(d)), the hyperbolic space exhibits significant fluctuations, which stem from its dynamic enhancement of sensitivity to weak anomalies under different sliding windows, highlighting the space's advantage in responding sensitively to specific data patterns.

## References

[1] Li, Z. et al. Multivariate time series anomaly detection and interpretation using hierarchical inter-metric and temporal embedding. In *Proc. ACM SIGKDD Conf. Knowl. Discov. Data Min.* (2021).

[2] Hundman, K. et al. Detecting spacecraft anomalies using LSTMs and nonparametric dynamic thresholding. In *Proc. ACM SIGKDD Int. Conf. Knowl. Discov. Data Min.* 387–395 (2018).

[3] Morris, T. and Gao, W. Industrial control system network traffic data sets to facilitate intrusion detection system research. In *Critical Infrastructure Protection VIII*, **14**, ISBN 978-3-662-45354-4 (2014).

[4] Li, G. & Jung, J. J. Deep learning for anomaly detection in multivariate time series: approaches, applications, and challenges. *Inform. Fusion.* **91**, 93–102 (2023).

[5] Li, S., Pandey, A., Hooi, B. *et al.* Dynamic graph-based anomaly detection in the electrical grid. *IEEE T. Power Syst.* **37**, 3408–3422 (2021).

[6] Ranshous, S. et al. Anomaly detection in dynamic networks: a survey. *Wires. Comput. Stat.* **7**, 223–247 (2015).

[7] Steffen Moritz, Frederik Rehbach, Sowmya Chandrasekaran, Margarita Rebolledo, and Thomas Bartz-Beielstein. 2018. GECCO Industrial Challenge 2018 Dataset: a water quality dataset for the "Internet of Things: Online Anomaly Detection for Drinking Water Quality" competition at the Genetic and Evolutionary Computation Conference 2018, Kyoto, Japan (2018)).

6. The authors should increase the number of datasets (>15) to provide stronger empirical validation of their model's generalizability. The current dataset selection is insufficient to convincingly demonstrate the robustness of the proposed approach. Expanding the experimental setup with diverse datasets would enhance the manuscript's impact and credibility.

**Response 6:** Thank you very much for pointing out that the dataset selection was insufficient to convincingly demonstrate the robustness of the method, and for suggesting that expanding the dataset would enhance the impact of the paper. We fully agree with this view and make substantial revisions to the experimental section accordingly. Specifically, we expand the number of datasets from six in Version 1 to sixteen, achieving broad cross-domain coverage, including: industrial control (SWaT, GPT), IT operations (ASD, SMD, PSM), critical infrastructure (WADI, GECCO), cybersecurity (CICIDS), and scientific observation (MSL, SMAP, SWAN) (see Table 3). This modification significantly enhances the representativeness of the experimental setup and the robustness of the methodology.

**Revision 6:** **[(Page 21 $\sim$ 23) 4.1 Dataset]** The experimental part of this paper used 16 publicly available datasets (Table 3), covering multiple different fields such as server cluster failures [5], Mars rover failures [6], and natural gas pipeline intrusions [13]. Given the diversity of the dataset's source scenarios, we have broadly categorized the anomaly types into point-level anomalies, regional anomalies, and sequence anomalies [4]. To facilitate the discussion of how these anomalies manifest and the challenges of identifying them in both univariate (Fig.8) and multivariate (Fig.9) scenarios, we provide detailed illustrations. Additionally, we have recorded the anomaly types included in this dataset in Table 3.

Currently, the main challenges in multivariable anomaly detection stem from the complexity of multivariable data in dynamic scenarios. To improve detection performance, models need to more deeply integrate the spatio-temporal joint features of multivariable data and accurately represent their coupling relationships, thereby enhancing data reconstruction capabilities and achieving more significant anomaly identification. However, existing research still faces several issues worthy of further consideration. Firstly, while spatio-temporal feature fusion significantly enhances the model's ability to represent multivariable data, it also introduces confusion in the dimension of anomaly causality interpretation. Secondly, the representation of multivariable spatio-temporal coupling relationships heavily relies on prior structural knowledge (e.g., [20] uses the output of topological estimation (NTP) from a grid energy management system (EMS) as a reference topology), and graph representation learning methods (including hyperbolic representation learning discussed in this paper) are employed to uncover spatio-temporal dynamic relationships [21]. However, this reliance on known prior structural knowledge significantly reduces the model's generalization ability in dynamic or unknown scenarios. Finally, this is the core research focus of this paper, anomaly causality analysis is precisely what is lacking in existing research. To address this, this paper proposes a unified learning framework that integrates data reconstruction, natural structure discovery, and geometric parameter adjustment, aiming to explore the aforementioned issues effectively.

## References

[1] Li, Z. et al. Multivariate time series anomaly detection and interpretation using hierarchical inter-metric and temporal embedding. In *Proc. ACM SIGKDD Conf. Knowl. Discov. Data Min.* (2021).

[2] Hundman, K. et al. Detecting spacecraft anomalies using LSTMs and nonparametric dynamic thresholding. In *Proc. ACM SIGKDD Int. Conf. Knowl. Discov. Data Min.* 387–395 (2018).

Table 3: Dataset Overview.

| Number | Dataset | Dataset Background Description | Statistical Features | | | | | Anomaly Types | | |
|---|---|---|---|---|---|---|---|---|---|---|
| | | | Train | Test | Dimensions | Anomalies | Entity | Point | Regional | Sequence |
| 1 | ASD [5] | Application Server Dataset (ASD) is a dataset collected by a team at Tsinghua University from the service cluster of a large internet company. The dataset contains 45 days of observation data (uniformly distributed at 5-minute intervals), divided into 30 days of data with no anomalies and 15 days of data containing anomalies. The dataset includes 19 metrics describing server status, covering key aspects such as CPU, memory, network, and virtual machines. | 102331 | 51840 | 19 | 0.0461 | 12 | ✓ | ✓ | - |
| 2 | MSL [6] | Mars Science Laboratory (MSL) is sensor and actuator data from the Mars rover provided by NASA. | 58317 | 73729 | 55 | 0.1048 | 27 | ✓ | - | ✓ |
| 3 | SMAP [6] | Soil Moisture Active Passive (SMAP) is soil sample data and remote sensing information data provided by NASA's Mars rover. | 140825 | 444035 | 25 | 0.1285 | 55 | ✓ | ✓ | - |
| 4 | SMD [7] | Server Machine Dataset (SMD) is a collection of operational data from a large internet company's computer cluster, compiled by a team from Tsinghua University. The dataset records the continuous resource utilization of a computing cluster consisting of 28 servers over a five-week period. | 708405 | 708420 | 38 | 0.0415 | 28 | ✓ | - | - |
| 5 | SWaT [8] | Secure Water Treatment (SWaT) system is a water treatment operation testing platform. Data collection lasted for 11 days, with the system operating around the clock. During the last four days of the 2016 collection process, a total of 36 attacks were recorded, targeting sensors (such as water level sensors and flow meters) and actuators (such as valves and pumps). | 496800 | 449919 | 51 | 0.2344 | 1 | ✓ | ✓ | - |
| 6 | PSM [9] | Pooling Server Metrics (PSM) dataset originates from the eBay shopping website and collects internal data from multiple application server nodes. The dataset consists of 13 weeks of normal operating data and 8 weeks of data containing anomalies. | 14990 | 14990 | 26 | 0.0795 | 1 | ✓ | ✓ | ✓ |
| 7 | MSDS [10] | Multi-Source Distributed System (MSDS) is a complex distributed system testing platform created by the Technical University of Berlin. Machine metrics are collected using Glances to gather metrics from physical nodes (such as CPU, memory, and load). Application logs are aggregated through OpenStack services to collect logs from all services running on physical nodes. Distributed tracing identifies workflows by capturing information such as HTTP requests, service latency, project associations, and the duration of different service request segments (including RPC and DB layer timing). | 29286 | 29286 | 10 | 0.7225 | 1 | ✓ | - | ✓ |
| 8 | WADI [11] | Water Distribution (WADI) dataset records 16 days of continuous operation, including 14 days of normal operation and 2 days of attack scenarios. Data collection covers all network traffic, sensor readings, and actuator statuses. | 1048571 | 172801 | 123 | 0.0599 | 1 | ✓ | ✓ | - |
| 9 | Synthetic [12] | Synthetic is a synthetic data method proposed by researchers at the University of Notre Dame to simulate the operational conditions of real power plants. The method is based on randomly selecting sine or cosine functions using a random seed and adding time delays, frequency changes, and random Gaussian noise to generate time series. Using this method, we generated 30 time series (each containing 20,000 data points) and randomly injected 5 shock-like outliers with durations of 30, 60, and 90 time units. | 10000 | 10000 | 30 | 0.048 | 1 | ✓ | - | - |
| 10 | GPT [13] | Gas Pipeline Testbed (GPT) was created by Gao Wei et al. to simulate a typical SCADA test system, including components such as MTU, RTU, and HMI. The system controls air pumps, pressure sensors, pressure relief valves, and electromagnetic pressure relief valves, with a pipeline pressure range of 0-20 PSI and a maximum acceptable pressure of 22 PSI. The system operates in three modes: low-pressure mode (approximately 0.1 PSI), medium-pressure mode (approximately 10 PSI, range 9-11 PSI), and high-pressure mode (approximately 20 PSI, range 18-22 PSI). Pressure exceeding 22 PSI or the system being in a mode transition state is considered an anomaly. | 14119 | 14119 | 26 | 0.0118 | 1 | - | ✓ | - |
| 11 | Power System [14] | Power System Datasets were created by Utam Adhikari et al. in collaboration with Raymond Boggs and Justin Beever from Oak Ridge National Laboratory (ORNL). They contain measurement data from power transmission systems under normal, disturbed, controlled, and cyberattack conditions. Data types include synchronous phasor measurements, as well as log information from the Snort intrusion detection system, simulated control panels, and relays. | 1282 | 5224 | 127 | 0.6609 | 1 | ✓ | - | ✓ |
| 12 | Gas Pipeline [15] | Gas Pipeline Datasets were created through a collaboration between Justin Beever and Raymond Boggs at Oak Ridge National Laboratory (ORNL). The Michigan State University (MSU) team was responsible for providing the raw data logs, while the ORNL team formatted these logs into usable datasets. | 37706 | 48510 | 26 | 0.5165 | 1 | - | ✓ | - |
| 13 | CICIDS [16] | CICIDS 2017 (Canadian Institute for Cybersecurity Intrusion Detection System 2017) is a critical network intrusion detection system (IDS) research dataset released by the Canadian Institute for Cybersecurity (CIC). Data collection took place over five working days (July 3 to July 7, 2017). Monday was a normal traffic day, while Tuesday through Friday involved the injection of various attacks, including FTP/SSH brute-force attacks, DoS attacks, Heartbleed exploits, web attacks, port scans, botnet activity, and DDoS attacks. These attacks were executed during both morning and afternoon sessions from Tuesday through Friday. | 97718 | 191033 | 78 | 0.01029 | 1 | ✓ | ✓ | ✓ |
| 14 | SKAB [17] | Skoltech Anomaly Benchmark (SKAB) is an anomaly detection benchmark designed for industrial scenarios that evaluate the performance of single-point (outlier) and collective (change point) anomaly detection algorithms. SKAB contains sensor data from 35 independent simulated industrial devices (motors, pumps, etc.). These multivariate time series data sources originate from sensors on test benches and include industrial indicators such as time, motor current, and pump backflow pressure. | 9401 | 1146 | 8 | 0.3517 | 1 | ✓ | ✓ | - |
| 15 | SWAN [18] | SWAN dataset was created by Georgia State University in Atlanta, Georgia, USA, and extracted from the Solar Hemisphere Vector Magnetogram (SHARP) series of space weather HMI active region patches. It compiles multivariate time series observation data from 4,098 solar active regions between May 2010 and December 2018 and records 51 predictive parameters. | 60000 | 60000 | 38 | 0.326 | 1 | ✓ | - | ✓ |
| 16 | GECCO [19] | GECCO dataset was provided by the SPOTeven laboratory during the 2018 Industrial Challenge. GECCO consists of measurement data collected by Thuringia Remote Water Supply Company to monitor water quality at critical nodes in its water supply system (such as water plant outlets and water tower inlets/outlets), recording water quality data and operational data every minute. Any changes in water quality parameters (chlorine dioxide concentration, pH value, oxidation-reduction potential, conductivity, turbidity) are considered events. Changes in operational data (water flow rate, water temperature) may be associated with water quality changes but do not constitute events themselves. | 69260 | 69260 | 9 | 0.0105 | 1 | ✓ | - | ✓ |

**Multivariate Dataset Anomaly Detection Difficulty Ranking: ⋆⋆⋆: #6, #13. ⋆⋆: #1, #2, #3, #5, #7, #8, #11, #14, #15, #16. ⋆: #4, #9, #10 ,#12.**


[3] Morris, T. and Gao, W. Industrial control system network traffic data sets to facilitate intrusion detection system research. In *Critical Infrastructure Protection VIII*, **14**, ISBN 978-3-662-45354-4 (2014).

[4] Li, G. & Jung, J. J. Deep learning for anomaly detection in multivariate time series: approaches, applications, and challenges. *Inform. Fusion.* **91**, 93–102 (2023).

[5] Li, Z. et al. Multivariate time series anomaly detection and interpretation using hierarchical inter-metric and temporal embedding. In *Proc. ACM SIGKDD Conf. Knowl. Discov. Data Min.* (2021).

[6] Hundman, K. et al. Detecting spacecraft anomalies using LSTMs and nonparametric dynamic thresholding. In *Proc. ACM SIGKDD Int. Conf. Knowl. Discov. Data Min.* 387–395 (2018).

[7] Su, Y. et al. Robust anomaly detection for multivariate time series through stochastic recurrent neural network. In *Proc. ACM SIGKDD Conf. Knowl. Discov. Data Min.* 2828–2837 (2019).

[8] Mathur, A. P. et al. SWaT: a water treatment testbed for research and training on ICS security. In *Proc. Int. Workshop Cyber-phys. Syst. Smart Water Netw. (CySWater)* 31–36 (2016).

[9] Abdulaal, A. et al. Practical approach to asynchronous multivariate time series anomaly detection and localization. In *Proc. 27th ACM SIGKDD Int. Conf. Knowl. Discov. Data Min.* 2485–2494 (2021).

[10] Nedelkoski, S. et al. Multi-source distributed system data for AI-powered analytics. In *European Conference on Service-Oriented and Cloud Computing* 161–176 (Springer, 2020).

[11] Chuadhry, M. A., Palleti, V. R. and Mathur, A. P. WADI: a water distribution testbed for research in the design of secure cyber physical systems. In *Proc. 3rd Int. Workshop Cyber-Phys. Syst. Smart Water Netw.* 25–28 (2017).

[12] Zhang, C. et al. A deep neural network for unsupervised anomaly detection and diagnosis in multivariate time series data. In *Proc. AAAI Conf. Artif. Intell.* **33**, 1409–1416 (2019).

[13] Morris, T. and Gao, W. Industrial control system network traffic data sets to facilitate intrusion detection system research. In *Critical Infrastructure Protection VIII*, **14**, ISBN 978-3-662-45354-4 (2014).

[14] Pan, S., Morris, T. and Adhikari, U. Classification of disturbances and cyber-attacks in power systems using heterogeneous time-synchronized data. *IEEE Trans. Ind. Inform.* **11**, 650–662 (2015).

[15] Beaver, J. M., Borges-Hink, R. C. and Buckner, M. A. An evaluation of machine learning methods to detect malicious SCADA communications. In *Proc. 12th Int. Conf. Mach. Learn. Appl. (ICMLA)* **2**, 54–59 (2013).

[16] Sharafaldin, I., Lashkari, A. H. and Ghorbani, A. A. Toward generating a new intrusion detection dataset and intrusion traffic characterization. In *Proc. 4th Int. Conf. Inf. Syst. Secur. Privacy (ICISSP)*. (2018).

[17] Katser, I. D. and Kozitsin, V. O. Skoltech anomaly benchmark (SKAB). Kaggle (2020). https://doi.org/10.34740/KAGGLE/DSV/1693952.

[18] Angryk, R. A. et al. Multivariate time series dataset for space weather data analytics. *Sci. Data* **7**, 227 (2020).

[19] Steffen Moritz, Frederik Rehbach, Sowmya Chandrasekaran, Margarita Rebolledo, and Thomas Bartz-Beielstein. 2018. GECCO Industrial Challenge 2018 Dataset: a water quality dataset for the "Internet of Things: Online Anomaly Detection for Drinking Water Quality" competition at the Genetic and Evolutionary Computation Conference 2018, Kyoto, Japan (2018)).

[20] Li, S., Pandey, A., Hooi, B. *et al.* Dynamic graph-based anomaly detection in the electrical grid. *IEEE T. Power Syst.* **37**, 3408–3422 (2021).

[21] Ranshous, S. et al. Anomaly detection in dynamic networks: a survey. *Wires. Comput. Stat.* **7**, 223–247 (2015).

7. The manuscript does not clearly define how anomalies are labeled in the experimental datasets. The authors must specify how they determine which samples are normal and which are anomalous. In the absence of domain-specific knowledge, can anomalies be identified based on waveform characteristics, inter-variable relationships, amplitude thresholds, or other quantitative measures? A more detailed discussion is necessary to establish the validity of the anomaly labels used in the study.

**Response 7:** We sincerely appreciate your reminder to pay attention to the abnormal annotation method. In Version 1, this point was not sufficiently explained, which may have caused readers to have difficulties understanding it. Therefore, we add a dedicated discussion in the revised version. Firstly, we clarify the criteria for determining general abnormal types. Secondly, we provide a detailed explanation of the structured decision-making approach we propose based on the causes of abnormalities. Third, we identify common quality issues, such as missed and incorrect annotations in the current dataset annotations. Finally, we conduct a specific analysis of how these annotation defects directly correlate with false positives and false negatives during model runtime. Through these additions, we aim to more clearly present the challenges of abnormal annotations and demonstrate the effectiveness of our method.

**Revision 7:** **[(Page 21 $\sim$ 23) 4.1 Dataset]** Regarding the field of multivariable anomaly detection and the rules for determining general anomaly types, we have provided detailed explanations in **Response 5** and **Response 6**.

**[(Page 38 $\sim$ 41) 4.5.2 Root Cause Diagnosis of Anomalies]** To further investigate the specific roles of temporal dependency and multivariate coupling in anomaly detection, we use the ASD dataset and analyze various difference metrics for representative sensors across the different geometric embedding spaces ($\mathcal{E}, \mathcal{P}, \mathcal{H}$) of the HAO model (as shown in subfigures a, b and c of Fig.13). In our analysis, we calculate the indicators of differences in data reconstruction (subfigure g), the structural reconciliation difference (subfigure f), and the Top-1 metric for the fusion of structural and data reconstruction differences (subfigure h). We relate it to temporal dependence with its natural structural differences (subfigure e), as well as multivariate coupling relationships and their structural characteristics (subfigure d). These comparisons are illustrated in Fig.13 Fig.14 and Fig.15.

Based on the embedding results of multivariate data in the $\mathcal{E}$ space (Fig.13), We found the following key phenomena:

(1)Synergistic enhancement effect of anomaly characteristics: During the 722-760 time period, the multivariate coupling structural difference (SSD) significantly increases while the temporal structural difference (TSD) remains stable. These two factors jointly amplify the data reconstruction error, ultimately forming a synergistic enhancement effect in anomaly detection. This mechanism enables HAO(E) model to precisely locate short-term anomalies (such as brief high-confidence predictions within this timeframe), confirming its ability to decouple anomaly sources. However, the coarse-grained anomaly annotation intervals (such as around 722-760) require the system to screen numerous low-risk areas, increasing the localization burden. HAO(E) model achieves fine-grained anomaly tracing by analyzing the prediction natural structure differences (TSD/SSD) that result from the interplay of temporal dependencies and multivariate coupling. This significantly reduces the localization complexity. Notably, its accurate identification of weak anomalies around 3380 (labeling true anomalies) further confirms the model's sensitive detection capability.

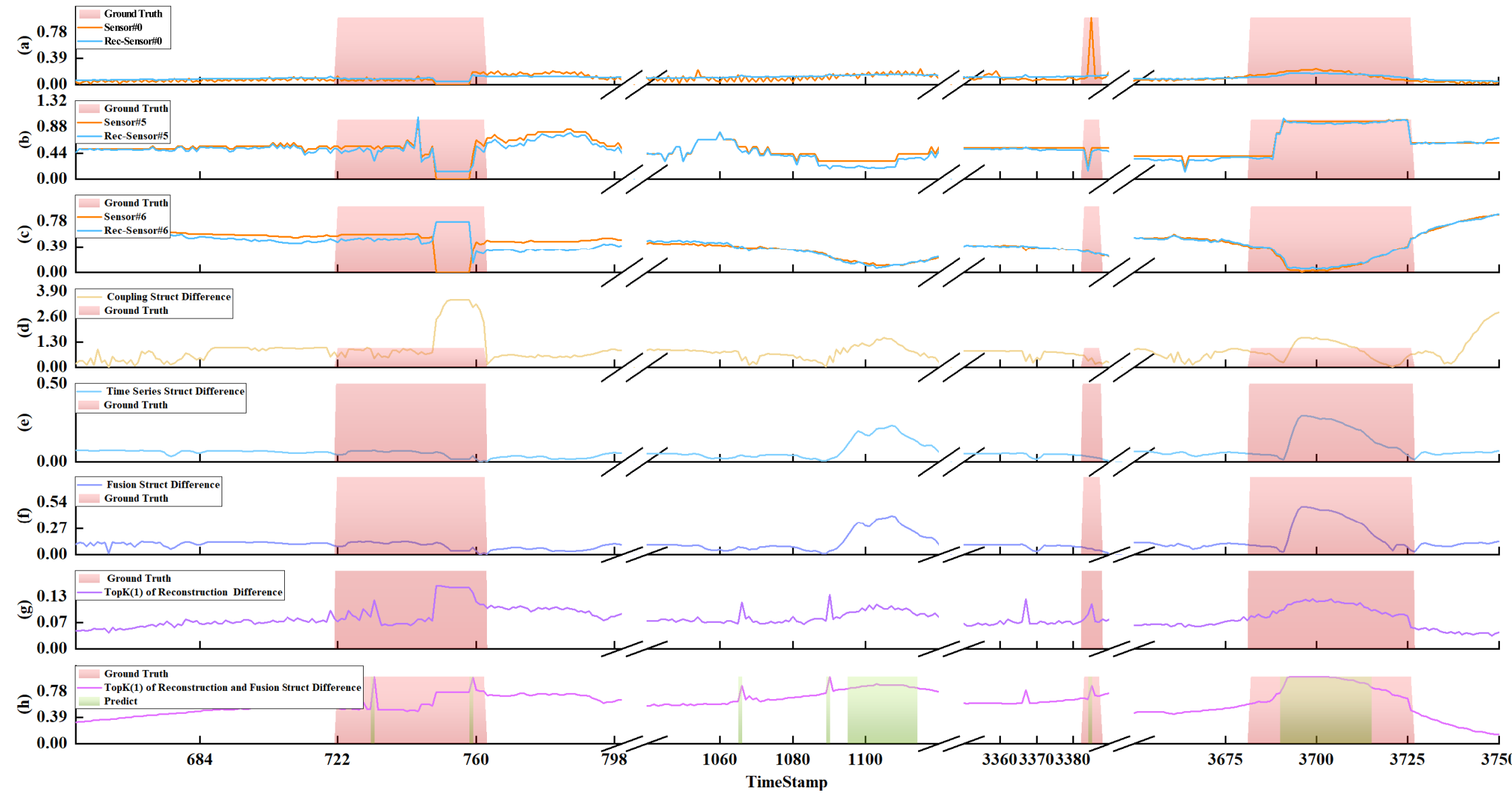

Figure 13: Anomaly Source Analysis in $\mathcal{E}$ Space.

(2) Identification and false alarm analysis of unlabeled anomalies: During the 1060-1080 time period, the model detects statistically significant fluctuations in the reconstructed data (unlabeled), which may be caused by: 1) the system elasticity failing to trigger the anomaly labeling threshold, and 2) Reconstruction error discrimination bias in HAO(E) model . However, during the 1100 time period, despite significant fluctuations in both SSD and TSD (unlabeled), this pattern closely resembles the labeled anomaly in the 3700 time period (as shown in Fig.13(e) and Fig.13(d)), and HAO(E) model successfully identifies both. This indicates an annotation gap in the 1100 time period while demonstrating the model's ability to capture potential anomalies.

(3) Strict determination mechanism based on structural parsing: HAO(E) model exhibits conservative characteristics in anomaly localization, which arise from its accurate perception of anomaly sources. For example, during the 3700 time period, SSD and TSD fluctuate synchronously and are accurately captured by the model, but the predicted anomaly region is significantly smaller than the annotated interval (Fig.13(h)). This spatial mismatch between prediction and annotation fundamentally reflects the model's strategy for achieving a low false positive rate through structural difference threshold constraints, providing a dual safeguard for the strictness of anomaly detection.

Based on the results shown in Fig.14, embedding multivariate data in the $\mathcal{P}$ space significantly reduces the false negative rate and false positive rate of anomaly detection compared to embedding in the $\mathcal{E}$ space. Regarding the reduction of the false negative rate, the HAO(E) model fails to effectively identify anomalies in medium-length intervals around timestamp 760. In contrast, the HAO(P) model significantly reduces the false negative rate in this area by extending the anomaly detection range from short intervals to medium-length intervals. In terms of reducing the false positive rate, the HAO(E) model exhibits uncertain anomalies around 1060 and 1080 (it is difficult to determine whether this is due to data mislabeling or model performance issues). However, the HAO(P) model further confirms that these two points are indeed false positives. Notably, the $\mathcal{P}$ space embedding is more sensitive to structural errors, especially TSD, with reconstruction difference values stabilizing within the 0-0.3 range. This model effectively integrates both types of information through structural harmonization differences, demonstrating excellent stability in anomaly detection.

As shown in Fig.15, after embedding multivariate data into the $\mathcal{H}$ space, the $\mathcal{H}$ space exhibits superior fitting characteristics compared to the $\mathcal{E}$ and $\mathcal{P}$ spaces (see Fig.15(a/b/c)). Specifically, the $\mathcal{H}$ space is more sensitive to structural differences (as indicated by the fluctuating curve in Fig.15(d)). Additionally, its temporal difference values are not only more stable than those in the $\mathcal{P}$ space (as shown by the smooth curve in Fig.15(e)) but are also associated with lower false negative and false positive rates.

However, these three embedding spaces all reveal a common phenomenon: around the 1100 time period, there are mislabeled anomalies in the predicted anomaly regions. This difference is particularly prominent in the temporal structure differences. However, it is worth noting that from the perspective of data reconstruction, this region does not have any abnormal characteristics. This phenomenon offers two insights: on the one hand, it validates the exceptional ability of the $\mathcal{H}$ space to efficiently capture temporal dependency anomalies, enabling the timely identification of

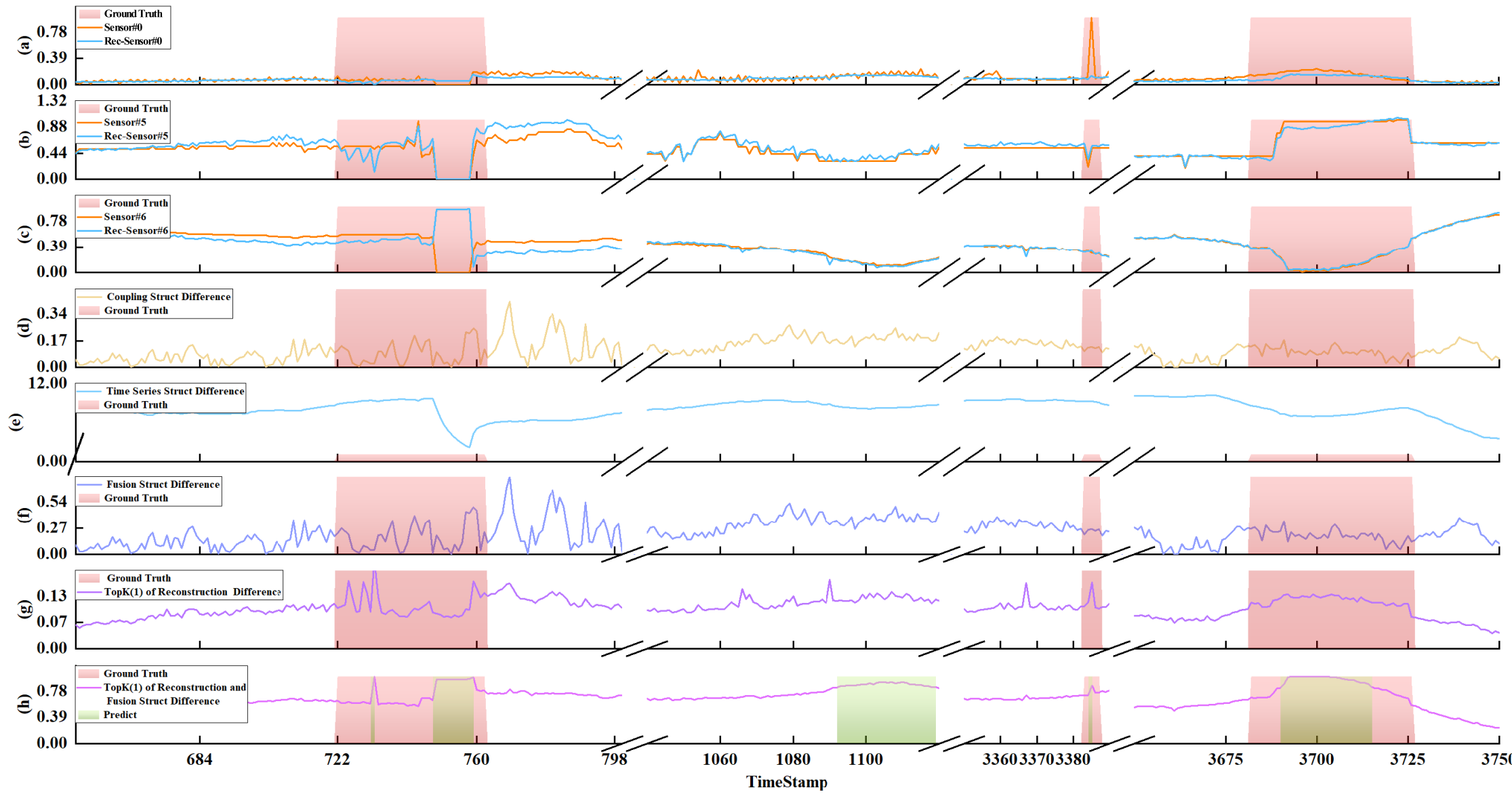

Figure 14: Anomaly Source Analysis in $\mathcal{P}$ Space.

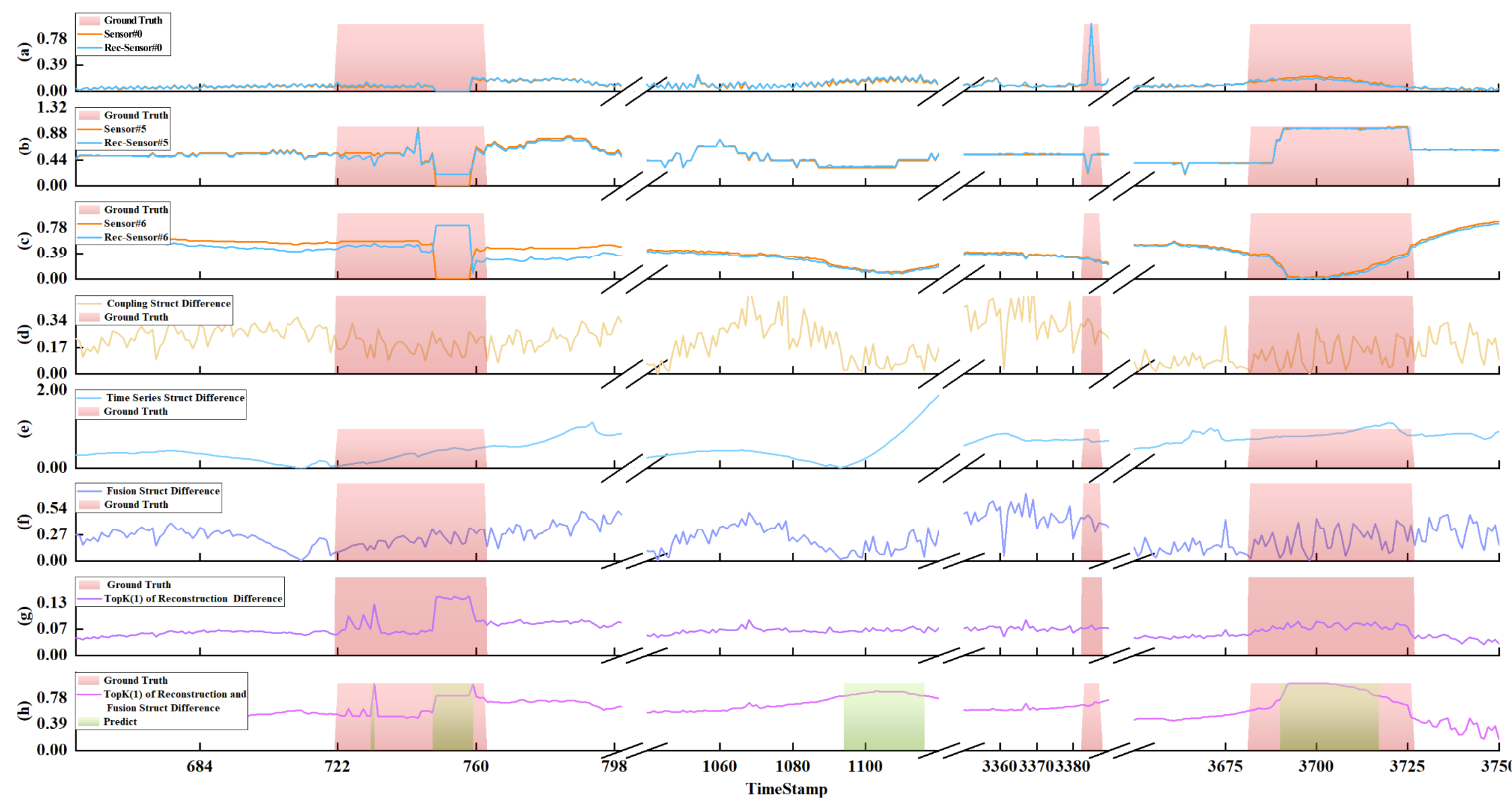

Figure 15: Anomaly Source Analysis in $\mathcal{H}$ Space.

potential issues; on the other hand, it highlights the effectiveness of the HAO(P) model in identifying weak anomalies (such as the transient mutations observed near 3380) and diagnosing potential mislabeling of anomalies.

8. The manuscript contains numerous abbreviations. So it is recommended that the authors include a table listing all abbreviations along with their full forms to improve readability and accessibility for a broader audience.

**Response 3.8 :** Thank you for your valuable suggestion to improve the readability and comprehensibility of the text by listing all abbreviations and their full names in an appendix. We fully agree with the value of this suggestion in serving a wider readership and make the necessary additions accordingly (see 3. HAO Model) to provide readers with a more user-friendly reading experience.

**Revision 3.8 :** **[(Page 13) 3.1 Symbol Conventions]** In order to improve the readability and comprehensibility of this paper, we have standardized the symbols and component abbreviations used in the text. Specific conventions are listed in Table 4.

Table 4: Notation Summary.

| No. | Symbol | Meaning |
|---|---|---|
| 1 | $\mathcal{E}$ | Euclidean space. |
| 2 | $\mathcal{P}$ | Poincaré ball space (a type of hyperbolic space). |
| 3 | $\mathcal{H}$ | Hyperbolic space. |
| 4 | $s$ | Multivariate coupled correlation perspective. |
| 5 | $t$ | Multivariate temporal dependency perspective. |
| 6 | $\tau$ | Multivariate anomaly detection threshold; $\tau_{best}$ denotes the optimal threshold. |
| 7 | $\mathbf{X} \in \mathbb{R}^{T \times N}$ | Multivariate time series data matrix, where $T$ is the observation duration and $N$ is the number of sensors or monitoring points. |
| 8 | $\mathcal{X} \in \mathbb{R}^{T \times W \times N}$ | Multivariate data representation sampled using a sliding window (of length $W$). |
| 9 | $\{S_i^{\mathcal{M}_\zeta}\}_{i=t-W}^{t-1}$ | Processed data sequence within the time window $[t-W, t-1]$, represented in the universal manifold space $\mathcal{M}_\zeta$. |
| 10 | $\mathcal{M} \in \{\mathcal{E}, \mathcal{P}, \mathcal{H}\}$ | Universal manifold space representation (covering Euclidean, Poincaré ball, and hyperbolic spaces). |
| 11 | $\mathbf{W}^{\mathcal{M}_\zeta}, \boldsymbol{x}^{\mathcal{M}_\zeta}$ | Matrix and vector representations in the universal manifold space $\mathcal{M}_\zeta$. |
| 12 | $\mathcal{D} \in \{s, t\}$ | Multivariate integrated perspective representation ($s$ for coupled correlation, $t$ for temporal dependency). |
| 13 | $K = -\frac{1}{\zeta^2}$ | Curvature of the universal manifold space $\mathcal{M}_\zeta$. |
| 14 | $\mathcal{T}_{\boldsymbol{x}}\mathcal{M}, \mathcal{M} \in \{\mathcal{E}, \mathcal{P}, \mathcal{H}\}$ | Tangent space of the universal manifold space $\mathcal{M}$ at point $\boldsymbol{x}$. |
| 15 | $g_{\boldsymbol{x}}^{\mathcal{M}}, \mathcal{M} \in \{\mathcal{E}, \mathcal{P}, \mathcal{H}\}$ | Riemannian metric of the universal manifold space $\mathcal{M}$ at point $\boldsymbol{x}$. |
| 16 | $\mathcal{M}_\zeta^n, \mathcal{M} \in \{\mathcal{E}, \mathcal{P}, \mathcal{H}\}$ | A specifically instantiated $n$-dimensional manifold space with curvature $K = -1/\zeta^2$. |
| 17 | $d^{\mathcal{M}_\zeta}(\boldsymbol{x}, \boldsymbol{y}), \mathcal{M} \in \{\mathcal{E}, \mathcal{P}, \mathcal{H}\}$ | Distance between points $\boldsymbol{x}$ and $\boldsymbol{y}$ in the manifold space $\mathcal{M}_\zeta$. |
| 18 | $\log_{\boldsymbol{x}}^{\mathcal{M}_\zeta}(\boldsymbol{y}), \mathcal{M} \in \{\mathcal{E}, \mathcal{P}, \mathcal{H}\}$ | The logarithmic map at base point $\boldsymbol{x}$, mapping point $\boldsymbol{y}$ on the manifold space $\mathcal{M}_\zeta$ to the tangent space $\mathcal{T}_{\boldsymbol{x}}\mathcal{M}$. |
| 19 | $\exp_{\boldsymbol{x}}^{\mathcal{M}_\zeta}(\boldsymbol{v}), \mathcal{M} \in \{\mathcal{E}, \mathcal{P}, \mathcal{H}\}$ | The exponential map at base point $\boldsymbol{x}$, mapping vector $\boldsymbol{v}$ in the tangent space $\mathcal{T}_{\boldsymbol{x}}\mathcal{M}$ back to the manifold space $\mathcal{M}_\zeta$. |
| 20 | $P_{\boldsymbol{x} \to \boldsymbol{y}}^{\mathcal{M}_\zeta}(\boldsymbol{v})$ | Vector transport in the manifold space $\mathcal{M}_\zeta$, moving vector $\boldsymbol{v}$ from point $\boldsymbol{x}$ to point $\boldsymbol{y}$. |
| 21 | $\oplus^\zeta, \otimes^\zeta$ | Generalized addition and multiplication operations in the manifold space $\mathcal{M}_\zeta$. |
| 22 | $\sigma^{\otimes^{\mathcal{M}_{\zeta_{l-1}, \zeta_l}}}$ | Non-linear activation function in the manifold space $\mathcal{M}_\zeta$, where $l$ denotes the model layer number (connecting layers with curvatures $\zeta_{l-1}$ and $\zeta_l$ respectively). |
| 23 | $\mathcal{A}^{\mathcal{M}_{\zeta, \mathcal{D}}} \in \{s, t\}, \mathcal{M} \in \{\mathcal{E}, \mathcal{P}, \mathcal{H}\}$ | Natural structures identified under the multivariate integrated perspective $\mathcal{D} \in \{s, t\}$ in the manifold space $\mathcal{M}_\zeta$. For simplicity, when the manifold space is fixed, it is also denoted as $\mathcal{A}_{\mathcal{D}}$. |
| 24 | $A_t^{\mathcal{M}_{\zeta, \mathcal{D}}}, \mathcal{D} \in \{s, t\}, \mathcal{M} \in \{\mathcal{E}, \mathcal{P}, \mathcal{H}\}$ | Local structure of the multivariate integrated perspective $\mathcal{D}$ in the manifold space $\mathcal{M}_\zeta$ at time $t$. |
| 25 | $\tilde{A}_{rc}^{\mathcal{M}_{\zeta, \mathcal{D}}}, \mathcal{D} \in \{s, t\}, \mathcal{M} \in \{\mathcal{E}, \mathcal{P}, \mathcal{H}\}$ | Anomaly root cause relationships identified under the multivariate integrated perspective $\mathcal{D}$ in the manifold space $\mathcal{M}_\zeta$. |
| 26 | $G_t^{\mathcal{M}_{\zeta, \mathcal{D}}}(V_t^{\mathcal{M}_{\zeta, \mathcal{D}}}, E_t^{\mathcal{M}_{\zeta, \mathcal{D}}}, S_t^{\mathcal{M}_{\zeta, \mathcal{D}}}), \mathcal{D} \in \{s, t\}, \mathcal{M} \in \{\mathcal{E}, \mathcal{P}, \mathcal{H}\}$ | Graph representation of the local structure under the multivariate integrated perspective $\mathcal{D}$ in the manifold space $\mathcal{M}_\zeta$ at time $t$, consisting of vertex set $V_t$, edge set $E_t$, and node features $S_t$. |
| 27 | $M^{\mathcal{M}_{\zeta, \mathcal{D}}} \in \{s, t\}, \mathcal{M} \in \{\mathcal{E}, \mathcal{P}, \mathcal{H}\}$ | Physical constraint matrix under the multivariate integrated perspective $\mathcal{D}$ in the manifold space $\mathcal{M}_\zeta$, used to suppress illegal connections. |
| 28 | $\mathcal{D} - \text{HDNN}(\cdot), \mathcal{D} \in \{s, t\}$ | Multiview linear transformation layer under the universal manifold space. |
| 29 | $\mathcal{D} - \text{HGCN}(\cdot), \mathcal{D} \in \{s, t\}$ | Multiview attention graph convolution layer under the universal manifold space. |
| 30 | $\text{HSD}(\cdot)$ | Multiview harmonic structure difference calculation under the universal manifold space. |

# REVISION OF MANUSCRIPT IN NATURE COMMUNICATIONS #NCOMMS-24-82438A

## Explanation of Revisions

We are submitting a revised version of our paper, "Hyperbolic Adaptive Spatial Perception Multivariate Time Series Anomaly Detection" (ID: #NCOMMS-24-82438A). We sincerely thank the reviewers for their valuable and constructive comments. We thoroughly revise the paper in accordance with these suggestions, provide detailed responses to all review comments and add new analytical content to further strengthen our research. We hereby declare that this paper has not been published elsewhere in any form.

In order to ensure that the revisions made in response to editorial comments and feedback from reviewers are clear, standardized and easy to review, the following is an explanation of how responses and revisions are marked:

- **Reviewer comment presentation:** We strictly quote the original comments of the reviewers and highlight them in black. For example: **The manuscript introduces the Hyperbolic Adaptive Spatial-Aware...**
- **Response Marking of responses to comments:** For each review comment, our response is uniformly marked in blue bold underlined format, labeled as: **Response 1 :**.
- **Revision Notes:** All modifications made in the text, whether they involve content adjustments or wording optimizations, will be marked using red bold underlined formatting, identified as: **Revision 1 :** Along with a brief explanation of the key points of the revision.
- **Location of Revisions:** To help reviewers quickly find the exact locations of the changes, we have marked the section titles and page numbers at the relevant modification points in red bold text, using the format: **[Section Title (Revised File: Page Number)]**, for example: **[Section 2.2.4 Hyperbolic Graph Representation Learning (Main Text: Page 7)]**.

## 1 Reviewer #1 (Remarks to the Author):

**Thank authors' response one by one for the comments. But these problems need to be solved before considering publication.**

**1. There are inconsistent representations in the revised manuscript, such as "To the best of our knowledge, the hyperbolic pre-training framework, adaptive hyperbolic graph structure learning, cross-region anomaly interpretation," in the ABSTRACT section, etc.**

**Response 1:** We greatly appreciate the reviewer's careful attention to detail and for pointing out the inconsistencies in terminology throughout the revised manuscript. This issue is specifically manifested in the following aspects:

**In the Abstract (Page 1)**, the statement is formulated as follows: *"...To the best of our knowledge, the hyperbolic adaptive pre-training framework, adaptive hyperbolic graph structure learning, cross-region anomaly interpretation and the small-sample dynamic mixed anomaly threshold determination algorithm are proposed and applied to the multivariate anomaly detection task for the first time..."*. However, inconsistencies were identified in the corresponding sections of the main text, where the technical terms and expressions were not fully consistent. **Section 1 Introduction (Main Text: Page 2)**: *"...an adaptive hyperbolic pre-training framework..."*, **Section 3 Methodology (Main Text: Page 11)**: *"...adaptive hyperbolic graph structure discovery... a small-sample dynamic mixed anomaly threshold determination algorithm..."*, **Section 4.3 Multivariate Anomaly Recognition (Main Text: Page 27)**: *"...small-sample dynamic threshold determination algorithm..."*.

In response to the aforementioned issue, we have unified the key terminology throughout the manuscript and conducted a comprehensive consistency check across the entire text. Furthermore, combined with further refinements to our methodological framework, we have standardized and supplemented the core innovations presented in the abstract. The revised version reads as follows:

*"...To the best of our knowledge, the proposed adaptive hyperbolic pre-training framework, adaptive hyperbolic graph structure discovery, multivariate anomaly spatio-temporal coordinate localization, multi-level signal-parsing attention mechanism and small-sample hybrid dynamic threshold determination algorithm are all proposed for the first time and successfully applied to multivariate anomaly detection..."*

We have also thoroughly proofread and unified the corresponding content in the main text to ensure consistent and rigorous expression throughout the manuscript.

**Revision 1:** **[ABSTRACT (Main Text: Page 1)]** *"...To the best of our knowledge, the proposed adaptive hyperbolic pre-training framework, adaptive hyperbolic graph structure discovery, multivariate anomaly spatio-temporal coordinate localization, multi-level signal parsing attention mechanism and small-sample hybrid dynamic threshold determination algorithm are all introduced here for the first time and successfully applied in this domain..."*

**[(Main Text : Page 1 - 42), (Appendix: Page 1 - 10)]** We have conducted a unified review of both the main text and appendix, with the checklist provided in Table 1.

Table 1: Key Terminology Checklist

| No. | Key Terminology | Main Text | Appendix |
|---|---|---|---|
| 1 | *Adaptive Hyperbolic Graph Structure Discovery* | ✓ | ✓ |
| 2 | *Adaptive Hyperbolic Pre-training Framework* | ✓ | ✓ |
| 3 | *Multivariate Anomaly Spatio-Temporal Coordinate Localization* | ✓ | ✓ |
| 4 | *Multi-level Signal Parsing Attention Mechanism* | ✓ | ✓ |
| 5 | *Small-sample Hybrid Dynamic Threshold Determination Algorithm* | ✓ | ✓ |

**2. There are format errors in the manuscript, such as the first author affiliation is not labeled in the manuscript, "coupling correlation relationship describes in the 9th page", etc.**

**Response 2:** We are sincerely grateful to the reviewer for the insightful and valuable comments. In response to the formatting issue regarding the unclear indication of the first author's affiliation in the revised manuscript, we have corrected and optimized the display of the first author's institutional information. Additionally, we have refined and supplemented the relevant expression on page 9, namely "*...coupling correlation relationship describes...*", to enhance its clarity and accuracy.

**Revision 2:** **[ABSTRACT (Main Text: Page 1)]** We have optimized the display format of the first author's institutional information to ensure that the details of each author are presented completely and clearly.

**[Section 2.3 Multivariate Anomaly Cause Interpretation (Main Text : Page 1)]** We have refined the overall section on the interpretation of multivariate anomaly causes. Specifically, the revised content now includes: *...Temporal dependency relationships describe the interactions between the operating states of a system at different time points...*, *...Coupling relationships describe the mutual interactions between different variables. Compared with temporal dependency relationships, coupling relationships generally exhibit weaker directional characteristics....*

**3. The whole language representation needs to be improved.**

**Response 3:** We are sincerely grateful to the reviewer for the insightful and valuable comments. In response to the concern regarding the need for improved language expression in the revised manuscript, we have conducted a more meticulous and rigorous refinement of the writing conventions and technical terminology throughout the entire text to enhance clarity and professionalism.

**Revision 1:** **[Main Text (Page 1 - 42), Appendix (Page 1 - 10)]** We have further polished and refined the linguistic expressions throughout both the main text and the appendix, with the aim of ensuring enhanced clarity, logical coherence and academic rigor across the entire manuscript.

**4. Some unimportant sections should be removed in the manuscript, or they are represented by the Appendix section. This enables the research work focus on key contents.**

**Response 4:** We sincerely appreciate the reviewer's valuable feedback. Regarding the issue that non-essential content in the revised manuscript has distracted from the presentation of our core arguments, we have moved this appendix to the appendix. This revision enables the manuscript to center more closely on our key contributions and primary research findings.

**Revision 4:** **[Section 3.1 Symbol Definitions; Section 3.2.2 Relevant Theorems and Lemmas; Section 4.1 Dataset,; Section 4.2 Table Confusion Matrix; Section 4.3 Experimental Setup; Section 4.6.5 Case Stydy]** We have presented this content in the form of an appendix.

## 2 Reviewer #3 (Remarks to the Author):

**1. From a system design and methodological integration standpoint, this paper presents a conceptually well-organized framework with potential for practical value. However, from the standpoint of theoretical depth or transformative methodological innovation, the work does not appear to reach a level of breakthrough that would justify publication in a high-impact venue such as Nature Communications. Additionally, its current implementation and experimental setup raise concerns about the validity and generalizability.**

**Response 1:** We sincerely appreciate the reviewer's recognition of the systematic design and method integration in this work, as well as the reviewer's valuable comments and incisive insights concerning the theoretical depth, methodological innovation and reliability of the experimental results. To directly address these core concerns, we have carried out systematic upgrades and comprehensive revisions to our research methodology and experimental validation, which has notably strengthened the theoretical originality, result validity and generalizability of the proposed approach. Detailed modifications and corresponding responses are presented below.

**Response to Concerns Regarding Theoretical Depth and Methodological Breakthrough.** We sincerely appreciate the reviewer's constructive comments on the theoretical depth and methodological innovation of this work. In response to the concern that the original manuscript lacks sufficient theoretical depth and methodological breakthroughs, we have systematically refined and strengthened the theoretical framework across the three core tasks of multivariate anomaly detection, recognition, diagnosis and interpretation. Through deep analysis, we identify a critical bottleneck in current research: label noise, which is widespread in real-world multivariate anomaly datasets and often leads to over-optimistic and inflated detection performance. In addition, existing annotations are mostly timestamp-based, where entire time windows are marked as anomalous once an event occurs. Such coarse labeling makes it difficult to support fine-grained anomaly interpretation and reliable model validation, which further restricts the application of mainstream models to complex real-world multivariate anomaly detection tasks [1]. A comprehensive review of recent studies [1, 2, 4, 5] indicates that mature and robust solutions for label noise in multivariate anomaly detection remain absent. Although this issue was briefly mentioned in the second-round revision, deep analysis and targeted solutions were not fully developed at that time.

In the current revised version, we have conducted a systematic investigation and proposed a complete solution. First, we enhanced the adaptive hyperbolic natural structure discovery method and achieved anomaly label correction and fine-grained annotation based on structural differences. This key breakthrough bridges the long-standing gap between anomaly detection and interpretation, establishing an end-to-end theoretical loop from anomaly detection to causal attribution. Furthermore, we performed cross-domain structural difference analysis, which verifies the transferability and generalization ability of the proposed method across diverse application scenarios. We also improved the anomaly cause explanation graph, providing a reliable benchmark for anomaly interpretation and overcoming the limitations of traditional weight-based interpretation mechanisms. The proposed method can accurately capture the spatio-temporal distribution and propagation paths of anomalies. Equipped with an open and adaptive explanation generalization mechanism, the model maintains stable interpretability, credibility and traceability even in the presence of unseen or novel anomalies. This work provides valuable guidance for promoting industrial cognitive intelligence from simple anomaly recognition toward deep causal insight, thereby significantly improving the reliability of decision-making under complex operating conditions. Through the above improvements, we have directly and thoroughly addressed the reviewer's concerns regarding theoretical depth and methodological innovation.

**Response to Concerns Regarding Experimental Reliability.** In response to the concerns raised regarding the reliability of the experimental results, we have conducted more comprehensive and rigorous experiments covering the full pipeline of multivariate anomaly detection, recognition, diagnosis and interpretation. We have also optimized the construction strategies and descriptive details of the experimental datasets. For the anomaly recognition experiments, we evaluated the model performance before and after label correction, verifying an overall performance improvement of 8%. This optimization effectively alleviates the issue of metric inflation caused by label noise. Additionally, we supplemented the experimental validation with ROC curves and P-R curves to further demonstrate the effectiveness of the proposed method. We performed ablation studies and stability tests to analyze the individual contribution of each key module in the adaptive hyperbolic natural structure discovery method. Moreover, t-tests were conducted to confirm the statistical significance of performance differences across diverse data representation spaces, with a p-value of less than 0.05. Comprehensive comparative analyses were carried out in terms of reconstruction error, memory consumption and inference time, which prove that our model surpasses existing baseline methods in overall performance. We also completed hyperparameter analysis to investigate the impacts of dataset split ratio, sliding window size, anomaly detection algorithm selection and adaptive curvature adjustment on the training stability of the model. For the anomaly diagnosis experiments, targeting the problem of insufficient fine-grained annotations in existing datasets, we supplemented the evaluation metrics with HitRate and NDCG. The experimental results indicate that our model achieves a HitRate of over 93% and an NDCG of over 89%, significantly outperforming all baseline methods.

We further analyzed the advantages and limitations of different data representation spaces in anomaly labeling, which provides additional validation for the reliability of our proposed label correction scheme. For the anomaly interpretation experiments, we compared the structural parsing performance of the model across different data representation spaces, explored the spatio-temporal structural differences and consistencies inherent in multivariate data, constructed detailed anomaly explanation graphs and summarized typical anomaly patterns. Furthermore, we added real-world industrial case studies, which illustrate that the model can accurately identify and locate critical anomaly events in practical application scenarios.

Through these multi-dimensional and systematic improvements to the experimental design and validation procedures, we have substantially enhanced the rigor, reproducibility and reliability of our experimental results.

## References


[1] Wu, R. J. and Keogh, E. J. Current time series anomaly detection benchmarks are flawed and are creating the illusion of progress. *IEEE T. Knowl. Data En.* **35**, 2421–2429 (2021).

[2] Zhang, C. et al. A deep neural network for unsupervised anomaly detection and diagnosis in multivariate time series data. In *Proc. AAAI Conf. Artif. Intell.* **33**, 1409–1416 (2019).

[3] Tang, C., Xu, L., Yang, B., Tang, Y. & Zhao, D. GRU-based interpretable multivariate time series anomaly detection in industrial control system. *Comput. Secur.* **127**, 103094 (2023).

[4] Huang, X. H. et al. Multivariate time series anomaly detection via dynamic graph attention network and Informer. *Appl. Intell.* **54**, 7636–7658 (2024).

[5] Zhang, W. et al. A graph attention network-based model for anomaly detection in multivariate time series. *J. Supercomput.* **80**, 8529–8549 (2024).


**Revision 1:** **[Section 1 (Main Text: Page 2)]**...*The credibility issue in multivariate anomaly labelling [1] primarily stems from prevalent omission and mislabelling during manual labeling, as shown in Fig. 2. Omission manifests at three levels...*

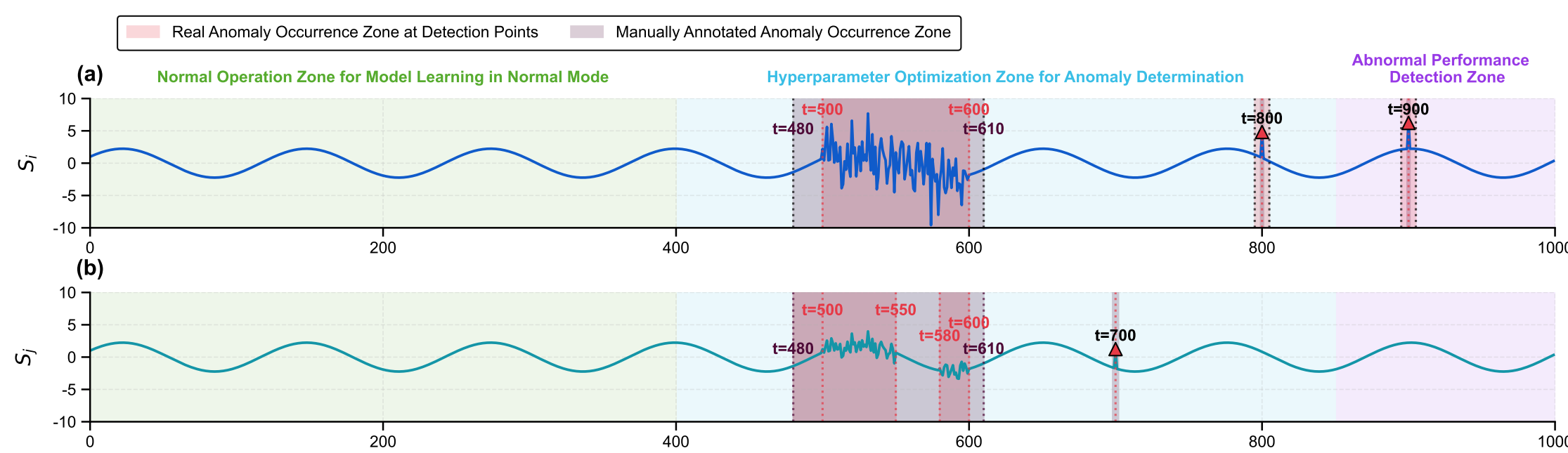


Figure 1: ***Risk Analysis of Anomaly Label Reliability.*** *1. Mislabelling of anomaly occurrence regions: Limited by retrospective estimation of anomaly onset, it is often difficult to pinpoint the exact moment starting moment, resulting in annotated anomaly regions that frequently exceed the true anomalous interval. For example, as shown in Fig. 2(a), the actual anomaly occurs within $t = 500 \rightarrow 600$, yet the labelled range extends from $t = 480 \rightarrow 610$. In practice, such temporal deviations may be even more pronounced. Mislabelling may also manifest as segments appearing normal at small time scales but being labelled as anomalous over larger scales. As shown in Fig.2(b), the interval $t = 550 \rightarrow 580$ is in fact normal, yet it is included within the annotated anomalous range $t = 480 \rightarrow 610$. 2. Omission of anomaly regions: While obvious point-level anomalies are generally annotated correctly, subtle point-level anomalies that are difficult to perceive are prone to omission. For instance, as shown in Fig. 2(a), anomalies at $t = 800$ and $t = 900$ have been labelled, whereas a similar anomaly at $t = 700$ has been overlooked. Both mislabelling and insufficient labelling in anomaly labeling not only compromise objective performance assessment of high-precision anomaly detection models, but the misleading labels can further degrade the ability of even high-performance models to correctly identify anomalies.*

**[Section 4 (Main Text : Page 23-42)]** We have separately supplemented targeted experiments for the three core components of the multivariate anomaly detection task: anomaly recognition, anomaly diagnosis and anomaly interpretation.

**[Section 4 (Appendix: Page 3-8)]** We have separately refined the descriptions of the dataset, comparative baseline models and training data construction strategy for the multivariate anomaly detection task.

**2. According to the description of "Data set division" on page 25, the experimental setup appears to have some critical limitations that warrant closer examination. The training set consists solely of normal data, while the test**

**and validation sets are constructed from artificially partitioned and recombined abnormal samples. This process fragments originally continuous abnormal events into isolated points or patterns, which are then distributed across different subsets. Such manipulation severely disrupts the contextual integrity of the anomalies and impairs the model's ability to learn the temporal dynamics and structural patterns associated with real-world anomalies. More critically, this partitioning strategy breaks the chronological order of the time series, thereby violating the core assumption of time series modeling — that models are trained on past data to predict or detect future events. As a result, the evaluation no longer reflects a realistic deployment scenario and fails to simulate the continuity and temporal causality of anomaly occurrences in practical applications.**

**Response 2:** We sincerely appreciate the reviewer for identifying the critical issues concerning dataset partitioning in our experimental setup. The reviewer's rigorous and meticulous review has offered invaluable guidance for improving the scientific rigor and rationality of this study. In response to the reviewer's constructive comments regarding the training set consisting solely of normal data, the segmentation of continuous anomaly events within the test and validation sets, as well as the potential violation of the temporal order assumption, we have supplemented corresponding experimental evidence and detailed explanations based on the design principles of the sliding window sampling mechanism and random sampling strategy, as detailed below:

1. To address the issue of preserving the contextual integrity of anomaly events, this study adopts a sliding window sampling mechanism [1], which adaptively adjusts the window size based on practical application scenarios. This method effectively avoids the fragmentation of continuous anomaly events. During the data preprocessing stage, we implement continuous sliding sampling on the original multivariate time series data, which ensures that each data sample block retains complete contextual information related to anomalies, thus preserving the temporal structure and evolutionary patterns of anomalous events.

2. To alleviate concerns regarding the temporal order assumption, this study utilizes sliding window sampling to implement continuous sequential sampling, which strictly follows the chronological order of the raw data. Meanwhile, the training, validation and test sets are partitioned along clear and strict temporal boundaries. There is no overlap or intersection across the three subsets in the time dimension. This partitioning protocol is fully consistent with the common standards and methodological guidelines for time series modeling and deep learning research [2].

3. During the model parameter learning phase, we adopt a random sampling strategy for training optimization. This strategy is designed to prevent the model from learning spurious patterns induced by fixed sample ordering, while boosting the diversity of the training data distribution. In turn, this improves the model's generalization capability and robustness in real-world scenarios characterized by concept drift.

In summary, the dataset partitioning strategy adopted in this study strictly conforms to the core assumptions of time series modeling. Sliding window sampling ensures the contextual integrity of anomalies. We appropriately designed random sampling to improve the model's generalization and robustness. The higher accuracy and stability of the experimental results further verify the rationality and effectiveness of the experimental setup. Once again, we sincerely thank the reviewer for these insightful suggestions. In the revised manuscript, we have supplemented detailed specifications regarding the temporal boundary definitions for dataset partitioning and added quantitative analyses of the sampling strategies, which further enhance the transparency and persuasiveness of the experimental design.

## References


[1] Zheng, Y. et al. Correlation-aware spatial–temporal graph learning for multivariate time-series anomaly detection. *IEEE T. Neur Net. Lear. Syst.* **35**, 11802–11816 (2024).

[2] LeCun, Y., Bengio, Y. & Hinton, G. Deep learning. *Nature* **521**, 436–444 (2015).


**Revision 2:** **[Section 2.3 Multivariate Anomaly Cause Interpretation (Main Text: Page 10) ]**The working principle of the sliding window sampling mechanism is shown by the red markings in Fig. 2.

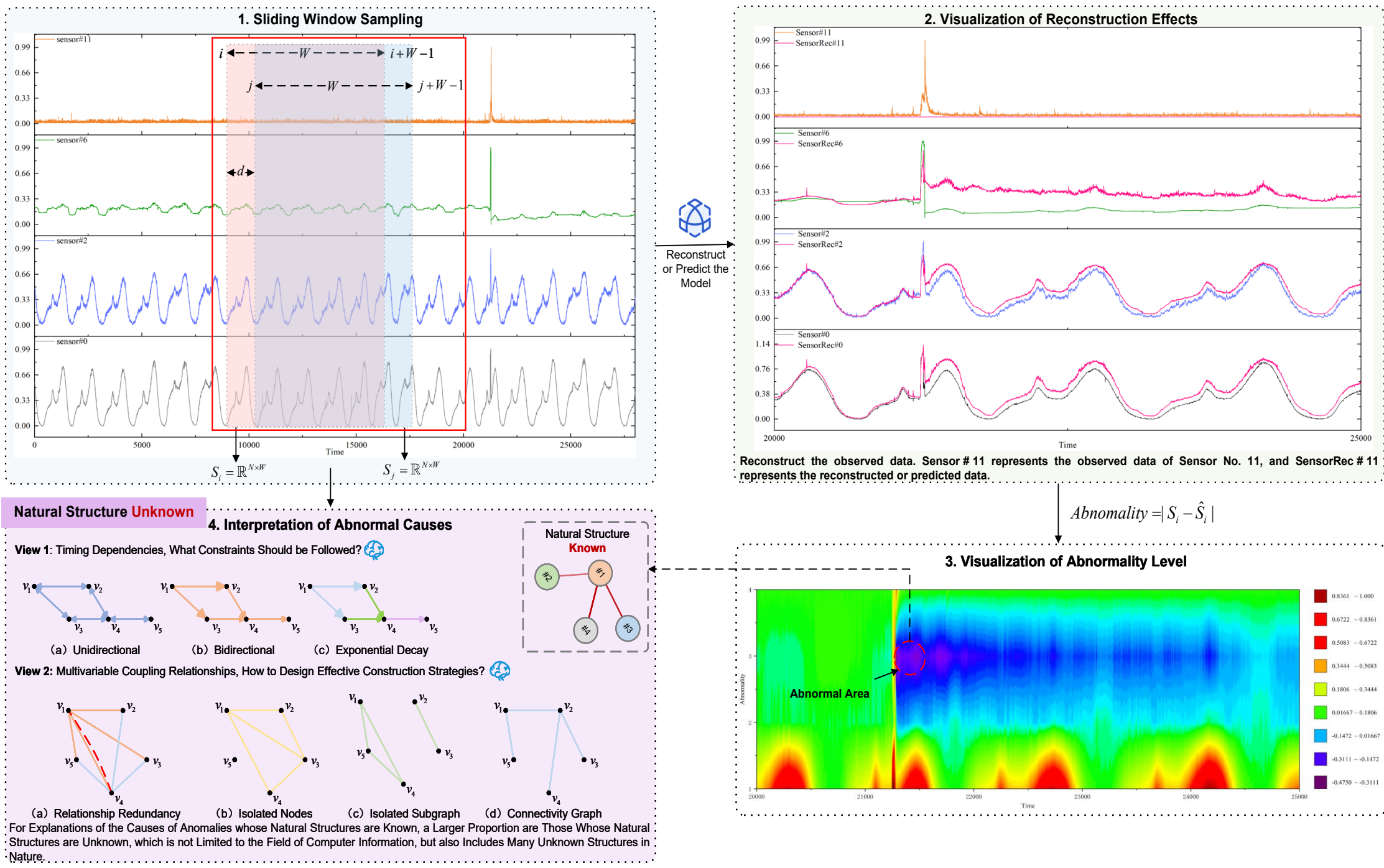


Figure 2: ...***Multivariate Anomaly Causation Interpretation Workflow [1, 2, 3, 4].*** *Data preprocessing stage: The sliding window ($W$) technique is adopted to perform sliding sampling on monitoring data, where the sampling interval ($d$) is selected to ensure the continuity of data processing. Sampling is continuous when $d = 1$ and discrete when $d > 1$...*

**[Section 4.1 Data Preprocessing (Appendix: Page 5) ]** *...to enhance the model's ability to capture latent patterns in the data and ensure the stability of the training process, this study sequentially performs data sampling and normalization operations. To preserve the complete contextual information around the moment an anomaly occurs, a sliding window strategy is adopted to sample the observed data. Specifically, the original observation data $X \in \mathbb{R}^{N\times T}$ is continuously sampled by sliding a window of size $W$, resulting in a dataset $\mathcal{X} \in \mathbb{R}^{T\times N\times W}$ that contains the anomaly context at each time step, with each data element $S_i \in \mathcal{X}$ serving as the basic unit for the model to learn anomaly patterns...*

**[Section 4.2 Dataset Partitioning (Appendix: Page 6) ]** *...the training dataset contains monitoring data of the observation targets under normal operating modes, while the test dataset covers scenarios with unknown system operating states, including both normal and anomalous mode data...*

## References


[1] Deng, A. & Hooi, B. Graph neural network-based anomaly detection in multivariate time series. In *Proc. AAAI Conf. Artif. Intell.* **35**, 4027–4035 (2021).

[2] Tang, C., Xu, L., Yang, B., Tang, Y. & Zhao, D. GRU-based interpretable multivariate time series anomaly detection in industrial control system. *Comput. Secur.* **127**, 103094 (2023).

[3] Zhao, H. et al. Multivariate time-series anomaly detection via graph attention network. In *Proc. IEEE Int. Conf. Data Min. (ICDM)*, 841–850 (2020).

[4] Tian, Z. et al. Anomaly detection using spatial and temporal information in multivariate time series. *Sci. Rep.* **13**, 4400 (2023).


**3. Although the proposed method is evaluated on 16 benchmark datasets, the results do not show a clear performance advantage in terms of the key evaluation metric, F1-score. More importantly, in many real-world applications such as security alerting, fault diagnosis and medical anomaly detection, missed detections (false negatives) are often more critical than false alarms. In such contexts, Recall becomes a crucial metric. Unfortunately, the proposed method also underperforms in Recall compared to existing baseline models on most datasets, further undermining its practical effectiveness and competitiveness.**

**Response 3:** We sincerely appreciate the reviewer's valuable comments regarding the experimental performance and practical applicability of our work. In response to the reviewer's observation that although the proposed method is evaluated on 16 benchmark datasets, it does not exhibit a clear performance advantage with respect to the key evaluation metric, namely the F1 score. Moreover, the reviewer pointed out that in many real-world applications such as security alerting, fault diagnosis and medical anomaly detection, missed detections are often more critical than false alarms, making recall a particularly crucial metric. However, our method underperforms in recall compared with existing baseline models on most datasets, which further limits its practical effectiveness and competitiveness. We have provided a detailed explanation in **Revision 1** regarding the fundamental reasons for the relatively low F1 score and recall. We attribute these shortcomings mainly to the severe label noise that is prevalent in multivariate anomaly data. To alleviate the performance degradation caused by label noise in multivariate anomaly detection, we have proposed a series of methodological innovations and conducted systematic experimental validation, as shown in Fig. 3. The content marked in red in Fig. 3 represents the new research added in this manuscript. This ensures that our model achieves the best performance across all 16 datasets. These improvements have led to a significant enhancement in the overall model performance, especially in recall, while further strengthening the applicability of the method in real-world scenarios. The specific responses and detailed revisions are presented below.

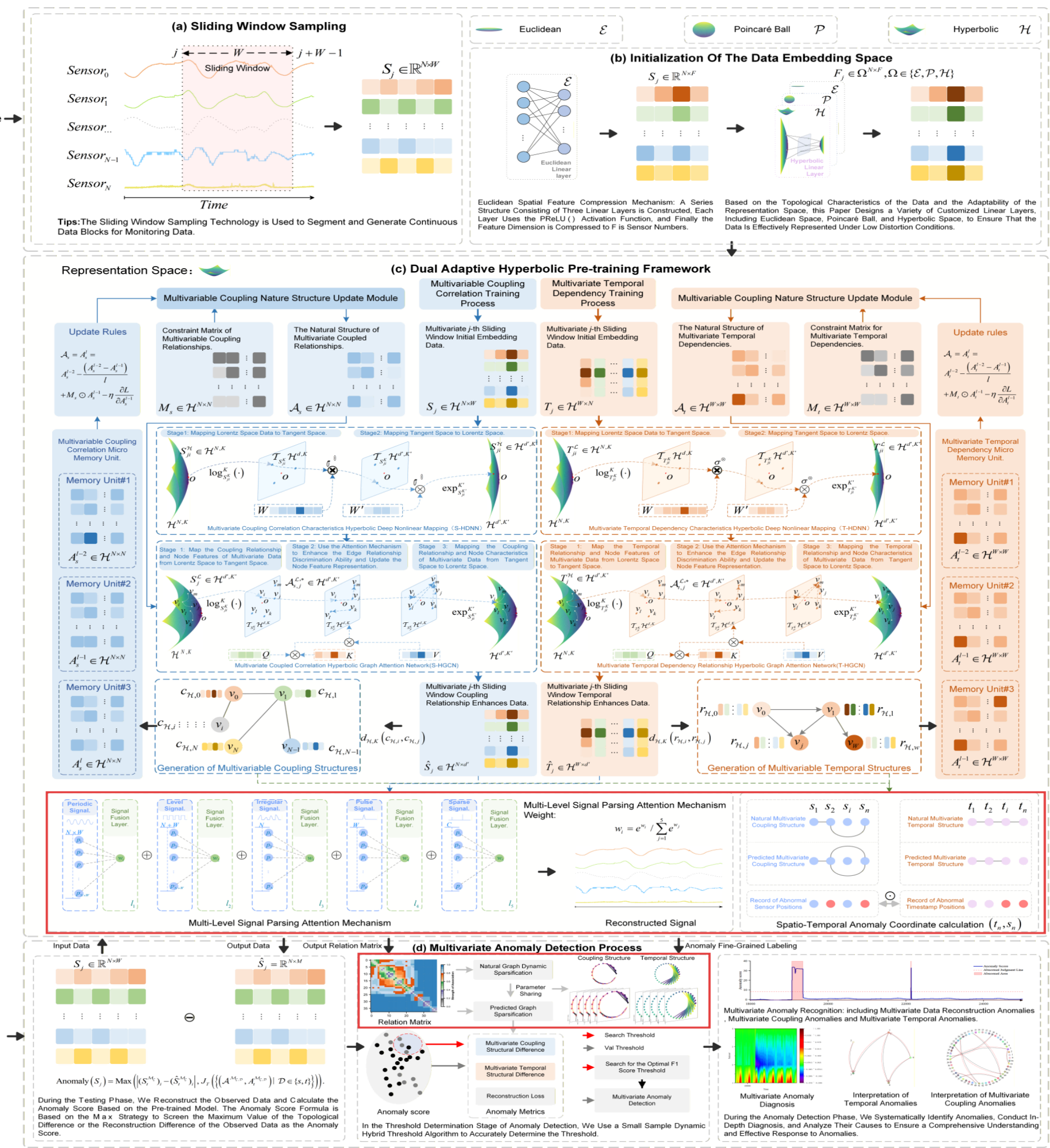

Figure 3: *Hyperbolic Adaptive Spatial-Aware Multivariate Temporal Anomaly Detection Model Framework*

First, to mitigate the adverse impact of label noise on multivariate anomaly detection performance, we propose a dynamic sparsification method for multivariate spatio-temporal natural graphs, coupled with a topology difference-based spatio-temporal coordinate localization algorithm tailored for multivariate anomalies. Specifically, the multivariate natural graph is dynamically sparsified in line with the requirements of downstream anomaly detection tasks. By

fully exploiting the synergistic effect between topological difference metrics and the physical constraint matrix, this approach adaptively filters out non-critical association edges, retaining the core structural information of the data while eliminating redundant and noisy connections. Subsequently, the proposed multivariate anomaly spatio-temporal coordinate localization algorithm is adopted to accurately locate anomalous spatio-temporal points, which corrects the raw noisy data labels. This strategy effectively alleviates the interference caused by label noise and further establishes a unified and reliable benchmark for multivariate anomaly recognition, diagnosis and interpretation.

Second, to tackle the problem of large reconstruction errors associated with sparse signals, we introduce a multi-level signal parsing attention mechanism that substantially boosts the signal reconstruction performance for diverse signal types. Compared with mainstream baseline models, the overall reconstruction error is reduced by 30%. In addition, this mechanism further enhances the stability and robustness of the mining process for multivariate spatio-temporal natural structures, laying a solid foundation for accurate subsequent anomaly detection and analysis.

In summary, through the aforementioned key methodological innovations, the proposed model achieves comprehensive performance enhancements, especially in resolving the critical issue of insufficient fine-grained annotations in multivariate anomaly tasks. Notably, our model attains state-of-the-art performance in multivariate anomaly diagnosis, rendering it better suited to meet the stringent demands of real-world practical applications. We once again express our sincere gratitude for the reviewer's constructive feedback, which has substantially elevated both the academic merit and practical application potential of this work.

**Revision 3:** **[Section 3 Methodology (Main Text: Page 12-13)]**...*In complex industrial systems and Internet of Things (IoT) scenarios, anomaly detection in multivariate time series data faces multiple challenges, including insufficient credibility of anomaly labels, unknown true system topology, distortion in the data embedding space and confusion among anomaly interpretation dimensions. To address these issues, a Hyperbolic Adaptive Spatial-Aware Framework is proposed for Multivariate Time Series Anomaly Detection, as shown in Fig. 3. By leveraging the hierarchical modelling capabilities of hyperbolic geometry and a dual adaptive hyperbolic pre-training framework, the framework enables the accurate recognition, diagnosis and interpretable analysis of multivariate anomalies...*

**[Section 3.2.5 Multi-Level Signal Parsing Attention Mechanism(MSCD) (Main Text: Page 18)]** *Due to the presence of complex and diverse signal forms in multivariate data such as periodic signals, level signals and sparse signals, traditional multilayer perceptrons struggle to effectively capture different signal features across multiple scales, leading to insufficient fitting performance with the original signals. To address this, a multi-level signal parsing attention mechanism is proposed, which dynamically adjusts the number of neurons according to the scale characteristics of the data block ($S_t \in \mathcal{M}^{N\times W}$), so as to accurately capture the data features of multivariate variables. The specific definition is as follows:*

$$\hat{S}_t = \sum_{l=1}^{L} w_l \times F_t, w_l = \frac{e^{w_l}}{\sum_{l=1}^{j} e^{w_j}}, \tag{1}$$

*where $L$ denotes the number of layers for signal parsing. The terms $w_1 \in \mathcal{M}^{N\times W}, w_2 \in \mathcal{M}^{N+W}, w_3 \in \mathcal{M}^{N}, w_4 \in \mathcal{M}^{W}$ and $w_5 \in \mathcal{M}^{C}$ represent constant scales determined by the dynamic perception mechanism of the multi-level signal parsing attention mechanism, used to set the number of neurons.*

**[Section 3.4.1 Topological Difference Metric (Main Text: Page 20-21)]** *By comparing the geometric deformation discrepancy between the system's natural topology ($\mathcal{A}$) and the predicted topology ($A$), anomalous relationships and associated sensor pairs can be identified. First, $\mathcal{A}$ and $A$ are sparsified and vectorised as follows:*

$$\mathcal{A}_{ij} = \begin{cases} \mathcal{A}_{ij} & if \mathcal{A}_{ij} \le T_{up} \\ 0 & otherwise \end{cases}, A_{ij} = \begin{cases} A_{ij} & if A_{ij} \le T_{up} \\ 0 & otherwise \end{cases}, \tag{2}$$

*where $T_{up}$ is the topological relationship judgment threshold, employed to dynamically filter those relationships that are beneficial for quantifying topological discrepancies, while also reducing the size and improving the efficiency of the candidate relationship set. Next, the sparsified topological matrices are transformed into vector form as follows:*

$$\begin{aligned} \overrightarrow{\mathcal{A}} &= [\mathcal{A}_{1,1}, \mathcal{A}_{1,2}, \cdots, \mathcal{A}_{1,W}, \mathcal{A}_{2,1}, \mathcal{A}_{2,2}, \cdots, \mathcal{A}_{2,W}, \cdots \mathcal{A}_{N,1}, \mathcal{A}_{N,2}, \cdots, \mathcal{A}_{N,W}], \\ \overrightarrow{A} &= [A_{1,1}, A_{1,2}, \cdots, A_{1,W}, A_{2,1}, A_{2,2}, \cdots, A_{2,W}, \cdots A_{N,1}, A_{N,2}, \cdots, A_{N,W}], \end{aligned} \tag{3}$$

*Finally, the geometric topological structures for comparison are selected based on the candidate relationships $K$:*

$$(C_{\mathcal{A}})_{ij} = \begin{cases} 1 & if \mathcal{A}_{ij} \in TopK(\overrightarrow{\mathcal{A}}, K) \\ 0 & otherwise \end{cases}, (C_{A})_{ij} = \begin{cases} 1 & if A_{ij} \in TopK(\overrightarrow{A}, K) \\ 0 & otherwise \end{cases}, \tag{4}$$

*where under $s$-relational topological comparison, $K = N$; under $t$-relational topological comparison, $K = W$. Based on the obtained topological relationships and inspired by the Jaccard similarity, a topological discrepancy metric*

*grounded in the Jaccard similarity is devised, defined as follows:*

$$J_T(C_{\mathcal{A}}, C_A) = 1 - \frac{|C_{\mathcal{A}} \cap C_A|}{|C_{\mathcal{A}} \cup C_A|}. \tag{5}$$

**[Section 3.4.2 Multivariate Anomaly Spatio-Temporal Coordinate Localization (Main Text: Page 21)]** *Multivariate anomaly spatio-temporal coordinate localization is achieved by pinpointing anomalous coordinates through topological deformation. Meanwhile, to mitigate the potential misguidance of model reconstruction performance on anomaly coordinate computation, joint multivariate anomaly spatio-temporal coordinate localization is performed using spatial coordinates ($L_{\boldsymbol{s}}$), temporal coordinates ($L_{\boldsymbol{t}}$) and reconstructed coordinates ($L_{\boldsymbol{r}}$). For a given data block $S_{t-W+1}^{t} \in \mathcal{M}^{N\times W}$, its potential fine-grained anomaly label is defined as $L = \mathbf{0}^{N\times W}$, as detailed below:*

$$\begin{gathered}
\boldsymbol{s} = \left\{ \lfloor N/\underset{s}{argmax}|\overrightarrow{\mathcal{A}_s} - \overrightarrow{A_s}|\rfloor, N\%\underset{s}{argmax}|\overrightarrow{\mathcal{A}_s} - \overrightarrow{A_s}| \right\}, L_{\boldsymbol{s}} = 1, \\
\boldsymbol{t} = \left\{ \lfloor W/\underset{t}{argmax}|\overrightarrow{\mathcal{A}_t} - \overrightarrow{A_t}|\rfloor, W\%\underset{t}{argmax}|\overrightarrow{\mathcal{A}_t} - \overrightarrow{A_t}| \right\}, L_{\boldsymbol{t}} = 1, \\
\boldsymbol{r} = \left\{ \underset{s,t}{argmax}|S_{t-W+1}^{t} - \hat{S}_{t-W+1}^{t}| \right\}, L_{\boldsymbol{r}} = 1, \\
L = L_{\boldsymbol{s}} \odot L_{\boldsymbol{t}} + L_{\boldsymbol{r}},
\end{gathered} \tag{6}$$

*where % denotes the modulo operation. $L_{\boldsymbol{s}}$ represents multivariate coupling anomaly labelling: Rows correspond to sensor pairs exhibiting anomalies, with the corresponding entry set to 1 when an anomaly occurs. $L_{\boldsymbol{t}}$ denotes multivariate temporal anomaly labelling: Columns correspond to time pairs exhibiting anomalies, with the corresponding entry set to 1 upon anomaly occurrence. $L_{\boldsymbol{r}}$ denotes multivariate data reconstruction anomaly labelling: Sensors at anomalous timestamps serve as anomalous coordinates, with the corresponding position set to 1 when an anomaly is present.*

**[Section 4.3.1 Comparative Experiments (Main Text: Page 23-24)]** *...Before correction, the F1-score of the HAO series models ranges from 0.8995 to 0.9112, which is significantly better than those of traditional models ranging from 0.5489 to 0.6640...*

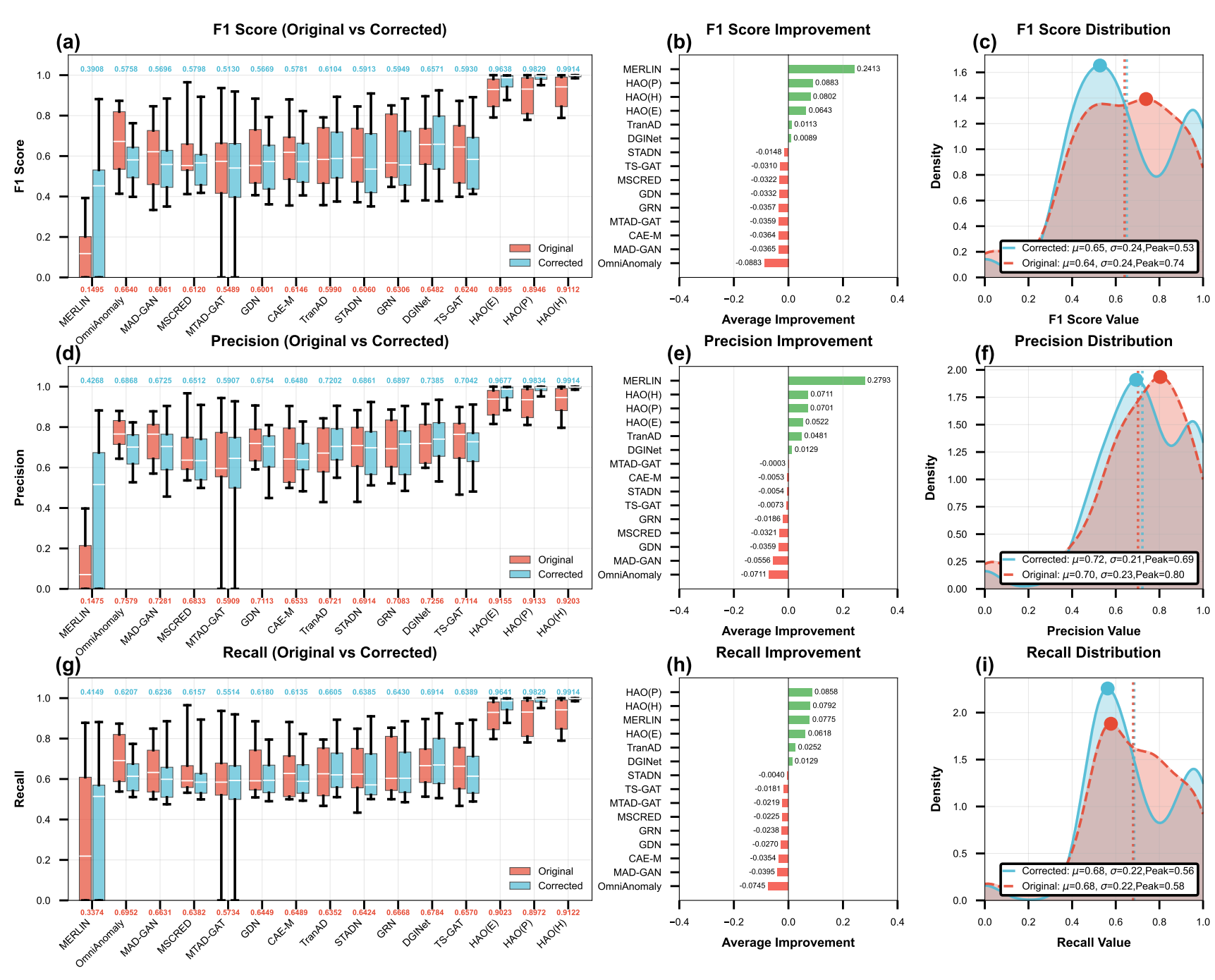


Figure 4: *Comprehensive Comparative Analysis of Multivariate Anomaly Detection Model Performance Based on F1-score, Precision and Recall Before and After Anomaly Label Correction. Figs. 2(a, d, g) show box plots of average performance across different datasets; Figs. 2(b, e, h) show the performance improvement effects; and Figs. 2(c, f, j) show the performance density distributions.*

*...The HAO series models achieve significant improvements in both precision and recall. The average precision is 0.9163 before correction and it increases to 0.9808 after correction, which represents an improvement of 0.0645. The average recall is 0.9039 before correction and it increases to 0.9794 after correction, which represents an improvement of 0.0755. These results demonstrate that the HAO model architecture can dynamically adapt to labeling noise and effectively avoid performance degradation caused by labeling bias in traditional models...*

**[Section 4.3.5 Spatio-Temporal Complexity and Reconstruction Error Analysis (Main Text: Page 28-29)]***The data reconstruction error of the HAO model series is significantly lower than that of the baseline models and the reconstruction error exhibits a gradually decreasing trend as the geometric representation capacity of the embedding space improves, as shown in Fig. 2(a). Among all comparison methods, MSCRED [1] achieves the best performance with a reconstruction error of 0.0044. In contrast, the reconstruction error of the HAO model is 0.0031 in the Euclidean space, 0.0030 in the Poincaré ball space and 0.0029 in the hyperbolic space, which represents a reduction of 34.09% compared with MSCRED...*

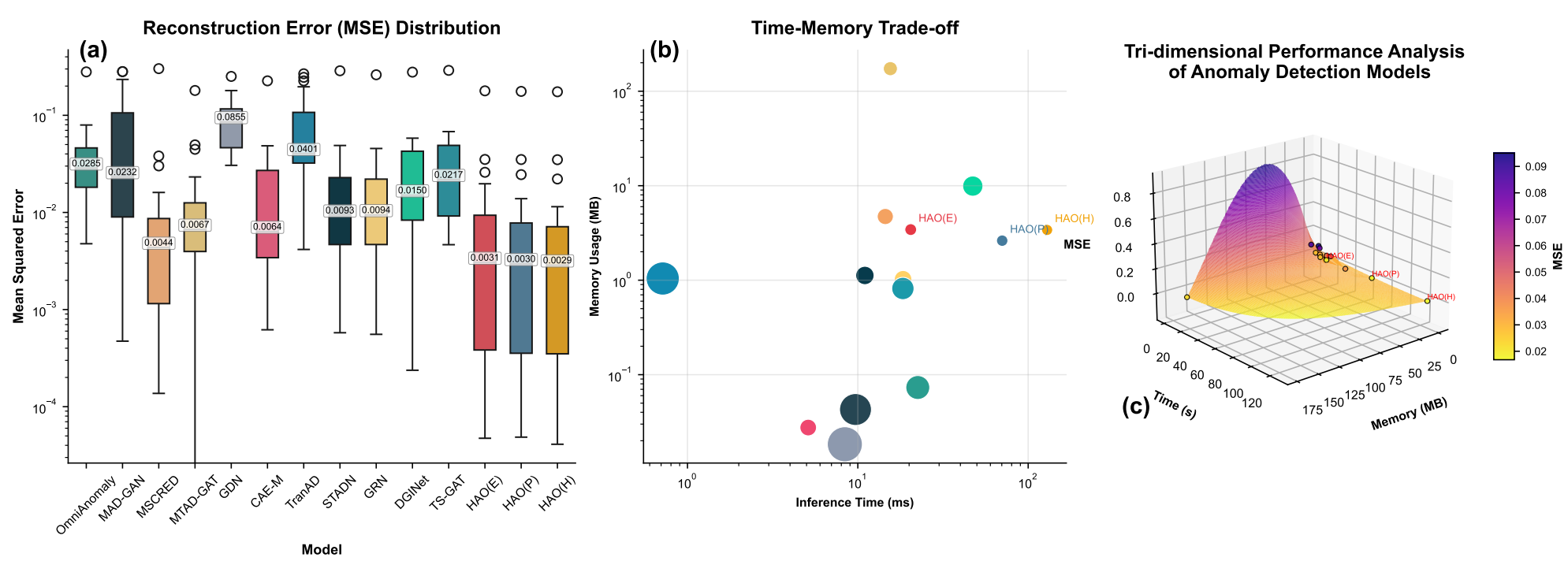


Figure 5: *Spatio-Temporal Complexity–Reconstruction Error and Three-Dimensional Performance Analysis of Multivariate Anomaly Detection Models*

**[Section 4.3.9 Training Stability Analysis(Main Text Page:34 - 35)]***...As training proceeds, the curvature of the spatio-temporal structure output module of the HAO model in hyperbolic space is dynamically adjusted and eventually converges to approximately 1.0...*

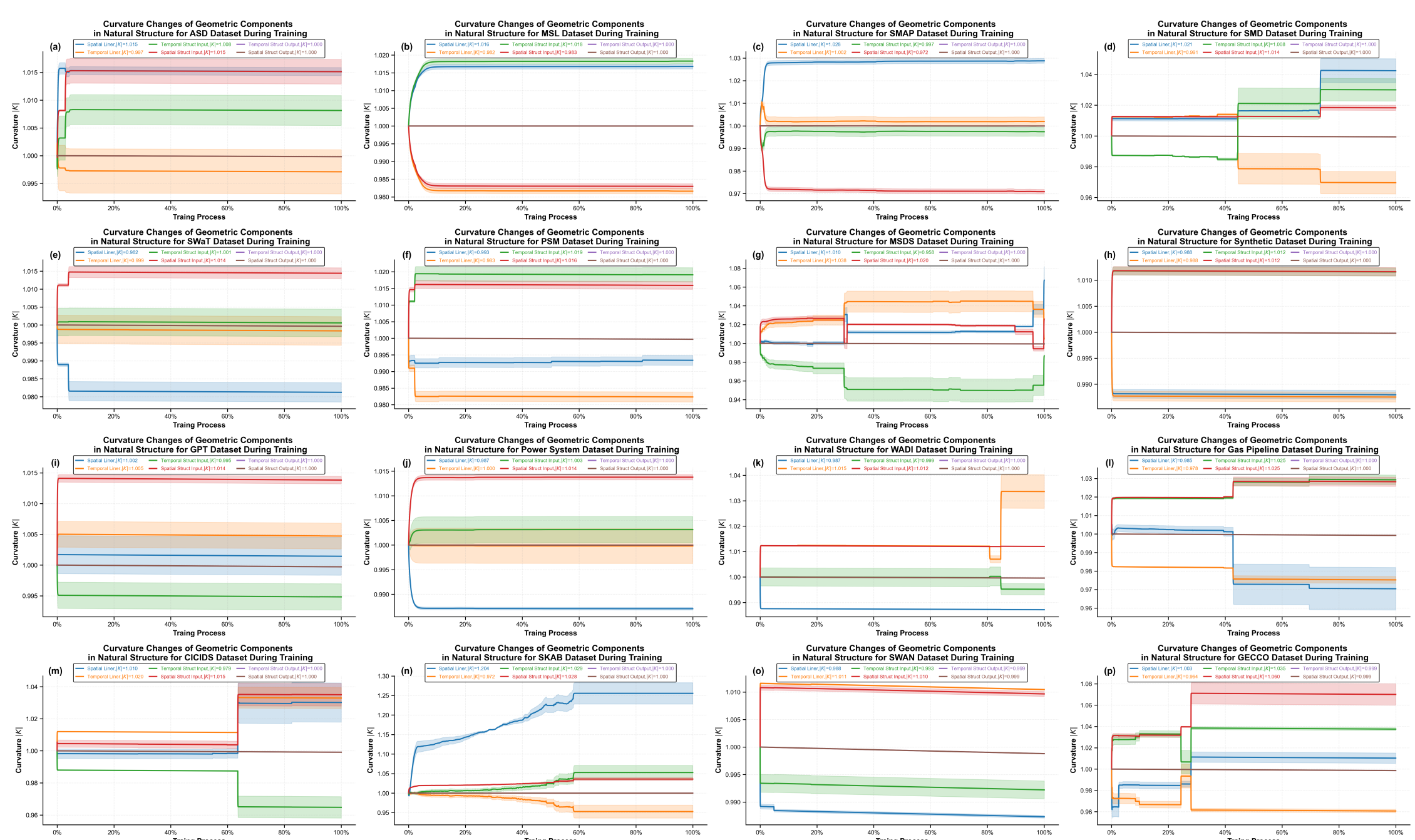

Figure 6: *Training Stability and Curvature Variation Analysis of the HAO Model*

**[Section 4.4.1 Comparative Performance Analysis of Anomaly Diagnosis (Main Text: Page 35-37)]**...*The performance of multivariate anomaly diagnosis HitRate@600% under different detection scenarios is shown in Fig. 7(a). Specifically, the average HitRate@600% of the HAO model in Euclidean space is 93.1%, that in the Poincaré ball space is 97.5% and that in hyperbolic space is 98.7%. In contrast, the average HitRate@600% of all baseline models is below 70%. Furthermore, in terms of anomaly diagnosis quality, as shown in Fig. 7(c), the average NDCG@600% of the HAO model in Euclidean space is 89.0%, that in the Poincaré ball space is 94.2% and that in hyperbolic space is 97.6%, while the average NDCG@600% of baseline models is below 40%. These results demonstrate that the key challenge in multivariate anomaly diagnosis lies in the precise attribution of multivariate spatio-temporal coupling relationships...*

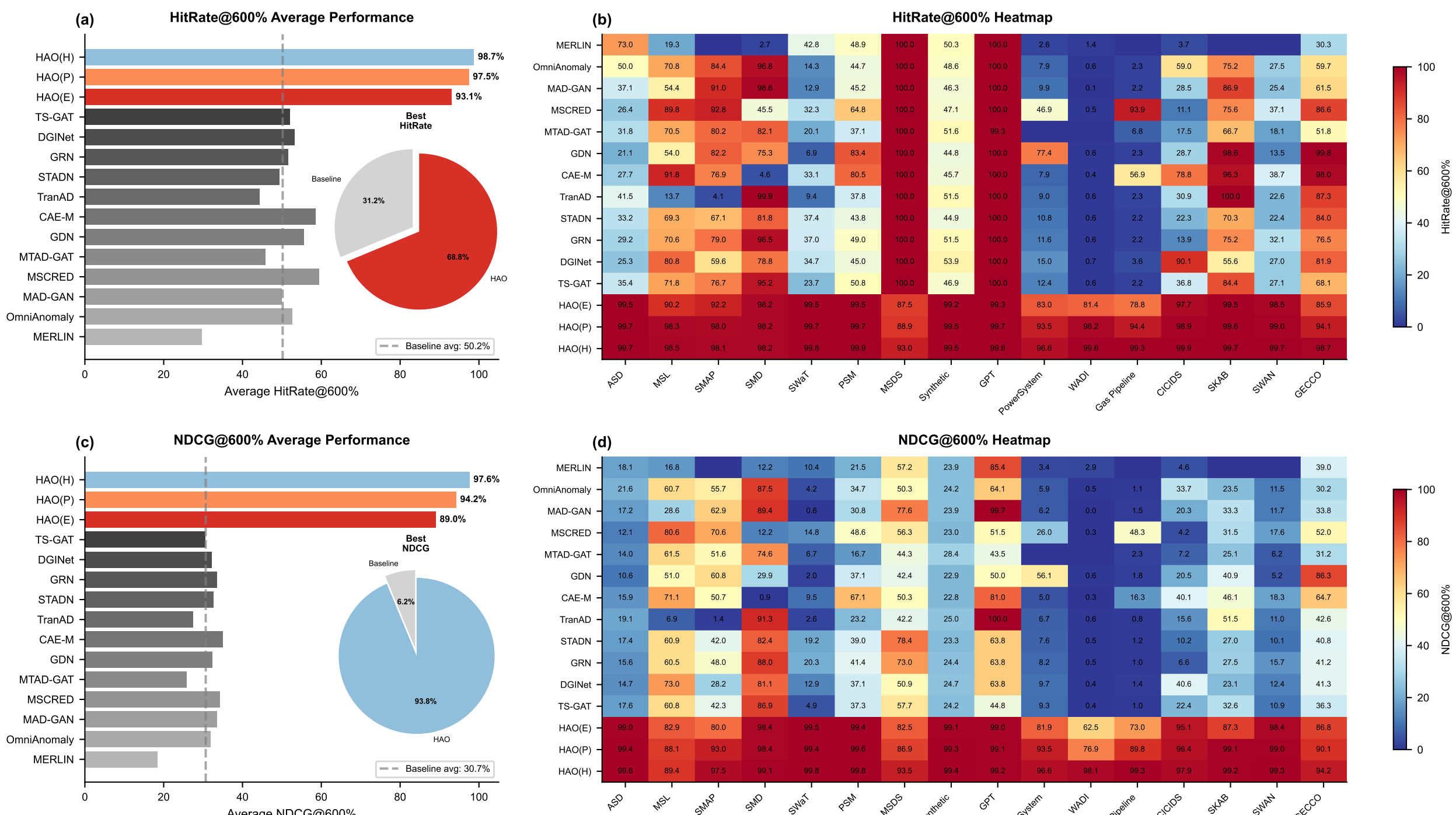


Figure 7: Comparative Analysis of Anomaly Diagnosis Performance for Multivariate Anomaly Detection Models

**[Section 4.5.1 Analysis of Multivariate Spatio-Temporal Relationship Parsing Capability (Main Text: Page 37-39)]** *...range of 0.2-0.8 with a uniform distribution and the $H_s$ remains stable between 11.30 and 11.31. For instance, the $H_s$ is 11.31 for both a sliding sampling window of 5 (Fig. 2(A(a))) and 110 (Fig. 2(A(f))), with consistent trends in correlation strength distribution. This demonstrates that the linear geometric nature of Euclidean space is inadequate to characterize the nonlinear dependencies among multivariate variables. The incremental temporal information introduced by the sliding sampling window fails to improve the ability of coupling structure mining, which only homogenizes the correlation strength without distinguishing the relationships between core and secondary coupling. In the Poincaré ball space, the $H_s$ of the multivariate coupling structure obtained by the HAO model decreases slightly as the sliding sampling window enlarges and the correlation strength exhibits local aggregation, which leads to better discrimination of coupling relationships compared with Euclidean space. The $H_s$ drops from 11.31 to 11.25 and the correlation strength distribution gradually shifts from uniform to locally high-density regions, where core coupling relationships begin to emerge...*

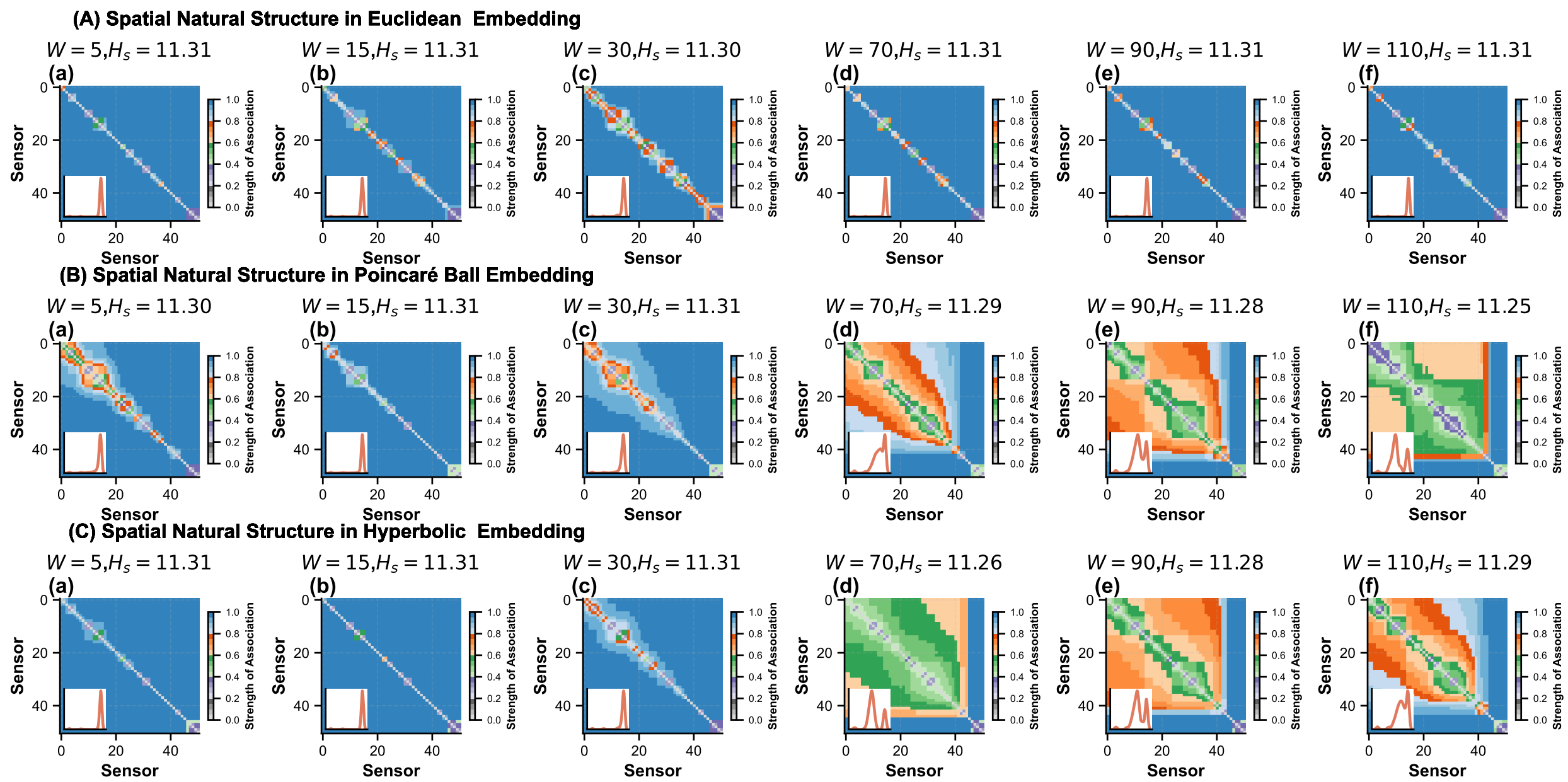


Figure 8: *Analysis of the Impact of Embedding Space and Sliding Window on Natural Multivariate Coupling Structures. The analysis is conducted on the SWaT dataset[2], which exhibits dynamic coupling relationships. Edge entropy, denoted as $H_{\boldsymbol{s}}(\mathbf{A}) = -\sum_{i=1}^{N} p_i \cdot \log(p_i)$, where $p_i = \mathbf{A}_i / \sum_{j=1}^{N} \mathbf{A}_j$ is used to reflect the richness of coupling relationships, while the association strength distribution reflects the tightness of dependencies between different sensors.*

## References


[1] Zhang, C. et al. A deep neural network for unsupervised anomaly detection and diagnosis in multivariate time series data. In *Proc. AAAI Conf. Artif. Intell.* **33**, 1409–1416 (2019).

[2] Mathur, A. P. et al. SWaT: a water treatment testbed for research and training on ICS security. In *Proc. Int. Workshop Cyber-phys. Syst. Smart Water Netw. (CySWater)* 31–36 (2016).


**4. Regarding reproducibility, the GitHub repository currently lacks several components required for end-to-end replication. For instance, in main.py, modules such as src.merlin, src.pot, and src.NoPOT appear to be missing or inaccessible.**

**Response 4:** We sincerely appreciate the reviewer's careful examination and valuable feedback concerning the code reproducibility of our work. We offer our sincere apologies for the omission of core code modules, including 'src.merlin', 'src.pot' and 'src.NoPOT', as **the reviewer** kindly pointed out. In direct response to this issue, we have thoroughly updated the entire code repository and performed comprehensive validation checks to guarantee the completeness and operational correctness of all code components.

All source code required to replicate the experimental results presented in this study, together with the supporting code for the corresponding pip packages, is publicly available under the open-source MIT license in our dedicated GitHub repository: `https://github.com/StructureMan/HAO`.

## 3 Reviewer #3 (Remarks on code availability):

**1. Regarding reproducibility, the GitHub repository currently lacks several components required for end-to-end replication. For instance, in main.py, modules such as src.merlin, src.pot, and src.NoPOT appear to be missing or inaccessible.**

**Response 1:** We sincerely appreciate the reviewer's valuable feedback concerning the availability of the source code, and a detailed response is provided in Response **Response 4**.

# Revision of manuscript in Nature Communications #NCOMMS-24-82438B-Z

## Explanation of Revisions

We are submitting a revised version of our paper, "Hyperbolic Adaptive Spatial Perception Multivariate Time Series Anomaly Detection" (ID: #NCOMMS-24-82438B-Z). We sincerely thank the reviewers for their valuable and constructive comments. We thoroughly revise the paper in accordance with these suggestions, provide detailed responses to all review comments and add new analytical content to further strengthen our research. We hereby declare that this paper has not been published elsewhere in any form.

In order to ensure that the revisions made in response to editorial comments and feedback from reviewers are clear, standardized and easy to review, the following is an explanation of how responses and revisions are marked:

- **Reviewer comment presentation:** We strictly quote the original comments of the reviewers and highlight them in black. For example: **The manuscript introduces the Hyperbolic Adaptive Spatial-Aware...**
- **Response Marking of responses to comments:** For each review comment, our response is uniformly marked in blue bold underlined format, labeled as: **Response 1 :**.
- **Revision Notes:** All modifications made in the text, whether they involve content adjustments or wording optimizations, will be marked using red bold underlined formatting, identified as: **Revision 1 :** Along with a brief explanation of the key points of the revision.
- **Location of Revisions:** To help reviewers quickly find the exact locations of the changes, we have marked the section titles and page numbers at the relevant modification points in red bold text, using the format: **[Section Title (Revised File: Page Number)]**, for example: **[Section 2.2.4 Hyperbolic Graph Representation Learning (Main Text: Page 7)]**.

## 1 Reviewer #2 (Remarks to the Author):

**Thanks for authors' revision. My concerns have been addressed.**

**Response :** We sincerely appreciate the insightful comments and valuable suggestions from the reviewers. These constructive feedback are extremely crucial for improving the academic quality and scientific rigor of this work. We once again express our deepest gratitude for your rigorous and careful review.

## 2 Reviewer #4 (Remarks to the Author):

**This paper proposes a novel method for multivariate time series anomaly detection. The proposed method uses an adaptive hyperbolic pre-training framework to capture the relationship between different variates, and employs a graph structure discovery mechanism to automatically learn a graph structure that connects different variates, without replying on predefined structure. A multi-level signal parsing attention mechanism is also introduced to tackle insufficient model robustness caused by complex signals. Experiments are conducted to show the effectiveness of the proposed method. The paper has several strong points: 1. The research problem is significant. 2. The empirical discussions are solid. 3. The proposed method is well designed. 4. The experiments are extensive. Despite its merit, this paper still requires a minor revision. Detailed comments are as follows:**

**1. The claims of "first time" are too strong and should be toned down or supported with clearer comparisons. At least, you can mention "to the best of our knowledge" for some unsure claims.**

**Response 1:** We greatly appreciate the valuable suggestions provided by the reviewers. We fully agree with the comments regarding the prudent use of absolute expressions including "first proposed" and "first discovered". As noted in the review comments, the tone of innovative descriptions ought to be appropriately moderated, and sufficient comparative experiments as well as literature reviews should be supplemented for solid support. For research conclusions without widespread academic consensus, restrictive phrases such as "to the best of our knowledge" are adopted to guarantee the rigor and objectivity of academic writing. Following the above suggestions, the entire manuscript has been thoroughly examined, and all non-standard expressions have been revised item by item. Detailed revision contents are presented as follows.

(1) **[Abstract(Main Text : Page I)]**To the best of our knowledge, the proposed adaptive hyperbolic pre-training framework, adaptive hyperbolic graph structure discovery, multivariate anomaly spatio-temporal coordinate localization, multi-level signal parsing attention mechanism and small-sample hybrid dynamic threshold determination algorithm are all introduced here for the first time and successfully applied in this domain.

(2) **[Abstract(Main Text : Page I)]**This not only validates the theoretical feasibility and application reliability of the proposed method, but also introduces, for the first time, research and exploration of explainable AI in the field of anomaly detection.

(3) **[Section 1 Introduction(Main Text : Page 5)]**· · · We propose an automated anomaly labelling and correction framework that, for the first time, exploits geometric discrepancies between natural and predicted topologies in conjunction with the distribution characteristics of anomaly scenarios to achieve label correction spanning from coarse to fine granularity. This not only establishes a more interpretable labelling paradigm but also provides a more objective and reliable benchmark for evaluating anomaly detection performance.· · ·

(4) **[Section 1 Introduction(Main Text : Page 5)]**To the best of our knowledge, this represents the first successful application of hyperbolic space theory in the field of unsupervised graph structure discovery.

(5) **[Section 1 Introduction(Main Text : Page 5)]**By offering a more comprehensive analytical perspective for understanding the underlying mechanisms of anomalous phenomena, this method constitutes the first exploration of explainable AI in the field of anomaly detection.

In accordance with the reviewers' comments, we have revised the relevant statements as follows:

For point (1), the original expression is redundant and fails to fully highlight the core contributions of this study. Accordingly, we added the qualifier "to the best of our knowledge", and the revised sentence is as follows: **To the best of our knowledge, based on the constructed adaptive original graph topology, this study establishes anomaly discrimination criteria and deviation correction mechanisms, and accomplishes end-to-end multivariate anomaly detection including recognition, diagnosis and interpretation within a unified reasoning granularity.**

For point (2), this study is indeed the first attempt to realize multivariate anomaly recognition, diagnosis and interpretation under unified reasoning granularity. Therefore, we retain the word "for the first time" in this manuscript.

For items (3-5), in response to the comments in Reviewer Comment 2 regarding ambiguous definition and insufficient distinction of core innovations, we reorganized the innovation points: The original innovation points (2) and (3) are merged, both of which belong to core breakthroughs in underlying model architecture and representation learning, namely hyperbolic space-based graph structure exploration and adaptive hyperbolic pre-training framework.

The original innovation points (1) and (4) are combined, which focus on the application and interpretation of anomaly analysis, including fine-grained anomaly label correction and anomaly cause interpretation, emphasizing interpretability and objectivity. The revised contents are shown below:

(1) **This study establishes an adaptive hyperbolic space representation learning framework, which overcomes the inherent drawbacks of conventional methods dependent on predefined prior structures and Euclidean space representations.** This study further propose a collaborative mechanism integrating Adaptive Unsupervised Hyperbolic Graph Structure Discovery (AH-GSD) and Adaptive Hyperbolic Reconstruction-Prediction Pre-training Framework (AH-PH). On the one hand, AH-GSD enables low-loss dynamic generation and sparsification of natural structures of multivariate data, removes rigid restrictions imposed by manually predefined priors, and autonomously identifies and collaboratively localizes spatio-temporal anomaly correlations. On the other hand, AH-PH dynamically formulates reconstruction rules adaptable to complex data distributions and optimizes hyperbolic linear layers as well as graph convolutional layers, thereby substantially boosting computational efficiency and embedding quality. To the best of our knowledge, this work achieves the first successful deployment of hyperbolic space theory in unsupervised adaptive graph structure discovery, and builds a high-quality fundamental embedding space for multivariate data analysis.

(2) **This study proposes a self-feedback anomaly detection research framework covering multivariate anomaly benchmark construction, anomaly recognition, anomaly diagnosis and anomaly interpretation.** In terms of data annotation, we design an automatic anomaly annotation and correction framework. For the first time, we utilize the geometric differences between natural topology and predicted topology as well as inherent anomaly distribution characteristics to implement coarse-to-fine label refinement, and establish interpretable annotation criteria and quantitative evaluation benchmarks. In terms of anomaly interpretation, we put forward a cross-domain anomaly cause analysis method. By deeply mining inherent spatio-temporal natural structures, this method realizes accurate anomaly traceability, and effectively compensates for the limitation that existing studies only focus on local features while ignoring global complex system coupling relationships. This work is a pioneering exploration of explainable artificial intelligence in multivariate anomaly detection, providing an effective analytical approach to reveal the deep internal mechanism of multivariate anomalies.

**Revision 1:**

**[Abstract(Main Text : Page I)]**

To the best of our knowledge, based on the constructed adaptive original graph topology, this study establishes anomaly discrimination criteria and deviation correction mechanisms, and accomplishes end-to-end multivariate anomaly detection including recognition, diagnosis and interpretation within a unified reasoning granularity.

**[Section 1 Introduction(Main Text : Page 5)]**

(1)**This study establishes an adaptive hyperbolic space representation learning framework, which overcomes the inherent drawbacks of conventional methods dependent on predefined prior structures and Euclidean space representations.** This study further propose a collaborative mechanism integrating Adaptive Unsupervised Hyperbolic Graph Structure Discovery (AH-GSD) and Adaptive Hyperbolic Reconstruction-Prediction Pre-training Framework (AH-PH). On the one hand, AH-GSD enables low-loss dynamic generation and sparsification of natural structures of multivariate data, removes rigid restrictions imposed by manually predefined priors, and autonomously identifies and collaboratively localizes spatio-temporal anomaly correlations. On the other hand, AH-PH dynamically formulates reconstruction rules adaptable to complex data distributions and optimizes hyperbolic linear layers as well as graph convolutional layers, thereby substantially boosting computational efficiency and embedding quality. To the best of our knowledge, this work achieves the first successful deployment of hyperbolic space theory in unsupervised adaptive graph structure discovery, and builds a high-quality fundamental embedding space for multivariate data analysis.

(2) **This study proposes a self-feedback anomaly detection research framework covering multivariate anomaly benchmark construction, anomaly recognition, anomaly diagnosis and anomaly interpretation.** In terms of data annotation, we design an automatic anomaly annotation and correction framework. For the first time, we utilize the geometric differences between natural topology and predicted topology as well as inherent anomaly distribution characteristics to implement coarse-to-fine label refinement, and establish interpretable annotation criteria and quantitative evaluation benchmarks. In terms of anomaly interpretation, we put forward a cross-domain anomaly cause analysis method. By deeply mining inherent spatio-temporal natural structures, this method realizes accurate

anomaly traceability, and effectively compensates for the limitation that existing studies only focus on local features while ignoring global complex system coupling relationships. This work is a pioneering exploration of explainable artificial intelligence in multivariate anomaly detection, providing an effective analytical approach to reveal the deep internal mechanism of multivariate anomalies.

**2. The proposed framework integrates multiple components, but the core novelty is not clearly distinguished. I suggest the authors to highlight the most significant 1-2 novel points in introduction/abstract, and the rest points can be regarded as supplemental.**

**Response 2:** We sincerely appreciate the reviewers for pointing out the issues including ambiguous definition of core innovations and insufficient distinction between different research contributions. In accordance with the review suggestions, we have optimized the expression of the abstract and introduction in **Response 1**, further clarified the orientation of core innovations and rationalized the logical hierarchy of all research contributions. We once again highly appreciate your constructive and insightful comments.

**3. Why hyperbolic space is better for such learning problem? More detailed discussion, especially from a theoretical perspective, is expected.**

**Response 3:** We highly appreciate the crucial guidance provided by the reviewers. In response to the comment that systematic theoretical demonstration is urgently needed to explain why hyperbolic space suits natural graph structure mining well, we elaborate the advantages of hyperbolic space over traditional Euclidean space from four perspectives: geometric properties, metric distortion, representation capacity and optimization mechanism. The detailed demonstration is presented as follows.

Most existing time series modeling methods adopt Euclidean space embedding, yet their geometric characteristic of uniform expansion pose inherent limitations. The volume of Euclidean space grows polynomially with radius, which cannot match the hierarchical and exponentially expanding inherent structure of real industrial time series systems [1]. This dimension growth mechanism leads to obvious geometric embedding distortion. Especially in dynamic temporal graph scenarios, it easily results in insufficient capture of long-range dependencies, deviation of dynamic evolution features and degradation of sparse structure representation. To address such structural defects, recent studies have introduced hyperbolic geometric space with negative curvature characteristics [2]. Its unique exponential volume expansion mechanism achieves natural geometric isomorphism with hierarchical topological structures of complex systems, providing a more suitable modeling foundation for this task. As shown in Fig. 1, hyperbolic space follows distinctive geometric expansion rules, and its volume conforms to $V(r) \propto e^{\zeta r}$, where $\zeta$ denotes the curvature parameter. The expansion intensity of the space can be adaptively adjusted by tuning the curvature value.

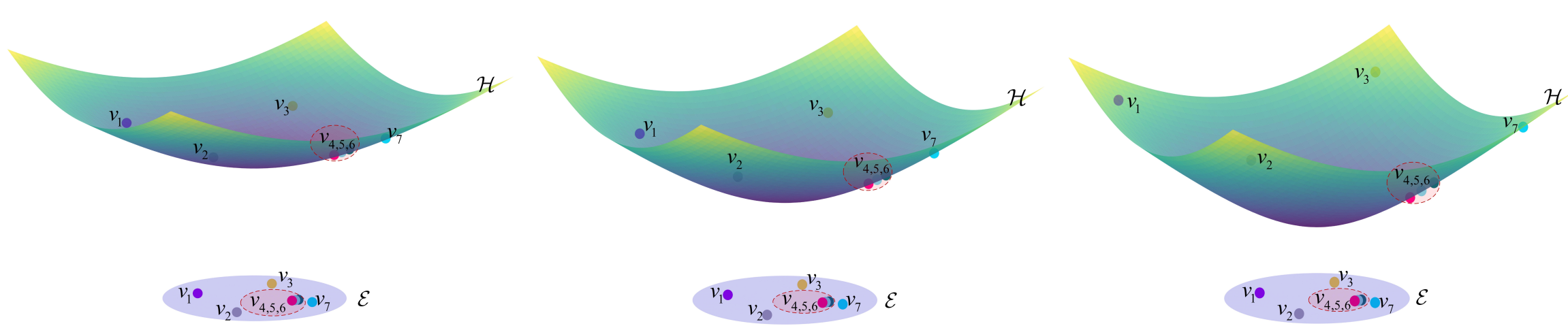

(a) $K = -1$ (b) $K = -2$ (c) $K = -4$

Figure 1: This figure shows the embedding representations of data points in hyperbolic spaces with different curvatures. In Euclidean space, the relationships among data points (e.g., $v_4, v_5, v_6$) exhibit weak discriminability while displaying strong clustering, or community structure [3]. However, after being mapped to hyperbolic space, as the absolute value of the curvature increases, corresponding sequentially to subfigure (a) $K = -1$, (b) $K = -2$ and (c) $K = -4$ , the geometric distances between these data points expand significantly. This indicates a systematic improvement in the discriminability of their natural relationships. Such enhanced differentiation of key relationships, especially anomalous relationships, not only facilitates more effective recognition of subtle anomaly types, but also provides finer and more robust evidence for uncovering the causes of previously unknown anomaly types.

It can be clearly observed from Fig. 1 that data points are densely distributed in Euclidean space with weakly distinguishable relational features, whereby numerous sparse, weak and abnormal structural connections are concealed by dense clusters[4]. In contrast, as the absolute value of curvature in hyperbolic space increases, the geometric distances between samples expand evidently, and the distinguishability of hierarchical structures as well as subtle abnormal connections is greatly improved. From the perspective of metric geometric distortion theory, hyperbolic embedding is capable of fitting complex hierarchical structures with extremely low distortion. Existing theoretical studies have proven that embedding finite $\delta$-hyperbolic structures into hyperbolic space $\mathcal{H}^d$ yields multiplicative distortion bounded by

$O(\delta \log n)$[5, 6]. When embedding identical hierarchical structures into Euclidean space, however, the lower bound of distortion increases polynomially with the number of nodes as $\Omega(n^{1/d})$. This demonstrates that Euclidean embedding inevitably distorts the inherent hierarchical distances of networks and artificially compresses the spatial distances of edge nodes and sparse structures. Consequently, numerous sparse abnormal nodes are crowded into dense normal clusters, making subtle anomalies unnoticeable and undetectable. In comparison, hyperbolic space effectively preserves hierarchical distance differences and mitigates structural embedding distortion, which theoretically accounts for the phenomenon that structural distinguishability is significantly enhanced with the increase in absolute curvature values shown in Fig. 1(a-c).

Complex dynamic industrial networks generally exhibit tree-like hierarchical characteristics[5][6][7]. Given a branching factor $b$, the number of nodes at depth $r$ grows exponentially as $b^r$. Constrained by its polynomial volume growth nature, Euclidean space fails to match such exponential structural expansion. It can only avoid node overlapping by continuously increasing embedding dimensions, which inevitably leads to severe dimensionality curse [8]. By virtue of its inherent exponential expansion property, hyperbolic space can accommodate tree-like hierarchical structures with arbitrary depths in low-dimensional spaces when the curvature parameter satisfies $b \approx e^{\zeta}$ [5]. This property enables the full retention of dynamic evolution details and sparse abnormal structures within multivariate time series data. More importantly, the curvature-aware gradient mechanism of hyperbolic space can accurately activate the representation learning of sparse abnormal nodes. Different from the isotropic and invariant gradient update rule in Euclidean space, hyperbolic Riemannian manifolds dynamically reshape the optimization landscape via metric tensors [6]. Within the tangent space $\mathcal{T}_{\boldsymbol{x}}\mathcal{H}^{d,\zeta}$, Riemannian gradients adaptively scale the parameter updating magnitude according to spatial curvature [9]. For feature points located at spatial boundaries, i.e., hierarchical leaf nodes and sparse abnormal samples, the metric tensor automatically amplifies the gradient norm, which effectively alleviates the prevalent gradient vanishing problem in deep temporal graph models [10]. This mechanism enables the model to finely tune and reinforce the learning of weak anomalous patterns and sparse abnormal correlations, and remarkably improves the detection performance for subtle and concealed anomalies, which is fully consistent with the visualized distribution rules in Fig. 1(a-c).

## References


[1] Krioukov, D., Papadopoulos, F., Kitsak, M., Vahdat, A. and Boguná, M. Hyperbolic geometry of complex networks. *Phys. Rev. E* **82**, 036106 (2010).

[2] Xu, Y. Y. et al. Scalable and effective temporal graph representation learning with hyperbolic geometry. *IEEE T. Neural Netw. Learn. Syst.* (2024).

[3] Ranshous, S. et al. Anomaly detection in dynamic networks: a survey. *Wires. Comput. Stat.* **7**, 223–247 (2015).

[4] Fu, X.C. et al. ACE-HGNN: Adaptive Curvature Exploration Hyperbolic Graph Neural Network. In *Int. Conf. Data Min.*, 111–120 (2021).

[5] Nickel, M. and Kiela, D. Learning continuous hierarchies in the Lorentz model of hyperbolic geometry. In *Proc. Int. Conf. Mach. Learn.*, 3776–3785 (2018).

[6] Chami, I. et al. Hyperbolic graph convolutional neural networks. *Adv. Neural Inf. Process. Syst.* **32** (2019).

[7] Ravasz, E. et al. Hierarchical organization in complex networks. *Phys. Rev. E* **67**, 026112-026119(2003).

[8] Dai, J. D. et al. A hyperbolic-to-hyperbolic graph convolutional network. In *Proc. IEEE/CVF Conf. Comput. Vis. Pattern Recognit.* (2021).

[9] Peng, W., Varanka, T., Mostafa, A., Shi, H. & Zhao, G. Hyperbolic deep neural networks: a survey. *IEEE. T. Pattern. Anal.* **44**, 10023–10044 (2022).

[10] Gulcehre, C. et al. Hyperbolic attention networks. In *Proc. Int. Conf. Learn. Represent.* (2019).


**Revision 3:**

**[Section 2.2.4 Hyperbolic Graph Representation Learning (Main Text: Page 9-10)]**

$\cdots$ Most existing time series modeling methods adopt Euclidean space embedding, yet their geometric characteristic of uniform expansion pose inherent limitations. The volume of Euclidean space grows polynomially with radius, which cannot match the hierarchical and exponentially expanding inherent structure of real industrial time series systems [1]. This dimension growth mechanism leads to obvious geometric embedding distortion. Especially in dynamic temporal graph scenarios, it easily results in insufficient capture of long-range dependencies, deviation of dynamic evolution features and degradation of sparse structure representation. To address such structural defects, recent studies have introduced hyperbolic geometric space with negative curvature characteristics [2]. Its unique exponential volume

expansion mechanism achieves natural geometric isomorphism with hierarchical topological structures of complex systems, providing a more suitable modeling foundation for this task. As shown in Fig. 1, hyperbolic space follows distinctive geometric expansion rules, and its volume conforms to $V(r) \propto e^{\zeta r}$, where $\zeta$ denotes the curvature parameter. The expansion intensity of the space can be adaptively adjusted by tuning the curvature value.

It can be clearly observed from Fig. 1 that data points are densely distributed in Euclidean space with weakly distinguishable relational features, whereby numerous sparse, weak and abnormal structural connections are concealed by dense clusters[4]. In contrast, as the absolute value of curvature in hyperbolic space increases, the geometric distances between samples expand evidently, and the distinguishability of hierarchical structures as well as subtle abnormal connections is greatly improved. From the perspective of metric geometric distortion theory, hyperbolic embedding is capable of fitting complex hierarchical structures with extremely low distortion. Existing theoretical studies have proven that embedding finite $\delta$-hyperbolic structures into hyperbolic space $\mathcal{H}^d$ yields multiplicative distortion bounded by $O(\delta \log n)$[5, 6]. When embedding identical hierarchical structures into Euclidean space, however, the lower bound of distortion increases polynomially with the number of nodes as $\Omega(n^{1/d})$. This demonstrates that Euclidean embedding inevitably distorts the inherent hierarchical distances of networks and artificially compresses the spatial distances of edge nodes and sparse structures. Consequently, numerous sparse abnormal nodes are crowded into dense normal clusters, making subtle anomalies unnoticeable and undetectable. In comparison, hyperbolic space effectively preserves hierarchical distance differences and mitigates structural embedding distortion, which theoretically accounts for the phenomenon that structural distinguishability is significantly enhanced with the increase in absolute curvature values shown in Fig. 1(a-c).

Complex dynamic industrial networks generally exhibit tree-like hierarchical characteristics[5][6][7]. Given a branching factor $b$, the number of nodes at depth $r$ grows exponentially as $b^r$. Constrained by its polynomial volume growth nature, Euclidean space fails to match such exponential structural expansion. It can only avoid node overlapping by continuously increasing embedding dimensions, which inevitably leads to severe dimensionality curse [8]. By virtue of its inherent exponential expansion property, hyperbolic space can accommodate tree-like hierarchical structures with arbitrary depths in low-dimensional spaces when the curvature parameter satisfies $b \approx e^{\zeta}$ [5]. This property enables the full retention of dynamic evolution details and sparse abnormal structures within multivariate time series data. More importantly, the curvature-aware gradient mechanism of hyperbolic space can accurately activate the representation learning of sparse abnormal nodes. Different from the isotropic and invariant gradient update rule in Euclidean space, hyperbolic Riemannian manifolds dynamically reshape the optimization landscape via metric tensors [6]. Within the tangent space $\mathcal{T}_{\boldsymbol{x}}\mathcal{H}^{d,\zeta}$, Riemannian gradients adaptively scale the parameter updating magnitude according to spatial curvature [9]. For feature points located at spatial boundaries, i.e., hierarchical leaf nodes and sparse abnormal samples, the metric tensor automatically amplifies the gradient norm, which effectively alleviates the prevalent gradient vanishing problem in deep temporal graph models [10]. This mechanism enables the model to finely tune and reinforce the learning of weak anomalous patterns and sparse abnormal correlations, and remarkably improves the detection performance for subtle and concealed anomalies, which is fully consistent with the visualized distribution rules in Fig. 1(a-c).

Driven by these theoretical advantages, hyperbolic representation learning has developed rapidly and been widely adopted. Initially used for modeling complex networks, hyperbolic space has five isometric models: the hyperboloid model (also known as the Lorentz model), the Klein model, the Gans model, the Poincaré ball model, and the hemisphere model. Among these, the Poincaré ball model has been widely adopted due to its numerical stability [5]. Building upon these foundations, hyperbolic graph representation learning has achieved remarkable success. For instance, HGNN (Hyperbolic Graph Neural Network) [5] demonstrates superior effectiveness over traditional Euclidean models in graph classification tasks. HGAT [10] further proposes a graph convolution method incorporating hyperbolic attention mechanisms, which enables information propagation via gyrovector operations and applies it to practical scenarios such as node classification and clustering. HGCN [6] focuses on local structures, designing a local aggregation scheme that performs hyperbolic graph embedding within local tangent spaces and introducing learnable curvature parameters to adapt to diverse data characteristics.

. . .

## References


[1] Krioukov, D., Papadopoulos, F., Kitsak, M., Vahdat, A. and Boguná, M. Hyperbolic geometry of complex networks. *Phys. Rev. E* **82**, 036106 (2010).

[2] Xu, Y. Y. et al. Scalable and effective temporal graph representation learning with hyperbolic geometry. *IEEE T. Neural Netw. Learn. Syst.* (2024).

[3] Ranshous, S. et al. Anomaly detection in dynamic networks: a survey. *Wires. Comput. Stat.* **7**, 223–247 (2015).

[4] Fu, X.C. et al. ACE-HGNN: Adaptive Curvature Exploration Hyperbolic Graph Neural Network. In *Int. Conf. Data Min.*, 111–120 (2021).

[5] Nickel, M. and Kiela, D. Learning continuous hierarchies in the Lorentz model of hyperbolic geometry. In *Proc. Int. Conf. Mach. Learn.*, 3776–3785 (2018).

[6] Chami, I. et al. Hyperbolic graph convolutional neural networks. *Adv. Neural Inf. Process. Syst.* **32** (2019).

[7] Ravasz, E. et al. Hierarchical organization in complex networks. *Phys. Rev. E* **67**, 026112-026119(2003).

[8] Dai, J. D. et al. A hyperbolic-to-hyperbolic graph convolutional network. In *Proc. IEEE/CVF Conf. Comput. Vis. Pattern Recognit.* (2021).

[9] Peng, W., Varanka, T., Mostafa, A., Shi, H. & Zhao, G. Hyperbolic deep neural networks: a survey. *IEEE. T. Pattern. Anal.* **44**, 10023–10044 (2022).

[10] Gulcehre, C. et al. Hyperbolic attention networks. In *Proc. Int. Conf. Learn. Represent.* (2019).

**4. Hyperbolic operations are computationally expensive, as well as the other complex components in the proposed method. In this case, a complexity analysis is expected to discuss the running efficiency of the proposed method. If possible, empirical results (e.g. run time comparison) are also preferred.**

**Response 4:** We greatly appreciate the reviewers' suggestions regarding the spatio-temporal complexity analysis and experimental comparison of the model. In accordance with the reviewers' comments, this paper has supplemented detailed theoretical derivation(Tab. 1) and horizontal comparative analysis of spatio-temporal complexity(Fig. 2) in the revised manuscript. Quantitative comparison experiments on actual running time and memory occupancy of DCdetector[1], ATrans[2], and TimesNet[3] have been added under the unified experimental hardware and software environments. The details are presented as follows:

(1) **The theoretical analysis of spatio-temporal complexity indicators for anomaly detection models is shown in Tab. 1.** The three variant models HAO(E), HAO(P), and HAO(H) proposed in this study adopt a unified complexity architecture with a time complexity of $O(N^2d + L^2d + N^2c)$ and a space complexity of $O(N^2 + L^2 + Nd + Ld)$. Specifically, $N^2d$ and $L^2d$ represent the basic computational costs of multivariate spatial coupled graph convolution and temporal dimension graph convolution, respectively. The additional $N^2c$ overhead is derived from the adaptive curvature distance calculation and dynamic adjacency structure updating module in Euclidean, Poincaré, and hyperbolic geometric spaces. The spatial overhead of the model is mainly dominated by the spatial-temporal adjacency matrix storage terms $N^2$ and $L^2$, resulting in a controllable overall complexity level and stable computational cost.

Table 1: Time and Space Complexity Comparison of Models.

| Model | Time Complexity | Space Complexity |
|---|---|---|
| OmniAnomaly | $O(LNd + Ld^2)$ | $O(Nd + d^2)$ |
| MAD-GAN | $O(LNd + Ld^2)$ | $O(Nd + d^2)$ |
| MSCRED | $O(LN^2k^2d)$ | $O(N^2d)$ |
| MTAD-GAT | $O(LN^2d + Ld^2)$ | $O(N^2 + Nd + d^2)$ |
| GDN | $O(LN^2d)$ | $O(N^2 + Nd)$ |
| CAE-M | $O(LN^2k^2d)$ | $O(N^2d)$ |
| TranAD | $O(L^2Nd)$ | $O(L^2 + LNd)$ |
| ATrans | $O(L^2Nd)$ | $O(L^2 + LNd)$ |
| TimesNet | $O(LNdk^2)$ | $O(LNd)$ |
| STADN | $O(LN^2d + Ld^2)$ | $O(N^2 + Nd + d^2)$ |
| GRN | $O(LN^2d + Ld^2)$ | $O(N^2 + Nd + d^2)$ |
| DCdetector | $O(L^2Nd)$ | $O(L^2 + LNd)$ |
| DGINet | $O(LN^2d + Ld^2)$ | $O(N^2 + Nd + d^2)$ |
| TS-GAT | $O(Ld^2 + LN^2d)$ | $O(N^2 + Nd + d^2)$ |
| HAO(E/P/H)(Ours) | $O(N^2d + L^2d + N^2c)$ | $O(N^2 + L^2 + Nd + Ld)$ |

**Symbol Definitions:** $L$ denotes the sliding window length; $N$ denotes the number of variables; $d$ denotes the hidden dimension; $c$ denotes the compression dimension in HAO; $k$ denotes the convolution kernel size.

In contrast, Transformer-based models including TranAD, ATrans, and DCdetector present a time complexity of $O(L^2Nd)$ that increases quadratically with sequence length, leading to a sharp surge in computational

cost and extremely high computing consumption in long-sequence industrial scenarios. The spatial-temporal feature decoupling architecture designed in this study confines the quadratic temporal computational overhead to an independent temporal subnetwork, effectively addressing the computational explosion problem caused by long-sequence inputs and significantly improving the model's adaptability to long time-series tasks. Although mainstream graph temporal models such as GDN and MTAD-GAT achieve the same $O(N^2d)$ space complexity level as the proposed model, they lack curvature-aware geometric modeling capabilities and fail to explore the deep latent structures of data. The minor $O(N^2c)$ computational overhead introduced in this study supports the core functions of multi-geometric-space adaptive modeling and hierarchical structure capture, achieving an optimal trade-off between model detection accuracy and computational efficiency.

(2) **Experimental results regarding the reconstruction performance, inference time, and memory footprint of the multivariate anomaly detection models are shown in Fig. 2.**

The HAO model series achieves a favorable trade-off between inference time and memory consumption. As shown in Fig. 2(b), the inference time and memory usage of the HAO model series remain at a moderate level. TimesNet has inference time and memory usage close to HAO(H). Its frequency-domain transformation and 2D convolution bring higher time overhead but reasonable memory consumption. Despite similar spatio-temporal complexity, HAO(H) achieves better performance. This advantage originates from architecture-level optimizations, including an independent learning structure of spatial and temporal features effectively simplifies high-dimensional feature disentanglement and computational procedures. Meanwhile, the dynamic graph structure discovery algorithm reduces redundant topology modeling, significantly decreasing computational and storage costs while maintaining detection performance. For further comprehensive performance analysis, the performance of the HAO model series in the three-dimensional space composed of inference time, memory usage and reconstruction error is displayed in Fig. 2(c). The HAO-series models consistently lie within the optimal performance set and they are the only multivariate anomaly detection models that simultaneously achieve lower spatial complexity, lower reconstruction error and higher detection performance. In contrast, some baseline models sacrifice performance in other dimensions by over-optimizing a single metric. For instance, MSCRED sacrifices spatio-temporal efficiency to improve detection effectiveness, while GDN suffers from degraded reconstruction and detection performance due to excessive computational simplification.

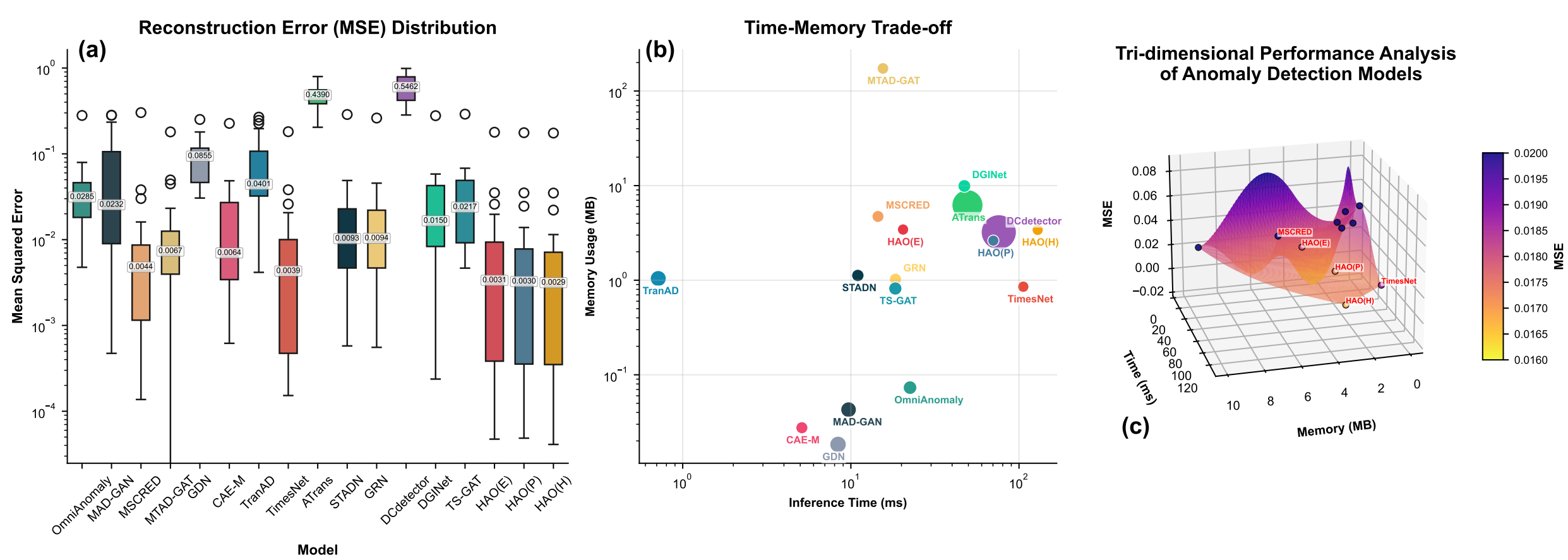


Figure 2: Spatio-Temporal Complexity–Reconstruction Error and Three-Dimensional Performance Analysis of Multivariate Anomaly Detection Models

Notably, although the inference time of the HAO model series increases slightly with performance improvement, its scalability remains controllable. Specifically, under the Euclidean space representation, the HAO model achieves the fastest inference speed with low memory usage and acceptable reconstruction error owing to its simple geometric structure. After introducing curved geometric constraints in the Poincaré ball space, the inference time rises moderately, but the reconstruction accuracy and detection performance are simultaneously improved. In the hyperbolic space, the model adopts a dynamic curvature adjustment mechanism and high-order geometric prior learning to achieve optimal reconstruction and detection results, which increases computational steps and results in the longest inference time among the three variants. This evolutionary trend indicates that the performance improvement of the HAO model series does not rely on simple computational overhead stacking, but it is achieved through the organic integration of natural geometric structures and multi-task collaborative optimization, which leads to cost-controllable performance expansion. This characteristic

enables the HAO model series to present strong potential for real-world engineering deployment in multivariate anomaly detection scenarios with diverse real-time requirements.

## References


[1] Yang, Y. et al. DCdetector: Dual Attention Contrastive Representation Learning for Time Series Anomaly Detection. In *Proc. ACM SIGKDD Conf. Knowl. Discov. Data Min.* 1–17 (2023).

[2] Xu, J. et al. Anomaly Transformer: Time Series Anomaly Detection with Association Discrepancy. In *Proc. Int. Conf. Learn. Represent.* (2022).

[3] Wu, H. et al. TimesNet: Temporal 2D-Variation Modeling for General Time Series Analysis. In *Proc. Int. Conf. Learn. Represent.* (2023).


**Revision 4:**

**[Section 4.7 Complexity Analysis (Supplementary Information: Page 8)]**

$\cdots$ The theoretical analysis of spatio-temporal complexity indicators for anomaly detection models is shown in Tab. 1. The three variant models HAO(E), HAO(P), and HAO(H) proposed in this study adopt a unified complexity architecture with a time complexity of $O(N^2d + L^2d + N^2c)$ and a space complexity of $O(N^2 + L^2 + Nd + Ld)$. Specifically, $N^2d$ and $L^2d$ represent the basic computational costs of multivariate spatial coupled graph convolution and temporal dimension graph convolution, respectively. The additional $N^2c$ overhead is derived from the adaptive curvature distance calculation and dynamic adjacency structure updating module in Euclidean, Poincaré, and hyperbolic geometric spaces. The spatial overhead of the model is mainly dominated by the spatial-temporal adjacency matrix storage terms $N^2$ and $L^2$, resulting in a controllable overall complexity level and stable computational cost.

$\cdots$

In contrast, Transformer-based models including TranAD, ATrans, and DCdetector present a time complexity of $O(L^2Nd)$ that increases quadratically with sequence length, leading to a sharp surge in computational cost and extremely high computing consumption in long-sequence industrial scenarios. The spatial-temporal feature decoupling architecture designed in this study confines the quadratic temporal computational overhead to an independent temporal subnetwork, effectively addressing the computational explosion problem caused by long-sequence inputs and significantly improving the model's adaptability to long time-series tasks. Although mainstream graph temporal models such as GDN and MTAD-GAT achieve the same $O(N^2d)$ space complexity level as the proposed model, they lack curvature-aware geometric modeling capabilities and fail to explore the deep latent structures of data. The minor $O(N^2c)$ computational overhead introduced in this study supports the core functions of multi-geometric-space adaptive modeling and hierarchical structure capture, achieving an optimal trade-off between model detection accuracy and computational efficiency. $\cdots$

**[Section 4.3.5 Spatio-Temporal Complexity and Reconstruction Error Analysis (Main Text: Page 30-31)]**

$\cdots$ The HAO model series achieves a favorable trade-off between inference time and memory consumption. As shown in Fig. 2(b), the inference time and memory usage of the HAO model series remain at a moderate level. TimesNet has inference time and memory usage close to HAO(H). Its frequency-domain transformation and 2D convolution bring higher time overhead but reasonable memory consumption. Despite similar spatio-temporal complexity, HAO(H) achieves better performance. This advantage originates from architecture-level optimizations, including an independent learning structure of spatial and temporal features effectively simplifies high-dimensional feature disentanglement and computational procedures. Meanwhile, the dynamic graph structure discovery algorithm reduces redundant topology modeling, significantly decreasing computational and storage costs while maintaining detection performance. For further comprehensive performance analysis, the performance of the HAO model series in the three-dimensional space composed of inference time, memory usage and reconstruction error is displayed in Fig. 2(c). The HAO-series models consistently lie within the optimal performance set and are the only multivariate anomaly detection models that simultaneously achieve lower spatial complexity, lower reconstruction error and higher detection performance. In contrast, some baseline models sacrifice performance in other dimensions by over-optimizing a single metric. For instance, MSCRED sacrifices spatio-temporal efficiency to improve detection effectiveness, while GDN suffers from degraded reconstruction and detection performance due to excessive computational simplification.$\cdots$ **5. The claim of causal interpretation is not sufficiently justified and appears to rely on heuristic explanations. The correctness and reliability of the "explanations" should be further discussed.**

**Response 5:** We sincerely appreciate the insightful and valuable comments raised by the reviewers. We fully agree with the issues pointed out that the statements regarding abnormal causal interpretation require more rigorous demonstrations,

and their validity and reliability should not be merely limited to heuristic explanations. In the original manuscript, we indeed had deficiencies in the logical connection between theoretical derivation and experimental verification, which may reduce the persuasiveness of our proposed causal interpretation mechanism. In fact, our causal interpretation is not derived from simple heuristic correlations, but established on solid topological deformation theory and dual verification strategies. We further supplement and elaborate the rationality and reliability of the causal interpretation from corresponding perspectives as follows:

In terms of rationality, the causal decoupling in this study is grounded in geometric isomorphism and physical constraints, rather than heuristic correlations. Most existing studies adopt weight-driven heuristic interpretation frameworks, which implicitly assume that nodes with high weights necessarily correspond to major anomaly sources. Essentially, such methods simplify the anomalies of complex systems into a node importance ranking problem, making it impossible to distinguish heterogeneous anomaly sources such as the accumulation of weak endogenous anomalies and sudden exogenous disturbances. Fundamentally different from these approaches, our causal interpretation is established based on the geometric deformation of natural topological structures[6] with rigorous theoretical basis:

(1) **Causal measurement based on topological deformation of natural and predictive structures.** This study defines the natural topological structure of a system as the inherent and persistent connection pattern between sensors and observation timestamps under healthy operating conditions. The occurrence of anomalies drives the system to deviate from its natural structural state. To quantify such topological deformation, we adopt a Jaccard similarity-based topological discrepancy metric to identify anomalous relationships by measuring the geometric difference between the natural topology $\mathcal{A}$ and the predicted topology $\hat{\mathbf{A}}$. The rigorous mathematical formulation is presented as follows:

$$J_T(C_{\mathcal{A}}, C_{\mathbf{A}}) = 1 - \frac{|C_{\mathcal{A}} \cap C_{\mathbf{A}}|}{|C_{\mathcal{A}} \cup C_{\mathbf{A}}|}, \tag{1}$$

where $C_{\mathcal{A}}$ and $C_{\mathbf{A}}$ denote the candidate relationship sets of natural and predictive structures after $TopK(\cdot)$ sparsification, respectively. This metric ensures that the resulting anomaly interpretation originates from global perturbations of the system's endogenous structure, rather than heuristic speculation based on superficial local correlations.

(2) **Causal fidelity based on hyperbolic geometric isomorphism.** The volume of Euclidean space increases polynomially with radius, which fails to match the exponential expansion inherent to hierarchical structures and thus causes severe geometric distortion in node embedding. In contrast, the volume of hyperbolic space grows exponentially with radius ($V(r) \propto e^{\zeta r}$), and its hierarchical expansion characteristics form a precise geometric isomorphism with the hierarchical topology of complex networks. Accordingly, the topological discrepancies between natural and predictive structures can be mapped to hyperbolic space for geometrically faithful causal tracing, which eliminates spurious correlations caused by geometric distortion in Euclidean space and guarantees the fidelity of causal interpretation.The theoretical analysis for this conclusion is presented in **Response 3**.

(3) **Causal decoupling mechanism based on independent learning of spatio-temporal features.** Existing mainstream methods adopt joint learning schemes for spatio-temporal feature representation, which inevitably causes coupled diffusion of anomalous signals across temporal and spatial dimensions and produces a causal shielding effect that obscures true causal relationships. To address this issue, we introduce a system-level adaptive topology discovery mechanism and define a multivariate temporal dependency graph $G_t^{M_\zeta,t}$ and a multivariate spatial coupling graph $G_s^{M_\zeta,s}$. Independent optimization is performed to capture the inherent natural structures for temporal and spatial domains separately. This mechanism is guaranteed by a low-coupling constraint in the joint optimization objective, which effectively alleviates mutual interference across different interpretation dimensions and ensures the independence of spatio-temporal causal attribution. Nevertheless, purely data-driven learning is susceptible to data fluctuations, leading to structural oscillation and instability of the learned natural topology under varying data distributions. To tackle this problem, we further conduct numerical solving of the natural structure based on expectation and impose a physical constraint matrix to guide stable structural convergence. The formal definition is given as follows:

$$(\mathcal{A}^{\mathcal{M}_{\zeta,\mathcal{D}}})_{ij} = \mathbb{E}_{t\sim T}\left[(A_t^{\mathcal{M}_{\zeta,\mathcal{D}}} \odot M^{\mathcal{M}_{\zeta,\mathcal{D}}})_{ij}\right] = \frac{1}{T}\sum_{i=1}^{T}(A_t^{\mathcal{M}_{\zeta,\mathcal{D}}} \odot M^{\mathcal{M}_{\zeta,\mathcal{D}}})_{ij}, \tag{2}$$

where $(\mathcal{A}^{\mathcal{M}_{\zeta,\mathcal{D}}})_{ij}$ denotes the weight of the final stable natural system topology; $\mathbb{E}_{t\sim T}[\cdot]$ denotes the expectation operation over the full time series; $A_t^{\mathcal{M}_{\zeta,\mathcal{D}}}$ is the topological adjacency matrix learned at a single time step; $M^{\mathcal{M}_{\zeta,\mathcal{D}}}$ denotes the physical constraint matrix; and $\odot$ denotes the element-wise multiplication

operation. By integrating global temporal information via temporal expectation averaging and correcting structural deviations caused by data fluctuations through the physical constraint matrix, this formula effectively eliminates topological oscillation induced by single-time-step data disturbance and achieves stable convergence of the learned natural topology.

(4) At present, the anomaly interpretation mechanism proposed in this study mainly conducts causal analysis and correlation reasoning based on the spatio-temporal localization results of abnormal events, which still has certain limitations. Due to the lack of comprehensive domain expert prior knowledge and refined anomaly semantic annotation systems, the model cannot yet realize fully accurate and interpretable anomaly attribution as well as standardized textual interpretation. However, the spatio-temporal anomaly interpretation graph constructed in this work can provide clear and quantifiable structural evidence for anomaly tracing and mechanism analysis. In particular, it can effectively characterize the spatio-temporal propagation laws and complex dynamic evolution characteristics of multivariate anomalies within the system [1]. In future research, we will further integrate domain expert knowledge, establish refined anomaly semantic annotation systems, and improve the theoretical framework of model interpretability. We aim to promote anomaly interpretation from structural visual analysis toward accurate and standardized mechanism attribution, which will serve as one of our core research priorities in the follow-up work.

In terms of reliability, this study supports causal interpretation through quantitative verification based on topological discrepancy metrics and intuitive visualization evidence. To fully validate the correctness and reliability of the proposed anomaly causal interpretation, we conduct rigorous experimental verification from two dimensions: quantitative evaluation and qualitative visualization. The detailed validations are presented as follows:

(1) **High HitRate and NDCG of anomaly diagnosis(Fig. 3**). In the multivariate anomaly diagnosis task, causal interpretation methods relying solely on heuristic assumptions suffer from limited accuracy in anomaly sensor localization and ranking. In contrast, the proposed HAO model yields superior diagnostic performance within the hyperbolic space. Evaluated on two core metrics, HitRate@600% and NDCG@600%, the HAO model achieves 98.7% and 97.6%, respectively, substantially outperforming baseline methods whose average performances remain below 70% and 40%. In particular, the outstanding NDCG@600% results demonstrate the high-quality ranking capability of our model for anomaly sources. This further verifies that the proposed causal interpretation can effectively distinguish the primary and secondary relationships of system anomalies rather than providing coarse-grained heuristic guesses, thereby solidifying the accuracy and reliability of our causal attribution.

(2) **Reverse validation of label reliability via causal interpretation.** The reliability of causal interpretation is embodied in its precise characterization of anomaly boundaries. We visualize the label correction performance in Fig. 4 to validate the effectiveness of the proposed mechanism. In Euclidean space, inherent geometric distortion leads to a low overlap between the reconstructed anomaly regions and the original annotations, making it difficult to correct mislabeled and missing anomaly samples. By contrast, benefiting from geometric isomorphism, the hyperbolic space can effectively capture the spatio-temporal coupling anomaly patterns of complex systems. The reconstruction error precisely covers the real anomaly range and eliminates falsely labeled points that lack reliable reconstruction error support, achieving accurate alignment between reconstructed anomalies and real anomalies. This reverse validation demonstrates that our causal interpretation mechanism can provide objective and fine-grained anomaly boundary descriptions, further verifying the reliability of causal attribution.

(3) **Mining of typical causal structures for multivariate spatio-temporal anomalies.** Based on the proposed causal interpretation framework, we systematically investigate the internal correlation and propagation mechanisms of complex system anomalies through the multivariate spatio-temporal anomaly interpretation diagram in Fig. 5. Three typical types of causal structures are summarized and identified. This finding enables structured and hierarchical characterization of the evolutionary rules of multivariate anomalies, further demonstrating the completeness and systematicness of our causal interpretation. Multivariate anomalies exhibit three typical correlation structures in the spatio-temporal dimension, as shown in Fig. 5(A, B). The first category is a **co-occurrence relationship**, as shown in Fig. 5(A(a, b, c, l)) and Fig. 5(B(b, e, g, m)). The key coupling edges in the predicted structure and the natural structure exhibit a significant synchronous distribution, which is consistent with the original spatio-temporal structural pattern of the multivariate data. This indicates that the system remains stable in most submodules. The second category is a **partial concomitant phenomenon**, as shown in Fig. 5(A(d, e, f, g, h, i, n, o, p)) and Fig. 5(B(a, c, f, j, k, o, p)). Some key coupling edges in the predicted structure are missing or weakened in the original structure, which suggests that the corresponding subsystems may deviate from the normal operating mode and reflects local functional reconstruction or the cumulative effect of disturbances. The third category is an **unstructured disorder**, as shown in Fig. 5(A(d, j, k)) and Fig. 5(B(d, h, i, l, n)). The coupling patterns between the predicted structure and the original

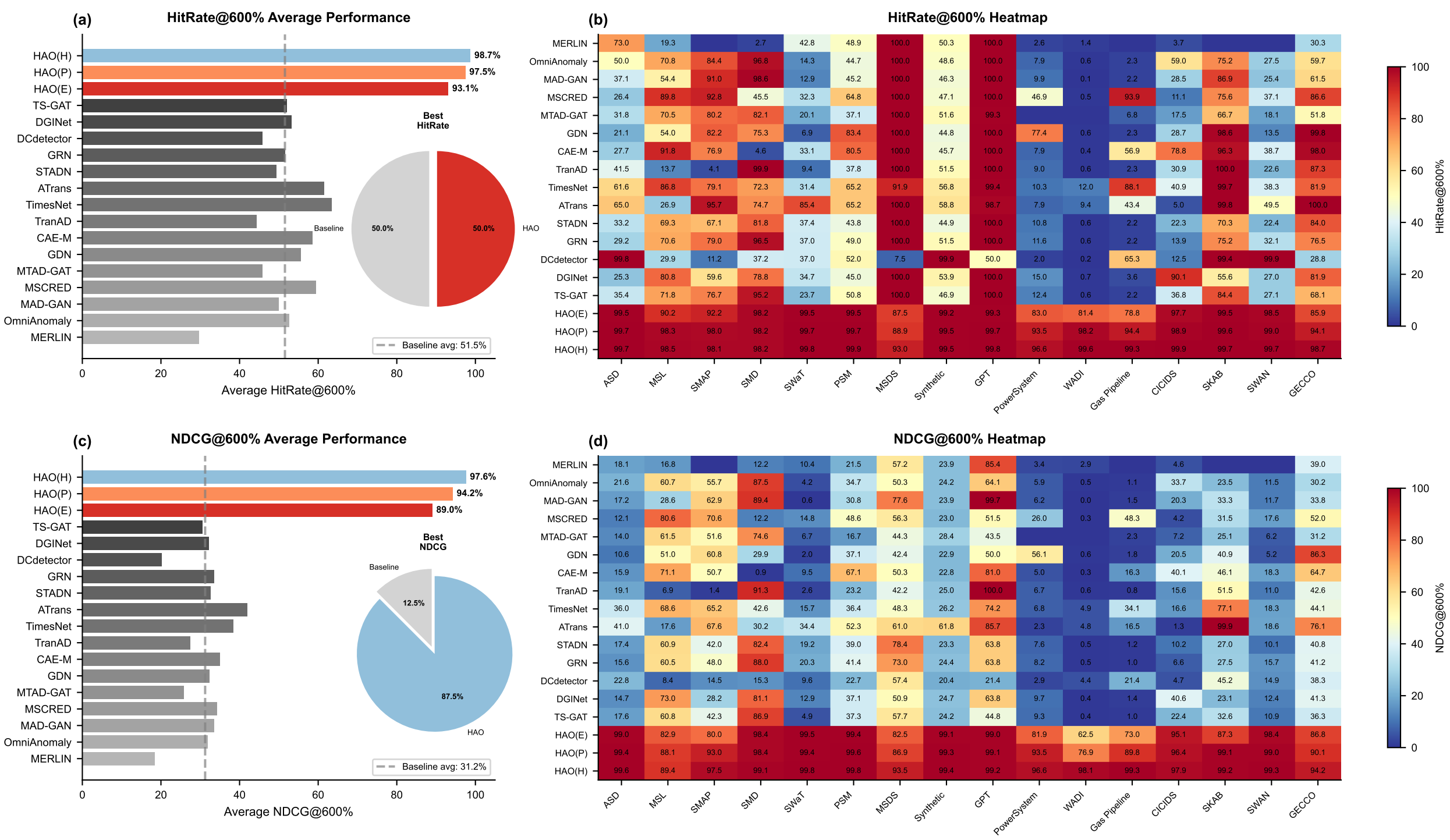


Figure 3: Comparative Analysis of Anomaly Diagnosis Performance for Multivariate Anomaly Detection Models

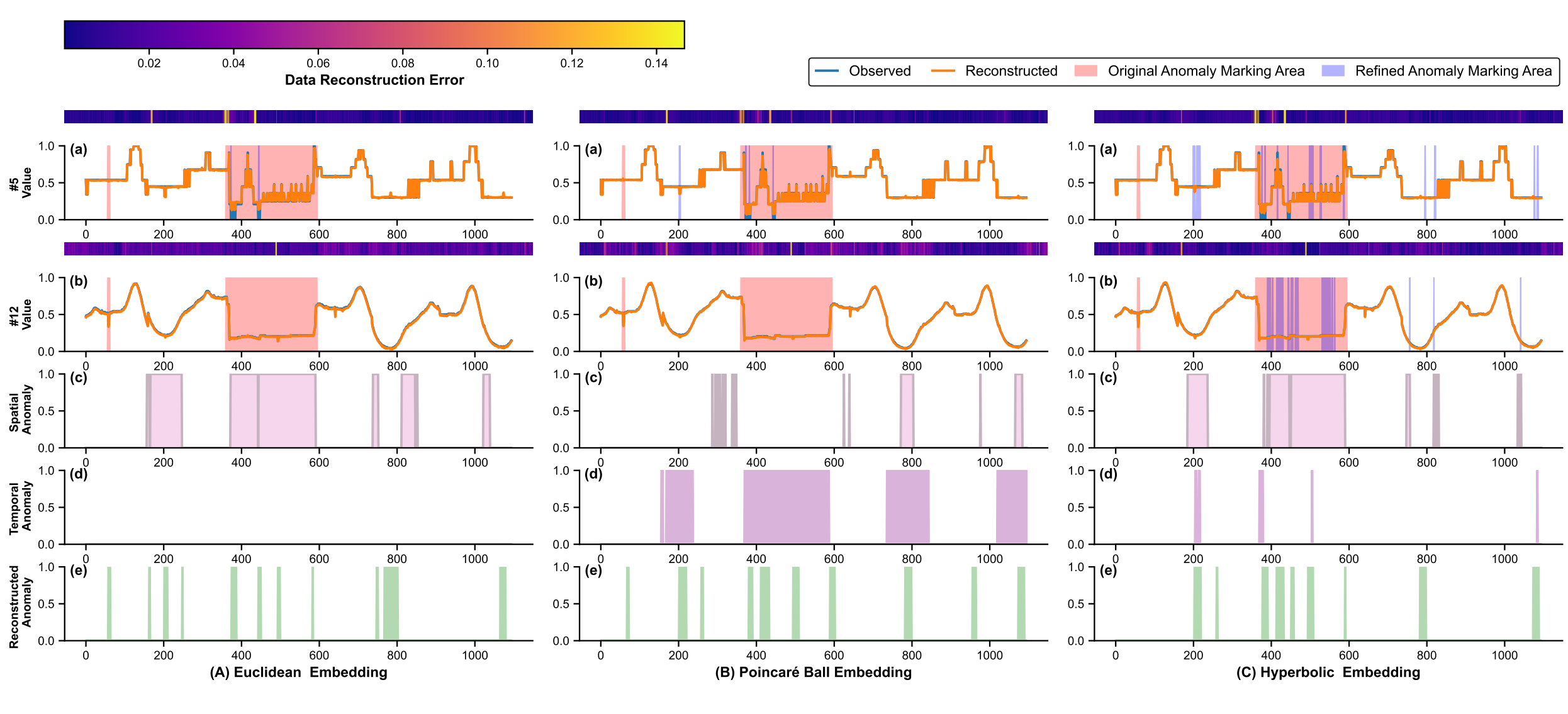


Figure 4: Analysis of HAO-series Models Correction Performance on Multivariate Anomaly Labelings

structure are highly fragmented without stable correspondence, which demonstrates that the system has entered a severely disordered state. The above classification reveals that the causes of multivariate anomalies can stem from either local changes in coupling relationships or global structural mismatch, which reflects the multi-level analytical capability of the proposed model for anomaly patterns. It also provides quantitative and structured support for subsequent anomaly localization and cause interpretation.

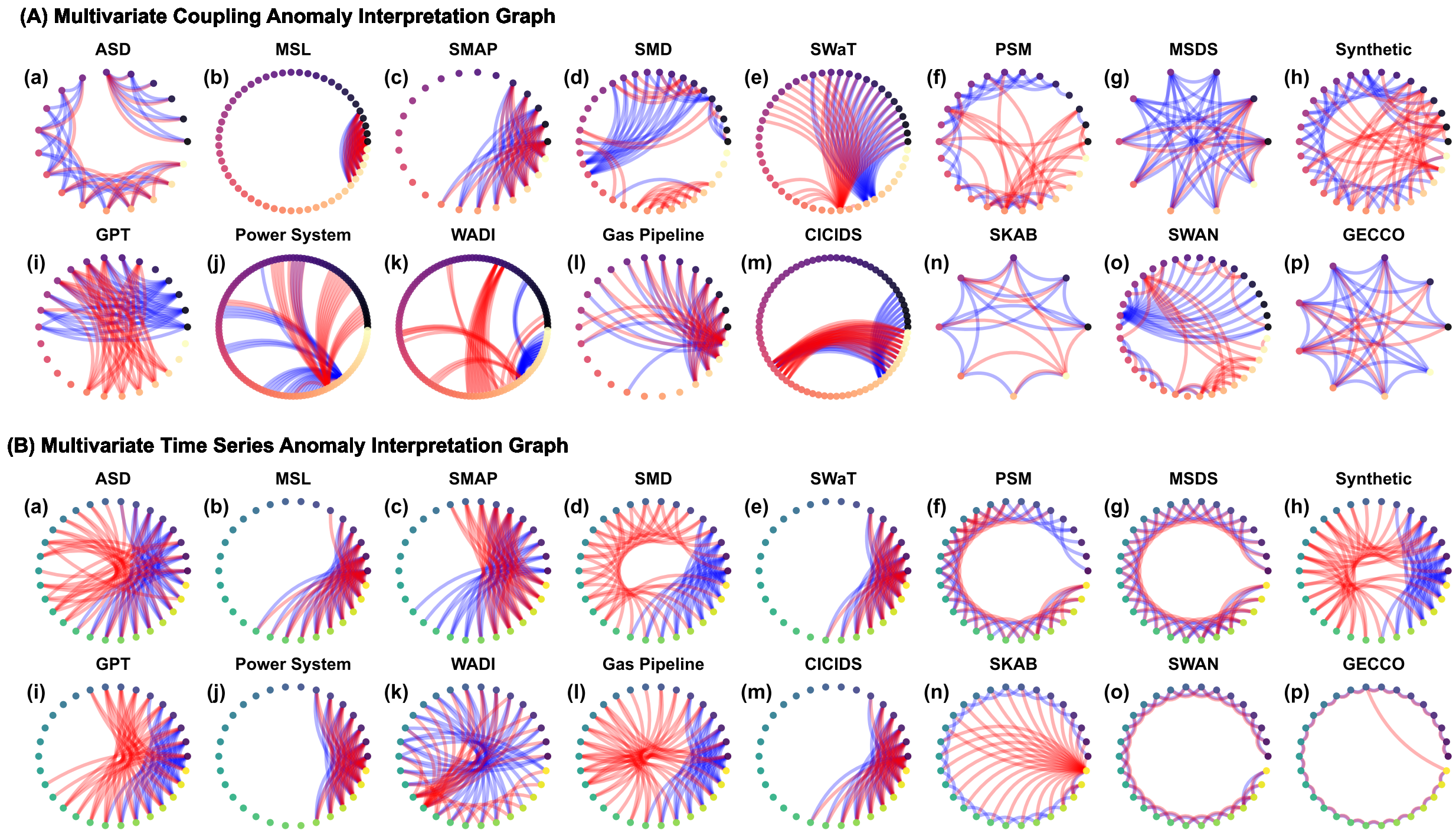


Figure 5: Multivariate Anomaly Interpretation. It shows an analysis of the effectiveness of multivariate temporal structure anomaly interpretation. When constructing the interpretation graph for multivariate temporal dependencies, the proposed method follows the procedure below. Using normal mode data with a sliding window size of 30, the natural temporal dependency structure is calculated and visualized, with blue lines representing these dependencies. Simultaneously, the predicted temporal structure is computed using the same sliding window size. Red indicates dependency strength and the top 30 strongest dependency relationships are visualized separately for comparative analysis. The analysis of the anomaly interpretation graph focuses on identifying co-occurrence relationships. If a predicted temporal dependency edge shown in red coexists with its corresponding normal mode edge in blue, this indicates the presence of accompanying phenomena and the dependency is classified as part of the normal pattern. Conversely, if the predicted edge lacks a corresponding blue edge, which means that the accompanying phenomenon is absent, it is identified as an anomalous association.

## References

[1] Battiston, F., Bick, C., Lucas, M. et al. Collective dynamics on higher-order networks. *Nat Rev Phys* **8**, 146–159 (2026).

**Revision 5:**

**[Section 3.2.4 Adaptive Hyperbolic Graph Structure Discovery(Main Text : Page 18)]**

$\cdots$ *Numerical solving of the natural structure is therefore performed again based on expectations and the physical constraint matrix is imposed to guide it toward convergence, as defined below:*

$$(\mathcal{A}^{\mathcal{M}_{\zeta,\mathcal{D}}})_{ij} = \mathbb{E}_{t\sim T}\left[(A_t^{\mathcal{M}_{\zeta,\mathcal{D}}} \odot M^{\mathcal{M}_{\zeta,\mathcal{D}}})_{ij}\right] = \frac{1}{T}\sum_{i=1}^{T}(A_t^{\mathcal{M}_{\zeta,\mathcal{D}}} \odot M^{\mathcal{M}_{\zeta,\mathcal{D}}})_{ij}. \tag{3}$$

where $(\mathcal{A}^{\mathcal{M}_{\zeta,\mathcal{D}}})_{ij}$ denotes the weight of the final stable natural system topology; $\mathbb{E}_{t\sim T}[\cdot]$ represents the expectation operation over the full time series; $A_t^{\mathcal{M}_{\zeta,\mathcal{D}}}$ is the topological adjacency matrix learned at a single time step; $M^{\mathcal{M}_{\zeta,\mathcal{D}}}$ denotes the physical constraint matrix; and $\odot$ denotes the element-wise multiplication operation. By integrating global temporal information via temporal expectation averaging and correcting structural deviations caused by data fluctuations through the physical constraint matrix, this formula effectively eliminates topological oscillation induced by single-time-step data disturbance and achieves stable convergence of the learned natural topology.$\cdots$

**[Section 3.4.1 Topological Difference Metric(Main Text : Page 21)]**

*⋯ a topological discrepancy metric grounded in the Jaccard similarity is devised, defined as follows:*

$$J_T(C_{\mathcal{A}}, C_A) = 1 - \frac{|C_{\mathcal{A}} \cap C_A|}{|C_{\mathcal{A}} \cup C_A|}. \tag{4}$$

where $C_{\mathcal{A}}$ and $C_{\mathbf{A}}$ denote the candidate relationship sets of natural and predictive structures after $TopK(\cdot)$ sparsification, respectively. This metric ensures that the resulting anomaly interpretation originates from global perturbations of the system's endogenous structure, rather than heuristic speculation based on superficial local correlations. ⋯

**[Section 4.4.1 Comparative Performance Analysis of Anomaly Diagnosis(Main Text : Page 37-38)]**

⋯ *In contrast, the average HitRate@600% of all baseline models is below 70%.* ATrans and TimesNet achieve stable average HitRate@600% values ranging from 61.6% to 63.5% across all experimental datasets yet remain within the low-performance range of traditional comparison models. ⋯

⋯ *However, existing baseline models mostly rely on static structures or the fusion learning of spatio-temporal features and lack fine-grained spatio-temporal localization for anomalies.* ATrans captures long-range temporal dependencies but neglects multivariate spatial topology. DCdetector screens local anomalies without global spatio-temporal logic. TimesNet extracts multi-scale temporal features but fails to decouple multivariate coupled anomalies. *They can only identify anomaly locations based on sensor information during anomaly reconstruction, which results in limited generalization ability.* ⋯

**6. The main figure (Figure 8) contains excessive details and dense textual annotations, which significantly affect its readability. It is recommended to simplify the textual content and improve the visual organization by clearly separating different functional regions (e.g., modules or stages). This would help readers better understand the overall framework.**

**Response 6:** We sincerely appreciate the valuable comments. In response to the reviewer's concerns that the original main figure (Figure 8) contained excessive module details, dense textual annotations and poor readability, we have substantially revised and optimized the figure in this revision. Specifically, we have greatly streamlined redundant textual descriptions, reconstructed the overall layout and visual hierarchy, and adopted differentiated color matching and partitioned border designs to clearly distinguish functional modules and technical stages. These modifications enable a well-organized and hierarchical presentation of the model architecture. The optimized full model framework is shown in Fig. 6.

In the data preparation stage, this study first adopts a sliding window sampling strategy (Fig. 8(A.a)) to continuously sample the observed time series using a sliding window with step size $j$ and window length $W$. This generates multivariate spatio-temporal data blocks formulated as $S_t = \{x_{i,t+j} \mid 1 \le i \le N,\ 0 \le j < W\}$ which preserves local temporal dependencies while mitigating the risk of global temporal information fragmentation. Based on, this study further constructs multivariate time series data and multivariate coupling graph data, respectively (Fig. 6(A.b)). All structured data are then projected into hyperbolic space via the initial hyperbolic embedding module (Fig. 6(A.c)), establishing a solid data foundation for discovering the natural spatio-temporal structures of multivariate time series. In the dual adaptive hyperbolic pre-training stage, the hyperbolic linear transformation module is first employed to suppress noise interference from raw data (Fig. 6(B.a)). Subsequently, the hyperbolic attention message aggregation mechanism (Fig. 6(B.b)) leverages spatial and temporal attention units to capture the natural dynamic topological representations $\mathcal{A}_s$ and $\mathcal{A}_t$ underlying multivariate coupling correlations and temporal dependencies. To enable incremental optimization of the inherent topological structure, we design multivariate coupling memory units and temporal dependency memory units. The topological representations are iteratively updated via the incremental steady-state topology solving module (Fig. 6(B.c)), followed by multivariate data reconstruction using a multi-scale signal parsing attention mechanism. Finally, the joint optimization objective (Fig. 6(B.d)) is optimized to achieve dynamic updating of natural multivariate spatio-temporal structures and stable representation learning in hyperbolic space.

In the multivariate anomaly detection stage, anomaly severity is first quantified from three perspectives: coupling structure discrepancy, temporal structure discrepancy, and data reconstruction discrepancy (Fig. 6(C.a)), enabling the quantitative calculation of multivariate spatio-temporal anomalies. Subsequently, the graph relation pruning module (Fig. 6(C.b(1))) and robust constraint optimization module (Fig. 6(C.b(2))) in the multivariate anomaly recognition procedure are adopted to screen sparse graph relations and anomaly factor sets. This procedure achieves the discrimination between explainable and unexplainable anomalies (Fig. 6(C.b(3))) and outputs reliable anomaly evaluation scores. For anomaly tracing, multivariate anomaly diagnosis is further performed (Fig. 6(C.c)). With the spatio-temporal coordinate localization of multivariate anomalies (Fig. 6(C.c(1))), the spatio-temporal positions of anomalies and the correlations among key variables are determined, and anomaly causal chains are constructed by integrating candidate relations and predicted topological structures. Finally, a multivariate spatio-temporal anomaly interpretation graph is established (Fig. 6(C.d(1, 2))) to interpret the propagation paths and root-cause correlations of detected anomalies.

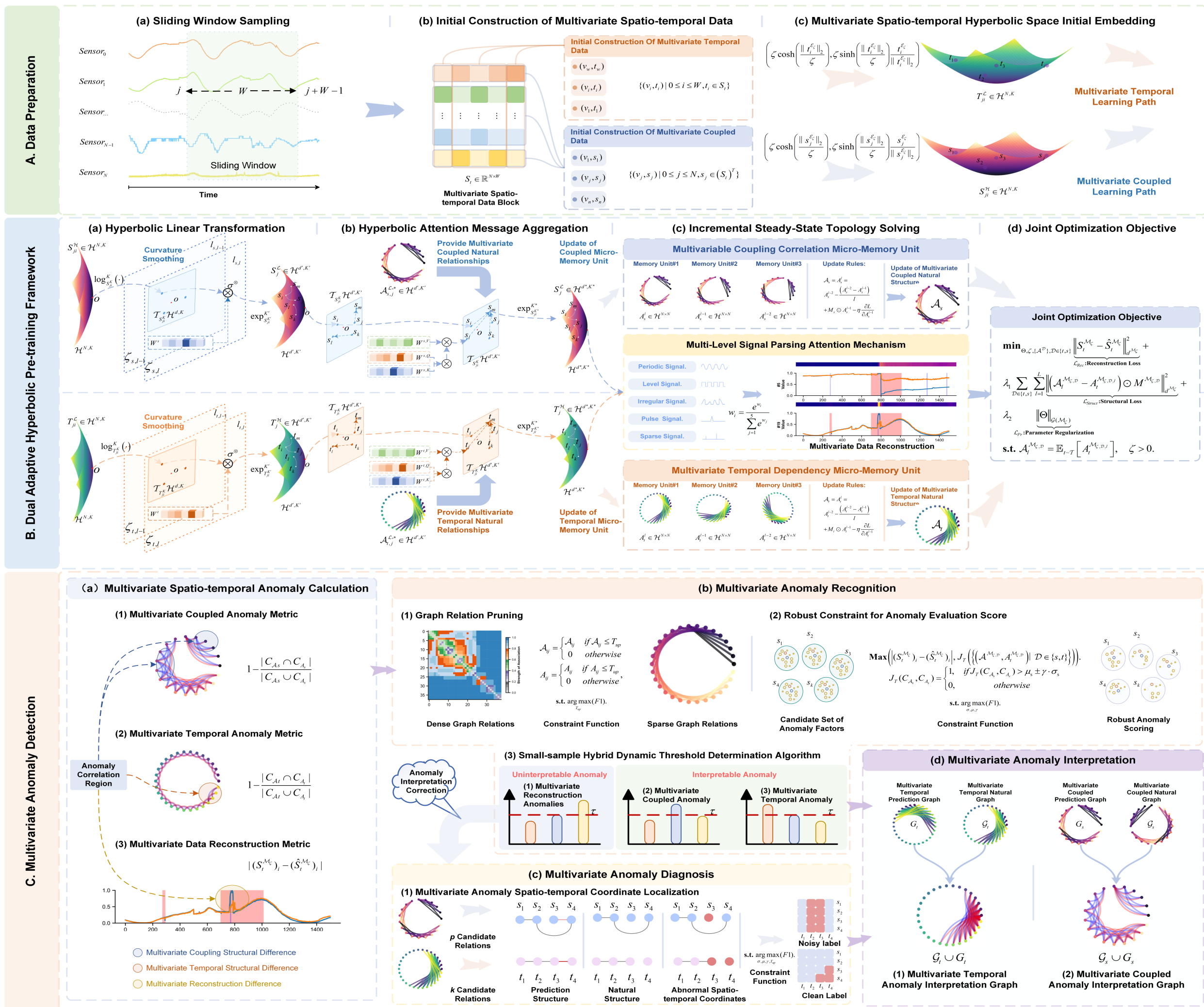


Figure 6: Hyperbolic Adaptive Spatial-Aware Multivariate Temporal Anomaly Detection Model Framework

**Revision 6:**

**[Section 3 Methodology (Main Text: Page 13-14)]**

$\cdots$ In the data preparation stage, this study first adopts a sliding window sampling strategy (Fig. 6(A.a)) to continuously sample the observed time series using a sliding window with step size $j$ and window length $W$. This generates multivariate spatio-temporal data blocks formulated as $S_t = \{x_{i,t+j} \mid 1 \leq i \leq N,\ 0 \leq j < W\}$ which preserves local temporal dependencies while mitigating the risk of global temporal information fragmentation. Based on, this study further constructs multivariate time series data and multivariate coupling graph data, respectively (Fig. 6(A.b)). All structured data are then projected into hyperbolic space via the initial hyperbolic embedding module (Fig. 6(A.c)), establishing a solid data foundation for discovering the natural spatio-temporal structures of multivariate time series. In the dual adaptive hyperbolic pre-training stage, the hyperbolic linear transformation module is first employed to suppress noise interference from raw data (Fig. 6(B.a)). Subsequently, the hyperbolic attention message aggregation mechanism (Fig. 6(B.b)) leverages spatial and temporal attention units to capture the natural dynamic topological representations $\mathcal{A}_s$ and $\mathcal{A}_t$ underlying multivariate coupling correlations and temporal dependencies. To enable incremental optimization of the inherent topological structure, we design multivariate coupling memory units and temporal dependency memory units. The topological representations are iteratively updated via the incremental steady-state topology solving module (Fig. 6(B.c)), followed by multivariate data reconstruction using a multi-scale signal parsing attention mechanism. Finally, the joint optimization objective (Fig. 6(B.d)) is optimized to achieve dynamic updating of natural multivariate spatio-temporal structures and stable representation learning in hyperbolic space. $\cdots$

## 3 Reviewer #4 (Remarks on code availability):

**The codes look well organized.**

**Response :** We sincerely thank the reviewer for the valuable suggestions regarding code organization.

## 4 Reviewer #5 (Remarks to the Author):

**1. This paper proposes using hyperbolic geometry for multivariate time series anomaly detection (TSAD). The core intuition that sensor (or univariate TS) coupling networks exhibit hierarchical structure better captured by hyperbolic space than Euclidean space is reasonable. However, the execution diverges significantly from what the TSAD community has come to expect of rigorous work in this area. The strongest contributions in recent TSAD research share a common trait: methodological focus, center on one clear idea, validate it thoroughly and let the results speak. This manuscript takes the opposite approach, simultaneously introducing five components, a label correction framework, a new thresholding algorithm and an interpretability pipeline, spread across 42 pages. The sheer volume makes it difficult to isolate what actually drives the reported improvements.**

**Response 1:** We sincerely appreciate the reviewer's valuable comments and insightful guidance. In response to the concerns raised regarding insufficient elaboration of the differences between this study and existing multivariate anomaly detection methods, the relatively large number of independent modules, and the ambiguous experimental validation logic caused by the complex module design, we have carefully sorted out all issues and provided item-by-item responses accordingly. To facilitate subsequent logical elaboration and theoretical clarification, we systematically divide the overall task into three core components based on the proposed multivariate anomaly detection paradigm: anomaly recognition, anomaly diagnosis, and anomaly interpretation. The detailed task framework is shown in Fig. 7.

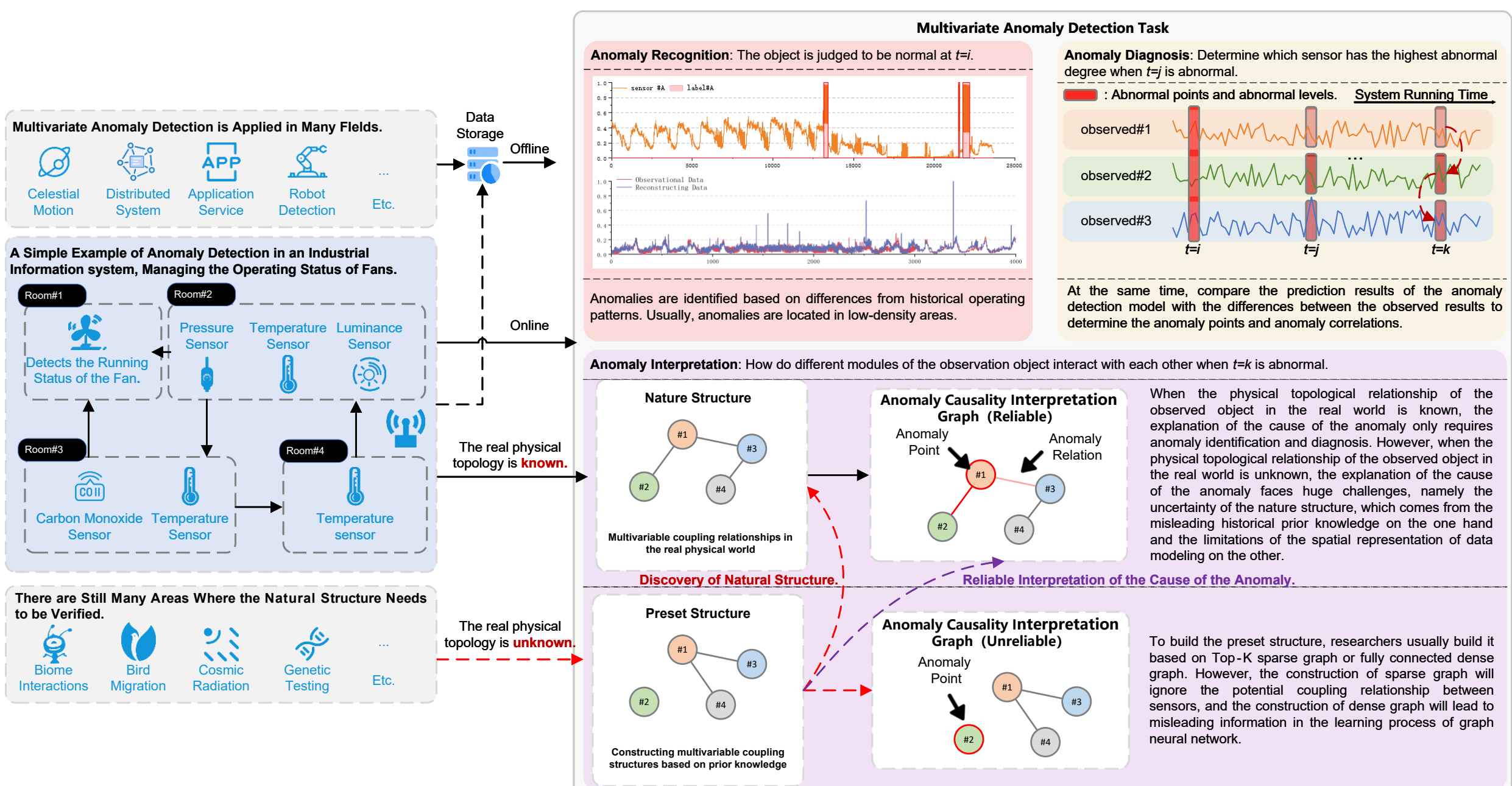


Figure 7: **Multivariate Anomaly Detection Framework.** Currently, multivariate anomaly detection has evolved into a three-tier progressive framework [1]: at the recognition layer, advanced pattern recognition algorithms screen out abnormal segments from massive time series data; at the diagnosis layer, spatio-temporal dynamic relationship analysis is used to precisely locate the spatio-temporal coordinates of anomalies and determine conditional thresholds; at the interpretation layer, techniques such as causal reasoning and knowledge graphs reveal the multi-level causal network underlying anomalies, encompassing both direct triggers and deep-seated systemic defects. This "recognition–diagnosis–interpretation" paradigm not only bridges the gap from observable phenomena to essential understanding, but also provides actionable decision support for the continuous optimization of production systems.

(1) **Supplementary explanation of the differences between our method and existing multivariate anomaly detection studies.** Recent years have witnessed substantial progress in time series anomaly detection. Benefit-

ing from abundant coarse-grained annotations, existing approaches have achieved mature performance in both univariate and multivariate anomaly recognition tasks. Nevertheless, due to the scarcity of fine-grained labels, most anomaly diagnosis studies are only validated on a limited number of finely annotated datasets such as SMD[2] and MSDS[3]. More importantly, a unified standard labeling system supporting fine-grained anomaly interpretation is still absent in the community. Furthermore, label quality issues have long been recognized as a critical challenge in time series anomaly detection[4][5][6]. In particular, ambiguous anomaly boundaries and inherent human labeling biases are prevalent and difficult to eliminate completely in practical applications. To mitigate performance fluctuations caused by inconsistent label quality, existing studies generally adopt robust evaluation metrics (e.g., VUS-PR and Range-ROC) to passively adapt to inherent labeling flaws[5][6].

Different from these passive compensation strategies, our study corrects noisy annotations actively from the perspective of topological deformation, rather than relying solely on robust metrics to tolerate label noise. Considering the widespread risks of mislabeling, missing labels, and ambiguous anomaly boundaries in real-world scenarios[4], this study propose a fine-grained label calibration strategy based on multivariate spatio-temporal coordinate localization. This strategy effectively reduces the interference of labeling biases on model evaluation, provides a feasible anomaly diagnosis solution for datasets lacking fine-grained annotations, and provides solid structural evidence for causal anomaly interpretation, thereby establishing a distinct technical route from existing works.

(2) **In response to the reviewer's comment that the adoption of numerous independent modules may hinder readers from fully understanding the design motivation and research significance of each module, we provide the corresponding explanations and revisions as follows:**

This study focuses on three closely related and progressive tasks, namely multivariate anomaly recognition, anomaly diagnosis and anomaly interpretation. All modules are designed around the core research direction of modeling inherent multivariate spatio-temporal structures, and each independent module is developed to address specific practical challenges and achieve definite optimization goals with no redundant functional designs. Furthermore, to enhance the readability of the overall model framework, this paper has redrawn and optimized the model structure diagram. Redundant text has been removed, and core functional modules are clearly delineated through color-coded partitioning and border grouping. For details, please refer to Fig. 8.

(a) In terms of anomaly recognition, this study adopts an incremental topology optimization strategy to suppress the inherent structural degradation of the multivariate spatio-temporal topology. A multi-scale signal-aware attention mechanism is proposed to improve the reconstruction stability of sparse, periodic, and irregular time-series signals, thereby boosting the overall multivariate data reconstruction capability and maintaining the stable optimization of natural spatio-temporal structures. Meanwhile, hyperbolic representation learning is introduced to alleviate feature distortion caused by traditional Euclidean space embeddings.

(b) For multivariate anomaly diagnosis with limited fine-grained labels, this study designs a spatio-temporal anomaly localization algorithm to perform precise fine-grained label annotation.

(c) To overcome the poor readability and difficulty in analyzing dense anomaly reasoning graphs, this study integrates graph structure pruning and robust constrained optimization modules, thereby further enhancing the interpretability of multivariate spatio-temporal anomaly analysis.

(3) **In response to the reviewer's concern that the large number of independent modules may lead to insufficient and ambiguous experimental support for each component, this study provides systematic supplementary explanations as follows:**

This study centers on the core task of multivariate time series anomaly detection, focusing on both theoretical expansion and practical application of inherent multivariate spatio-temporal structure modeling. All experimental designs strictly correspond to the proposed model framework. As shown in Fig. 8, this study conducts comprehensive and hierarchical experimental validations from three progressive task dimensions, providing solid and distinguishable experimental support for every designed module.

(a) In the multivariate anomaly recognition task, this study quantitatively compares the accuracy, precision, VUS_ROC, and F1-score before and after label calibration, verifying the optimal performance of the proposed adaptive threshold search strategy. Furthermore, ROC and P-R metrics are adopted to validate the model's superiority under threshold-free evaluation protocols. In addition, extensive ablation experiments are conducted to individually verify the contribution of each functional module. This study also thoroughly investigates the influence of different embedding spaces on anomaly recognition performance and validate the superiority of our reconstruction mechanism through multi-scale signal reconstruction comparisons. Moreover, the temporal and spatial complexity is analyzed to verify the lightweight nature of our model. Finally, hyperparameter experiments are performed to explore the effects of validation ratio, sliding

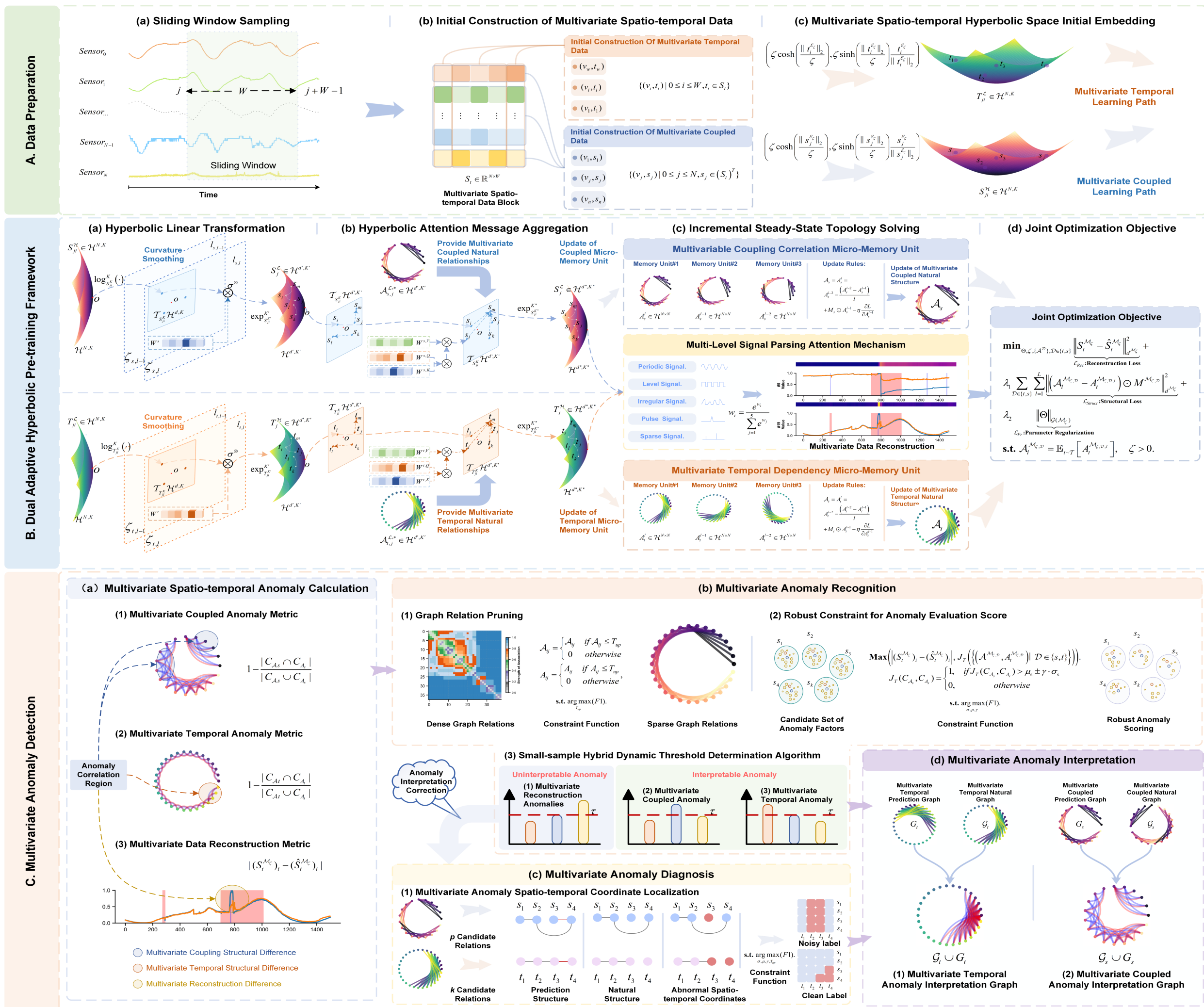


Figure 8: Hyperbolic Adaptive Spatial-Aware Multivariate Temporal Anomaly Detection Model Framework

window size, threshold search algorithms, and hyperbolic curvature variations, which fully demonstrate the stability and adaptability of our method.

(b) In the multivariate anomaly diagnosis task, this study adopts HitRate and NDCG to quantitatively evaluate fine-grained anomaly localization and ranking performance, thereby verifying the performance gains brought by the proposed label calibration strategy. **To the best of our knowledge, few existing methods can simultaneously achieve fine-grained label calibration and quantitative anomaly diagnosis. This study further compares label calibration performance under different representation spaces and analyzes the underlying influencing factors, demonstrating the unique advantages of hyperbolic geometric isomorphism in fine-grained anomaly diagnosis and label denoising.**

(c) In the multivariate anomaly interpretation task, this study systematically validates the interpretability of our method from three perspectives. First, the model's capability to capture complex spatio-temporal correlations is verified, providing structural support for mining weak and hidden anomalies. Second, this study summarizes the evolutionary characteristics[7] of inherent spatio-temporal structures, offering valuable insights for reverse analysis of internal system mechanisms. Finally, this study constructs interpretable spatio-temporal anomaly propagation graphs to analyze anomaly diffusion patterns and internal coupling characteristics, achieving a complete closed-loop pipeline from anomaly detection to mechanism interpretation.

Therefore, this study conducts a systematic and comprehensive exploration covering multivariate anomaly recognition, anomaly diagnosis and anomaly interpretation. All modules are not simply combined, but mutually coordinated and complementary. This work aims to improve the interpretable modeling capability in the field of multivariate anomaly detection, and establishes a complete research framework and practical solutions from the data level, model level and application level. We sincerely appreciate the constructive comments from the reviewers.

## References


[1] Li, G. & Jung, J. J. Deep learning for anomaly detection in multivariate time series: approaches, applications, and challenges. *Inform. Fusion.* **91**, 93–102 (2023).

[2] Su, Y. et al. Robust anomaly detection for multivariate time series through stochastic recurrent neural network. In *Proc. ACM SIGKDD Conf. Knowl. Discov. Data Min.* 2828–2837 (2019).

[3] Nedelkoski, S. et al. Multi-source distributed system data for AI-powered analytics. In *European Conference on Service-Oriented and Cloud Computing* 161–176 (Springer, 2020).

[4] Wu, R. J. and Keogh, E. J. Current time series anomaly detection benchmarks are flawed and are creating the illusion of progress. *IEEE T. Knowl. Data En.* **35**, 2421–2429 (2021).

[5] Paparrizos, J. et al. Volume under the surface: a new accuracy evaluation measure for time-series anomaly detection. In *Proc. VLDB Endow.* **15**, 2774–2787 (2022).

[6] Huet, A. et al. Local evaluation of time series anomaly detection algorithms. In *Proc. ACM SIGKDD Conf. Knowl. Discov. Data Min.* 1–11 (2022).

[7] Battiston, F., Bick, C., Lucas, M. et al. Collective dynamics on higher-order networks. *Nat Rev Phys* **8**, 146–159 (2026).


**Revision 1:**

**[Section 3 Methodology (Main Text: Page 13-14)]**

$\cdots$ In the data preparation stage, this study first adopts a sliding window sampling strategy (Fig. 8(A.a)) to continuously sample the observed time series using a sliding window with step size $j$ and window length $W$. This generates multivariate spatio-temporal data blocks formulated as $S_t = \{x_{i,t+j} \mid 1 \leq i \leq N, \, 0 \leq j < W\}$ which preserves local temporal dependencies while mitigating the risk of global temporal information fragmentation. Based on, this study further constructs multivariate time series data and multivariate coupling graph data, respectively (Fig. 8(A.b)). All structured data are then projected into hyperbolic space via the initial hyperbolic embedding module (Fig. 8(A.c)), establishing a solid data foundation for discovering the natural spatio-temporal structures of multivariate time series. In the dual adaptive hyperbolic pre-training stage, the hyperbolic linear transformation module is first employed to suppress noise interference from raw data (Fig. 8(B.a)). Subsequently, the hyperbolic attention message aggregation mechanism (Fig. 8(B.b)) leverages spatial and temporal attention units to capture the natural dynamic topological representations $\mathcal{A}_s$ and $\mathcal{A}_t$ underlying multivariate coupling correlations and temporal dependencies. To enable incremental optimization of the inherent topological structure, this study designs multivariate coupling memory units and temporal dependency memory units. The topological representations are iteratively updated via the incremental steady-state topology solving module (Fig. 8(B.c)), followed by multivariate data reconstruction using a multi-scale signal parsing attention mechanism. Finally, the joint optimization objective (Fig. 8(B.d)) is optimized to achieve dynamic updating of natural multivariate spatio-temporal structures and stable representation learning in hyperbolic space.

In the multivariate anomaly detection stage, anomaly severity is first quantified from three perspectives: coupling structure discrepancy, temporal structure discrepancy, and data reconstruction discrepancy (Fig. 8(C.a)), enabling the quantitative calculation of multivariate spatio-temporal anomalies. Subsequently, the graph relation pruning module (Fig. 8(C.b(1))) and robust constraint optimization module (Fig. 8(C.b(2))) in the multivariate anomaly recognition procedure are adopted to screen sparse graph relations and anomaly factor sets. This procedure achieves the discrimination between explainable and unexplainable anomalies (Fig. 8(C.b(3))) and outputs reliable anomaly evaluation scores. For anomaly tracing, multivariate anomaly diagnosis is further performed (Fig. 8(C.c)). With the spatio-temporal coordinate localization of multivariate anomalies (Fig. 8(C.c(1))), the spatio-temporal positions of anomalies and the correlations among key variables are determined, and anomaly causal chains are constructed by integrating candidate relations and predicted topological structures. Finally, a multivariate spatio-temporal anomaly interpretation graph is established (Fig. 8(C.d(1, 2))) to interpret the propagation paths and root-cause correlations of detected anomalies. $\cdots$

**2. The evaluation protocol does not align with current community standards. Since Wu & Keogh (2022) demonstrated that traditional point-adjust F1 creates an illusion of progress, the field has moved toward more robust metrics: VUS (Paparrizos et al., VLDB 2022), range-based and affiliation-based measures (Huet et al., 2022). None of these appear here. More critically, the model corrects the anomaly labels using its own learned topology, then evaluates all methods on those corrected labels. This circularity undermines the reported F1-scores of 0.99+, which are already unusually high for datasets like WADI and CICIDS where the community consistently reports much lower perfs. The benchmark suite itself omits the most prominent recent methods, including Anomaly Transformer, DCdetector and TimesNet, while relying heavily on baselines from 2019.**

**Response 2:** We sincerely appreciate the reviewers' careful review as well as their valuable and instructive comments on this work. In this revised manuscript, we have conducted comparative experiments against mainstream multivariate anomaly detection models including Anomaly Transformer(ATrans)[1], DCdetector[2] and TimesNet[3], and supplemented sufficient experimental validations and result analyses based on relevant evaluation metrics. In the following content, we respond point by point to all concerns raised by the reviewers, with reference to the comparative results in Fig. 9 and the experimental performance of all models on the WADI[4] and CICIDS[5] datasets shown in Tab. 2.

(1) **Selection of Evaluation Metrics and Alignment with Domain General Standards.** Multivariate anomaly detection is inherently confronted with severe class imbalance, where normal samples dominate the entire dataset. Traditional equally-weighted metrics tend to overestimate overall model performance and cannot objectively reflect the detection capability for sparse anomalies. To eliminate evaluation bias arising from imbalanced class distribution, this study adopts the inverse class frequency-based weighting strategy to calculate weighted precision, recall and F1-score, thus achieving reliable quantitative evaluation [6, 7]. In addition, this study newly introduces Volume Under the Surface of ROC (VUS_ROC) [8] in this revision to further enrich the performance evaluation dimensions for multivariate anomaly detection. According to the experimental results of F1-score and VUS_ROC (Fig. 9(a,j)), the average VUS_ROC values of all models across full datasets are generally higher than weighted F1-scores. This is mainly because our weighted balanced F1-score effectively avoids the performance overestimation caused by traditional point-wise correction strategies.

(2) **In response to the reviewers' comments, we have supplemented comparative experiments with representative baselines including ATrans, DCdetector and TimesNet.** Experimental results show that TimesNet achieves competitive overall performance in terms of precision, recall, F1-score and VUS_ROC, which is highly consistent with the benchmark results released in the mainstream open-source time series anomaly detection repository[1].

After label correction, obvious performance gains in F1-score, Precision and Recall are observed for ATrans, DCdetector and TimesNet, as shown in Figs. 9(b, e, h, k). From the VUS_ROC performance density curve (Fig. 9(l)), we can observe that the peak and mean values of VUS_ROC remain stable before and after label correction without drastic performance fluctuations. In summary, the weighted balanced F1-score adopted in this work can effectively distinguish the anomaly detection performance of different models. As a threshold-independent metric, VUS_ROC can stably and faithfully reflect the inherent anomaly mining capability for multivariate time-series data. The combination of the two metrics not only verifies the rationality and reliability of our evaluation system, but also strongly demonstrates the superior stability of the proposed method.

(3) **On the Circular Reasoning between Label Revision and Model Evaluation.** The label revision strategy proposed in this work is designed based on the inherent topological characteristics and real anomaly boundaries of datasets, which is completely independent of model prediction outputs. Therefore, no circular reasoning exists in the whole evaluation procedure. To clarify this concern, we have supplemented Tab. 2 to report full precision, recall, F1-score and VUS_ROC results of all comparison models on the WADI and CICIDS datasets before and after label revision. It is evident that all baseline models exhibit consistent performance variations after revision, and such adjustment effect is not limited to our proposed HAO variants. On the WADI dataset, MSCRED yields an original precision of 0.6013 and a corrected precision of 0.4680, while its original F1-score of 0.5264 drops to 0.4182 after revision. By contrast, the F1-score of TranAD rises from 0.5448 to 0.6161. Such variation trend align well with the overall tendencies shown in Figs. 9(c, f, i, l), which proves that label revision is an objective dataset-oriented standard calibration rather than targeted optimization for specific models. All reported F1-score results are fully supported by solid experimental evidence. After label revision, HAO obtains an F1-score of 0.9943 on WADI and 0.9995 on CICIDS. Such outstanding detection performance has been validated over sixteen diverse datasets, rather than accidental outcomes on individual datasets.

(4) **Completeness of Benchmark Datasets and Comparison Methods.** The benchmark system constructed in this work covers mainstream time series anomaly detection algorithms proposed in recent years instead of

[1]The open-source benchmark library is available at https://github.com/thuml/Time-Series-Library.

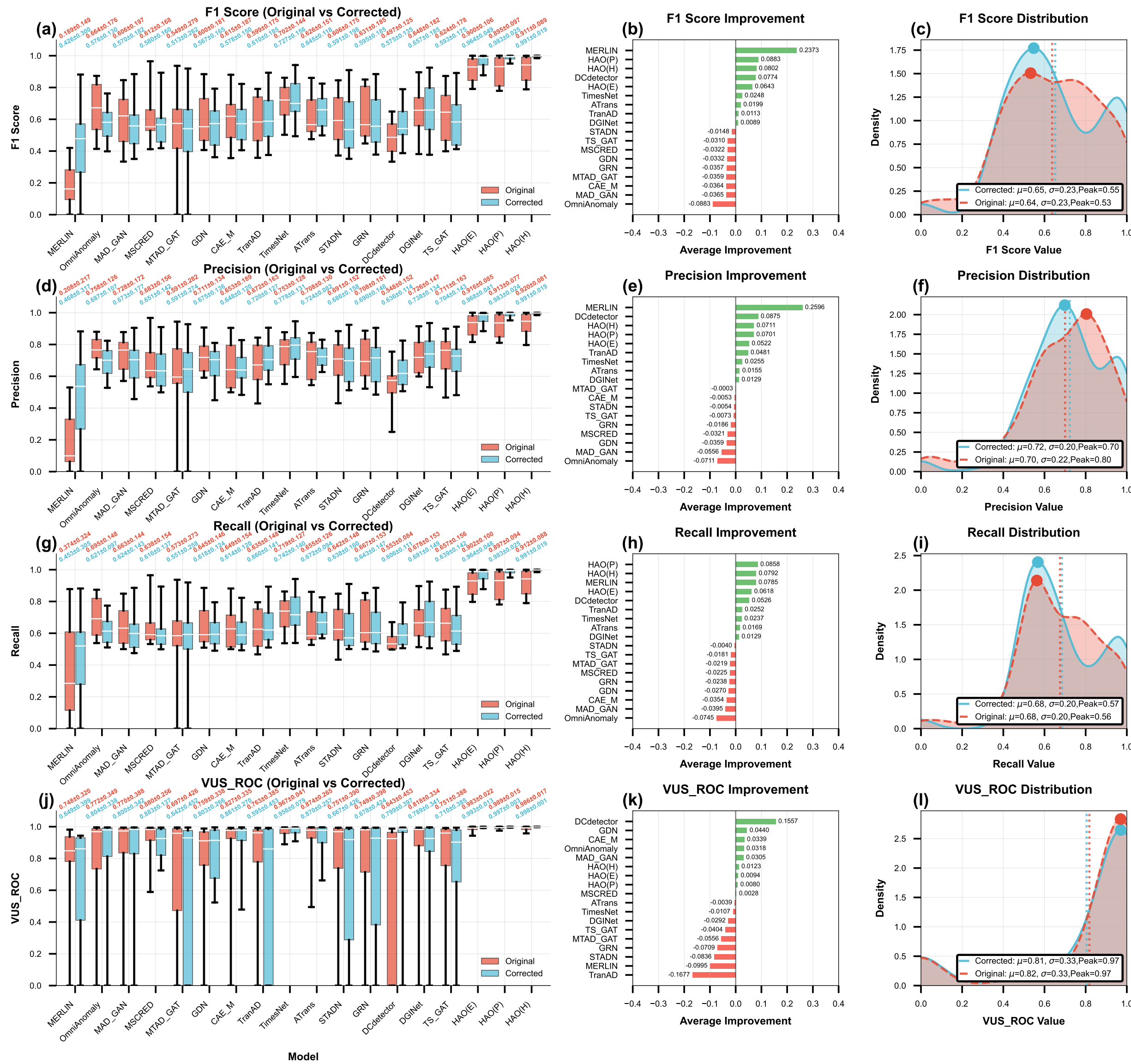


Figure 9: Comprehensive Comparative Analysis of Multivariate Anomaly Detection Model Performance Based on F1-score, Precision, Recall and VUS_ROC Before and After Anomaly Label Correction. Figs. 9(a, d, g, j) show box plots of average performance across different datasets; Figs. 9(b, e, h, k) show the performance improvement effects; Figs. 9(c, f, i, l) show the performance density distributions.

merely employing outdated baselines, which ensures the comprehensiveness of comparative experiments. The selected comparison methods consist of both state-of-the-art models and classic approaches, including recently proposed high-performance models ATrans, DCdetector and TimesNet published in top-tier conferences, as well as widely acknowledged classic baselines such as MSCRED, MAD-GAN and CAE-M. According to the overall results of precision, recall, F1-score and VUS_ROC in Figs. 9(a, d, g, j) and the detailed quantitative results on WADI and CICIDS in Tab. 2, our proposed HAO series methods consistently outperform all competing methods across all core evaluation metrics and maintain stable superiority on diverse datasets, fully verifying its remarkable detection performance and strong generalization ability.

Furthermore, we have supplemented additional comparative experiments to enrich empirical validations. For multivariate anomaly recognition tasks, we add ROC curves (Fig. 10), P-R curves (Fig. 11) and spatio-temporal complexity analyses (Fig. 12) of ATrans, DCdetector and TimesNet. For multivariate anomaly diagnosis tasks,

Table 2: Performance comparison of WADI and CICIDS before and after label correction.

| Method | WADI | | | | | | | | CICIDS | | | | | | | |
|---|---|---|---|---|---|---|---|---|---|---|---|---|---|---|---|---|
| | Pre | | Rec | | F1 | | VUS_ROC | | Pre | | Rec | | F1 | | VUS_ROC | |
| | Corrected | Original | Corrected | Original | Corrected | Original | Corrected | Original | Corrected | Original | Corrected | Original | Corrected | Original | Corrected | Original |
| MERLIN | 0.5113 | 0.1112 | 0.5111 | 0.5847 | 0.5088 | 0.1868 | 0.9431 | 0.9079 | 0.3560 | 0.1970 | 0.3706 | 0.1386 | 0.3542 | 0.1627 | 0.8620 | 0.8208 |
| OmniAnomaly | 0.7102 | 0.7370 | 0.5284 | 0.5513 | 0.3983 | 0.4420 | 0.6333 | 0.4234 | 0.4789 | 0.8188 | 0.4888 | 0.8187 | 0.4211 | 0.8187 | - | 0.9884 |
| MAD-GAN | 0.5879 | 0.7522 | 0.5045 | 0.5089 | 0.3505 | 0.3527 | 0.4606 | - | 0.2500 | 0.5910 | 0.5000 | 0.5001 | 0.3333 | 0.3337 | - | - |
| MSCRED | 0.4680 | 0.6013 | 0.4821 | 0.5668 | 0.4182 | 0.5264 | 0.4993 | 0.9921 | 0.4990 | 0.5645 | 0.4993 | 0.5510 | 0.4567 | 0.5263 | 0.9943 | 0.9928 |
| MTAD-GAT | * | * | * | * | * | * | * | * | 0.4558 | 0.5148 | 0.4857 | 0.5044 | 0.3810 | 0.3988 | - | - |
| GDN | 0.7194 | 0.7578 | 0.5074 | 0.5432 | 0.3504 | 0.4231 | 0.5231 | 0.3697 | 0.6739 | 0.8169 | 0.6111 | 0.7544 | 0.5726 | 0.7416 | 0.9257 | 0.9516 |
| CAE-M | 0.5965 | 0.5212 | 0.5081 | 0.5020 | 0.3621 | 0.3558 | 0.4785 | - | 0.4828 | 0.5216 | 0.4929 | 0.5100 | 0.4055 | 0.4343 | - | - |
| TranAD | 0.7737 | 0.6543 | 0.6570 | 0.5913 | 0.6161 | 0.5448 | 0.8751 | 0.7825 | 0.5498 | 0.4291 | 0.5001 | 0.4983 | 0.3340 | 0.3363 | - | - |
| TimesNet | 0.7685 | 0.7338 | 0.6172 | 0.6245 | 0.5545 | 0.5748 | 0.6787 | 0.9958 | 0.7227 | 0.8317 | 0.6915 | 0.8117 | 0.6803 | 0.8088 | 0.9953 | 0.9940 |
| ATrans | 0.7212 | 0.7613 | 0.6005 | 0.5848 | 0.5374 | 0.5005 | 0.7181 | 0.4944 | 0.7723 | 0.8722 | 0.7541 | 0.8440 | 0.7500 | 0.8410 | 0.9625 | 0.9722 |
| STADN | 0.7616 | 0.7108 | 0.6975 | 0.6718 | 0.6778 | 0.6558 | 0.9389 | 0.9010 | 0.5120 | 0.5833 | 0.5007 | 0.5029 | 0.3473 | 0.3447 | - | - |
| GRN | 0.5648 | 0.6256 | 0.5328 | 0.5702 | 0.4671 | 0.5170 | - | 0.9942 | 0.5201 | 0.5218 | 0.5005 | 0.5001 | 0.3396 | 0.3350 | - | - |
| DCdetector | 0.5351 | 0.7550 | 0.5075 | 0.5195 | 0.3870 | 0.3754 | - | - | 0.6162 | 0.6087 | 0.5861 | 0.5893 | 0.5574 | 0.5701 | 0.9887 | 0.9954 |
| DGINet | 0.7203 | 0.6207 | 0.7001 | 0.6015 | 0.6931 | 0.5850 | 0.9122 | 0.9107 | 0.5313 | 0.6204 | 0.5055 | 0.5128 | 0.3768 | 0.3727 | - | - |
| TS-GAT | 0.7253 | 0.7023 | 0.5536 | 0.5603 | 0.4485 | 0.4666 | 0.7541 | 0.5528 | 0.7475 | 0.8164 | 0.6383 | 0.7179 | 0.5935 | 0.6941 | 0.9011 | 0.9313 |
| HAO(E) | 0.9345 | 0.9018 | 0.9247 | 0.8779 | 0.9243 | 0.8760 | 0.9977 | 0.9999 | 0.9986 | 0.9999 | 0.9986 | 0.9999 | 0.9986 | 0.9999 | 0.9976 | 0.9997 |
| HAO(P) | 0.9516 | 0.8107 | 0.9509 | 0.7338 | 0.9508 | 0.7162 | 0.9999 | 0.9999 | 0.9987 | 0.9998 | 0.9987 | 0.9998 | 0.9987 | 0.9998 | 0.9977 | 0.9996 |
| HAO(H) | 0.9943 | 0.7968 | 0.9943 | 0.7898 | 0.9943 | 0.7885 | 0.9999 | 0.9999 | 0.9995 | 0.9995 | 0.9995 | 0.9995 | 0.9995 | 0.9995 | 0.9979 | 0.9995 |

**Symbol Definitions:** "$*$" indicates the evaluation failed due to **Out of Memory (OOM)**; "$-$" denotes **fragmented predictions** that do not meet the requirements for VUS_ROC computation.

we also conduct supplementary experiments on the above models based on HitRate and NDCG metrics (Fig. 13).

(5) **Supplementary Explanation on Performance Gap between WADI and CICIDS Datasets.** We note that HAO(H) achieves an extremely high original-label F1-score of 0.9995 on the CICIDS dataset, while it only reaches 0.7885 on the original WADI dataset. This distinct performance discrepancy stems from inherent dataset characteristics rather than unreasonable evaluation settings. CICIDS contains network traffic data corresponding to cyber attacks. Anomalies are triggered by definite attack behaviors with sharp and distinguishable feature patterns. The original labels sufficiently cover most prominent anomalies, leaving little space for label revision. Accordingly, HAO can achieve superior detection performance directly using original labels. In comparison, the WADI dataset is built for industrial control systems, where most anomalies correspond to subtle and gradual data drifts. A large number of gradual abnormal patterns are unmarked in the original dataset. Under original label settings, model detection results are severely penalized by false positives, leading to relatively low evaluation scores. After label revision, real anomaly boundaries are restored, and the full detection potential of the model is released, with the F1-score increasing from 0.7885 to 0.9943. This performance variation is highly consistent with the overall F1 distribution and improvement trends shown in Figs. 9(b, c), further confirming the rationality of our evaluation protocol and label revision strategy.

## References


[1] Xu, J. et al. Anomaly Transformer: Time Series Anomaly Detection with Association Discrepancy. In *Proc. Int. Conf. Learn. Represent.* (2022).

[2] Yang, Y. et al. DCdetector: Dual Attention Contrastive Representation Learning for Time Series Anomaly Detection. In *Proc. ACM SIGKDD Conf. Knowl. Discov. Data Min.* 1–17 (2023).

[3] Wu, H. et al. TimesNet: Temporal 2D-Variation Modeling for General Time Series Analysis. In *Proc. Int. Conf. Learn. Represent.* (2023).

[4] Chuadhry, M. A., Palleti, V. R. and Mathur, A. P. WADI: a water distribution testbed for research in the design of secure cyber physical systems. In *Proc. 3rd Int. Workshop Cyber-Phys. Syst. Smart Water Netw.* 25–28 (2017).

[5] Sharafaldin, I., Lashkari, A. H. and Ghorbani, A. A. Toward generating a new intrusion detection dataset and intrusion traffic characterization. In *Proc. 4th Int. Conf. Inf. Syst. Secur. Privacy (ICISSP)*. (2018).

[6] Deng, A. & Hooi, B. Graph neural network-based anomaly detection in multivariate time series. In *Proc. AAAI Conf. Artif. Intell.* **35**, 4027–4035 (2021).

[7] Zheng, Y. et al. Correlation-aware spatial–temporal graph learning for multivariate time-series anomaly detection. *IEEE T. Neur Net. Lear. Syst.* **35**, 11802–11816 (2024).

[8] Paparrizos, J. et al. Volume under the surface: a new accuracy evaluation measure for time-series anomaly detection. In *Proc. VLDB Endow.* **15**, 2774–2787 (2022).


**Revision 2:** **[Section 4 Experiment (Main Text: Page 2)]**

··· Full details of covariate control, hyperparameter tuning, and statistical analysis are provided in **Appendix Section 4.5**.···

**[Section 4.2 Evalutation Metrics (Main Text: Page 23)]**

··· Multivariate time series anomaly detection suffers from severe class imbalance, which tends to overestimate the overall model performance and fails to reflect practical anomaly detection capability. To mitigate evaluation bias caused by imbalanced data distribution, this study adopts the inverse class frequency weighting strategy to optimize weighted Precision(Pre), Recall(Rec) and F1-score [1, 2], defined as follows:

$$\text{Pre}_w = \frac{\sum_{i \in TP} s_i}{\sum_{i \in TP} s_i + \sum_{i \in FP} s_i}, \text{Rec}_w = \frac{\sum_{i \in TP} s_i}{\sum_{i \in TP} s_i + \sum_{i \in FN} s_i}, \text{F1-score}_w = 2 \cdot \frac{\text{Pre}_w \cdot \text{Rec}_w}{\text{Pre}_w + \text{Rec}_w}, \tag{5}$$

where $s_i$ denotes the inverse frequency weight assigned to each sample. Let $N_0$ be the number of normal samples, $N_1$ be the number of anomalous samples, and the total sample size is $N = N_0 + N_1$. Specifically, normal samples with larger proportion are assigned low weight $s_i = w_1 = N_1/N$, while sparse anomalous samples are assigned high weight $s_i = w_0 = N_0/N$ to balance their evaluation importance.

Furthermore, evaluation results derived from fixed thresholds are highly sensitive to threshold selection. To address this issue, we adopt Volume Under the Surface of ROC (VUS_ROC) for comprehensive model evaluation [3]. Given a monotonically increasing threshold sequence $Th = [Th_0, Th_1, \dots, Th_N]$ satisfying $0 = Th_0 < Th_1 < \cdots < Th_N = 1$, and a buffer length sequence $\mathcal{L} = [\ell_0, \ell_1, \dots, \ell_L]$ satisfying $0 = \ell_0 < \ell_1 < \cdots < \ell_L = \ell$, the VUS_ROC is defined as follows:

$$\text{VUS\_ROC} = \frac{1}{4} \sum_{w=1}^{L} \sum_{k=1}^{N} \Delta^{(k,w)} \cdot \Delta^{w}, \tag{6}$$

where $\Delta^{(k,w)} = \Delta^k_{TPR_{\ell_w}} \cdot \Delta^k_{FPR_{\ell_w}} + \Delta^k_{TPR_{\ell_{w-1}}} \cdot \Delta^k_{FPR_{\ell_{w-1}}}$, $\Delta^k_{FPR_{\ell_w}} = \text{FPR}_{\ell_w}(Th_k) - \text{FPR}_{\ell_w}(Th_{k-1})$, $\Delta^k_{TPR_{\ell_w}} = \text{TPR}_{\ell_w}(Th_{k-1}) + \text{TPR}_{\ell_w}(Th_k)$, $\Delta^w = |\ell_w - \ell_{w-1}|$. ···

**[Section 4.3.1 Comparative Experiments (Main Text: Page 24-26)]**

··· *For instance, the F1-score of OmniAnomaly drops from 0.6640 to 0.5758, with a decrease of 0.0883.* Transformer-based models including TimesNet[5], ATrans[6], DCdetector[7] and TranAD[8] achieve significant performance gains after label correction, demonstrating their strong ability to capture temporal dependencies and anomalies. However, these models lack robustness to label noise and depend highly on accurate supervision. *This demonstrates that the HAO series models are less sensitive to the limitations of anomaly labeling and they can achieve effective performance on higher baseline models.* ···

··· *In addition, MERLIN is more sensitive to interpretable anomalies due to its detection mechanism and its precision improvement reaches as high as 0.2596 after correction.* The precision of ATrans, DCdetector and TimesNet increases by 0.0155, 0.0875 and 0.0255 respectively after label correction. These improvements reflect that the label correction method proposed in this paper provides reliable and accurate supervision signals and helps these models give full play to their inherent detection performance. *This indirectly reflects that the HAO series models can effectively analyze the original structural variations caused by anomalies and eliminate false anomaly signals, thus achieving a more interpretable anomaly detection mechanism.* ···

··· VUS_ROC performance before and after label correction is shown in Fig. 9(j, k, l). The HAO series models maintain VUS_ROC values above 0.98 throughout label correction and exhibit small performance fluctuations across datasets. The DCdetector model achieves considerable performance improvement but lacks sufficient stability across different scenarios. It is worth noting that ATrans and TimesNet achieve gains in F1 score, precision, and recall in Fig. 9(b, e, h) while suffering a decline in VUS_ROC in Fig. 9(k). This contradiction arises because point-wise indicators for discrete classification directly benefit from refined anomaly boundaries generated by label correction. VUS_ROC evaluates global ranking performance under continuous thresholds and is less disturbed by threshold selection strategies. It thus reveals structural limitations of models that rely on static attention, frequency domain periodic modeling, or contrastive learning priors and show weak generalization ability under clean labels. The density distribution in Fig. 9(l) confirms this trend. The overall average VUS_ROC decreases slightly from 0.82 to 0.81, which is caused by performance degradation of unstable models. ···

**[Section 4.3.2 Comparative Analysis Based on ROC Curves and AUC Values (Main Text: Page 26-27)]**

··· *DGINet is suitable for high-noise industrial time series and achieves an AUC of 0.9331 on the Synthetic dataset, but only 0.7001 on the WADI dataset.* ATrans models temporal association discrepancies through a two-branch anomaly-

attention mechanism, achieving an AUC value of 0.8261 on the Synthetic dataset with prominent temporal features and 0.7943 on the high-dimensional and strongly coupled PSM dataset. *GDN is adept at modeling dependencies among variables and ranks first among baseline models on both the CICIDS and GECCO datasets, with AUC values of 0.6331 and 0.6111, respectively.* · · ·

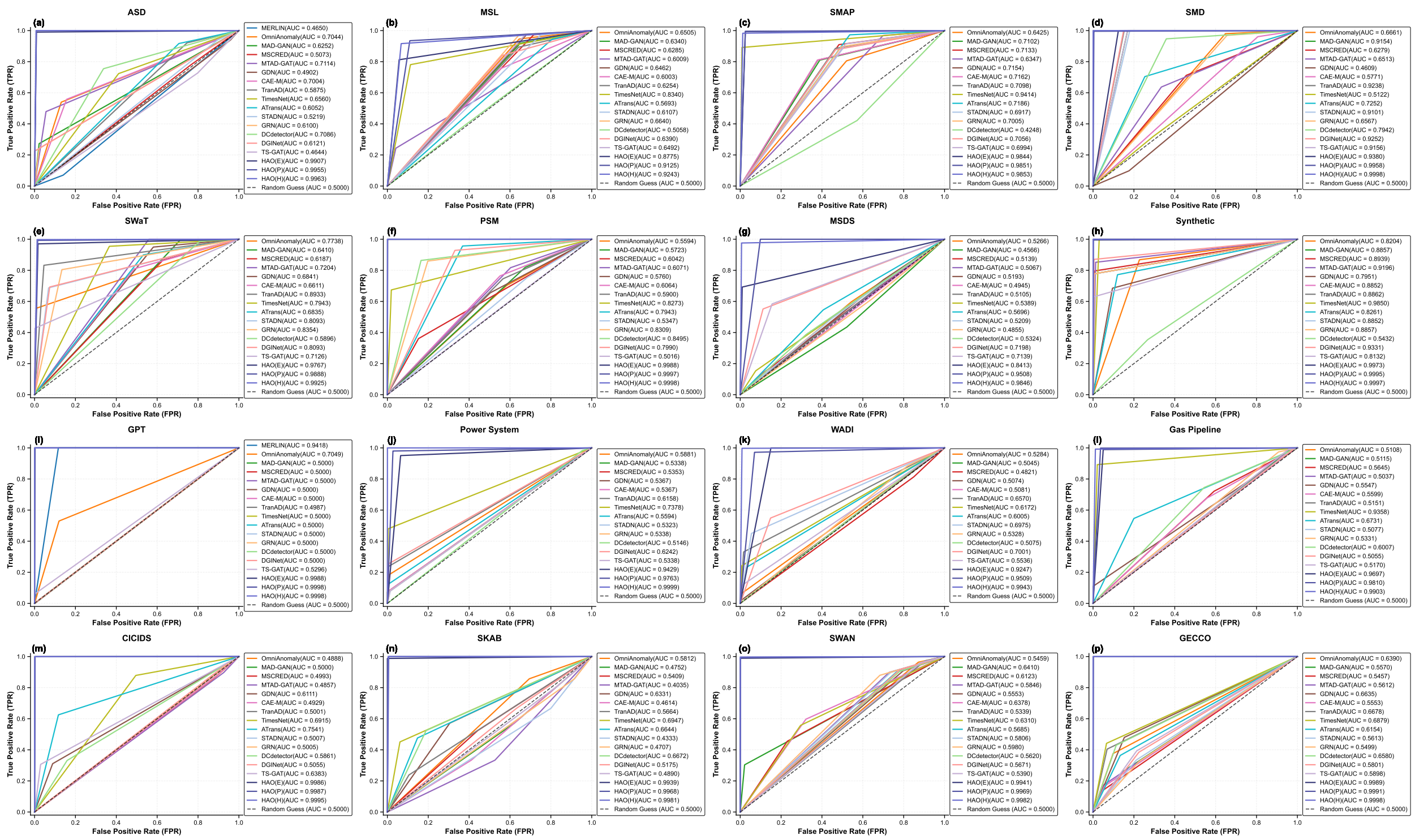


Figure 10: Comparison of ROC Performance of Multivariate Anomaly Detection Models Across Multiple Datasets

**[Section 4.3.3 Comparative Analysis Based on Precision-Recall Curves and AP Values (Main Text: Page 27-28)]**

· · · *The main reason for this trend is that the baseline models lack the ability to disentangle high-dimensional features and struggle to adapt to the complex spatio-temporal coupling relationships of diverse anomalies, thereby impairing the synergistic performance between precision and recall.* On the CICIDS dataset, TimesNet, ATrans, and DCdetector obtain APs of 0.6224, 0.7141, and 0.5581. All three models degrade with higher dimensionality and anomaly complexity, revealing challenges from high-dimensional intricate patterns instead of architectural representation bottlenecks.· · ·

**[Section 4.3.5 Spatio-Temporal Complexity and Reconstruction Error Analysis (Main Text: Page 30-31)]**

· · · *Among all comparison methods,* TimesNet achieves the best performance with a reconstruction error of 0.0039. · · ·

· · · *the inference time and memory usage of the HAO model series remain at a moderate level.* TimesNet has inference time and memory usage close to HAO(H). *Its frequency-domain transformation and 2D convolution bring higher time overhead but reasonable memory consumption. Despite similar spatio-temporal complexity, HAO(H) achieves better performance.* · · ·

**[Section 4.4.1 Comparative Performance Analysis of Anomaly Diagnosis (Main Text: Page 37-38)]**

· · · *In contrast, the average HitRate@600% of all baseline models is below 70%.* ATrans and TimesNet achieve stable average HitRate@600% values ranging from 61.6% to 63.5% across all experimental datasets yet remain within the low-performance range of traditional comparison models. *Furthermore, in terms of anomaly diagnosis quality, as shown in Fig. 13(c), the average NDCG@600% of the HAO model in Euclidean space is 89.0%, that in the Poincaré ball space is 94.2% and that in hyperbolic space is 97.6%,* while the average NDCG@600% of baseline models is below 50%. · · ·

· · · ATrans captures long-range temporal dependencies but neglects multivariate spatial topology. DCdetector screens local anomalies without global spatio-temporal logic. TimesNet extracts multi-scale temporal features but fails to

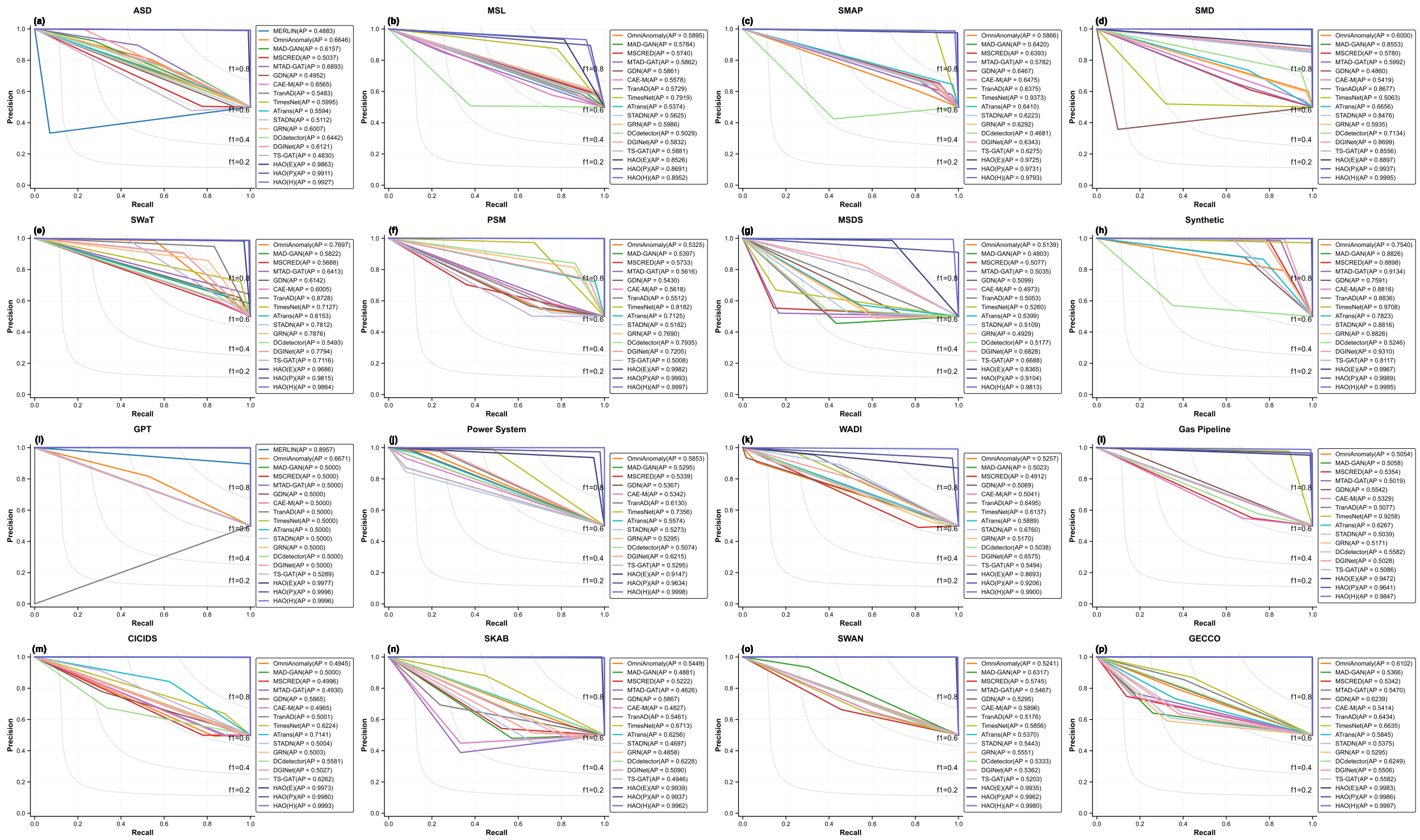


Figure 11: Comparison of P–R Curves for Multivariate Anomaly Detection Models

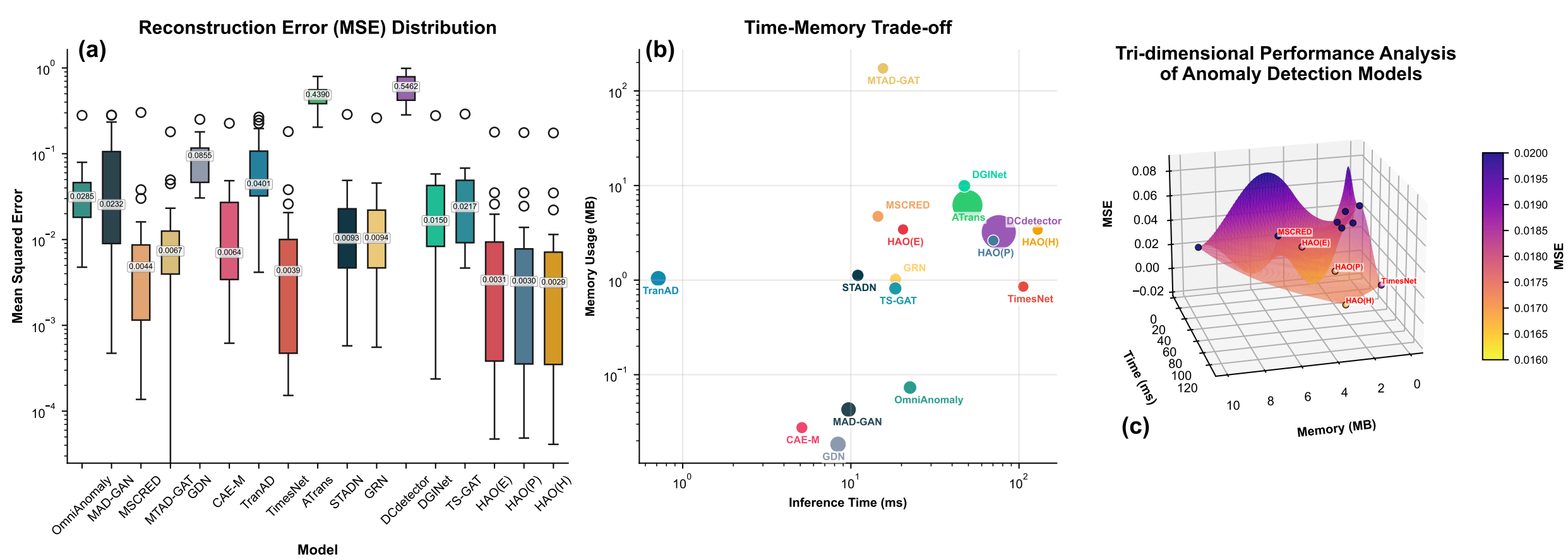


Figure 12: Spatio-Temporal Complexity–Reconstruction Error and Three-Dimensional Performance Analysis of Multivariate Anomaly Detection Models

decouple multivariate coupled anomalies. *They can only identify anomaly locations based on sensor information during anomaly reconstruction· · ·*

**[Section 4.3.4 Ablation Study and Stability Analysis (Main Text: Page 29-30)]**

*· · · In contrast, the Euclidean space cannot fully characterize the complex spatio-temporal coupling relationships among variables and data representation is prone to distortion, which limits the ability to identify labeling errors and results in a relatively limited label correction effect.* · · ·

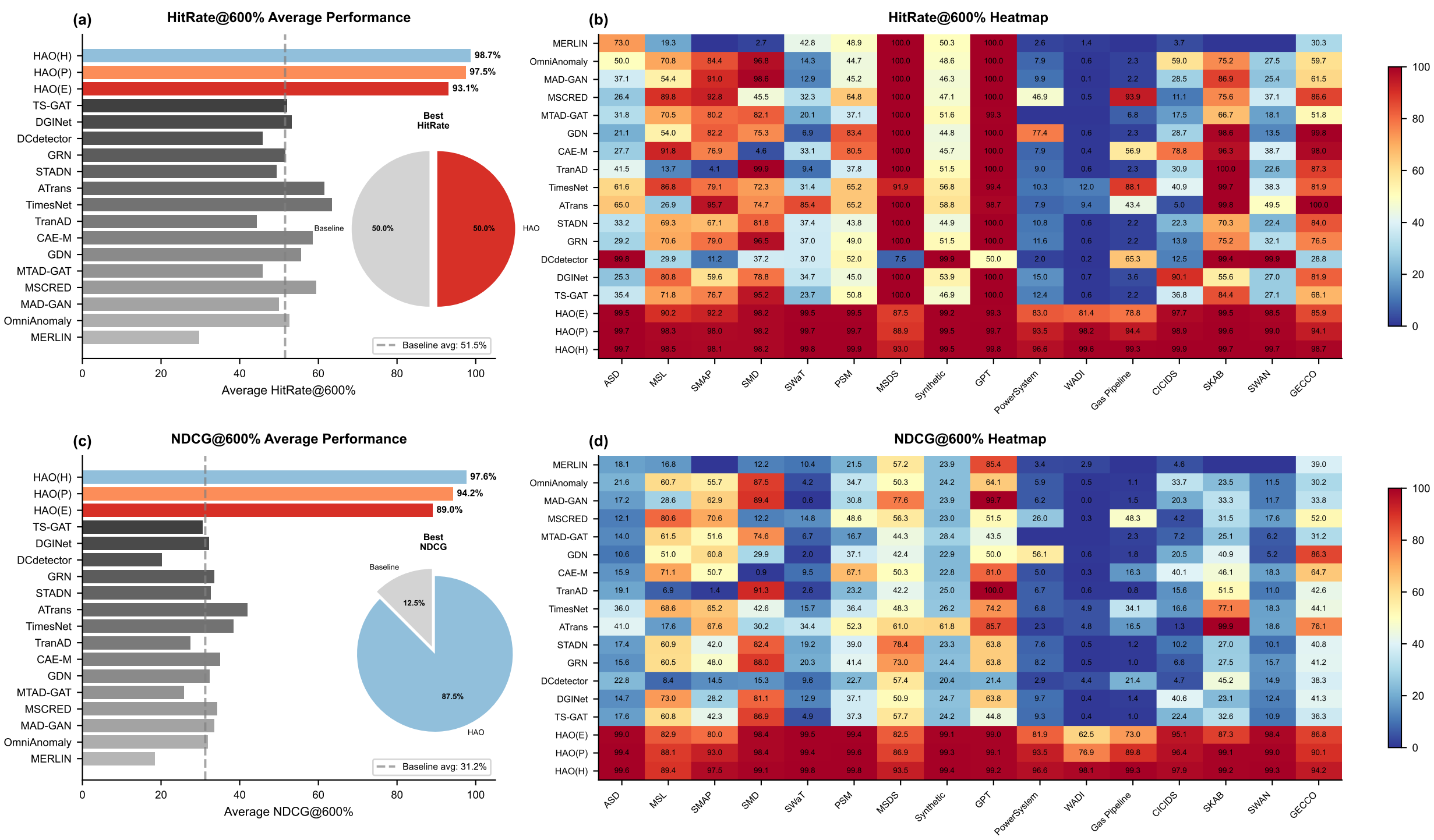


Figure 13: Comparative Analysis of Anomaly Diagnosis Performance for Multivariate Anomaly Detection Models

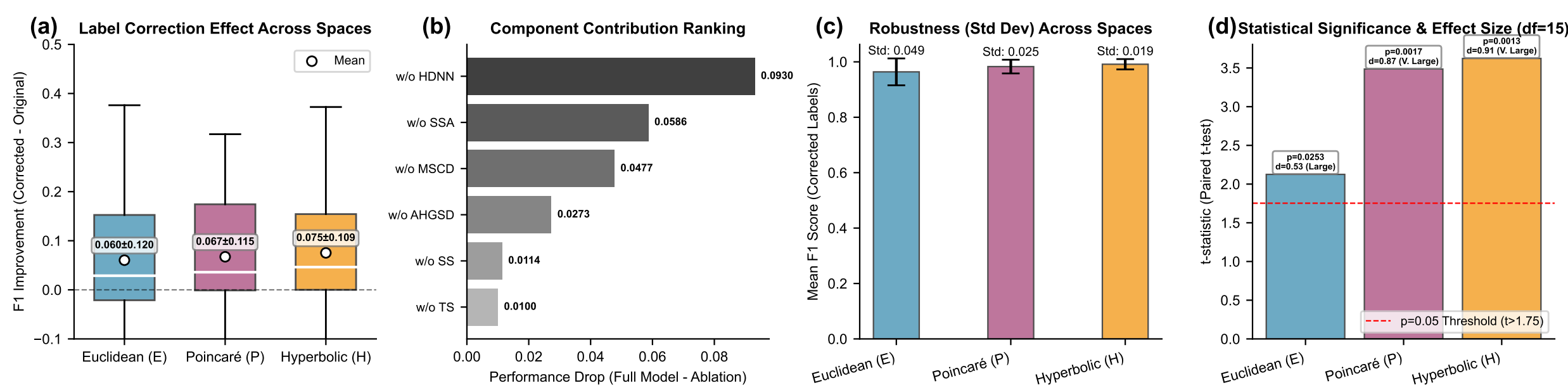


Figure 14: *Ablation Study and Stability Analysis of the HAO Model. Fig. 14(b) shows performance changes after removing each core component, where w/o indicates removal of the corresponding module: w/o HDNN removes the geometric learning module; w/o MSCD removes the multi-level signal parsing attention mechanism module; w/o AHGSD removes the spatio-temporal adaptive hyperbolic graph structure discovery module; w/o SSA removes the natural graph dynamic sparsification module; w/o TS removes the multivariate temporal natural structure discovery module; w/o SS removes the multivariate coupling structure discovery module.* Fig. 14(d) shows the results of two-sided paired $t$-tests and corresponding effect sizes for performance differences between models in three geometric spaces and the baseline model. The effect size is reported as Cohen's $d$, computed using the formula: $d = \bar{D}/SD_D$, where $\bar{D}$ is the mean of performance differences between the proposed model and the baseline across all datasets, and $SD_D$ is the standard deviation of these differences.

**[Section 4.5 Covariate Analysis and Statistical Methods (Supplementray Information: Page 7)]** All covariates that may influence model performance and experimental outcomes were systematically tested, controlled, and reported in this study. These covariates are categorized into three groups: data-level covariates, model-level covariates, and experimental design covariates.

Data-level covariates included the sliding window size ( $W \in \{5, 15, 30, 70, 90, 110\}$ ), multivariate dimensionality (sensor count, ranging from 8 to 127), anomaly difficulty level (low/medium/high), anomaly type (point, regional, sequential), and sequence length. Min–max normalization was applied to all features, as detailed in Section 4.1. The

sliding window size was varied for sensitivity analysis (Section 4.3.7) but fixed to an optimal value for the main experiments.

Model-level covariates comprised three embedding spaces (Euclidean, Poincaré ball, Hyperbolic), the adaptive curvature parameter ( $K = -1/\zeta^2$ ) in Hyperbolic space (Section 4.3.9), hidden dimension, learning rate, AdamW optimizer with step scheduling (Section 4.4), core model components (HDNN, MSCD, AHGSD, SSA, TS, SS), and anomaly scoring algorithms (STS, POT, TTS, NSTS) (Section 4.3.8). Hyperparameters were tuned via grid search and fixed across all datasets to ensure fair comparison. Additionally, ablation studies were conducted by systematically removing individual components to quantify their contributions (Section 4.3.4).

Experimental design covariates included the validation set ratio (ranging from 0.1 to 0.9; Section 4.3.6), label condition (original labels vs. corrected labels; Section 4.3.1), evaluation metrics (F1-score, Precision, Recall, AUC, AP, VUS_ROC, HitRate@600%, NDCG@600%), and 5 independent repeated runs. All experimental settings and computational environments were standardized (Section 4.4) to ensure reproducibility and unbiased comparison across models.

All statistical comparisons were performed using two-sided paired t-tests (Section 4.3.4). Results are reported as mean ± standard deviation (SD), test statistics, degrees of freedom, exact p-values, 95% confidence intervals (CI), and effect sizes (Cohen's d). Model stability was quantified using standard deviation and the coefficient of variation (Sections 4.3.4, 4.3.6, and 4.3.7).

**[Section 4.6 Comparison Models (Supplementray Information: Page 7-8)]**

Table 3: Description of Multivariate Anomaly Detection Comparison Models

| **Number** | **Method** | **Threshould Selection($\tau$)** | **Model Description** | **Interpretation Method** | **Relesed Year** |
|---|---|---|---|---|---|
| ... | ... | ... | ... | ... | ... |
| 9 | TimesNet[5] | **I** | TimesNet simultaneously captures the intraperiod and interperiod variations of time series by transforming 1D sequential data into structured 2D tensors and adopting parameter-efficient 2D inception blocks. Specifically, the model first adaptively discovers multiple dominant periods via Fast Fourier Transform (FFT) on the frequency domain, then reshapes the 1D time series into a set of 2D tensors to disentangle and unify intraperiod variations within each cycle and interperiod variations across different cycles. It then utilizes a shared inception block with multi-scale 2D kernels to extract comprehensive temporal 2D variations from the reshaped tensors, and fuses the multi-period representations adaptively according to the amplitude weights derived from FFT.<br>TimesNet employs a residual stacked architecture with repeated TimesBlocks as its backbone, enabling end-to-end representation learning for general time series analysis tasks. For forecasting, imputation, classification, and anomaly detection, the model generates task-specific outputs through tailored projection heads, and identifies anomalies or prediction deviations based on reconstruction or forecasting errors, achieving state-of-the-art performance across five mainstream time series analysis tasks in a unified framework. | **A** | 2022 |
| 10 | Anomaly Transformer (**ATrans**)[6] | **I** | ATrans simultaneously models the prior-association and series-association of time series by reforming the vanilla self-attention into a novel two-branch Anomaly-Attention mechanism. Specifically, the model first generates prior-association using a learnable Gaussian kernel to reflect the adjacent-concentration inductive bias of time points, and computes series-association via standard self-attention to capture global temporal dependencies from the raw sequence. It then calculates the Association Discrepancy as the symmetric KL divergence between these two distributions, which serves as an inherent distinguishable criterion between normal and anomalous time points.<br>The anomaly score is computed by jointly fusing the normalized negative Association Discrepancy and the reconstruction error through element-wise multiplication, which amplifies the separability between normal and anomalous samples. Finally, the model is optimized under a minimax strategy to alternately adjust the prior-association and series-association, further enhancing the discriminability of anomaly scores and enabling accurate unsupervised detection and localization of anomalies in multivariate time series. | **A** | 2023 |
| ... | ... | ... | ... | ... | ... |
| 13 | DCdetector[7] | **I** | DCdetector simultaneously learns discriminative permutation-invariant representations for time series anomaly detection by employing a dual-branch contrastive structure with dual attention operated on multi-scale patched sequences. Specifically, the model first normalizes input multivariate time series via instance normalization and adopts channel-independent patching to divide each dimension into patches, then constructs patch-wise attention to model dependencies across patches and in-patch attention to capture relations within patches under multi-scale settings, and uses upsampling to restore two branches to the same temporal length. It optimizes the network purely by contrastive loss based on symmetric KL divergence between the patch-wise and in-patch representations with stop-gradient for asynchronous training, avoiding model collapse thanks to the intrinsic asymmetric design of dual branches. The anomaly score is computed as the symmetric KL divergence between representations from the two branches, where anomalies yield significantly higher discrepancy values than normal points. Finally, time points with anomaly scores exceeding a predefined threshold are judged as anomalies, enabling effective and robust unsupervised detection and localization for both point-wise and pattern-wise anomalies in time series. | **A** | 2023 |
| ... | ... | ... | ... | ... | ... |

## References


[1] Deng, A. & Hooi, B. Graph neural network-based anomaly detection in multivariate time series. In *Proc. AAAI Conf. Artif. Intell.* **35**, 4027–4035 (2021).

[2] Zheng, Y. et al. Correlation-aware spatial–temporal graph learning for multivariate time-series anomaly detection. *IEEE T. Neur Net. Lear. Syst.* **35**, 11802–11816 (2024).

[3] Paparrizos, J. et al. Volume under the surface: a new accuracy evaluation measure for time-series anomaly detection. In *Proc. VLDB Endow.* **15**, 2774–2787 (2022).

[4] Li, D. et al. MAD-GAN: multivariate anomaly detection for time series data with generative adversarial networks. In *Proc. Int. Conf. Artif. Neural Netw. (ICANN)*, 703–716 (2019).

[5] Wu, H. et al. TimesNet: Temporal 2D-Variation Modeling for General Time Series Analysis. In *Proc. Int. Conf. Learn. Represent.* (2023).

[6] Xu, J. et al. Anomaly Transformer: Time Series Anomaly Detection with Association Discrepancy. In *Proc. Int. Conf. Learn. Represent.* (2022).

[7] Yang, Y. et al. DCdetector: Dual Attention Contrastive Representation Learning for Time Series Anomaly Detection. In *Proc. ACM SIGKDD Conf. Knowl. Discov. Data Min.* 1–17 (2023).

[8] Tuli, S. et al. TranAD: Deep transformer networks for anomaly detection in multivariate time series data. In *Proc. VLDB Endow.* **15**, 1201–1214 (2022).

**3. The datasets used are individually well-known in the community, but the way they are handled raises questions. The artificial recombination of normal and anomalous data for test and validation sets, rather than strict temporal splitting, contradicts standard practice for time series evaluation. The sliding window argument in the rebuttal preserves local continuity but does not address the global temporal ordering violation.**

**Response 3:** We sincerely appreciate the reviewers' targeted and professional valuable comments. The reviewer pointed out that we restructured normal and abnormal samples during the construction of test and validation sets, instead of adopting the strictly chronological splitting strategy commonly used in time series tasks, which may be inconsistent with the standard experimental specifications in time series anomaly detection. We fully agree that temporal integrity and temporal causal constraints are the core principles of time series anomaly detection (TSAD). In the following, we provide systematic and detailed explanations combined with the core design mechanism, training and testing logic of our model, as well as supplementary experimental compensation strategies.

(1) **During the training phase, this study completely preserves and solidifies global temporal dependencies and strictly complies with core specifications of temporal modeling.** Based on the hyperbolic adaptive pre-training framework, we feed complete, untruncated and unsorted original time series into the model. The model fully learns the long-term stable spatial coupling correlations and global temporal evolution rules among multivariate sensors, and further constructs fixed multivariate coupling graphs and temporal graphs to establish stable global topological prior knowledge. Sufficient learning of global temporal information ensures that the model can effectively model the overall system operation rules, long-period evolution characteristics and global anomaly patterns, which meets the modeling requirements for global temporal dependence in regional anomaly detection and fundamentally conforms to standard temporal modeling principles. In addition, we adjust the sliding window length to fully explore and adapt to long-period abnormal features of time series data, so as to further enhance the model's perception capability towards complex temporal anomalies.

(2) **The sliding window-based block inference adopted in the test phase does not abandon or destroy global temporal information, but it serves as a reasonable design to realize unified multi-task inference and adapt to practical engineering scenarios.** This work simultaneously implements three coupled tasks including anomaly recognition, anomaly diagnosis and anomaly interpretation. Direct inference with full-length long sequences will easily lead to accumulated redundant noise[1, 2, 3], temporal distribution shift and ambiguous spatio-temporal anomaly localization, resulting in inconsistent inference granularity across tasks and difficulties in aligning anomaly tracing and causal interpretation.

Accordingly, we adopt sliding window strategy to partition test data and conduct collaborative multi-task inference under unified spatio-temporal granularity, which effectively guarantees the consistency and traceability of results for anomaly recognition, diagnosis and interpretation. It is worth emphasizing that window-based inference only limits the local input range of each single forward pass. The global topological structure and temporal prior learned during training continuously impose constraints and calibration effects. All local temporal inferences are guided by global temporal knowledge without breaking the inherent temporal correlation of event-level anomalies. Furthermore, we introduce hyperbolic representation learning to strengthen the embedding of subtle abnormal features via its superior negative curvature geometric property. This method well compensates for potential feature insufficiency caused by local window inference and improves the detection performance on faint temporal anomalies.

(3) **We specially design a fine-grained spatio-temporal label correction module to compensate for temporal integrity deviation caused by window sampling and guarantee reliable experimental results.** Window-based block inference inevitably leads to abnormal event truncation, ambiguous boundary annotation and temporal shift. To eliminate such interference on quantitative evaluation, we adopt the multivariate anomaly spatio-temporal coordinate positioning algorithm to uniformly revise mislabeling, missing labels and labeling delays introduced by window sampling. It reconstructs complete regional anomaly evaluation results from segmented window-level outputs, alleviates the loss of temporal integrity, and ensures that all final metrics can truly and objectively reflect the model's detection capability for holistic temporal abnormal events.

## References


[1] Wu, R. J. and Keogh, E. J. Current time series anomaly detection benchmarks are flawed and are creating the illusion of progress.

[2] Paparrizos, J. et al. Volume under the surface: a new accuracy evaluation measure for time-series anomaly detection. In *Proc. VLDB Endow.* **15**, 2774–2787 (2022).

[3] Huet, A. et al. Local evaluation of time series anomaly detection algorithms. In *Proc. ACM SIGKDD Conf. Knowl. Discov. Data Min.* 1–11 (2022).

**4. The theoretical content is presented with considerable formalism but limited depth. Classical results from probability theory are restated in the appendix without being formally connected to the paper's specific convergence claims. The adaptive curvature, a key theoretical motivation, converges to the same fixed value of 1.0 across all datasets, a result that the paper reports but does not discuss, even though it directly contradicts the need for learning curvature at all.**

**Response 4:** We sincerely thank the reviewers for their valuable and constructive comments. The reviewers pointed out key deficiencies in the theoretical part of this paper, including redundant formal expressions, insufficient theoretical depth, weak logical correlation between classical probabilistic conclusions in the appendix and model convergence proofs, unreasonable interpretation of adaptive curvature convergence and inadequate illustration on the necessity of adaptive curvature learning. We fully acknowledge these deficiencies. We have supplemented the theoretical framework, built coherent logical links and further interpreted the internal working mechanisms. The detailed point-by-point responses are presented as follows.

(1) **Supplementary connection between classical probability theories and model convergence conclusions.** To address this concern, we have fully revised the theoretical content in the appendix. We establish coherent logical links between the following probability theorems and the parameter updating, topology iteration and representation convergence processes of our hyperbolic adaptive learning framework, so as to lay a solid theoretical foundation for model convergence.

(a) **Theorem 3 (Lindeberg–Lévy Central Limit Theorem[1]):** Let $\xi_1, \xi_2, \cdots, \xi_n, \cdots$ be a sequence of independent and identically distributed (i.i.d.) random variables satisfying $E\xi_k = \mu, D\xi_k = \sigma^2$. Define the standardized sum as $\zeta_n = \frac{1}{\sigma\sqrt{n}}\sum_{k=1}^{n}(\xi_k - \mu)$. If $0 < \sigma^2 < \infty$, the limiting distribution of $\zeta_n$ satisfies:

$$\lim_{n\to\infty} P\left\{\zeta_n < x\right\} = \frac{1}{\sqrt{2\pi}}\int_{-\infty}^{x} e^{-\frac{t^2}{2}}\mathrm{d}t. \tag{7}$$

This theorem ensures the asymptotic normality of multivariate time series reconstruction errors in iterative model optimization and verifies that such errors converge stably in line with Gaussian distribution. It provides an essential theoretical prerequisite for stable hyperbolic embedding parameter updating and steady iterative optimization of spatio-temporal topological structures.

(b) **Theorem 4 (Bernoulli's Law of Large Numbers[1]):** Let $\mu_n$ denote the occurrence frequency of event $A$ within $n$ independent Bernoulli trials and $p$ represent the occurrence probability of event $A$ in a single trial. For any $\varepsilon > 0$, the following formula holds:

$$\lim_{n\to\infty} P\left\{\left|\frac{\mu_n}{n} - p\right| < \varepsilon\right\} = 1. \tag{8}$$

The law of large numbers reveals the statistical stability of event frequency in repeated trials. In this work, it provides theoretical support for sliding window sampling of multivariate time series and statistical estimation of anomaly probability. It explains that continuous window sampling enables the model to capture stable statistical features of spatio-temporal structures and acquire reliable anomaly probability estimation, and effectively eliminates interference brought by random errors from single-window sampling.

(c) **Theorem 5 (Gärtner–Ellis inequality[1]):** Let $\{\xi_i\}$ be a sequence of independent random variables with $D\xi_i = \sigma_i^2 < \infty, (i = 1, 2, \cdots, \infty)$ and $\{C_n\}$ be a non-increasing positive constant sequence. For any positive integers $m, n$ satisfying $m < n$ and arbitrary $\varepsilon > 0$, we have:

$$P\left\{\max_{m\le j\le n}\left|\sum_{i=1}^{j}(\xi_i - E\xi_i)\right| \ge \varepsilon\right\} \le \frac{1}{\varepsilon^2}\left(C_m^2\sum_{j=1}^{m}\sigma_j^2 + \sum_{j=m+1}^{n}C_j^2\sigma_j^2\right). \tag{9}$$

This theorem constrains the range of cumulative errors generated in hyperbolic embedding updating and incremental topology iteration. It strictly proves that the parameter updating error of the adaptive curvature module achieves bounded convergence during iterative training, which provides core theoretical guarantee for the convergence and stability of the entire adaptive hyperbolic learning framework.

(2) **In response to the insightful comment that curvature values converge uniformly to a fixed value of 1.0 on all datasets(Fig. 15)**, which seems seemingly inconsistent with the original intention of adaptive curvature learning[2], we provide further clarified explanations in separate subsections below.

(a) Definition of fixed convergence target. The unified convergence of curvature parameters in the output module to 1.0 will not weaken the effectiveness of adaptive curvature learning. Instead, it is preset as the final geometric optimization calibration target in this study. This setting only restricts the final

convergence state of the model, without imposing rigid fixed constraints on curvature parameters or limiting dynamic parameter adjustment in the whole training process.

(b) Effectiveness verification of adaptive curvature learning. Core modules of the model including the input module and intermediate spatio-temporal linear modules retain full dynamic adjustment capacity for adaptive curvature throughout training. These modules can autonomously fit and optimize curvature parameters according to heterogeneous geometric features and diverse spatio-temporal dependencies of different datasets, which fully fulfills the theoretical motivation and core value of adaptive curvature learning in exploring dataset-specific geometric characteristics.

(c) Hierarchical logic of geometric learning. The model adopts a mutually supportive hierarchical geometric learning paradigm with clear functional division. Adaptive curvature learning in multi-layer intermediate modules focuses on fitting personalized spatio-temporal geometric attributes of different datasets and alleviating cross-dataset heterogeneity. By contrast, the unified convergence of the output module towards curvature 1.0 aims to build standardized unified geometric benchmarks for subsequent bimodal fusion scoring and anomaly recognition, so as to ensure consistency and stability of downstream decision-making processes.

(d) Resolution of the above contradiction. In conclusion, the adaptive learning process and fixed final convergence mechanism are not mutually exclusive. Adaptive curvature optimization enables the model to mine inherent geometric rules and adapt to heterogeneous data characteristics, while fixed convergence targets standardize evaluation criteria for downstream anomaly detection tasks. This elaborate hierarchical design resolves the apparent conflict between adaptive geometric optimization and fixed-value convergence, and balances the flexibility of geometric representation and the standardization of model output results.

## References


[1] Kallenberg, O. Foundations of Modern Probability. New York: Springer, 1997.

[2] Fu, X.C. et al. ACE-HGNN: Adaptive Curvature Exploration Hyperbolic Graph Neural Network. In *Int. Conf. Data Min.*, 111–120 (2021).


**Revision 4: [Section 2 Relevant Theorems and Lemmas (Supplementary Information: Page 2-3)]**

***Theorem 3 (Lindeberg–Lévy Central Limit Theorem[1]):*** *Let $\xi_1, \xi_2, \cdots, \xi_n, \cdots$ be a sequence of independent and identically distributed (i.i.d.) random variables satisfying $E\xi_k = \mu, D\xi_k = \sigma^2$. Define the standardized sum as $\zeta_n = \frac{1}{\sigma\sqrt{n}} \sum_{k=1}^{n} (\xi_k - \mu)$. If $0 < \sigma^2 < \infty$, the limiting distribution of $\zeta_n$ satisfies:*

$$\lim_{n\to\infty} P\left\{\zeta_n < x\right\} = \frac{1}{\sqrt{2\pi}} \int_{-\infty}^{x} e^{-\frac{t^2}{2}} \mathrm{d}t. \tag{10}$$

This theorem ensures the asymptotic normality of multivariate time series reconstruction errors in iterative model optimization and verifies that such errors converge stably in line with Gaussian distribution. It provides an essential theoretical prerequisite for stable hyperbolic embedding parameter updating and steady iterative optimization of spatio-temporal topological structures.

***Theorem 4 (Bernoulli's Law of Large Numbers[1]):*** *Let $\mu_n$ denote the occurrence frequency of event $A$ within $n$ independent Bernoulli trials and $p$ represent the occurrence probability of event $A$ in a single trial. For any $\varepsilon > 0$, the following formula holds:*

$$\lim_{n\to\infty} P\left\{\left|\frac{\mu_n}{n} - p\right| < \varepsilon\right\} = 1. \tag{11}$$

The law of large numbers reveals the statistical stability of event frequency in repeated trials. In this work, it provides theoretical support for sliding window sampling of multivariate time series and statistical estimation of anomaly probability. It explains that continuous window sampling enables the model to capture stable statistical features of spatio-temporal structures and acquire reliable anomaly probability estimation, and effectively eliminates interference brought by random errors from single-window sampling.

***Theorem 5 (Gärtner–Ellis inequality[1]):*** *Let $\{\xi_i\}$ be a sequence of independent random variables with $D\xi_i = \sigma_i^2 < \infty, (i = 1, 2, \cdots, \infty)$ and $\{C_n\}$ be a non-increasing positive constant sequence. For any positive integers $m, n$ satisfying $m < n$ and arbitrary $\varepsilon > 0$, we have:*

$$P\left\{\max_{m\le j\le n} \left|\sum_{i=1}^{j} (\xi_i - E\xi_i)\right| \ge \varepsilon\right\} \le \frac{1}{\varepsilon^2} \left(C_m^2 \sum_{j=1}^{m} \sigma_j^2 + \sum_{j=m+1}^{n} C_j^2 \sigma_j^2\right). \tag{12}$$

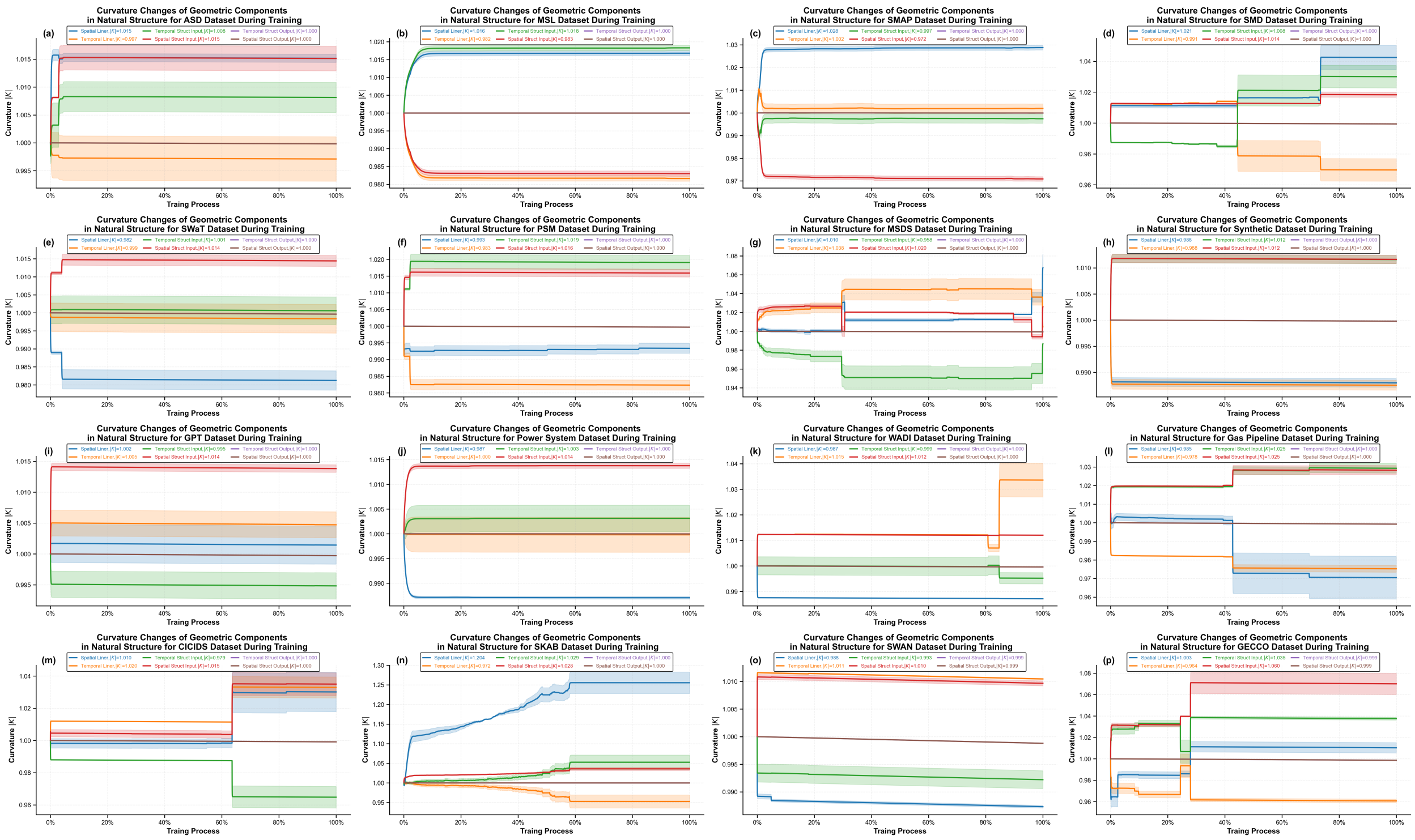


Figure 15: Training Stability and Curvature Variation Analysis of the HAO Model

This theorem constrains the range of cumulative errors generated in hyperbolic embedding updating and incremental topology iteration. It strictly proves that the parameter updating error of the adaptive curvature module achieves bounded convergence during iterative training, which provides core theoretical guarantee for the convergence and stability of the entire adaptive hyperbolic learning framework.

**[Section 4.3.9 Training Stability Analysis (Main Text: Page 35-36)]** $\cdots$ *Benefiting from the synergy between dynamic graph structure learning and geometric parameter optimization, the HAO model automatically adapts to the heterogeneous geometric features of different datasets and eventually achieves consistent convergence.* Specifically, the multi-module hierarchical curvature learning of the full model is fully implemented throughout the training process. The geometric modules at the input and middle layers maintain sufficient adaptive curvature adjustment margins to match diverse natural geometric distributions of data [2]. The final output module uniformly converges to a curvature of 1.0, which serves as the standardized geometric calibration criterion for downstream anomaly scoring and feature fusion. *From the perspective of anomaly detection difficulty, the curvature fluctuation amplitude of geometric components on datasets with high detection difficulty is significantly larger than that on low-difficulty datasets.* $\cdots$

## References


[1] Kallenberg, O. Foundations of Modern Probability. New York: Springer, 1997.

[2] Fu, X.C. et al. ACE-HGNN: Adaptive Curvature Exploration Hyperbolic Graph Neural Network. In *Int. Conf. Data Min.*, 111–120 (2021).


**5. The TSAD field has matured considerably in recent years. The community now values clean experimental protocols, honest baselines, appropriate metrics and focused contributions over architectural complexity. This manuscript would benefit substantially from engaging with that direction: one clear contribution, properly measured, on well-established benchmarks, compared against current state-of-the-art under identical conditions.**

**Response 5:** We greatly appreciate the reviewer's accurate summary of the development trends in the time series anomaly detection (TSAD) field and valuable constructive comments. We fully recognize that the TSAD research community has become increasingly mature, with the research focus gradually shifting from elaborate model structure stacking toward concise and original innovations, standardized experimental workflows, scientific evaluation metrics,

and fair baseline comparisons. Following this advanced research paradigm, we have comprehensively revised and improved the entire manuscript in accordance with the above core suggestions.

Combined with the discussions in **Response 1** , the multi-module architecture proposed in this paper is not a redundant stacking of model structures. Instead, it comprises differentiated functional modules specifically designed to address the unique technical challenges in the respective stages of multivariate time series anomaly recognition, anomaly diagnosis, and anomaly interpretation. These modules cooperate closely and couple with each other, realizing the integrated and systematic modeling of multivariate time series anomaly recognition, diagnosis, and interpretable analysis under a unified inference granularity. In strict accordance with the reviewer's requirements of refining a single core innovation, standardizing the experimental evaluation system, adopting canonical benchmark datasets, and conducting fair and state-of-the-art comparisons, we have systematically improved and optimized the overall experimental framework of the manuscript. The specific improvements are detailed as follows:

(1) We refine the statement of core innovations and remove redundant contents. The manuscript focuses on the core academic contribution of realizing multivariate time series anomaly recognition, diagnosis and interpretable analysis based on multivariate spatio-temporal original structure. Meanwhile, we have correspondingly optimized the contents of the abstract and introduction.

(2) We introduce the state-of-the-art robust evaluation metric VUS_ROC to systematically compare model performance before and after label correction[1, 2]. This strategy effectively alleviates evaluation bias caused by label noise and guarantees the authenticity and reliability of experimental results, as detailed in **Response 2** . Meanwhile, for multivariate anomaly recognition, we supplement ROC curves, P-R curves, and spatial-temporal complexity analyses of ATrans[3], DCdetector[4], and TimesNet[5]. For multivariate anomaly diagnosis, we further conduct comparative experiments in terms of HitRate and NDCG metrics. These supplementary experiments enrich the experimental dimensions and significantly strengthen the persuasiveness of our results.

(3) This multivariate anomaly detection study is deeply inspired by existing geometric topology research[6] and anomaly visualization methods[2, 7]. It also serves as a vital exploration to advance multivariate anomaly detection toward intelligent interpretation of anomaly causes.

We once again express our sincere gratitude to the reviewer for all valuable suggestions and guidance.

## References


[1] Paparrizos, J. et al. Volume under the surface: a new accuracy evaluation measure for time-series anomaly detection. In *Proc. VLDB Endow.* **15**, 2774–2787 (2022).

[2] Huet, A. et al. Local evaluation of time series anomaly detection algorithms. In *Proc. ACM SIGKDD Conf. Knowl. Discov. Data Min.* 1–11 (2022).

[3] Yang, Y. et al. DCdetector: Dual Attention Contrastive Representation Learning for Time Series Anomaly Detection. In *Proc. ACM SIGKDD Conf. Knowl. Discov. Data Min.* 1–17 (2023).

[4] Xu, J. et al. Anomaly Transformer: Time Series Anomaly Detection with Association Discrepancy. In *Proc. Int. Conf. Learn. Represent.* (2022).

[5] Wu, H. et al. TimesNet: Temporal 2D-Variation Modeling for General Time Series Analysis. In *Proc. Int. Conf. Learn. Represent.* (2023).

[6] Battiston, F., Bick, C., Lucas, M. et al. Collective dynamics on higher-order networks. *Nat Rev Phys* **8**, 146–159 (2026).

[7] Wu, R. J. and Keogh, E. J. Current time series anomaly detection benchmarks are flawed and are creating the illusion of progress. *IEEE T. Knowl. Data En.* **35**, 2421–2429 (2021).

# REVISION OF MANUSCRIPT IN NATURE COMMUNICATIONS #NCOMMS-24-82438C

## Explanation of Revisions

We are submitting a revised version of our paper, "Hyperbolic Adaptive Spatial Perception Multivariate Time Series Anomaly Detection" (ID: #NCOMMS-24-82438C). We sincerely thank the reviewers for their valuable and constructive comments. We thoroughly revise the paper in accordance with these suggestions, provide detailed responses to all review comments and add new analytical content to further strengthen our research. We hereby declare that this paper has not been published elsewhere in any form.

## 1 Reviewer #2 (Remarks to the Author):

**The authors'responses have addressed my concerns.**

**Response :** We sincerely thank the reviewer for their positive feedback and for recognizing that our responses have adequately addressed their concerns.

## 2 Reviewer #4 (Remarks to the Author):

**I appreciate the authors for the detailed response. The current version of manuscript looks nice from my side. In this case, I suggest to accept this paper.**

**Response :** We sincerely thank the reviewer for their thorough review, positive feedback, and valuable time. We are thrilled that the reviewer is satisfied with our revisions and supports the acceptance of our manuscript.
